# Supplementary material for: Nickase fidelity drives EvolvR-mediated diversification in mammalian cells
Source: Nat Commun. 2025 Apr 19;16:3723. doi: 10.1038/s41467-025-58414-0 (PMC12009436; doi:10.1038/s41467-025-58414-0)
Supplement: Supplementary file 1 — Supplementary Information [file 41467_2025_58414_MOESM1_ESM.pdf]

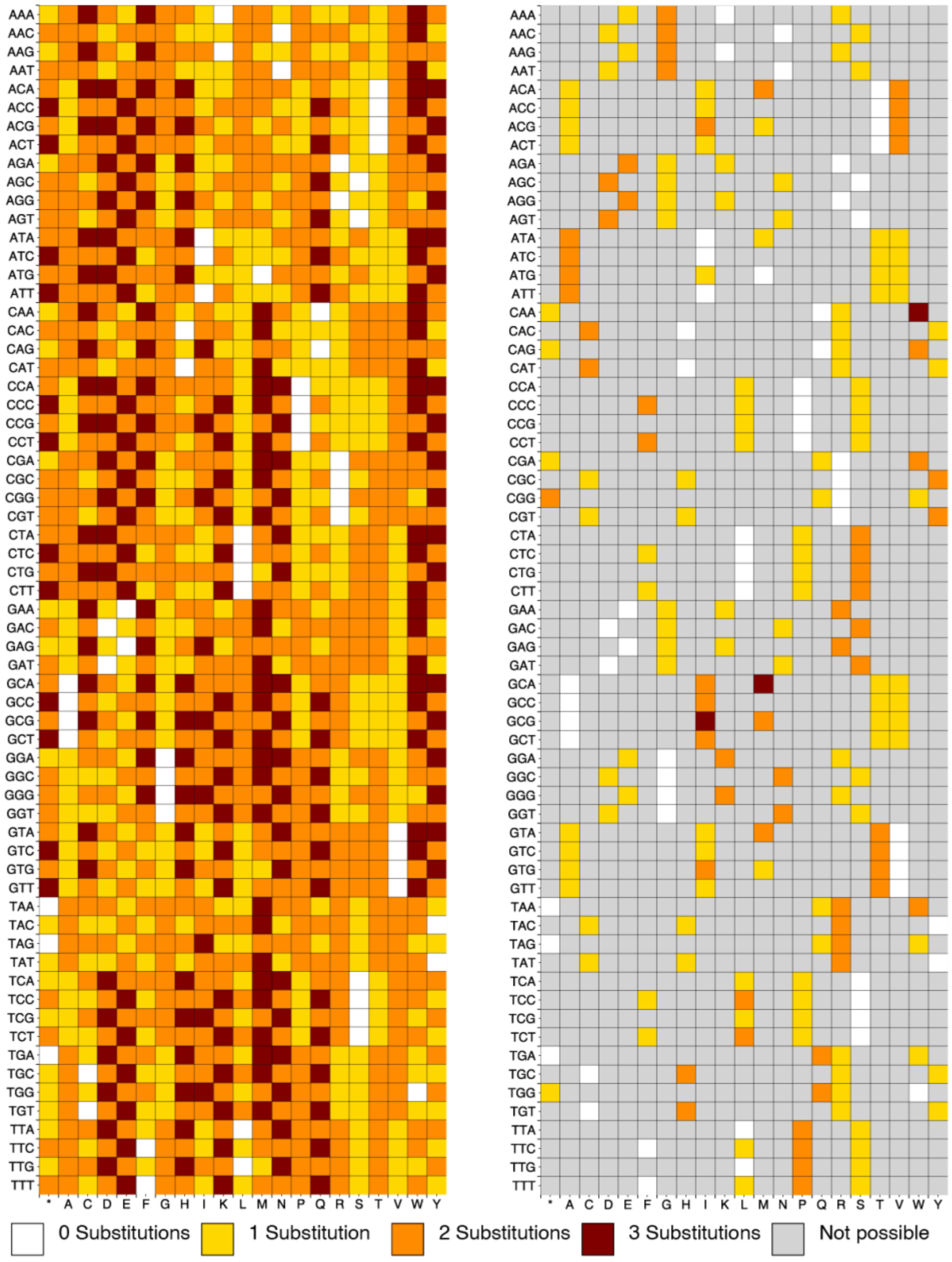

Supplementary Figure 1. **Transition mutations enable a minority of missense mutations.** This heatmap shows the minimum number of substitutions necessary for converting each of 64 codons to encode any of the other 19 amino acids or stop codons. The left heatmap represents

missense mutations accessible with all 12 substitutions. The right heatmap represents missense mutations accessible using only transition mutations.

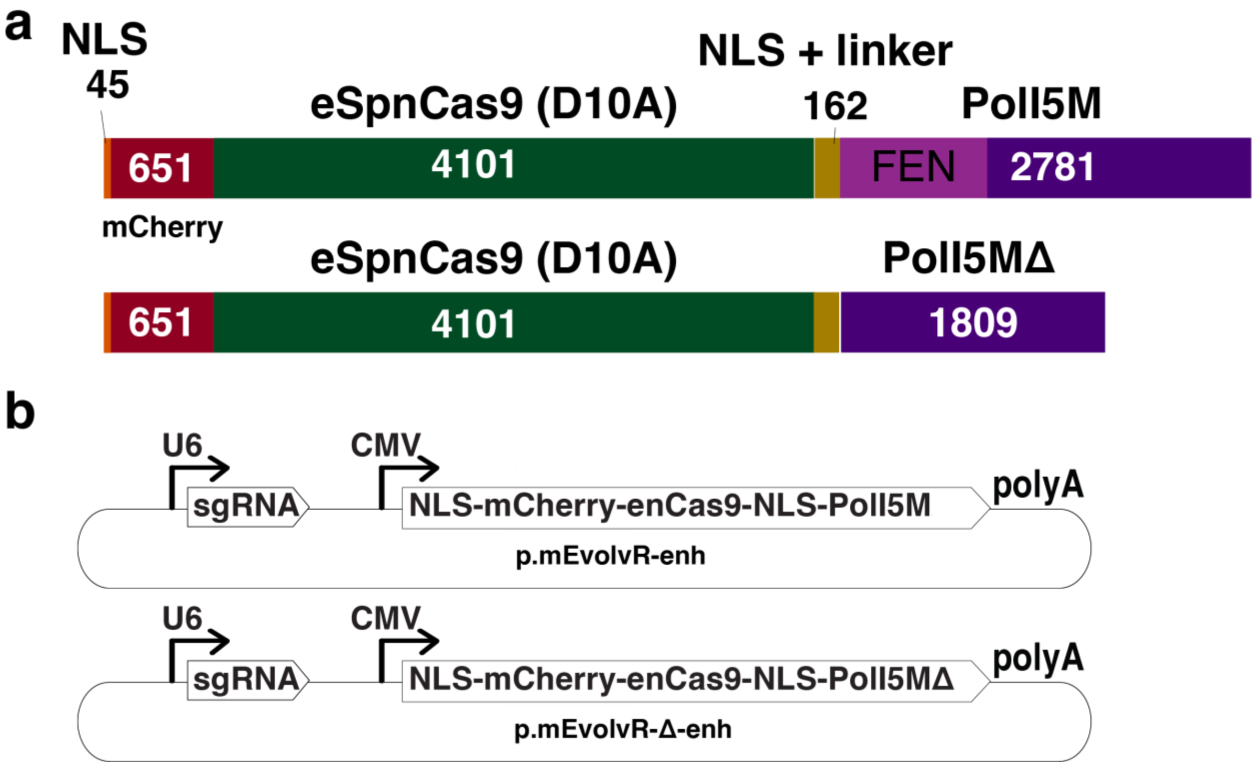

Supplementary Figure 2. **Plasmids for expression of EvolvR in human cells. a,** Schematics representing the length and composition of EvolvR variants tested. eSpnCas9 represents SpCas9 with mutations D10A, K848A, K1003A, and R1060A introduced. Numbers represent length in bp. PolI5MΔ indicates the deletion of the first 325 amino acids from PolI5M. **b,** Map depicts a plasmid encoding EvolvR for expression in mammalian cells was constructed as an mCherry-tagged enhanced nicking Cas9 flanked by two nuclear localization sequences and fused to PolI5M's Klenow fragment with or without PolI5M's flap endonuclease domain.

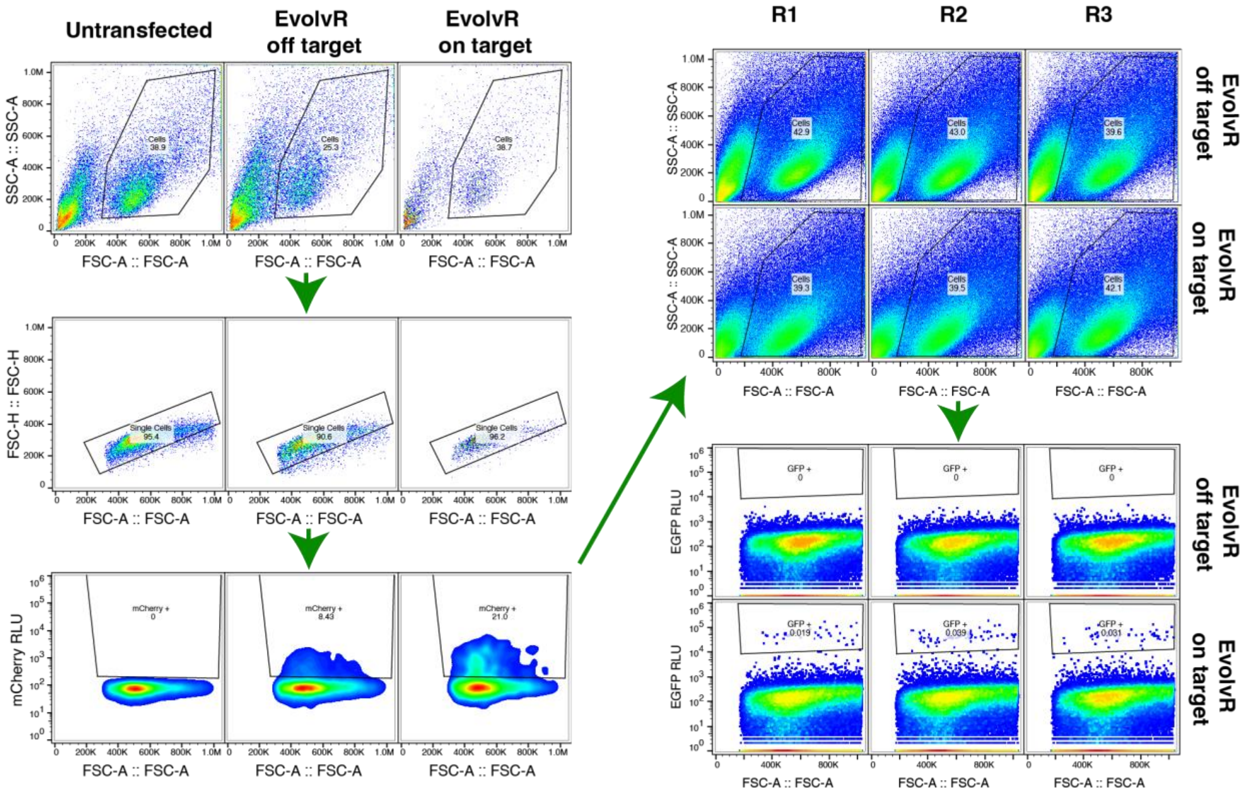

Supplementary Figure 3. **Gating strategy for measuring the frequency of BFP to GFP edits in BFP HEK293 cells.** Recently transfected cells are gated for single cells by conventionally gating cells for a linear relationship between height and area. Transfection efficiency is quantified by measuring the frequency of mCherry positive cells against an untransfected negative control. Cells are cultured and later harvested to measure the frequency of GFP positive cells. In order to include as close to 100% of GFP positive cells present in the population at the end of the experiment in the analysis and minimize error due to stochastic exclusion of rare cells, the bulk cell population in each biological replicate (R1, R2, R3) is analyzed for the presence of GFP positive cells rather than excluding doublets.

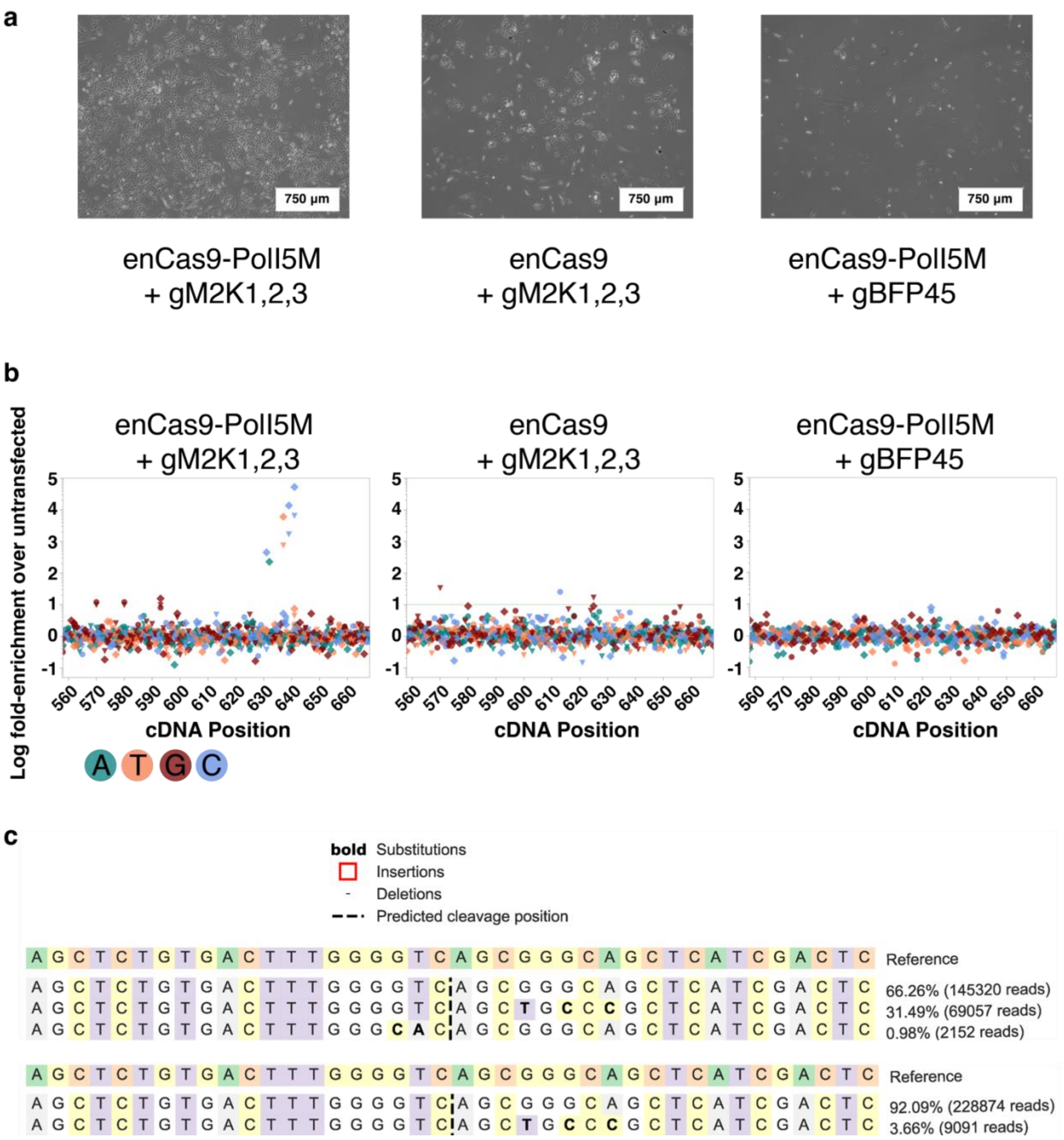

Supplementary Figure 4. **EvolvR generated selumetinib-resistant MAP2K1 substitution variants. a,** Differences in cell growth under selective media were visible by microscope 36 days after initiation of selection. **b,** EvolvR targeted to exon 6 of MAP2K1 generated substitution variants that were enriched compared to untransfected cells by 10-50,000-fold after 40 days of selection, while enCas9 alone targeted to exon 6 generated fewer substitution variants enriched by at least 10-fold and EvolvR targeted to a nonexistent BFP gene generated no enriched variants. Triangles, diamonds, and circles correspond to different biological replicates. Green, orange, red, and blue points represent A, T, G, and C variants respectively. Green dashed lines mark the 10-fold enrichment threshold. **c,** Representative CRISPRESSO allele tables showing

enriched alleles containing multiple substitutions in cell populations generated by EvolvR targeted to exon 6 of MAP2K1.

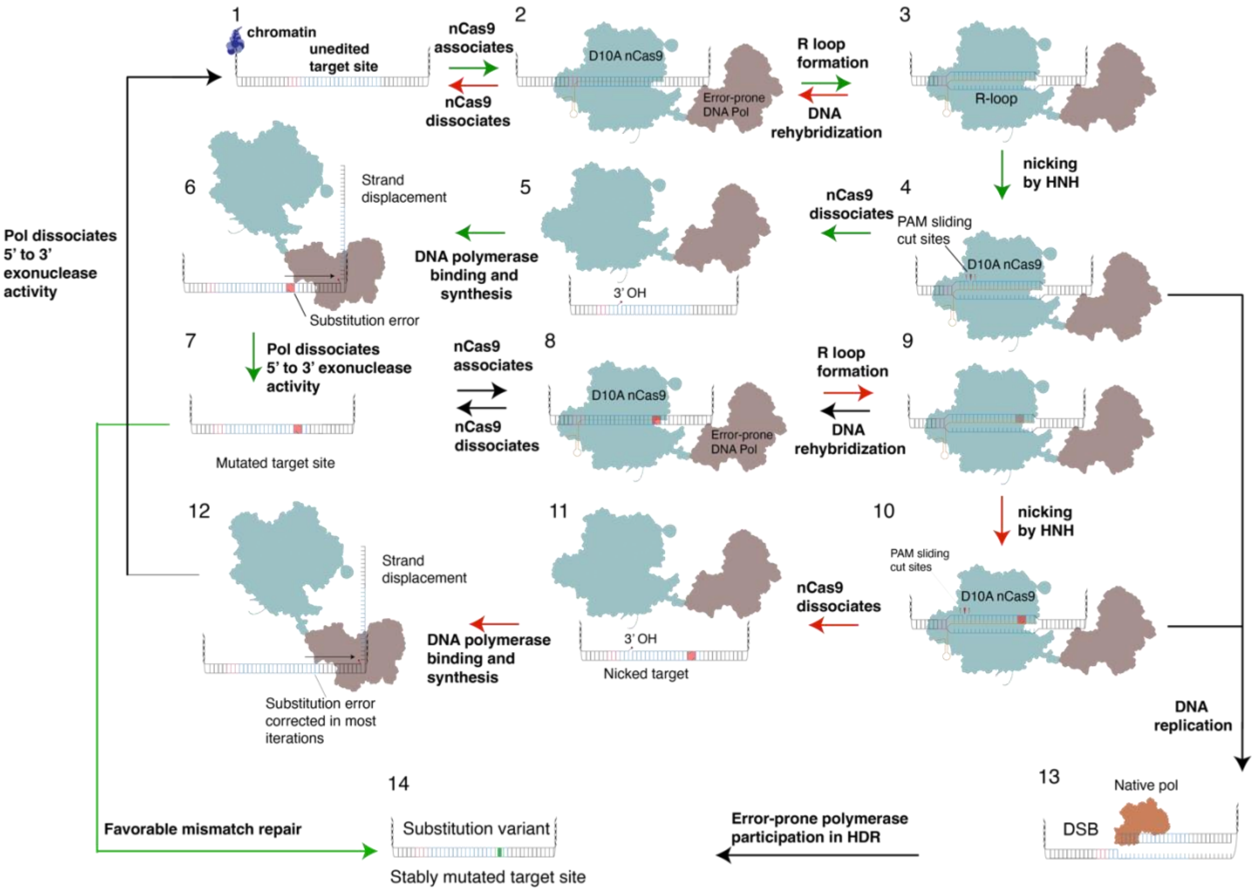

Supplementary Figure 5. **Mismatch tolerance bias model of EvolvR mutagenesis.** The schematic above depicts a proposed mechanism for explaining variability in EvolvR's performance across different gRNA and nickases. Unedited target sites (1) undergo initial recognition by nCas9 (2) , enabling the initiation of R loop formation (3). Complete R loop formation enables nicking Cas9 to generate a single-stranded break (4). Cas9 dissociates after DNA-DNA hybridization (5), allowing PolI to initiate strand-displacing DNA synthesis (6). After resection of the 5' flap by native exonucleases (7), nCas9 has the potential to reassociate, nick, dissociate, and perform additional rounds of strand-displacing synthesis (8,9,10,11,12). At steps in which nCas9 is bound to a nicked substrate (4,10), the replication fork can collide with and displace Cas9 resulting in a single-ended double stranded break (13), initiating homology-directed repair which PolI may participate in. Alternatively, when a mismatched but ligated target locus exists at the point of DNA replication (7), mutations may be stably installed as substitution variants in one of the two daughter cells (14). Green arrows represent steps expected to synergize with EvolvR-mediated mutagenesis, while red arrows represent steps expected to impede EvolvR-mediated mutagenesis. EvolvR cartoon is adapted from Halperin et al. 2018[1].

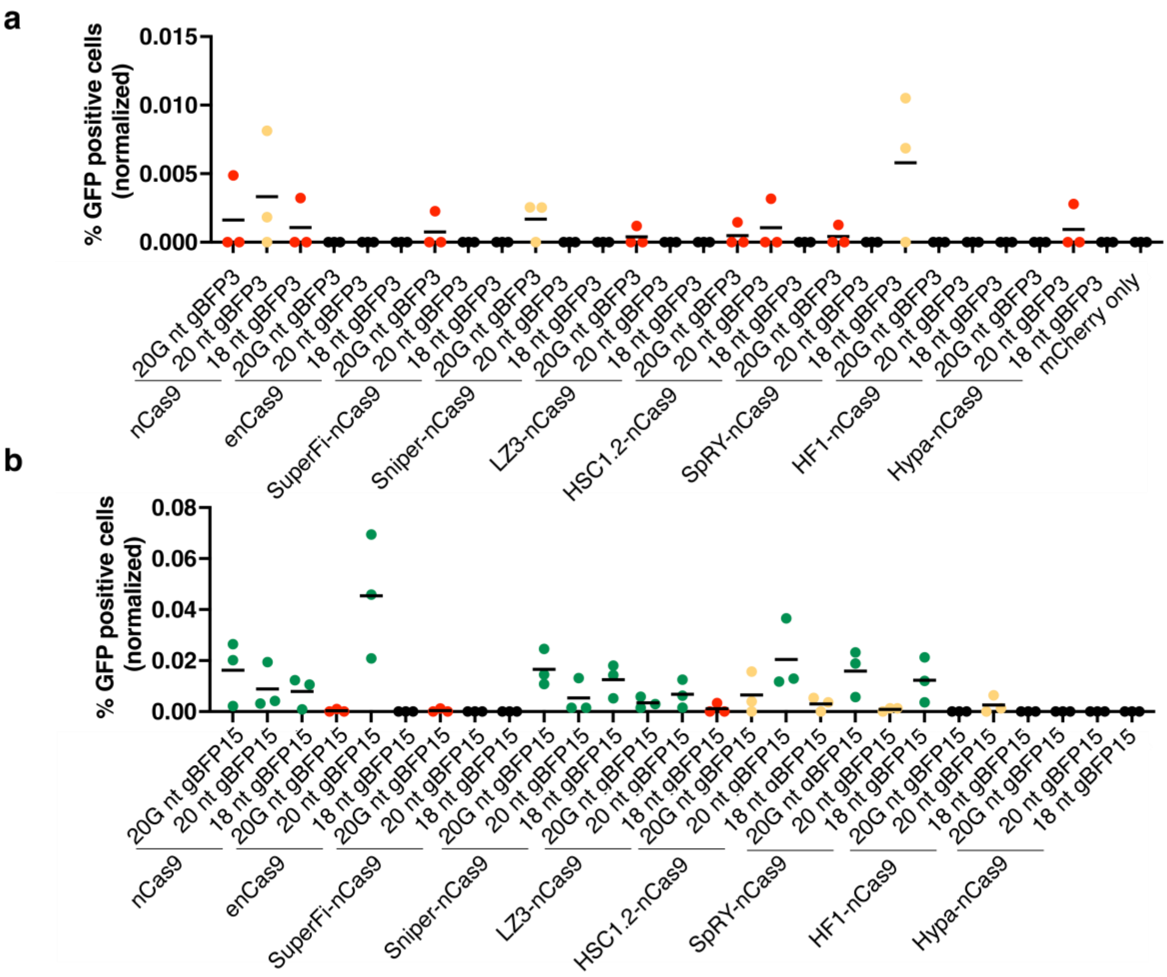

Supplementary Figure 6. **SpCas9 nickases enable variable mutagenicity depending on gRNA design and nuclease engineering. a,b** EvolvR performance varies depending on gRNA spacer sequence, gRNA design and nuclease variant used. The percentage of GFP positive cells generated by each construct was measured by flow cytometry and normalized by transfection efficiency. Green, yellow, red, and black dots represent that three, two, one, or zero out of three biological replicates contained GFP positive cells, respectively. Black lines represent mean % GFP positive cells. gBFP3 and gBFP15 indicate the use of gRNAs enabling nicking 3 bp and 15 bp upstream of H67. 20G indicates the use 20 nt gRNAs with additional 5' terminal guanines.

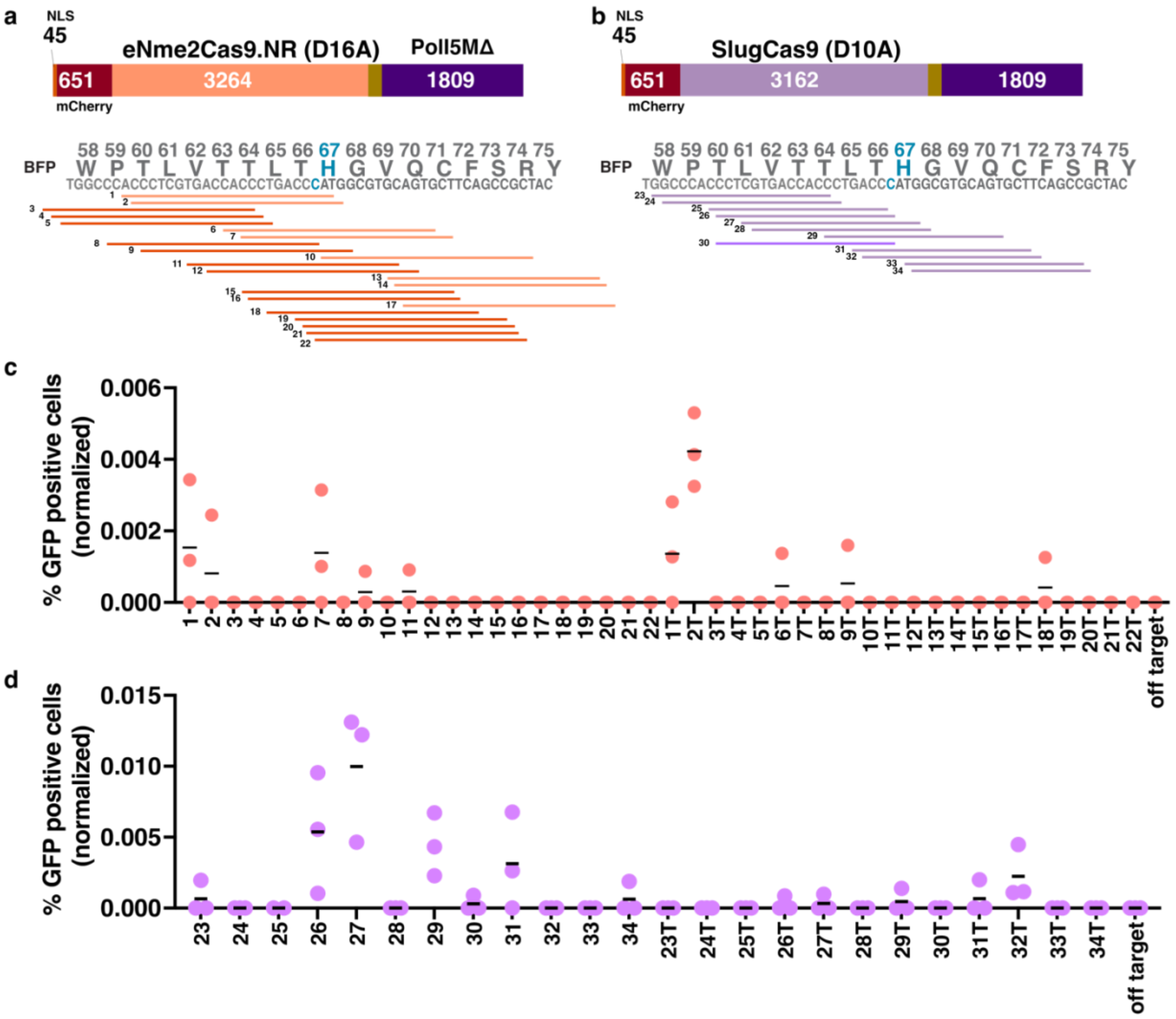

Supplementary Figure 7. **PAM-flexible nucleases (D10A) enable targeted mutagenesis of BFP when fused to PolI5MΔ. a,b** Schematics representing the composition of EvolvR variants tested. Numerical values indicate the length of individual components. eNme2Cas9.NR represents a nickase derived from a PAM flexible variant of *Neisseria meningitidis* (Nme2) Cas9 containing mutations D16A, K104T, D152A, F260L, A263T, A303S, D451V, E932K, N1031S, R1033G, K1044R, Q1047R, V1056A. SlugCas9 represents a PAM nickase derived from *Staphylococcus lugdunensis* containing mutation D10A. **c,d** eNme2-nCas9-PolI5MΔ and Slug-nCas9 enable guide-dependent mutagenesis of BFP. The diagram depicts a BFP fluorescent reporter gene for EvolvR mutagenesis an a panel of spacer sequences generating nicks in either strand and either direction from the nucleotide encoding the H67Y missense mutation. Light orange and purple lines overlap with unique target sequences in the sense strand of BFP, while dark orange and purple lines overlap with unique target sequences in the antisense strand of BFP. Substitution of a T for a C in H67 yields expression of GFP instead of BFP. The percentage of GFP positive cells generated by each construct was measured by flow cytometry and normalized by transfection efficiency. Dots represent individual biological replicates. Black lines represent mean % GFP positive cells. gRNA numbers correspond to the spacers labelled in the diagrams

in **a,b**. The absence or presence of a “T” after the gRNA label indicates the use of truncated gRNAs. eNme2-nCas9-PolI5MΔ was complexed with 23 nt and 21 nt truncated gRNAs, while Slug-nCas9-PolI5MΔ was complexed with 20 nt and 18 nt truncated gRNAs.

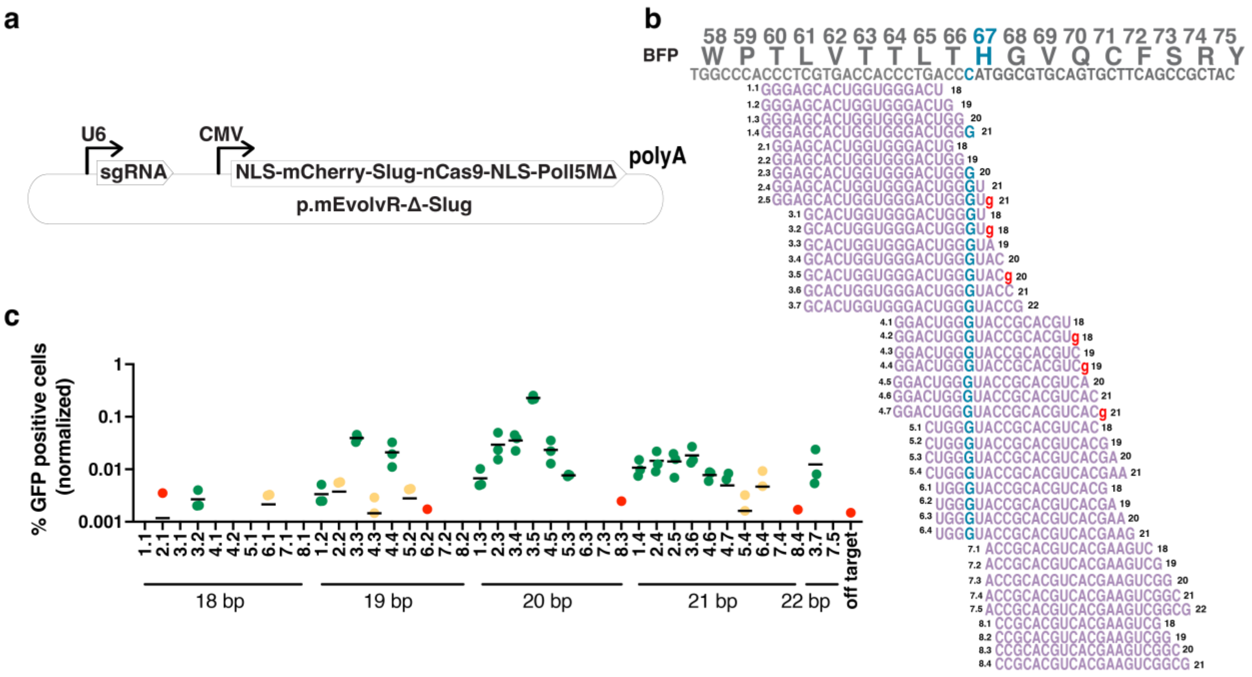

Supplementary Figure 8. **Optimal Slug-nCas9-PolI5MΔ mutagenicity requires at least 20 bp of complementarity with target site. a,** Map depicts a plasmid encoding expression of EvolvR comprising an mCherry-tagged SlugCas9 (D10A) fused to PolI5MΔ at its C terminus. **b,** Diagram depicts a panel of 39 gRNAs targeting 8 different spacer sequences in the BFP gene. Decimal labels correspond to the spacer sequence (first digit) followed by an identifier for a gRNA targeting that spacer sequence (second digit). Numbers to the right of gRNA sequences in purple correspond to the length of complementarity to the target sequence. Blue G's correspond to the G complementary to the C in H67 that can undergo mutagenesis to T to generate GFP. Red lowercase g's represent mismatched terminal guanines, which are added to all gRNAs that do not naturally end in a purine (G or A). **c,** Slug-nCas9-PolI5MΔ enables guide-dependent mutagenesis of BFP most consistently when gRNAs share 20 or 21 bp of complementarity with the target strand. The percentage of GFP positive cells generated by each construct was measured by flow cytometry and normalized by transfection efficiency. Dots represent individual biological replicates. gRNA numbers correspond to the spacers labelled in **b**. bp labels under groups of gRNAs indicate the number of nt within a given guide that are complementary to their target strand.

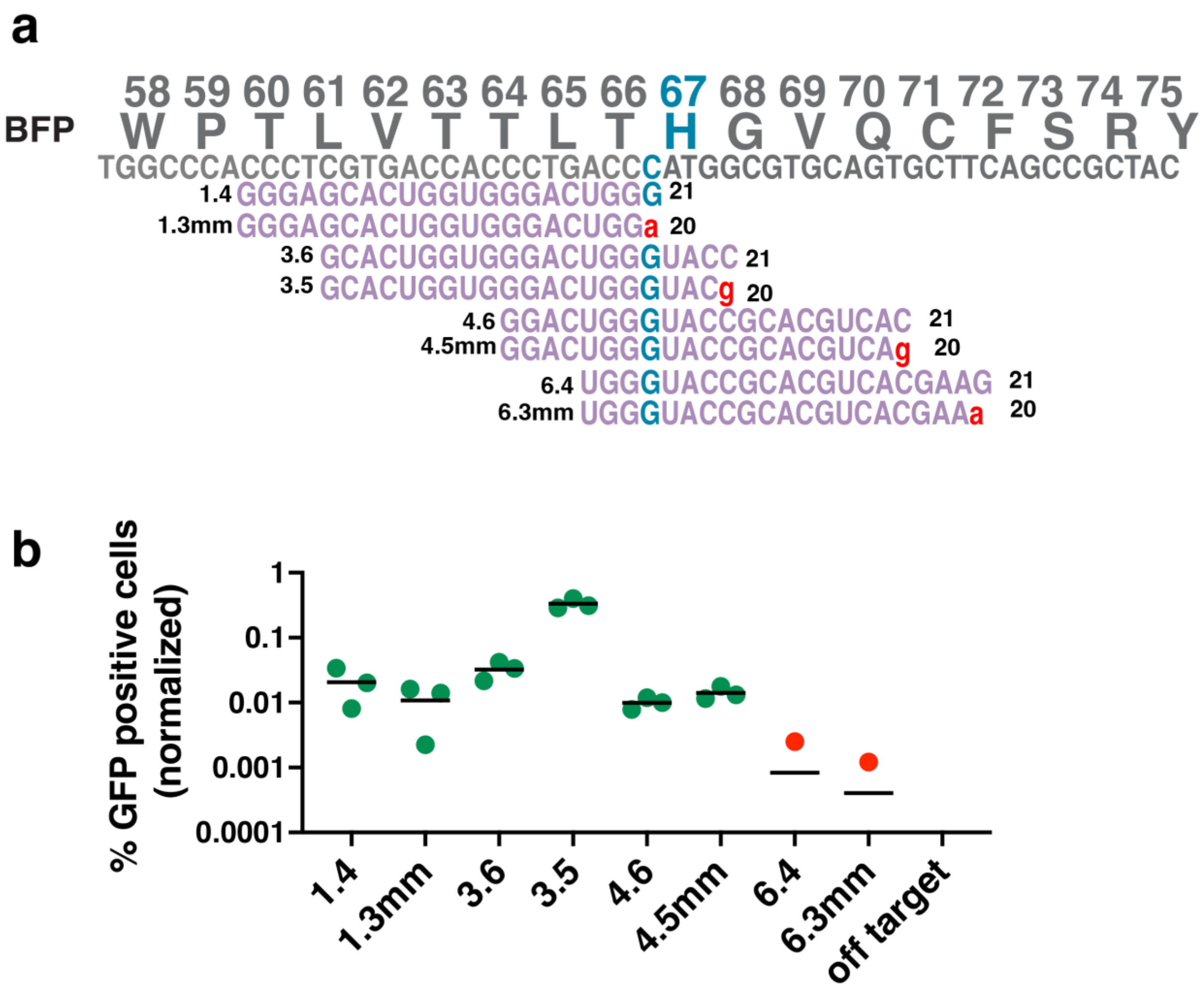

Supplementary Figure 9. **5' terminal mismatches affect EvolvR's substitution rates. a,** Diagram depicts a panel of eight gRNAs targeting four distinct spacer sequences in the BFP gene. Decimal labels correspond to the spacer sequence (first digit) followed by an identifier for a gRNA targeting that spacer sequence (second digit). Numbers to the right of gRNA sequences in purple correspond to the length of complementarity to the target sequence. Blue G's correspond to the G complementary to the C in H67 that can undergo mutagenesis to T to generate GFP. Red lowercase letters represent mismatched terminal purines, which are added to test EvolvR's mutagenesis using 21 nt gRNAs with and without 5' terminal mismatches. **b,** The effect of 5' terminal mismatched purines in 21 nt gRNAs results in guide-dependent improvements on EvolvR mutagenesis. The percentage of GFP positive cells generated by each construct was measured by flow cytometry and normalized by transfection efficiency. Dots represent individual biological replicates. Green, yellow, red, or no dots represent that three, two, one, or zero out of three biological replicates contained GFP positive cells, respectively. Black lines represent mean % GFP positive cells. gRNA labels correspond to labels in **a**.

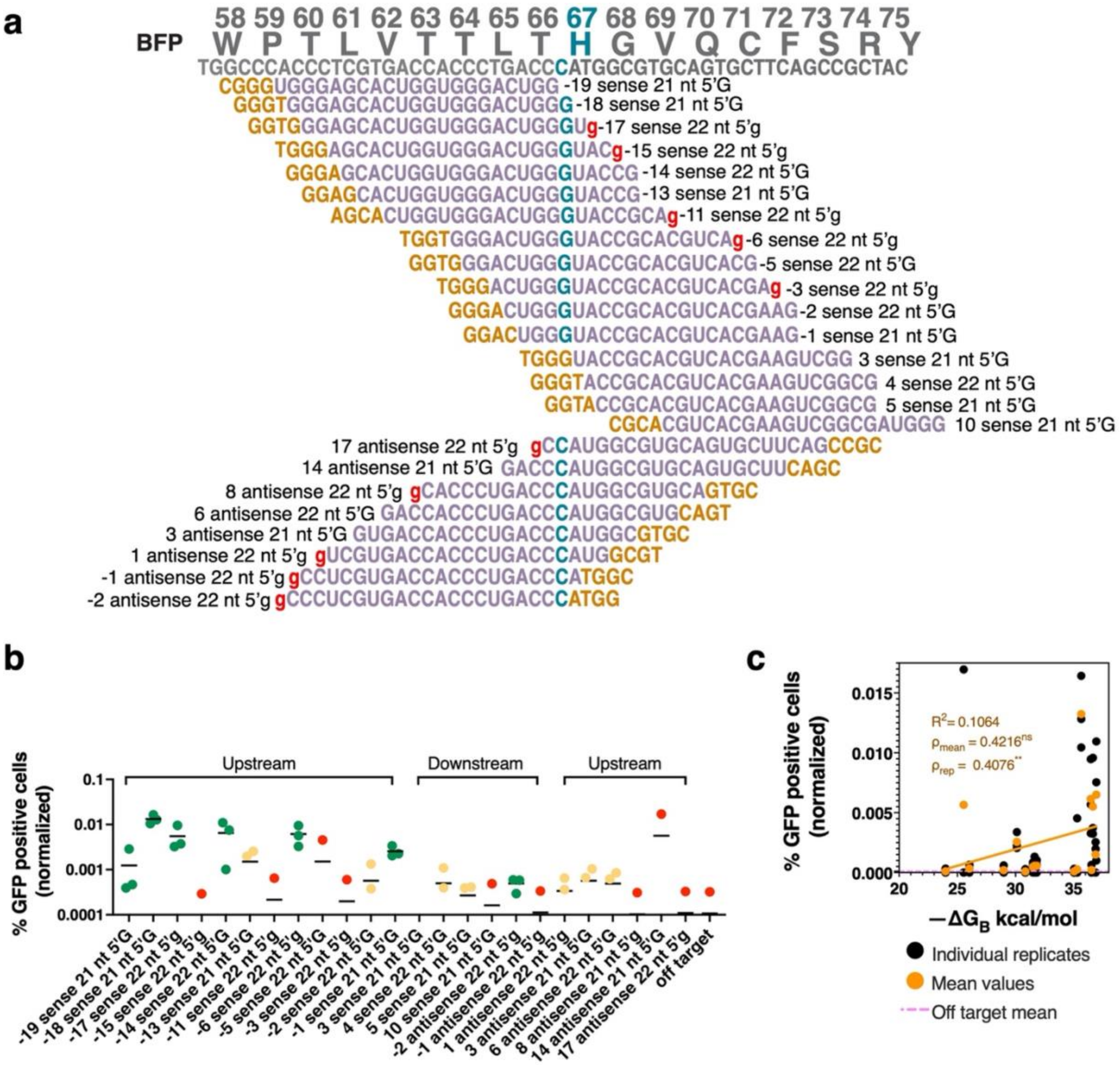

Supplementary Figure 10. **NNG-Slug-nCas9 enables highly redundant targeting of genomic nucleotides for mutagenesis. a,** Schematic depicts 24 gRNAs targeting either strand of the BFP transgene. gRNA and PAM sequences are purple and gold, respectively. gRNA labels indicate the position of gRNA-specific cut sites relative to C199 and gRNA length. Uppercase and lowercase G/g's indicate terminal guanines are matched or mismatched to the target sequence, respectively. **b,** NNG-Slug-nCas9-PolI5MΔ enabled mutagenesis of C199 with most gRNAs resulting in nicks 5' of C199 on the target strand. Dots represent the percentage of GFP positive cells detected by flow cytometry normalized by the transfection efficiency of each condition. Green, yellow, red, or no dots indicate that three, two, one, or zero out of three biological replicates contained GFP positive cells, respectively. Black lines represent mean % GFP positive cells. gRNA labels correspond to distance in bp from C199. Negative values correspond to positions 5' of C199 on the sense strand; positive values correspond to positions 3' of C199 on the sense strand. "Sense" and "antisense" correspond to the gRNA target strand. "21 nt" and "22 nt" correspond to the total length of each gRNA. Lowercase and uppercase G's correspond to mismatched and complementary 5' terminal G's in each gRNA, respectively. **c,** The frequency of

GFP positive cells generated by each gRNA is weakly correlated with the total free energy change of R loop formation. The mean normalized frequency of GFP positive cells generated by each of 17 gRNAs overlapping with and generating nicks upstream of C199 is plotted alongside the normalized frequency of GFP positive cells generated by each biological replicate. ρ_{rep} and ρ_{mean} represent Spearman rank correlation coefficients calculated for individual and mean values of percent GFP positive cells, respectively. Two asterisks (**) indicate a p value less than 0.003 and ns (not significant) indicates a p value of 0.0934. The orange line represents a linear regression fit to mean GFP positive cell frequencies.

Supplementary Table 1: **Plasmids used in this study.**

p.mCherry	CGCGGAACCCCTATTTGTTTATTTTTCTAAATACATTCAAATATGTA<br>TCCGCTCATGAGACAATAACCCTGATAAATGCTTCAATAATATTGA<br>AAAAGGAAGAGTATGAGTATTCAACATTTCCGTGTCGCCCTTATTC<br>CCTTTTTTGCGGCATTTTGCCTTCCTGTTTTTGCTCACCCAGAAACG<br>CTGGTGAAAGTAAAAGATGCTGAAGATCAGTTGGGTGCACGAGTG<br>GGTTACATCGAACTGGATCTCAACAGCGGTAAGATCCTTGAGAGT<br>TTTCGCCCCGAAGAACGTTTTCCAATGATGAGCACTTTTAAAGTTC<br>TGCTATGTGGCGCGGTATTATCCCGTATTGACGCCGGGCAAGAGC<br>AACTCGGTCGCCGCATACACTATTCTCAGAATGACTTGGTTGAGTA<br>CTCACCAGTCACAGAAAAGCATCTTACGGATGGCATGACAGTAAG<br>AGAATTATGCAGTGCTGCCATAACCATGAGTGATAACACTGCGGC<br>CAACTTACTTCTGACAACGATCGGAGGACCGAAGGAGCTAACCGC<br>TTTTTTGCACAACATGGGGGATCATGTAACTCGCCTTGATCGTTGG<br>GAACCGGAGCTGAATGAAGCCATACCAAACGACGAGCGTGACACC<br>ACGATGCCTGTAGCAATGGCAACAACGTTGCGCAAACTATTAACT<br>GGCGAACTACTTACTCTAGCTTCCCGGCAACAATTAATAGACTGGA<br>TGGAGGCGGATAAAGTTGCAGGACCACTTCTGCGCTCGGCCCTTCC<br>GGCTGGCTGGTTTATTGCTGATAAATCTGGAGCCGGTGAGCGTGGT<br>TCTCGCGGTATCATTGCAGCACTGGGGCCAGATGGTAAGCCCTCCC<br>GTATCGTAGTTATCTACACGACGGGGAGTCAGGCAACTATGGATG<br>AACGAAATAGACAGATCGCTGAGATAGGTGCCTCACTGATTAAGC<br>ATTGGTAACTGTCAGACCAAGTTTACTCATATATACTTTAGATTGA<br>TTTAAAACTTCATTTTTAATTTAAAAGGATCTAGGTGAAGATCCTT<br>TTTGATAATCTCATGACCAAAATCCCTTAACGTGAGTTTTCGTTCC<br>ACTGAGCGTCAGACCCCGTAGAAAAGATCAAAGGATCTTCTTGAG<br>ATCCTTTTTTTCTGCGCGTAATCTGCTGCTTGCAAACAAAAAAACC<br>ACCGCTACCAGCGGTGGTTTGTTTGCCGGATCAAGAGCTACCAACT<br>CTTTTTCCGAAGGTAACTGGCTTCAGCAGAGCGCAGATACCAAAT<br>ACTGTCCTTCTAGTGTAGCCGTAGTTAGGCCACCACTTCAAGAACT<br>CTGTAGCACCGCCTACATACCTCGCTCTGCTAATCCTGTTACCAGT<br>GGCTGCTGCCAGTGGCGATAAGTCGTGTCTTACCGGGTTGGACTCA<br>AGACGATAGTTACCGGATAAGGCGCAGCGGTCGGGCTGAACGGGG<br>GGTTCGTGCACACAGCCCAGCTTGGAGCGAACGACCTACACCGAA<br>CTGAGATACCTACAGCGTGAGCTATGAGAAAGCGCCACGCTTCCC<br>GAAGGGAGAAAGGCGGACAGGTATCCGGTAAGCGGCAGGGTCGG<br>AACAGGAGAGCGCACGAGGGAGCTTCCAGGGGGAAACGCCTGGT<br>ATCTTTATAGTCCTGTCGGGTTTCGCCACCTCTGACTTGAGCGTCG<br>ATTTTTGTGATGCTCGTCAGGGGGGCGGAGCCTATGGAAAAACGC<br>CAGCAACGCGGCCTTTTTACGGTTCCTGGCCTTTTGCTGGCCTTTTG<br>CTCACATGTTCTTTCCTGCGTTATCCCCTGATTCTGTGGATAACCGT<br>ATTACCGCCTTTGAGTGAGCTGATACCGCTCGCCGCAGCCGAACG<br>ACCGAGCGCAGCGAGTCAGTGAGCGAGGAAGCGGAAGAGCGCCC<br>AATACGCAAACCGCCTCTCCCCGCGCGTTGGCCGATTCATTAATGC<br>AGCTGGCACGACAGGTTTCCCGACTGGAAAGCGGGCAGTGAGCGC

	AACGCAATTAATGTGAGTTAGCTCACTCATTAGGCACCCCAGGCTT<br>TACACTTTATGCTTCCGGCTCGTATGTTGTGTGGAATTGTGAGCGG<br>ATAACAATTTCACACAGGAAACAGCTATGACCATGATTACGCCAA<br>GCGCGCAATTAACCCTCACTAAAGGGAACAAAAGCTGGAGCTCCA<br>CCGCGGTGGCGGCCGCTCTTAAGGGGTGCAGCGGCCTCCGCGCCG<br>GGTTTTGGCGCCTCGATCCAAGGTCGGGCAGGAAGAGGGCCTATT<br>TCCCATGATTCCTTCATATTTGCATATACGATACAAGGCTGTTAGA<br>GAGATAATTAGAATTAATTTGACTGTAAACACAAAGATATTAGTA<br>CAAAATACGTGACGTAGAAAGTAATAATTTCTTGGGTAGTTTGCA<br>GTTTTAAAATTATGTTTTAAAATGGACTATCATATGCTTACCGTAA<br>CTTGAAAGTATTTCGATTTCTTGGCTTTATATATCTTGTGGAAAGG<br>ACGAAACACC-[gRNA SEQUENCE]-<br>GTTTTAGAGCTAGAAATAGCAAGTTAAAATAAGGCTAGTCCGTTA<br>TCAACTTGAAAAAGTGGCACCGAGTCGGTGCTTTTTTCCGCGGCCT<br>CTAGACTCGAGGCGTTGACATTGATTATTGACTAGTTATTAATAGT<br>AATCAATTACGGGGTCATTAGTTCATAGCCCATATATGGAGTTCCG<br>CGTTACATAACTTACGGTAAATGGCCCGCCTGGCTGACCGCCCAAC<br>GACCCCCGCCCATTGACGTCAATAATGACGTATGTTCCCATAGTAA<br>CGCCAATAGGGACTTTCCATTGACGTCAATGGGTGGAGTATTTACG<br>GTAAACTGCCCACTTGGCAGTACATCAAGTGTATCATATGCCAAGT<br>ACGCCCCCTATTGACGTCAATGACGGTAAATGGCCCGCCTGGCATT<br>ATGCCCAGTACATGACCTTATGGGACTTTCCTACTTGGCAGTACAT<br>CTACGTATTAGTCATCGCTATTACCATGGTGATGCGGTTTTGGCAG<br>TACATCAATGGGCGTGGATAGCGGTTTGACTCACGGGGATTTCCA<br>AGTCTCCACCCCATTGACGTCAATGGGAGTTTGTTTTGGCACCAAA<br>ATCAACGGGACTTTCCAAAATGTCGTAACAACTCCGCCCCATTGAC<br>GCAAATGGGCGGTAGGCGTGTACGGTGGGAGGTCTATATAAGCAG<br>AGCTCTCTGGCTAACTACCGGTGCCACCATGGCCCCAAAGAAGAA<br>GCGGAAGGTCGGTATCCACGGAGTCCCAGCAGCCGTGAGCAAGGG<br>CGAGGAGGATAACATGGCCATCATCAAGGAGTTCATGCGCTTCAA<br>GGTGCACATGGAGGGCTCCGTGAACGGCCACGAGTTCGAGATCGA<br>GGGCGAGGGCGAGGGCCGCCCCTACGAGGGCACCCAGACCGCCA<br>AGCTGAAGGTGACCAAGGGTGGCCCCCTGCCCTTCGCCTGGGACA<br>TCCTGTCCCCTCAGTTCATGTACGGCTCCAAGGCCTACGTGAAGCA<br>CCCCGCCGACATCCCCGACTACTTGAAGCTGTCCTTCCCCGAGGGC<br>TTCAAGTGGGAGCGCGTGATGAACTTCGAGGACGGCGGCGTGGTG<br>ACCGTGACCCAGGACTCCTCCCTGCAGGACGGCGAGTTCATCTAC<br>AAGGTGAAGCTGCGCGGCACCAACTTCCCCTCCGACGGCCCCGTA<br>ATGCAGAAGAAGACCATGGGCTGGGAGGCCTCCTCCGAGCGGATG<br>TACCCCGAGGACGGCGCCCTGAAGGGCGAGATCAAGCAGAGGCTG<br>AAGCTGAAGGACGGCGGCCACTACGACGCTGAGGTCAAGACCACC<br>TACAAGGCCAAGAAGCCCGTGCAGCTGCCCGGCGCCTACAACGTC<br>AACATCAAGTTGGACATCACCTCCCACAACGAGGACTACACCATC<br>GTGGAACAGTACGAACGCGCCGAGGGCCGCCACTCCACCGGCGGC<br>ATGGACGAGCTGTACAAGGGATCCGGTATGTAATGGCTGAAAACT<br>GGGACCAGGCTCACTAACTCAGATCCTACTAGGTTTAATAAACATC

	TTTATTTTCATTACATCTGTGTGTTGGTTTTTTGTGTGGTACCCAAT<br>TCGCCCTATAGTGAGTCGTATTACTCACTGGCCGTCGTTTTACAAC<br>GTCGTGACTGGGAAAACCCTGGCGTTACCCAACTTAATCGCCTTGC<br>AGCACATCCCCCTTTCGCCAGCTGGCGTAATAGCGAAGAGGCCCG<br>CACCGATCGCCCTTCCCAACAGTTGCGCAGCCTGAATGGCGAATG<br>GGACGCGCCCTGTAGCGGCGCATTAAGCGCGGCGGGTGTGGTGGT<br>TACGCGCAGCGTGACCGCTACACTTGCCAGCGCCCTAGCGCCCGCT<br>CCTTTCGCTTTCTTCCCTTCCTTTCTCGCCACGTTCGCCGGCTTTCCC<br>CGTCAAGCTCTAAATCGGGGGCTCCCTTTAGGGTTCCGATTTAGTG<br>CTTTACGGCACCTCGACCCCAAAAAACTTGATTAGGGTGATGGTTC<br>ACGTAGTGGGCCATCGCCCTGATAGACGGTTTTTCGCCCTTTGACG<br>TTGGAGTCCACGTTCTTTAATAGTGGACTCTTGTTCCAAACTGGAA<br>CAACACTCAACCCTATCTCGGTCTATTCTTTTGATTTATAAGGGATT<br>TTGCCGATTTCGGCCTATTGGTTAAAAAATGAGCTGATTTAACAAA<br>AATTTAACGCGAATTTTAACAAAATATTAACGCTTACAATTTAGGT<br>GGCACTTTTCGGGGAAATGTG<br><br>**Ampicillin resistance cassette-ColE1 origin of replication-hU6 promoter-gRNA sequence-SpCas9 gRNA scaffold-CMV promoter-NLS-mCherry-GSGM-stop-SV40 polyA**
p.enCas9	CGCGGAACCCCTATTTGTTTATTTTTCTAAATACATTCAAATATGTA<br>TCCGCTCATGAGACAATAACCCTGATAAATGCTTCAATAATATTGA<br>AAAAGGAAGAGTATGAGTATTCAACATTTCCGTGTCGCCCTTATTC<br>CCTTTTTTGCGGCATTTTGCCTTCCTGTTTTTGCTCACCCAGAAACG<br>CTGGTGAAAGTAAAAGATGCTGAAGATCAGTTGGGTGCACGAGTG<br>GGTTACATCGAACTGGATCTCAACAGCGGTAAGATCCTTGAGAGT<br>TTTCGCCCCGAAGAACGTTTTCCAATGATGAGCACTTTTAAAGTTC<br>TGCTATGTGGCGCGGTATTATCCCGTATTGACGCCGGGCAAGAGC<br>AACTCGGTCGCCGCATACACTATTCTCAGAATGACTTGGTTGAGTA<br>CTCACCAGTCACAGAAAAGCATCTTACGGATGGCATGACAGTAAG<br>AGAATTATGCAGTGCTGCCATAACCATGAGTGATAACACTGCGGC<br>CAACTTACTTCTGACAACGATCGGAGGACCGAAGGAGCTAACCGC<br>TTTTTTGCACAACATGGGGGATCATGTAACTCGCCTTGATCGTTGG<br>GAACCGGAGCTGAATGAAGCCATACCAAACGACGAGCGTGACACC<br>ACGATGCCTGTAGCAATGGCAACAACGTTGCGCAAACTATTAACT<br>GGCGAACTACTTACTCTAGCTTCCCGGCAACAATTAATAGACTGGA<br>TGGAGGCGGATAAAGTTGCAGGACCACTTCTGCGCTCGGCCCTTCC<br>GGCTGGCTGGTTTATTGCTGATAAATCTGGAGCCGGTGAGCGTGGT<br>TCTCGCGGTATCATTGCAGCACTGGGGCCAGATGGTAAGCCCTCCC<br>GTATCGTAGTTATCTACACGACGGGGAGTCAGGCAACTATGGATG<br>AACGAAATAGACAGATCGCTGAGATAGGTGCCTCACTGATTAAGC<br>ATTGGTAACTGTCAGACCAAGTTTACTCATATATACTTTAGATTGA<br>TTTAAAACTTCATTTTTAATTTAAAAGGATCTAGGTGAAGATCCTT<br>TTTGATAATCTCATGACCAAAATCCCTTAACGTGAGTTTTCGTTCC

	ACTGAGCGTCAGACCCCGTAGAAAAGATCAAAGGATCTTCTTGAG<br>ATCCTTTTTTTCTGCGCGTAATCTGCTGCTTGCAAACAAAAAAACC<br>ACCGCTACCAGCGGTGGTTTGTTTGCCGGATCAAGAGCTACCAACT<br>CTTTTTCCGAAGGTAACTGGCTTCAGCAGAGCGCAGATACCAAAT<br>ACTGTCCTTCTAGTGTAGCCGTAGTTAGGCCACCACTTCAAGAACT<br>CTGTAGCACCGCCTACATACCTCGCTCTGCTAATCCTGTTACCAGT<br>GGCTGCTGCCAGTGGCGATAAGTCGTGTCTTACCGGGTTGGACTCA<br>AGACGATAGTTACCGGATAAGGCGCAGCGGTCGGGCTGAACGGGG<br>GGTTCGTGCACACAGCCCAGCTTGGAGCGAACGACCTACACCGAA<br>CTGAGATACCTACAGCGTGAGCTATGAGAAAGCGCCACGCTTCCC<br>GAAGGGAGAAAGGCGGACAGGTATCCGGTAAGCGGCAGGGTCGG<br>AACAGGAGAGCGCACGAGGGAGCTTCCAGGGGGAAACGCCTGGT<br>ATCTTTATAGTCCTGTCGGGTTTCGCCACCTCTGACTTGAGCGTCG<br>ATTTTTGTGATGCTCGTCAGGGGGGCGGAGCCTATGGAAAAACGC<br>CAGCAACGCGGCCTTTTTACGGTTCCTGGCCTTTTGCTGGCCTTTTG<br>CTCACATGTTCTTTCCTGCGTTATCCCCTGATTCTGTGGATAACCGT<br>ATTACCGCCTTTGAGTGAGCTGATACCGCTCGCCGCAGCCGAACG<br>ACCGAGCGCAGCGAGTCAGTGAGCGAGGAAGCGGAAGAGCGCCC<br>AATACGCAAACCGCCTCTCCCCGCGCGTTGGCCGATTCATTAATGC<br>AGCTGGCACGACAGGTTTCCCGACTGGAAAGCGGGCAGTGAGCGC<br>AACGCAATTAATGTGAGTTAGCTCACTCATTAGGCACCCCAGGCTT<br>TACACTTTATGCTTCCGGCTCGTATGTTGTGTGGAATTGTGAGCGG<br>ATAACAATTTCACACAGGAAACAGCTATGACCATGATTACGCCAA<br>GCGCGCAATTAACCCTCACTAAAGGGAACAAAAGCTGGAGCTCCA<br>CCGCGGTGGCGGCCGCTCTTAAGGGGTGCAGCGGCCTCCGCGCCG<br>GGTTTTGGCGCCTCGATCCAAGGTCGGGCAGGAAGAGGGCCTATT<br>TCCCATGATTCCTTCATATTTGCATATACGATACAAGGCTGTTAGA<br>GAGATAATTAGAATTAATTTGACTGTAAACACAAAGATATTAGTA<br>CAAAATACGTGACGTAGAAAGTAATAATTTCTTGGGTAGTTTGCA<br>GTTTTAAAATTATGTTTTAAAATGGACTATCATATGCTTACCGTAA<br>CTTGAAAGTATTTCGATTTCTTGGCTTTATATATCTTGTGGAAAGG<br>ACGAAACACC-[gRNA SEQUENCE]-<br>GTTTTAGAGCTAGAAATAGCAAGTTAAAATAAGGCTAGTCCGTTA<br>TCAACTTGAAAAAGTGGCACCGAGTCGGTGCTTTTTTCCGCGGCCT<br>CTAGACTCGAGGCGTTGACATTGATTATTGACTAGTTATTAATAGT<br>AATCAATTACGGGGTCATTAGTTCATAGCCCATATATGGAGTTCCG<br>CGTTACATAACTTACGGTAAATGGCCCGCCTGGCTGACCGCCCAAC<br>GACCCCCGCCCATTGACGTCAATAATGACGTATGTTCCCATAGTAA<br>CGCCAATAGGGACTTTCCATTGACGTCAATGGGTGGAGTATTTACG<br>GTAAACTGCCCACTTGGCAGTACATCAAGTGTATCATATGCCAAGT<br>ACGCCCCCTATTGACGTCAATGACGGTAAATGGCCCGCCTGGCATT<br>ATGCCCAGTACATGACCTTATGGGACTTTCCTACTTGGCAGTACAT<br>CTACGTATTAGTCATCGCTATTACCATGGTGATGCGGTTTTGGCAG<br>TACATCAATGGGCGTGGATAGCGGTTTGACTCACGGGGATTTCCA<br>AGTCTCCACCCCATTGACGTCAATGGGAGTTTGTTTTGGCACCAAA<br>ATCAACGGGACTTTCCAAAATGTCGTAACAACTCCGCCCCATTGAC

	GCAAATGGGCGGTAGGCGTGTACGGTGGGAGGTCTATATAAGCAG<br>AGCTCTCTGGCTAACTACCGGTGCCACCATGGCCCCAAAGAAGAA<br>GCGGAAGGTCGGTATCCACGGAGTCCCAGCAGCCGTGAGCAAGGG<br>CGAGGAGGATAACATGGCCATCATCAAGGAGTTCATGCGCTTCAA<br>GGTGCACATGGAGGGCTCCGTGAACGGCCACGAGTTCGAGATCGA<br>GGGCGAGGGCGAGGGCCGCCCCTACGAGGGCACCCAGACCGCCA<br>AGCTGAAGGTGACCAAGGGTGGCCCCCTGCCCTTCGCCTGGGACA<br>TCCTGTCCCCTCAGTTCATGTACGGCTCCAAGGCCTACGTGAAGCA<br>CCCCGCCGACATCCCCGACTACTTGAAGCTGTCCTTCCCCGAGGGC<br>TTCAAGTGGGAGCGCGTGATGAACTTCGAGGACGGCGGCGTGGTG<br>ACCGTGACCCAGGACTCCTCCCTGCAGGACGGCGAGTTCATCTAC<br>AAGGTGAAGCTGCGCGGCACCAACTTCCCCTCCGACGGCCCCGTA<br>ATGCAGAAGAAGACCATGGGCTGGGAGGCCTCCTCCGAGCGGATG<br>TACCCCGAGGACGGCGCCCTGAAGGGCGAGATCAAGCAGAGGCTG<br>AAGCTGAAGGACGGCGGCCACTACGACGCTGAGGTCAAGACCACC<br>TACAAGGCCAAGAAGCCCGTGCAGCTGCCCGGCGCCTACAACGTC<br>AACATCAAGTTGGACATCACCTCCCACAACGAGGACTACACCATC<br>GTGGAACAGTACGAACGCGCCGAGGGCCGCCACTCCACCGGCGGC<br>ATGGACGAGCTGTACAAGGGATCCGGTATGGACAAGAAGTACAGC<br>ATCGGCCTGGCCATCGGCACCAACTCTGTGGGCTGGGCCGTGATC<br>ACCGACGAGTACAAGGTGCCCAGCAAGAAATTCAAGGTGCTGGGC<br>AACACCGACCGGCACAGCATCAAGAAGAACCTGATCGGCGCCCTG<br>CTGTTCGACAGCGGAGAAACAGCCGAGGCCACCCGGCTGAAGAGA<br>ACCGCCAGAAGAAGATACACCAGACGGAAGAACCGGATCTGCTAT<br>CTGCAAGAGATCTTCAGCAACGAGATGGCCAAGGTGGACGACAGC<br>TTCTTCCACAGACTGGAAGAGTCCTTCCTGGTGGAAGAGGATAAG<br>AAGCACGAGCGGCACCCCATCTTCGGCAACATCGTGGACGAGGTG<br>GCCTACCACGAGAAGTACCCCACCATCTACCACCTGAGAAAGAAA<br>CTGGTGGACAGCACCGACAAGGCCGACCTGCGGCTGATCTATCTG<br>GCCCTGGCCCACATGATCAAGTTCCGGGGCCACTTCCTGATCGAGG<br>GCGACCTGAACCCCGACAACAGCGACGTGGACAAGCTGTTCATCC<br>AGCTGGTGCAGACCTACAACCAGCTGTTCGAGGAAAACCCCATCA<br>ACGCCAGCGGCGTGGACGCCAAGGCCATCCTGTCTGCCAGACTGA<br>GCAAGAGCAGACGGCTGGAAAATCTGATCGCCCAGCTGCCCGGCG<br>AGAAGAAGAATGGCCTGTTCGGCAACCTGATTGCCCTGAGCCTGG<br>GCCTGACCCCCAACTTCAAGAGCAACTTCGACCTGGCCGAGGATG<br>CCAAACTGCAGCTGAGCAAGGACACCTACGACGACGACCTGGACA<br>ACCTGCTGGCCCAGATCGGCGACCAGTACGCCGACCTGTTTCTGGC<br>CGCCAAGAACCTGTCCGACGCCATCCTGCTGAGCGACATCCTGAG<br>AGTGAACACCGAGATCACCAAGGCCCCCCTGAGCGCCTCTATGAT<br>CAAGAGATACGACGAGCACCACCAGGACCTGACCCTGCTGAAAGC<br>TCTCGTGCGGCAGCAGCTGCCTGAGAAGTACAAAGAGATTTTCTTC<br>GACCAGAGCAAGAACGGCTACGCCGGCTACATCGATGGCGGAGCC<br>AGCCAGGAAGAGTTCTACAAGTTCATCAAGCCCATCCTGGAAAAG<br>ATGGACGGCACCGAGGAACTGCTCGTGAAGCTGAACAGAGAGGA<br>CCTGCTGCGGAAGCAGCGGACCTTCGACAACGGCAGCATCCCCCA

	CCAGATCCACCTGGGAGAGCTGCACGCCATTCTGCGGCGGCAGGA<br>AGATTTTTACCCATTCCTGAAGGACAACCGGGAAAAGATCGAGAA<br>GATCCTGACCTTCCGCATCCCCTACTACGTGGGCCCTCTGGCCAGG<br>GGAAACAGCAGATTCGCCTGGATGACCAGAAAGAGCGAGGAAAC<br>CATCACCCCCTGGAACTTCGAGGAAGTGGTGGACAAGGGCGCCAG<br>CGCCCAGAGCTTCATCGAGCGGATGACCAACTTCGATAAGAACCT<br>GCCCAACGAGAAGGTGCTGCCCAAGCACAGCCTGCTGTACGAGTA<br>CTTCACCGTGTACAACGAGCTGACCAAAGTGAAATACGTGACCGA<br>GGGAATGAGAAAGCCCGCCTTCCTGAGCGGCGAGCAGAAAAAAG<br>CCATCGTGGACCTGCTGTTCAAGACCAACCGGAAAGTGACCGTGA<br>AGCAGCTGAAAGAGGACTACTTCAAGAAAATCGAGTGCTTCGACT<br>CCGTGGAAATCTCCGGCGTGGAAGATCGGTTCAACGCCTCCCTGG<br>GCACATACCACGATCTGCTGAAAATTATCAAGGACAAGGACTTCC<br>TGGACAATGAGGAAAACGAGGACATTCTGGAAGATATCGTGCTGA<br>CCCTGACACTGTTTGAGGACAGAGAGATGATCGAGGAACGGCTGA<br>AAACCTATGCCCACCTGTTCGACGACAAAGTGATGAAGCAGCTGA<br>AGCGGCGGAGATACACCGGCTGGGGCAGGCTGAGCCGGAAGCTG<br>ATCAACGGCATCCGGGACAAGCAGTCCGGCAAGACAATCCTGGAT<br>TTCCTGAAGTCCGACGGCTTCGCCAACAGAAACTTCATGCAGCTGA<br>TCCACGACGACAGCCTGACCTTTAAAGAGGACATCCAGAAAGCCC<br>AGGTGTCCGGCCAGGGCGATAGCCTGCACGAGCACATTGCCAATC<br>TGGCCGGCAGCCCCGCCATTAAGAAGGGCATCCTGCAGACAGTGA<br>AGGTGGTGGACGAGCTCGTGAAAGTGATGGGCCGGCACAAGCCCG<br>AGAACATCGTGATCGAAATGGCCAGAGAGAACCAGACCACCCAG<br>AAGGGACAGAAGAACAGCCGCGAGAGAATGAAGCGGATCGAAGA<br>GGGCATCAAAGAGCTGGGCAGCCAGATCCTGAAAGAACACCCCGT<br>GGAAAACACCCAGCTGCAGAACGAGAAGCTGTACCTGTACTACCT<br>GCAGAATGGGCGGGATATGTACGTGGACCAGGAACTGGACATCAA<br>CCGGCTGTCCGACTACGATGTGGACCATATCGTGCCTCAGAGCTTT<br>CTGGCAGACGACTCCATCGATAACAAAGTGCTGACTCGGAGCGAC<br>AAGAACCGGGGCAAGAGCGACAACGTGCCCTCCGAAGAGGTCGT<br>GAAGAAGATGAAGAACTACTGGCGCCAGCTGCTGAATGCCAAGCT<br>GATTACCCAGAGGAAGTTCGACAATCTGACCAAGGCCGAGAGAGG<br>CGGCCTGAGCGAACTGGATAAGGCCGGCTTCATCAAGAGACAGCT<br>GGTGGAAACCCGGCAGATCACAAAGCACGTGGCACAGATCCTGGA<br>CTCCCGGATGAACACTAAGTACGACGAGAACGACAAACTGATCCG<br>GGAAGTGAAAGTGATCACCCTGAAGTCCAAGCTGGTGTCCGATTT<br>CCGGAAGGATTTCCAGTTTTACAAAGTGCGCGAGATCAACAACTA<br>CCACCACGCCCACGACGCCTACCTGAACGCCGTCGTGGGAACCGC<br>CCTGATCAAAAAGTACCCTGCGCTGGAAAGCGAGTTCGTGTACGG<br>CGACTACAAGGTGTACGACGTGCGGAAGATGATCGCCAAGAGCGA<br>GCAGGAAATCGGCAAGGCTACCGCCAAGTACTTCTTCTACAGCAA<br>CATCATGAACTTTTTCAAGACCGAGATTACCCTGGCCAACGGCGA<br>GATCCGGAAGGCGCCTCTGATCGAGACAAACGGCGAAACAGGCG<br>AGATCGTGTGGGATAAGGGCCGGGACTTTGCCACCGTGCGGAAAG<br>TGCTGTCTATGCCCCAAGTGAATATCGTGAAAAAGACCGAGGTGC

	AGACAGGCGGCTTCAGCAAAGAGTCTATCCTGCCCAAGAGGAACA GCGACAAGCTGATCGCCAGAAAGAAGGACTGGGACCCTAAGAAG TACGGCGGCTTCGACAGCCCCACCGTGGCCTATTCTGTGCTGGTGG TGGCCAAAGTGGAAAAGGGCAAGTCCAAGAAACTGAAGAGTGTG AAAGAGCTGCTGGGGATCACCATCATGGAAAGAAGCAGCTTCGAG AAGAATCCCATCGACTTTCTGGAAGCCAAGGGCTACAAAGAAGTG AAAAAGGACCTGATCATCAAGCTGCCTAAGTACTCCCTGTTCGAG CTGGAAAACGGCCGGAAGAGAATGCTGGCCTCTGCCGGCGAACTG CAGAAGGGAAACGAACTGGCCCTGCCCTCCAAATATGTGAACTTC CTGTACCTGGCCAGCCACTATGAGAAGCTGAAGGGCTCCCCCGAG GATAATGAGCAGAAACAGCTGTTTGTGGAACAGCACAAACACTAC CTGGACGAGATCATCGAGCAGATCAGCGAGTTCTCCAAGAGAGTG ATCCTGGCCGACGCTAATCTGGACAAGGTGCTGAGCGCCTACAAC AAGCACAGAGACAAGCCTATCAGAGAGCAGGCCGAGAATATCATC CACCTGTTTACCCTGACCAATCTGGGAGCCCCTGCCGCCTTCAAGT ACTTTGACACCACCATCGACCGGAAGAGGTACACCAGCACCAAAG AGGTGCTGGACGCCACCCTGATCCACCAGAGCATCACCGGCCTGT ACGAGACACGGATCGACCTGTCTCAGCTGGGAGGCGACGCCTATC CCTATGACGTGCCCGATTATGCCAGCCTGGGCAGCGGCTCCCCCAA GAAAAAACGCAAGGTGGAAGATCCTAAGAAAAAGCGGAAAGGTT CTTAATGGCTGAAAACTGGGACCAGGCTCACTAACTCAGATCCTA CTAGGTTTAATAAACATCTTTATTTTCATTACATCTGTGTGTTGGTT TTTTGTGTGGTACCCAATTCGCCCTATAGTGAGTCGTATTACTCACT GGCCGTCGTTTTACAACGTCGTGACTGGGAAAACCCTGGCGTTACC CAACTTAATCGCCTTGCAGCACATCCCCCTTTCGCCAGCTGGCGTA ATAGCGAAGAGGCCCGCACCGATCGCCCTTCCCAACAGTTGCGCA GCCTGAATGGCGAATGGGACGCGCCCTGTAGCGGCGCATTAAGCG CGGCGGGTGTGGTGGTTACGCGCAGCGTGACCGCTACACTTGCCA GCGCCCTAGCGCCCGCTCCTTTCGCTTTCTTCCCTTCCTTTCTCGCC ACGTTCGCCGGCTTTCCCCGTCAAGCTCTAAATCGGGGGCTCCCTT TAGGGTTCCGATTTAGTGCTTTACGGCACCTCGACCCCAAAAAACT TGATTAGGGTGATGGTTCACGTAGTGGGCCATCGCCCTGATAGAC GGTTTTTCGCCCTTTGACGTTGGAGTCCACGTTCTTTAATAGTGGA CTCTTGTTCCAAACTGGAACAACACTCAACCCTATCTCGGTCTATT CTTTTGATTTATAAGGGATTTTGCCGATTTCGGCCTATTGGTTAAA AAATGAGCTGATTTAACAAAAATTTAACGCGAATTTTAACAAAAT ATTAACGCTTACAATTTAGGTGGCACTTTTCGGGGAAATGTG<br><br>**Ampicillin resistance cassette-ColE1 origin of replication-hU6 promoter-gRNA sequence-SpCas9 gRNA scaffold-CMV promoter-NLS-mCherry-GSGM-enCas9 (D10A)-NLS-stop-SV40 polyA**
p.PolI5M	CGCGGAACCCCTATTTGTTTATTTTTCTAAATACATTCAAATATGTA TCCGCTCATGAGACAATAACCCTGATAAATGCTTCAATAATATTGA AAAAGGAAGAGTATGAGTATTCAACATTTCCGTGTCGCCCTTATTC

	CCTTTTTTGCGGCATTTTGCCTTCCTGTTTTTGCTCACCCAGAAACG<br>CTGGTGAAAGTAAAAGATGCTGAAGATCAGTTGGGTGCACGAGTG<br>GGTTACATCGAACTGGATCTCAACAGCGGTAAGATCCTTGAGAGT<br>TTTCGCCCCGAAGAACGTTTTCCAATGATGAGCACTTTTAAAGTTC<br>TGCTATGTGGCGCGGTATTATCCCGTATTGACGCCGGGCAAGAGC<br>AACTCGGTCGCCGCATACACTATTCTCAGAATGACTTGGTTGAGTA<br>CTCACCAGTCACAGAAAAGCATCTTACGGATGGCATGACAGTAAG<br>AGAATTATGCAGTGCTGCCATAACCATGAGTGATAACACTGCGGC<br>CAACTTACTTCTGACAACGATCGGAGGACCGAAGGAGCTAACCGC<br>TTTTTTGCACAACATGGGGGATCATGTAACTCGCCTTGATCGTTGG<br>GAACCGGAGCTGAATGAAGCCATACCAAACGACGAGCGTGACACC<br>ACGATGCCTGTAGCAATGGCAACAACGTTGCGCAAACTATTAACT<br>GGCGAACTACTTACTCTAGCTTCCCGGCAACAATTAATAGACTGGA<br>TGGAGGCGGATAAAGTTGCAGGACCACTTCTGCGCTCGGCCCTTCC<br>GGCTGGCTGGTTTATTGCTGATAAATCTGGAGCCGGTGAGCGTGGT<br>TCTCGCGGTATCATTGCAGCACTGGGGCCAGATGGTAAGCCCTCCC<br>GTATCGTAGTTATCTACACGACGGGGAGTCAGGCAACTATGGATG<br>AACGAAATAGACAGATCGCTGAGATAGGTGCCTCACTGATTAAGC<br>ATTGGTAACTGTCAGACCAAGTTTACTCATATATACTTTAGATTGA<br>TTTAAAACTTCATTTTTAATTTAAAAGGATCTAGGTGAAGATCCTT<br>TTTGATAATCTCATGACCAAAATCCCTTAACGTGAGTTTTCGTTCC<br>ACTGAGCGTCAGACCCCGTAGAAAAGATCAAAGGATCTTCTTGAG<br>ATCCTTTTTTTCTGCGCGTAATCTGCTGCTTGCAAACAAAAAAACC<br>ACCGCTACCAGCGGTGGTTTGTTTGCCGGATCAAGAGCTACCAACT<br>CTTTTTCCGAAGGTAACTGGCTTCAGCAGAGCGCAGATACCAAAT<br>ACTGTCCTTCTAGTGTAGCCGTAGTTAGGCCACCACTTCAAGAACT<br>CTGTAGCACCGCCTACATACCTCGCTCTGCTAATCCTGTTACCAGT<br>GGCTGCTGCCAGTGGCGATAAGTCGTGTCTTACCGGGTTGGACTCA<br>AGACGATAGTTACCGGATAAGGCGCAGCGGTCGGGCTGAACGGGG<br>GGTTCGTGCACACAGCCCAGCTTGGAGCGAACGACCTACACCGAA<br>CTGAGATACCTACAGCGTGAGCTATGAGAAAGCGCCACGCTTCCC<br>GAAGGGAGAAAGGCGGACAGGTATCCGGTAAGCGGCAGGGTCGG<br>AACAGGAGAGCGCACGAGGGAGCTTCCAGGGGGAAACGCCTGGT<br>ATCTTTATAGTCCTGTCGGGTTTCGCCACCTCTGACTTGAGCGTCG<br>ATTTTTGTGATGCTCGTCAGGGGGGCGGAGCCTATGGAAAAACGC<br>CAGCAACGCGGCCTTTTTACGGTTCCTGGCCTTTTGCTGGCCTTTTG<br>CTCACATGTTCTTTCCTGCGTTATCCCCTGATTCTGTGGATAACCGT<br>ATTACCGCCTTTGAGTGAGCTGATACCGCTCGCCGCAGCCGAACG<br>ACCGAGCGCAGCGAGTCAGTGAGCGAGGAAGCGGAAGAGCGCCC<br>AATACGCAAACCGCCTCTCCCCGCGCGTTGGCCGATTCATTAATGC<br>AGCTGGCACGACAGGTTTCCCGACTGGAAAGCGGGCAGTGAGCGC<br>AACGCAATTAATGTGAGTTAGCTCACTCATTAGGCACCCCAGGCTT<br>TACACTTTATGCTTCCGGCTCGTATGTTGTGTGGAATTGTGAGCGG<br>ATAACAATTTCACACAGGAAACAGCTATGACCATGATTACGCCAA<br>GCGCGCAATTAACCCTCACTAAAGGGAACAAAAGCTGGAGCTCCA<br>CCGCGGTGGCGGCCGCTCTTAAGGGGTGCAGCGGCCTCCGCGCCG

	GGTTTTGGCGCCTCGATCCAAGGTCGGGCAGGAAGAGGGCCTATT<br>TCCCATGATTCCTTCATATTTGCATATACGATACAAGGCTGTTAGA<br>GAGATAATTAGAATTAATTTGACTGTAAACACAAAGATATTAGTA<br>CAAAATACGTGACGTAGAAAGTAATAATTTCTTGGGTAGTTTGCA<br>GTTTTAAAATTATGTTTTAAAATGGACTATCATATGCTTACCGTAA<br>CTTGAAAGTATTTCGATTTCTTGGCTTTATATATCTTGTGGAAAGG<br>ACGAAACACC-[gRNA SEQUENCE]-<br>GTTTTAGAGCTAGAAATAGCAAGTTAAAATAAGGCTAGTCCGTTA<br>TCAACTTGAAAAAGTGGCACCGAGTCGGTGCTTTTTTCCGCGGCCT<br>CTAGACTCGAGGCGTTGACATTGATTATTGACTAGTTATTAATAGT<br>AATCAATTACGGGGTCATTAGTTCATAGCCCATATATGGAGTTCCG<br>CGTTACATAACTTACGGTAAATGGCCCGCCTGGCTGACCGCCCAAC<br>GACCCCCGCCCATTGACGTCAATAATGACGTATGTTCCCATAGTAA<br>CGCCAATAGGGACTTTCCATTGACGTCAATGGGTGGAGTATTTACG<br>GTAAACTGCCCACTTGGCAGTACATCAAGTGTATCATATGCCAAGT<br>ACGCCCCCTATTGACGTCAATGACGGTAAATGGCCCGCCTGGCATT<br>ATGCCCAGTACATGACCTTATGGGACTTTCCTACTTGGCAGTACAT<br>CTACGTATTAGTCATCGCTATTACCATGGTGATGCGGTTTTGGCAG<br>TACATCAATGGGCGTGGATAGCGGTTTGACTCACGGGGATTTCCA<br>AGTCTCCACCCCATTGACGTCAATGGGAGTTTGTTTTGGCACCAAA<br>ATCAACGGGACTTTCCAAAATGTCGTAACAACTCCGCCCCATTGAC<br>GCAAATGGGCGGTAGGCGTGTACGGTGGGAGGTCTATATAAGCAG<br>AGCTCTCTGGCTAACTACCGGTGCCACCATGGCCCCAAAGAAGAA<br>GCGGAAGGTCGGTATCCACGGAGTCCCAGCAGCCGTGAGCAAGGG<br>CGAGGAGGATAACATGGCCATCATCAAGGAGTTCATGCGCTTCAA<br>GGTGCACATGGAGGGCTCCGTGAACGGCCACGAGTTCGAGATCGA<br>GGGCGAGGGCGAGGGCCGCCCCTACGAGGGCACCCAGACCGCCA<br>AGCTGAAGGTGACCAAGGGTGGCCCCCTGCCCTTCGCCTGGGACA<br>TCCTGTCCCCTCAGTTCATGTACGGCTCCAAGGCCTACGTGAAGCA<br>CCCCGCCGACATCCCCGACTACTTGAAGCTGTCCTTCCCCGAGGGC<br>TTCAAGTGGGAGCGCGTGATGAACTTCGAGGACGGCGGCGTGGTG<br>ACCGTGACCCAGGACTCCTCCCTGCAGGACGGCGAGTTCATCTAC<br>AAGGTGAAGCTGCGCGGCACCAACTTCCCCTCCGACGGCCCCGTA<br>ATGCAGAAGAAGACCATGGGCTGGGAGGCCTCCTCCGAGCGGATG<br>TACCCCGAGGACGGCGCCCTGAAGGGCGAGATCAAGCAGAGGCTG<br>AAGCTGAAGGACGGCGGCCACTACGACGCTGAGGTCAAGACCACC<br>TACAAGGCCAAGAAGCCCGTGCAGCTGCCCGGCGCCTACAACGTC<br>AACATCAAGTTGGACATCACCTCCCACAACGAGGACTACACCATC<br>GTGGAACAGTACGAACGCGCCGAGGGCCGCCACTCCACCGGCGGC<br>ATGGACGAGCTGTACAAGGGATCCGGTATGGGTTCTCCACCAAAG<br>AAGAAGAGAAAGGTTGGTTCTGGTTCTAGTGAAACCCCGGGAACA<br>AGTGAGTCGGCCACCCCTGAAGGTGGATCAGGGGGTAGCGGATCC<br>GTTCAGATCCCGCAGAACCCGCTGATTCTGGTTGACGGATCTAGTT<br>ACCTGTACCGTGCTTACCATGCTTTCCCGCCTTTGACCAATTCTGCT<br>GGTGAACCTACGGGAGCTATGTACGGAGTTCTGAATATGTTGCGTT<br>CTTTAATTATGCAGTACAAGCCTACCCACGCTGCTGTTGTTTTCGA

	TGCTAAAGGTAAGACGTTCCGCGACGAGTTATTCGAGCACTATAA<br>GTCTCACCGTCCTCCGATGCCTGATGACTTACGCGCTCAGATTGAG<br>CCGCTGCATGCTATGGTGAAGGCTATGGGTTTACCTCTTTTGGCTG<br>TCAGCGGTGTTGAGGCTGATGATGTCATTGGCACCTTAGCTCGTGA<br>GGCTGAGAAGGCTGGTCGCCCTGTTTTGATTTCTACCGGTGACAAG<br>GACATGGCTCAATTGGTTACCCCGAACATCACCCTGATCAACACCA<br>TGACCAACACGATTCTGGGTCCTGAGGAAGTTGTTAACAAATATG<br>GTGTTCCTCCGGAGTTGATTATTGACTTTCTTGCTCTTATGGGCGAT<br>TCTTCAGACAATATCCCGGGTGTTCCAGGTGTTGGAGAGAAGACT<br>GCTCAAGCTCTGCTTCAGGGTCTGGGTGGTTTGGACACCCTTTACG<br>CTGAACCGGAGAAGATCGCCGGTCTGTCTTTTCGCGGTGCTAAGAC<br>CATGGCTGCTAAACTGGAACAGAATAAGGAGGTCGCATACCTGTC<br>TTATCAATTGGCTACCATCAAGACGGATGTGGAGTTAGAACTTACG<br>TGCGAGCAGCTTGAGGTTCAACAGCCTGCTGCTGAGGAACTGCTG<br>GGTCTTTTTAAGAAATACGAATTTAAGCGTTGGACCGCCGACGTTG<br>AGGCTGGTAAGTGGCTGCAAGCTAAGGGTGCTAAGCCGGCTGCTA<br>AACCGCAAGAAACGAGTGTCGCTGATGAGGCTCCGGAGGTTACCG<br>CTACCGTTATCTCTTACGATAATTATGTTACGATTCTGGACGAGGA<br>AACCTTAAAGGCTTGGATCGCTAAATTAGAGAAGGCTCCTGTTTTC<br>GCTTTCGACACGGAAACGGATTCTCTGGACAATATTAGTGCGAATC<br>TTGTTGGTCTGAGTTTCGCAATTGAACCGGGTGTTGCTGCTTACAT<br>CCCTGTGGCACACGACTACCTGGACGCTCCGGACCAGATTTCACGT<br>GAACGCGCTCTGGAACTGCTGAAGCCTTTATTAGAGGACGAGAAA<br>GCTTTGAAAGTTGGTCAGAATTTGAAGTATGCTCGTGGAATCTTAG<br>CTAATTATGGTATCGAGTTGCGCGGTATCGCTTTCGACACGATGTT<br>GGAATCTTATATCCTGAACTCTGTCGCTGGTCGCCATGACATGGAC<br>TCTCTGGCTGAGCGCTGGCTGAAACATAAGACGATTACCTTCGAG<br>GAAATCGCAGGAAAGGGTAAGAACCAGCTCACGTTCAATCAAATC<br>GCTCTGGAGGAAGCTGGTCGCTATGCTGCTGAGGACGCTGACGTT<br>ACTCTGCAACTGCACTTGAAGATGTGGCCTGACTTGCAGAAGCAT<br>AAGGGTCCACTGAATGTTTTTGAAAACATTGAGATGCCTTTGGTTC<br>CAGTTCTGTCTCGTATCGAGCGCAATGGCGTTAAAATTGACCCAAA<br>GGTTTTACATAACCACTCAGAGGAACTGACGCTGCGCTTAGCCGA<br>ATTGGAGAAAAAGGCTCACGAGATCGCTGGCGAAGAGTTCAATCT<br>GTCATCTACGAAACAACTGCAGACTATCCTGTTCGAGAAGCAAGG<br>TATCAAGCCATTAAAAAAGACCCCTGGCGGTGCTCCGTCTACCTCT<br>GAGGAAGTTTTGGAGGAGTTAGCTTTGGATTACCCTCTGCCGAAG<br>GTTATCTTGGAATACCGCGGTTTGGCTAAATTGAAGTCTACTTATA<br>CGGATAAACTTCCTTTGATGATTAATCCAAAGACGGGTCGCGTTCA<br>CACGTCGTACCATCAAGCTGTTACCGCTACCGGTCGCCTGTCTTCT<br>ACGGATCCGAATTTACAGAATATTCCTGTGCGCAATGAGGAGGGC<br>CGCCGCATTCGTCAAGCTTTTATCGCTCCGGAAGACTACGTTATCG<br>TTTCTGCTGATTATTCTCAAAATGAATTACGTATCATGGCTCACCT<br>GTCTCGCGATAAGGGTCTGTTGACGGCCTTTGCTGAGGGTAAGGA<br>CATTCATCGTGCTACCGCTGCTGAGGTTTACGGCCTGCCGTTGGAA<br>ACGGTTACGTCTGAACAGCGTCGCTCTGCTAAGCGTATTAATTTCG

	GCTTAATCTACGGTATGTCTGCGTTTGGCTTAGCTCGTCAGCTGAA<br>TATCCCGCGCAAGGAAGCTCAAAAATATATGGATCTGTATTTTGAG<br>CGTTACCACGGTGTTTTGGAATACATGGAGCGTACGCGCGCGCAA<br>GCTAAGGAACAAGGTTATGTGGAAACCTTGGATGGTCGTCGCTTG<br>TACTTGCCTGACATTAAGTCTTCTAACGGCGCCCGCCGCGCTGCTG<br>CCGAGCGCGCTGCTATCAATGCTCCGATGCAAGGTACTGCTGCTGA<br>TATTATTAAGCGTGCTATGATCGCTGTGGACGCTTGGCTGCAAGCT<br>GAACAGCCTCGCGTTCGCATGATTATGCAAGTTCATGACGAGTTGG<br>TTTTCGAGGTGCATAAGGACGACGTGGACGCTGTTGCTAAACAAA<br>TCCACCAGTTGATGGAGAATTGCACGCGCTTAGACGTTCCGCTGCT<br>GGTTGAAGTTGGTTCTGGTGAAAACTGGGACCAGGCTCACTAATA<br>ATGGCTGAAAACTGGGACCAGGCTCACTAACTCAGATCCTACTAG<br>GTTTAATAAACATCTTTATTTTCATTACATCTGTGTGTTGGTTTTTT<br>GTGTGGTACCCAATTCGCCCTATAGTGAGTCGTATTACTCACTGGC<br>CGTCGTTTTACAACGTCGTGACTGGGAAAACCCTGGCGTTACCCAA<br>CTTAATCGCCTTGCAGCACATCCCCCTTTCGCCAGCTGGCGTAATA<br>GCGAAGAGGCCCGCACCGATCGCCCTTCCCAACAGTTGCGCAGCC<br>TGAATGGCGAATGGGACGCGCCCTGTAGCGGCGCATTAAGCGCGG<br>CGGGTGTGGTGGTTACGCGCAGCGTGACCGCTACACTTGCCAGCG<br>CCCTAGCGCCCGCTCCTTTCGCTTTCTTCCCTTCCTTTCTCGCCACG<br>TTCGCCGGCTTTCCCCGTCAAGCTCTAAATCGGGGGCTCCCTTTAG<br>GGTTCCGATTTAGTGCTTTACGGCACCTCGACCCCAAAAAACTTGA<br>TTAGGGTGATGGTTCACGTAGTGGGCCATCGCCCTGATAGACGGTT<br>TTTCGCCCTTTGACGTTGGAGTCCACGTTCTTTAATAGTGGACTCTT<br>GTTCCAAACTGGAACAACACTCAACCCTATCTCGGTCTATTCTTTT<br>GATTTATAAGGGATTTTGCCGATTTCGGCCTATTGGTTAAAAAATG<br>AGCTGATTTAACAAAAATTTAACGCGAATTTTAACAAAATATTAAC<br>GCTTACAATTTAGGTGGCACTTTTCGGGGAAATGTGTAATGGCTGA<br>AAACTGGGACCAGGCTCACTAACTCAGATCCTACTAGGTTTAATA<br>AACATCTTTATTTTCATTACATCTGTGTGTTGGTTTTTTGTGTGGTA<br>CCCAATTCGCCCTATAGTGAGTCGTATTACTCACTGGCCGTCGTTT<br>TACAACGTCGTGACTGGGAAAACCCTGGCGTTACCCAACTTAATC<br>GCCTTGCAGCACATCCCCCTTTCGCCAGCTGGCGTAATAGCGAAGA<br>GGCCCGCACCGATCGCCCTTCCCAACAGTTGCGCAGCCTGAATGG<br>CGAATGGGACGCGCCCTGTAGCGGCGCATTAAGCGCGGCGGGTGT<br>GGTGGTTACGCGCAGCGTGACCGCTACACTTGCCAGCGCCCTAGC<br>GCCCGCTCCTTTCGCTTTCTTCCCTTCCTTTCTCGCCACGTTCGCCG<br>GCTTTCCCCGTCAAGCTCTAAATCGGGGGCTCCCTTTAGGGTTCCG<br>ATTTAGTGCTTTACGGCACCTCGACCCCAAAAAACTTGATTAGGGT<br>GATGGTTCACGTAGTGGGCCATCGCCCTGATAGACGGTTTTTCGCC<br>CTTTGACGTTGGAGTCCACGTTCTTTAATAGTGGACTCTTGTTCCA<br>AACTGGAACAACACTCAACCCTATCTCGGTCTATTCTTTTGATTTA<br>TAAGGGATTTTGCCGATTTCGGCCTATTGGTTAAAAAATGAGCTGA<br>TTTAACAAAAATTTAACGCGAATTTTAACAAAATATTAACGCTTAC<br>AATTTAGGTGGCACTTTTCGGGGAAATGTG

	**Ampicillin resistance cassette-ColE1 origin of replication-hU6 promoter-gRNA sequence-SpCas9 gRNA scaffold-CMV promoter-NLS-mCherry-GSGM-NLS-linker-PolI5M-stop-SV40 polyA**
p.nCas9-PolI5M	CGCGGAACCCCTATTTGTTTATTTTTCTAAATACATTCAAATATGTA<br>TCCGCTCATGAGACAATAACCCTGATAAATGCTTCAATAATATTGA<br>AAAAGGAAGAGTATGAGTATTCAACATTTCCGTGTCGCCCTTATTC<br>CCTTTTTTGCGGCATTTTGCCTTCCTGTTTTTGCTCACCCAGAAACG<br>CTGGTGAAAGTAAAAGATGCTGAAGATCAGTTGGGTGCACGAGTG<br>GGTTACATCGAACTGGATCTCAACAGCGGTAAGATCCTTGAGAGT<br>TTTCGCCCCGAAGAACGTTTTCCAATGATGAGCACTTTTAAAGTTC<br>TGCTATGTGGCGCGGTATTATCCCGTATTGACGCCGGGCAAGAGC<br>AACTCGGTCGCCGCATACACTATTCTCAGAATGACTTGGTTGAGTA<br>CTCACCAGTCACAGAAAAGCATCTTACGGATGGCATGACAGTAAG<br>AGAATTATGCAGTGCTGCCATAACCATGAGTGATAACACTGCGGC<br>CAACTTACTTCTGACAACGATCGGAGGACCGAAGGAGCTAACCGC<br>TTTTTTGCACAACATGGGGGATCATGTAACTCGCCTTGATCGTTGG<br>GAACCGGAGCTGAATGAAGCCATACCAAACGACGAGCGTGACACC<br>ACGATGCCTGTAGCAATGGCAACAACGTTGCGCAAACTATTAACT<br>GGCGAACTACTTACTCTAGCTTCCCGGCAACAATTAATAGACTGGA<br>TGGAGGCGGATAAAGTTGCAGGACCACTTCTGCGCTCGGCCCTTCC<br>GGCTGGCTGGTTTATTGCTGATAAATCTGGAGCCGGTGAGCGTGGT<br>TCTCGCGGTATCATTGCAGCACTGGGGCCAGATGGTAAGCCCTCCC<br>GTATCGTAGTTATCTACACGACGGGGAGTCAGGCAACTATGGATG<br>AACGAAATAGACAGATCGCTGAGATAGGTGCCTCACTGATTAAGC<br>ATTGGTAACTGTCAGACCAAGTTTACTCATATATACTTTAGATTGA<br>TTTAAAACTTCATTTTTAATTTAAAAGGATCTAGGTGAAGATCCTT<br>TTTGATAATCTCATGACCAAAATCCCTTAACGTGAGTTTTCGTTCC<br>ACTGAGCGTCAGACCCCGTAGAAAAGATCAAAGGATCTTCTTGAG<br>ATCCTTTTTTTCTGCGCGTAATCTGCTGCTTGCAAACAAAAAAACC<br>ACCGCTACCAGCGGTGGTTTGTTTGCCGGATCAAGAGCTACCAACT<br>CTTTTTCCGAAGGTAACTGGCTTCAGCAGAGCGCAGATACCAAAT<br>ACTGTCCTTCTAGTGTAGCCGTAGTTAGGCCACCACTTCAAGAACT<br>CTGTAGCACCGCCTACATACCTCGCTCTGCTAATCCTGTTACCAGT<br>GGCTGCTGCCAGTGGCGATAAGTCGTGTCTTACCGGGTTGGACTCA<br>AGACGATAGTTACCGGATAAGGCGCAGCGGTCGGGCTGAACGGGG<br>GGTTCGTGCACACAGCCCAGCTTGGAGCGAACGACCTACACCGAA<br>CTGAGATACCTACAGCGTGAGCTATGAGAAAGCGCCACGCTTCCC<br>GAAGGGAGAAAGGCGGACAGGTATCCGGTAAGCGGCAGGGTCGG<br>AACAGGAGAGCGCACGAGGGAGCTTCCAGGGGGAAACGCCTGGT<br>ATCTTTATAGTCCTGTCGGGTTTCGCCACCTCTGACTTGAGCGTCG<br>ATTTTTGTGATGCTCGTCAGGGGGGCGGAGCCTATGGAAAAACGC<br>CAGCAACGCGGCCTTTTTACGGTTCCTGGCCTTTTGCTGGCCTTTTG<br>CTCACATGTTCTTTCCTGCGTTATCCCCTGATTCTGTGGATAACCGT<br>ATTACCGCCTTTGAGTGAGCTGATACCGCTCGCCGCAGCCGAACG

	ACCGAGCGCAGCGAGTCAGTGAGCGAGGAAGCGGAAGAGCGCCC<br>AATACGCAAACCGCCTCTCCCCGCGCGTTGGCCGATTCATTAATGC<br>AGCTGGCACGACAGGTTTCCCGACTGGAAAGCGGGCAGTGAGCGC<br>AACGCAATTAATGTGAGTTAGCTCACTCATTAGGCACCCCAGGCTT<br>TACACTTTATGCTTCCGGCTCGTATGTTGTGTGGAATTGTGAGCGG<br>ATAACAATTTCACACAGGAAACAGCTATGACCATGATTACGCCAA<br>GCGCGCAATTAACCCTCACTAAAGGGAACAAAAGCTGGAGCTCCA<br>CCGCGGTGGCGGCCGCTCTTAAGGGGTGCAGCGGCCTCCGCGCCG<br>GGTTTTGGCGCCTCGATCCAAGGTCGGGCAGGAAGAGGGCCTATT<br>TCCCATGATTCCTTCATATTTGCATATACGATACAAGGCTGTTAGA<br>GAGATAATTAGAATTAATTTGACTGTAAACACAAAGATATTAGTA<br>CAAAATACGTGACGTAGAAAGTAATAATTTCTTGGGTAGTTTGCA<br>GTTTTAAAATTATGTTTTAAAATGGACTATCATATGCTTACCGTAA<br>CTTGAAAGTATTTCGATTTCTTGGCTTTATATATCTTGTGGAAAGG<br>ACGAAACACC-[gRNA SEQUENCE]-<br>GTTTTAGAGCTAGAAATAGCAAGTTAAAATAAGGCTAGTCCGTTA<br>TCAACTTGAAAAAGTGGCACCGAGTCGGTGCTTTTTTCCGCGGCCT<br>CTAGACTCGAGGCGTTGACATTGATTATTGACTAGTTATTAATAGT<br>AATCAATTACGGGGTCATTAGTTCATAGCCCATATATGGAGTTCCG<br>CGTTACATAACTTACGGTAAATGGCCCGCCTGGCTGACCGCCCAAC<br>GACCCCCGCCCATTGACGTCAATAATGACGTATGTTCCCATAGTAA<br>CGCCAATAGGGACTTTCCATTGACGTCAATGGGTGGAGTATTTACG<br>GTAAACTGCCCACTTGGCAGTACATCAAGTGTATCATATGCCAAGT<br>ACGCCCCCTATTGACGTCAATGACGGTAAATGGCCCGCCTGGCATT<br>ATGCCCAGTACATGACCTTATGGGACTTTCCTACTTGGCAGTACAT<br>CTACGTATTAGTCATCGCTATTACCATGGTGATGCGGTTTTGGCAG<br>TACATCAATGGGCGTGGATAGCGGTTTGACTCACGGGGATTTCCA<br>AGTCTCCACCCCATTGACGTCAATGGGAGTTTGTTTTGGCACCAAA<br>ATCAACGGGACTTTCCAAAATGTCGTAACAACTCCGCCCCATTGAC<br>GCAAATGGGCGGTAGGCGTGTACGGTGGGAGGTCTATATAAGCAG<br>AGCTCTCTGGCTAACTACCGGTGCCACCATGGCCCCAAAGAAGAA<br>GCGGAAGGTCGGTATCCACGGAGTCCCAGCAGCCGTGAGCAAGGG<br>CGAGGAGGATAACATGGCCATCATCAAGGAGTTCATGCGCTTCAA<br>GGTGCACATGGAGGGCTCCGTGAACGGCCACGAGTTCGAGATCGA<br>GGGCGAGGGCGAGGGCCGCCCCTACGAGGGCACCCAGACCGCCA<br>AGCTGAAGGTGACCAAGGGTGGCCCCCTGCCCTTCGCCTGGGACA<br>TCCTGTCCCCTCAGTTCATGTACGGCTCCAAGGCCTACGTGAAGCA<br>CCCCGCCGACATCCCCGACTACTTGAAGCTGTCCTTCCCCGAGGGC<br>TTCAAGTGGGAGCGCGTGATGAACTTCGAGGACGGCGGCGTGGTG<br>ACCGTGACCCAGGACTCCTCCCTGCAGGACGGCGAGTTCATCTAC<br>AAGGTGAAGCTGCGCGGCACCAACTTCCCCTCCGACGGCCCCGTA<br>ATGCAGAAGAAGACCATGGGCTGGGAGGCCTCCTCCGAGCGGATG<br>TACCCCGAGGACGGCGCCCTGAAGGGCGAGATCAAGCAGAGGCTG<br>AAGCTGAAGGACGGCGGCCACTACGACGCTGAGGTCAAGACCACC<br>TACAAGGCCAAGAAGCCCGTGCAGCTGCCCGGCGCCTACAACGTC<br>AACATCAAGTTGGACATCACCTCCCACAACGAGGACTACACCATC

	GTGGAACAGTACGAACGCGCCGAGGGCCGCCACTCCACCGGCGGC<br>ATGGACGAGCTGTACAAGGGATCCGGTATGGACAAGAAGTACAGC<br>ATCGGCCTGGCCATCGGCACCAACTCTGTGGGCTGGGCCGTGATC<br>ACCGACGAGTACAAGGTGCCCAGCAAGAAATTCAAGGTGCTGGGC<br>AACACCGACCGGCACAGCATCAAGAAGAACCTGATCGGCGCCCTG<br>CTGTTCGACAGCGGAGAAACAGCCGAGGCCACCCGGCTGAAGAGA<br>ACCGCCAGAAGAAGATACACCAGACGGAAGAACCGGATCTGCTAT<br>CTGCAAGAGATCTTCAGCAACGAGATGGCCAAGGTGGACGACAGC<br>TTCTTCCACAGACTGGAAGAGTCCTTCCTGGTGGAAGAGGATAAG<br>AAGCACGAGCGGCACCCCATCTTCGGCAACATCGTGGACGAGGTG<br>GCCTACCACGAGAAGTACCCCACCATCTACCACCTGAGAAAGAAA<br>CTGGTGGACAGCACCGACAAGGCCGACCTGCGGCTGATCTATCTG<br>GCCCTGGCCCACATGATCAAGTTCCGGGGCCACTTCCTGATCGAGG<br>GCGACCTGAACCCCGACAACAGCGACGTGGACAAGCTGTTCATCC<br>AGCTGGTGCAGACCTACAACCAGCTGTTCGAGGAAAACCCCATCA<br>ACGCCAGCGGCGTGGACGCCAAGGCCATCCTGTCTGCCAGACTGA<br>GCAAGAGCAGACGGCTGGAAAATCTGATCGCCCAGCTGCCCGGCG<br>AGAAGAAGAATGGCCTGTTCGGCAACCTGATTGCCCTGAGCCTGG<br>GCCTGACCCCCAACTTCAAGAGCAACTTCGACCTGGCCGAGGATG<br>CCAAACTGCAGCTGAGCAAGGACACCTACGACGACGACCTGGACA<br>ACCTGCTGGCCCAGATCGGCGACCAGTACGCCGACCTGTTTCTGGC<br>CGCCAAGAACCTGTCCGACGCCATCCTGCTGAGCGACATCCTGAG<br>AGTGAACACCGAGATCACCAAGGCCCCCCTGAGCGCCTCTATGAT<br>CAAGAGATACGACGAGCACCACCAGGACCTGACCCTGCTGAAAGC<br>TCTCGTGCGGCAGCAGCTGCCTGAGAAGTACAAAGAGATTTTCTTC<br>GACCAGAGCAAGAACGGCTACGCCGGCTACATCGATGGCGGAGCC<br>AGCCAGGAAGAGTTCTACAAGTTCATCAAGCCCATCCTGGAAAAG<br>ATGGACGGCACCGAGGAACTGCTCGTGAAGCTGAACAGAGAGGA<br>CCTGCTGCGGAAGCAGCGGACCTTCGACAACGGCAGCATCCCCCA<br>CCAGATCCACCTGGGAGAGCTGCACGCCATTCTGCGGCGGCAGGA<br>AGATTTTTACCCATTCCTGAAGGACAACCGGGAAAAGATCGAGAA<br>GATCCTGACCTTCCGCATCCCCTACTACGTGGGCCCTCTGGCCAGG<br>GGAAACAGCAGATTCGCCTGGATGACCAGAAAGAGCGAGGAAAC<br>CATCACCCCCTGGAACTTCGAGGAAGTGGTGGACAAGGGCGCCAG<br>CGCCCAGAGCTTCATCGAGCGGATGACCAACTTCGATAAGAACCT<br>GCCCAACGAGAAGGTGCTGCCCAAGCACAGCCTGCTGTACGAGTA<br>CTTCACCGTGTACAACGAGCTGACCAAAGTGAAATACGTGACCGA<br>GGGAATGAGAAAGCCCGCCTTCCTGAGCGGCGAGCAGAAAAAG<br>CCATCGTGGACCTGCTGTTCAAGACCAACCGGAAAGTGACCGTGA<br>AGCAGCTGAAAGAGGACTACTTCAAGAAAATCGAGTGCTTCGACT<br>CCGTGGAAATCTCCGGCGTGGAAGATCGGTTCAACGCCTCCCTGG<br>GCACATACCACGATCTGCTGAAAATTATCAAGGACAAGGACTTCC<br>TGGACAATGAGGAAAACGAGGACATTCTGGAAGATATCGTGCTGA<br>CCCTGACACTGTTTGAGGACAGAGAGATGATCGAGGAACGGCTGA<br>AAACCTATGCCCACCTGTTCGACGACAAAGTGATGAAGCAGCTGA<br>AGCGGCGGAGATACACCGGCTGGGGCAGGCTGAGCCGGAAGCTG

	ATCAACGGCATCCGGGACAAGCAGTCCGGCAAGACAATCCTGGAT<br>TTCCTGAAGTCCGACGGCTTCGCCAACAGAAACTTCATGCAGCTGA<br>TCCACGACGACAGCCTGACCTTTAAAGAGGACATCCAGAAAGCCC<br>AGGTGTCCGGCCAGGGCGATAGCCTGCACGAGCACATTGCCAATC<br>TGGCCGGCAGCCCCGCCATTAAGAAGGGCATCCTGCAGACAGTGA<br>AGGTGGTGGACGAGCTCGTGAAAGTGATGGGCCGGCACAAGCCCG<br>AGAACATCGTGATCGAAATGGCCAGAGAGAACCAGACCACCCAG<br>AAGGGACAGAAGAACAGCCGCGAGAGAATGAAGCGGATCGAAGA<br>GGGCATCAAAGAGCTGGGCAGCCAGATCCTGAAAGAACACCCCGT<br>GGAAAACACCCAGCTGCAGAACGAGAAGCTGTACCTGTACTACCT<br>GCAGAATGGGCGGGATATGTACGTGGACCAGGAACTGGACATCAA<br>CCGGCTGTCCGACTACGATGTGGACCATATCGTGCCTCAGAGCTTT<br>CTGAAGGACGACTCCATCGATAACAAAGTGCTGACTCGGAGCGAC<br>AAGAACCGGGGCAAGAGCGACAACGTGCCCTCCGAAGAGGTCGT<br>GAAGAAGATGAAGAACTACTGGCGCCAGCTGCTGAATGCCAAGCT<br>GATTACCCAGAGGAAGTTCGACAATCTGACCAAGGCCGAGAGAGG<br>CGGCCTGAGCGAACTGGATAAGGCCGGCTTCATCAAGAGACAGCT<br>GGTGGAAACCCGGCAGATCACAAAGCACGTGGCACAGATCCTGGA<br>CTCCCGGATGAACACTAAGTACGACGAGAACGACAAACTGATCCG<br>GGAAGTGAAAGTGATCACCCTGAAGTCCAAGCTGGTGTCCGATTT<br>CCGGAAGGATTTCCAGTTTTACAAAGTGCGCGAGATCAACAACTA<br>CCACCACGCCCACGACGCCTACCTGAACGCCGTCGTGGGAACCGC<br>CCTGATCAAAAAGTACCCTAAGCTGGAAAGCGAGTTCGTGTACGG<br>CGACTACAAGGTGTACGACGTGCGGAAGATGATCGCCAAGAGCGA<br>GCAGGAAATCGGCAAGGCTACCGCCAAGTACTTCTTCTACAGCAA<br>CATCATGAACTTTTTCAAGACCGAGATTACCCTGGCCAACGGCGA<br>GATCCGGAAGCGGCCTCTGATCGAGACAAACGGCGAAACAGGCG<br>AGATCGTGTGGGATAAGGGCCGGGACTTTGCCACCGTGCGGAAAG<br>TGCTGTCTATGCCCCAAGTGAATATCGTGAAAAAGACCGAGGTGC<br>AGACAGGCGGCTTCAGCAAAGAGTCTATCCTGCCCAAGAGGAACA<br>GCGACAAGCTGATCGCCAGAAAGAAGGACTGGGACCCTAAGAAG<br>TACGGCGGCTTCGACAGCCCCACCGTGGCCTATTCTGTGCTGGTGG<br>TGGCCAAAGTGGAAAAGGGCAAGTCCAAGAAACTGAAGAGTGTG<br>AAAGAGCTGCTGGGGATCACCATCATGGAAAGAAGCAGCTTCGAG<br>AAGAATCCCATCGACTTTCTGGAAGCCAAGGGCTACAAAGAAGTG<br>AAAAAGGACCTGATCATCAAGCTGCCTAAGTACTCCCTGTTCGAG<br>CTGGAAAACGGCCGGAAGAGAATGCTGGCCTCTGCCGGCGAACTG<br>CAGAAGGGAAACGAACTGGCCCTGCCCTCCAAATATGTGAACTTC<br>CTGTACCTGGCCAGCCACTATGAGAAGCTGAAGGGCTCCCCCGAG<br>GATAATGAGCAGAAACAGCTGTTTGTGGAACAGCACAAACACTAC<br>CTGGACGAGATCATCGAGCAGATCAGCGAGTTCTCCAAGAGAGTG<br>ATCCTGGCCGACGCTAATCTGGACAAGGTGCTGAGCGCCTACAAC<br>AAGCACAGAGACAAGCCTATCAGAGAGCAGGCCGAGAATATCATC<br>CACCTGTTTACCCTGACCAATCTGGGAGCCCCTGCCGCCTTCAAGT<br>ACTTTGACACCACCATCGACCGGAAGAGGTACACCAGCACCAAAG<br>AGGTGCTGGACGCCACCCTGATCCACCAGAGCATCACCGGCCTGT

	ACGAGACACGGATCGACCTGTCTCAGCTGGGAGGCGACGCCTATC<br>CCTATGACGTGCCCGATTATGCCAGCCTGGGCAGCGGCTCCCCCAA<br>GAAAAAACGCAAGGTGGAAGATCCTAAGAAAAAGCGGAAAGGTT<br>CTAGTGAAACCCCGGGAACAAGTGAGTCGGCCACCCCTGAAGGTG<br>GATCAGGGGGTAGCGGATCCGTTCAGATCCCGCAGAACCCGCTGA<br>TTCTGGTTGACGGATCTAGTTACCTGTACCGTGCTTACCATGCTTTC<br>CCGCCTTTGACCAATTCTGCTGGTGAACCTACGGGAGCTATGTACG<br>GAGTTCTGAATATGTTGCGTTCTTTAATTATGCAGTACAAGCCTAC<br>CCACGCTGCTGTTGTTTTCGATGCTAAAGGTAAGACGTTCCGCGAC<br>GAGTTATTCGAGCACTATAAGTCTCACCGTCCTCCGATGCCTGATG<br>ACTTACGCGCTCAGATTGAGCCGCTGCATGCTATGGTGAAGGCTAT<br>GGGTTTACCTCTTTTGGCTGTCAGCGGTGTTGAGGCTGATGATGTC<br>ATTGGCACCTTAGCTCGTGAGGCTGAGAAGGCTGGTCGCCCTGTTT<br>TGATTTCTACCGGTGACAAGGACATGGCTCAATTGGTTACCCCGAA<br>CATCACCCTGATCAACACCATGACCAACACGATTCTGGGTCCTGAG<br>GAAGTTGTTAACAAATATGGTGTTCCTCCGGAGTTGATTATTGACT<br>TTCTTGCTCTTATGGGCGATTCTTCAGACAATATCCCGGGTGTTCC<br>AGGTGTTGGAGAGAAGACTGCTCAAGCTCTGCTTCAGGGTCTGGG<br>TGGTTTGGACACCCTTTACGCTGAACCGGAGAAGATCGCCGGTCTG<br>TCTTTTCGCGGTGCTAAGACCATGGCTGCTAAACTGGAACAGAATA<br>AGGAGGTCGCATACCTGTCTTATCAATTGGCTACCATCAAGACGG<br>ATGTGGAGTTAGAACTTACGTGCGAGCAGCTTGAGGTTCAACAGC<br>CTGCTGCTGAGGAACTGCTGGGTCTTTTTAAGAAATACGAATTTAA<br>GCGTTGGACCGCCGACGTTGAGGCTGGTAAGTGGCTGCAAGCTAA<br>GGGTGCTAAGCCGGCTGCTAAACCGCAAGAAACGAGTGTCGCTGA<br>TGAGGCTCCGGAGGTTACCGCTACCGTTATCTCTTACGATAATTAT<br>GTTACGATTCTGGACGAGGAAACCTTAAAGGCTTGGATCGCTAAA<br>TTAGAGAAGGCTCCTGTTTTCGCTTTCGACACGGAAACGGATTCTC<br>TGGACAATATTAGTGCGAATCTTGTTGGTCTGAGTTTCGCAATTGA<br>ACCGGGTGTTGCTGCTTACATCCCTGTGGCACACGACTACCTGGAC<br>GCTCCGGACCAGATTTCACGTGAACGCGCTCTGGAACTGCTGAAG<br>CCTTTATTAGAGGACGAGAAAGCTTTGAAAGTTGGTCAGAATTTG<br>AAGTATGCTCGTGGAATCTTAGCTAATTATGGTATCGAGTTGCGCG<br>GTATCGCTTTCGACACGATGTTGGAATCTTATATCCTGAACTCTGT<br>CGCTGGTCGCCATGACATGGACTCTCTGGCTGAGCGCTGGCTGAA<br>ACATAAGACGATTACCTTCGAGGAAATCGCAGGAAAGGGTAAGAA<br>CCAGCTCACGTTCAATCAAATCGCTCTGGAGGAAGCTGGTCGCTAT<br>GCTGCTGAGGACGCTGACGTTACTCTGCAACTGCACTTGAAGATGT<br>GGCCTGACTTGCAGAAGCATAAGGGTCCACTGAATGTTTTTGAAA<br>ACATTGAGATGCCTTTGGTTCCAGTTCTGTCTCGTATCGAGCGCAA<br>TGGCGTTAAAATTGACCCAAAGGTTTTACATAACCACTCAGAGGA<br>ACTGACGCTGCGCTTAGCCGAATTGGAGAAAAAGGCTCACGAGAT<br>CGCTGGCGAAGAGTTCAATCTGTCATCTACGAAACAACTGCAGAC<br>TATCCTGTTCGAGAAGCAAGGTATCAAGCCATTAAAAAAGACCCC<br>TGGCGGTGCTCCGTCTACCTCTGAGGAAGTTTTGGAGGAGTTAGCT<br>TTGGATTACCCTCTGCCGAAGGTTATCTTGGAATACCGCGGTTTGG

	CTAAATTGAAGTCTACTTATACGGATAAACTTCCTTTGATGATTAA<br>TCCAAAGACGGGTCGCGTTCACACGTCGTACCATCAAGCTGTTACC<br>GCTACCGGTCGCCTGTCTTCTACGGATCCGAATTTACAGAATATTC<br>CTGTGCGCAATGAGGAGGGCCGCCGCATTCGTCAAGCTTTTATCGC<br>TCCGGAAGACTACGTTATCGTTTCTGCTGATTATTCTCAAAATGAA<br>TTACGTATCATGGCTCACCTGTCTCGCGATAAGGGTCTGTTGACGG<br>CCTTTGCTGAGGGTAAGGACATTCATCGTGCTACCGCTGCTGAGGT<br>TTACGGCCTGCCGTTGGAAACGGTTACGTCTGAACAGCGTCGCTCT<br>GCTAAGCGTATTAATTTCGGCTTAATCTACGGTATGTCTGCGTTTG<br>GCTTAGCTCGTCAGCTGAATATCCCGCGCAAGGAAGCTCAAAAAT<br>ATATGGATCTGTATTTTGAGCGTTACCACGGTGTTTTGGAATACAT<br>GGAGCGTACGCGCGCGCAAGCTAAGGAACAAGGTTATGTGGAAAC<br>CTTGGATGGTCGTCGCTTGTACTTGCCTGACATTAAGTCTTCTAAC<br>GGCGCCCGCCGCGCTGCTGCCGAGCGCGCTGCTATCAATGCTCCG<br>ATGCAAGGTACTGCTGCTGATATTATTAAGCGTGCTATGATCGCTG<br>TGGACGCTTGGCTGCAAGCTGAACAGCCTCGCGTTCGCATGATTAT<br>GCAAGTTCATGACGAGTTGGTTTTCGAGGTGCATAAGGACGACGT<br>GGACGCTGTTGCTAAACAAATCCACCAGTTGATGGAGAATTGCAC<br>GCGCTTAGACGTTCCGCTGCTGGTTGAAGTTGGTTCTGGTGAAAAC<br>TGGGACCAGGCTCACTAATAATGGCTGAAAACTGGGACCAGGCTC<br>ACTAACTCAGATCCTACTAGGTTTAATAAACATCTTTATTTTCATTA<br>CATCTGTGTGTTGGTTTTTTGTGTGGTACCCAATTCGCCCTATAGTG<br>AGTCGTATTACTCACTGGCCGTCGTTTTACAACGTCGTGACTGGGA<br>AAACCCTGGCGTTACCCAACTTAATCGCCTTGCAGCACATCCCCCT<br>TTCGCCAGCTGGCGTAATAGCGAAGAGGCCCGCACCGATCGCCCT<br>TCCCAACAGTTGCGCAGCCTGAATGGCGAATGGGACGCGCCCTGT<br>AGCGGCGCATTAAGCGCGGCGGGTGTGGTGGTTACGCGCAGCGTG<br>ACCGCTACACTTGCCAGCGCCCTAGCGCCCGCTCCTTTCGCTTTCTT<br>CCCTTCCTTTCTCGCCACGTTCGCCGGCTTTCCCCGTCAAGCTCTAA<br>ATCGGGGGCTCCCTTTAGGGTTCCGATTTAGTGCTTTACGGCACCT<br>CGACCCCAAAAAACTTGATTAGGGTGATGGTTCACGTAGTGGGCC<br>ATCGCCCTGATAGACGGTTTTTCGCCCTTTGACGTTGGAGTCCACG<br>TTCTTTAATAGTGGACTCTTGTTCCAAACTGGAACAACACTCAACC<br>CTATCTCGGTCTATTCTTTTGATTTATAAGGGATTTTGCCGATTTCG<br>GCCTATTGGTTAAAAAATGAGCTGATTTAACAAAAATTTAACGCG<br>AATTTTAACAAAATATTAACGCTTACAATTTAGGTGGCACTTTTCG<br>GGGAAATGTG<br><br>**Ampicillin resistance cassette-ColE1 origin of replication-hU6 promoter-gRNA sequence-SpCas9 gRNA scaffold-CMV promoter-NLS-mCherry-GSGM- nCas9(D10A)-NLS-linker-PolI5M-stop-SV40 polyA**
p.enCas9(H840A)-PolI5M	CGCGGAACCCCTATTTGTTTATTTTTCTAAATACATTCAAATATGTA<br>TCCGCTCATGAGACAATAACCCTGATAAATGCTTCAATAATATTGA<br>AAAAGGAAGAGTATGAGTATTCAACATTTCCGTGTCGCCCTTATTC

	CCTTTTTTGCGGCATTTTGCCTTCCTGTTTTTGCTCACCCAGAAACG<br>CTGGTGAAAGTAAAAGATGCTGAAGATCAGTTGGGTGCACGAGTG<br>GGTTACATCGAACTGGATCTCAACAGCGGTAAGATCCTTGAGAGT<br>TTTCGCCCCGAAGAACGTTTTCCAATGATGAGCACTTTTAAAGTTC<br>TGCTATGTGGCGCGGTATTATCCCGTATTGACGCCGGGCAAGAGC<br>AACTCGGTCGCCGCATACACTATTCTCAGAATGACTTGGTTGAGTA<br>CTCACCAGTCACAGAAAAGCATCTTACGGATGGCATGACAGTAAG<br>AGAATTATGCAGTGCTGCCATAACCATGAGTGATAACACTGCGGC<br>CAACTTACTTCTGACAACGATCGGAGGACCGAAGGAGCTAACCGC<br>TTTTTTGCACAACATGGGGGATCATGTAACTCGCCTTGATCGTTGG<br>GAACCGGAGCTGAATGAAGCCATACCAAACGACGAGCGTGACACC<br>ACGATGCCTGTAGCAATGGCAACAACGTTGCGCAAACTATTAACT<br>GGCGAACTACTTACTCTAGCTTCCCGGCAACAATTAATAGACTGGA<br>TGGAGGCGGATAAAGTTGCAGGACCACTTCTGCGCTCGGCCCTTCC<br>GGCTGGCTGGTTTATTGCTGATAAATCTGGAGCCGGTGAGCGTGGT<br>TCTCGCGGTATCATTGCAGCACTGGGGCCAGATGGTAAGCCCTCCC<br>GTATCGTAGTTATCTACACGACGGGGAGTCAGGCAACTATGGATG<br>AACGAAATAGACAGATCGCTGAGATAGGTGCCTCACTGATTAAGC<br>ATTGGTAACTGTCAGACCAAGTTTACTCATATATACTTTAGATTGA<br>TTTAAAACTTCATTTTTAATTTAAAAGGATCTAGGTGAAGATCCTT<br>TTTGATAATCTCATGACCAAAATCCCTTAACGTGAGTTTTCGTTCC<br>ACTGAGCGTCAGACCCCGTAGAAAAGATCAAAGGATCTTCTTGAG<br>ATCCTTTTTTTCTGCGCGTAATCTGCTGCTTGCAAACAAAAAAACC<br>ACCGCTACCAGCGGTGGTTTGTTTGCCGGATCAAGAGCTACCAACT<br>CTTTTTCCGAAGGTAACTGGCTTCAGCAGAGCGCAGATACCAAAT<br>ACTGTCCTTCTAGTGTAGCCGTAGTTAGGCCACCACTTCAAGAACT<br>CTGTAGCACCGCCTACATACCTCGCTCTGCTAATCCTGTTACCAGT<br>GGCTGCTGCCAGTGGCGATAAGTCGTGTCTTACCGGGTTGGACTCA<br>AGACGATAGTTACCGGATAAGGCGCAGCGGTCGGGCTGAACGGGG<br>GGTTCGTGCACACAGCCCAGCTTGGAGCGAACGACCTACACCGAA<br>CTGAGATACCTACAGCGTGAGCTATGAGAAAGCGCCACGCTTCCC<br>GAAGGGAGAAAGGCGGACAGGTATCCGGTAAGCGGCAGGGTCGG<br>AACAGGAGAGCGCACGAGGGAGCTTCCAGGGGGAAACGCCTGGT<br>ATCTTTATAGTCCTGTCGGGTTTCGCCACCTCTGACTTGAGCGTCG<br>ATTTTTGTGATGCTCGTCAGGGGGGCGGAGCCTATGGAAAAACGC<br>CAGCAACGCGGCCTTTTTACGGTTCCTGGCCTTTTGCTGGCCTTTTG<br>CTCACATGTTCTTTCCTGCGTTATCCCCTGATTCTGTGGATAACCGT<br>ATTACCGCCTTTGAGTGAGCTGATACCGCTCGCCGCAGCCGAACG<br>ACCGAGCGCAGCGAGTCAGTGAGCGAGGAAGCGGAAGAGCGCCC<br>AATACGCAAACCGCCTCTCCCCGCGCGTTGGCCGATTCATTAATGC<br>AGCTGGCACGACAGGTTTCCCGACTGGAAAGCGGGCAGTGAGCGC<br>AACGCAATTAATGTGAGTTAGCTCACTCATTAGGCACCCCAGGCTT<br>TACACTTTATGCTTCCGGCTCGTATGTTGTGTGGAATTGTGAGCGG<br>ATAACAATTTCACACAGGAAACAGCTATGACCATGATTACGCCAA<br>GCGCGCAATTAACCCTCACTAAAGGGAACAAAAGCTGGAGCTCCA<br>CCGCGGTGGCGGCCGCTCTTAAGGGGTGCAGCGGCCTCCGCGCCG

	GGTTTTGGCGCCTCGATCCAAGGTCGGGCAGGAAGAGGGCCTATT<br>TCCCATGATTCCTTCATATTTGCATATACGATACAAGGCTGTTAGA<br>GAGATAATTAGAATTAATTTGACTGTAAACACAAAGATATTAGTA<br>CAAAATACGTGACGTAGAAAGTAATAATTTCTTGGGTAGTTTGCA<br>GTTTTAAAATTATGTTTTAAAATGGACTATCATATGCTTACCGTAA<br>CTTGAAAGTATTTCGATTTCTTGGCTTTATATATCTTGTGGAAAGG<br>ACGAAACACC-[gRNA SEQUENCE]-<br>GTTTTAGAGCTAGAAATAGCAAGTTAAAATAAGGCTAGTCCGTTA<br>TCAACTTGAAAAAGTGGCACCGAGTCGGTGCTTTTTTCCGCGGCCT<br>CTAGACTCGAGGCGTTGACATTGATTATTGACTAGTTATTAATAGT<br>AATCAATTACGGGGTCATTAGTTCATAGCCCATATATGGAGTTCCG<br>CGTTACATAACTTACGGTAAATGGCCCGCCTGGCTGACCGCCCAAC<br>GACCCCCGCCCATTGACGTCAATAATGACGTATGTTCCCATAGTAA<br>CGCCAATAGGGACTTTCCATTGACGTCAATGGGTGGAGTATTTACG<br>GTAAACTGCCCACTTGGCAGTACATCAAGTGTATCATATGCCAAGT<br>ACGCCCCCTATTGACGTCAATGACGGTAAATGGCCCGCCTGGCATT<br>ATGCCCAGTACATGACCTTATGGGACTTTCCTACTTGGCAGTACAT<br>CTACGTATTAGTCATCGCTATTACCATGGTGATGCGGTTTTGGCAG<br>TACATCAATGGGCGTGGATAGCGGTTTGACTCACGGGGATTTCCA<br>AGTCTCCACCCCATTGACGTCAATGGGAGTTTGTTTTGGCACCAAA<br>ATCAACGGGACTTTCCAAAATGTCGTAACAACTCCGCCCCATTGAC<br>GCAAATGGGCGGTAGGCGTGTACGGTGGGAGGTCTATATAAGCAG<br>AGCTCTCTGGCTAACTACCGGTGCCACCATGGCCCCAAAGAAGAA<br>GCGGAAGGTCGGTATCCACGGAGTCCCAGCAGCCGTGAGCAAGGG<br>CGAGGAGGATAACATGGCCATCATCAAGGAGTTCATGCGCTTCAA<br>GGTGCACATGGAGGGCTCCGTGAACGGCCACGAGTTCGAGATCGA<br>GGGCGAGGGCGAGGGCCGCCCCTACGAGGGCACCCAGACCGCCA<br>AGCTGAAGGTGACCAAGGGTGGCCCCCTGCCCTTCGCCTGGGACA<br>TCCTGTCCCCTCAGTTCATGTACGGCTCCAAGGCCTACGTGAAGCA<br>CCCCGCCGACATCCCCGACTACTTGAAGCTGTCCTTCCCCGAGGGC<br>TTCAAGTGGGAGCGCGTGATGAACTTCGAGGACGGCGGCGTGGTG<br>ACCGTGACCCAGGACTCCTCCCTGCAGGACGGCGAGTTCATCTAC<br>AAGGTGAAGCTGCGCGGCACCAACTTCCCCTCCGACGGCCCCGTA<br>ATGCAGAAGAAGACCATGGGCTGGGAGGCCTCCTCCGAGCGGATG<br>TACCCCGAGGACGGCGCCCTGAAGGGCGAGATCAAGCAGAGGCTG<br>AAGCTGAAGGACGGCGGCCACTACGACGCTGAGGTCAAGACCACC<br>TACAAGGCCAAGAAGCCCGTGCAGCTGCCCGGCGCCTACAACGTC<br>AACATCAAGTTGGACATCACCTCCCACAACGAGGACTACACCATC<br>GTGGAACAGTACGAACGCGCCGAGGGCCGCCACTCCACCGGCGGC<br>ATGGACGAGCTGTACAAGGGATCCGGTATGGACAAGAAGTACAGC<br>ATCGGCCTGGATATCGGCACCAACTCTGTGGGCTGGGCCGTGATC<br>ACCGACGAGTACAAGGTGCCCAGCAAGAAATTCAAGGTGCTGGGC<br>AACACCGACCGGCACAGCATCAAGAAGAACCTGATCGGCGCCCTG<br>CTGTTCGACAGCGGAGAAACAGCCGAGGCCACCCGGCTGAAGAGA<br>ACCGCCAGAAGAAGATACACCAGACGGAAGAACCGGATCTGCTAT<br>CTGCAAGAGATCTTCAGCAACGAGATGGCCAAGGTGGACGACAGC

	TTCTTCCACAGACTGGAAGAGTCCTTCCTGGTGGAAGAGGATAAG<br>AAGCACGAGCGGCACCCCATCTTCGGCAACATCGTGGACGAGGTG<br>GCCTACCACGAGAAGTACCCCACCATCTACCACCTGAGAAAGAAA<br>CTGGTGGACAGCACCGACAAGGCCGACCTGCGGCTGATCTATCTG<br>GCCCTGGCCCACATGATCAAGTTCCGGGGCCACTTCCTGATCGAGG<br>GCGACCTGAACCCCGACAACAGCGACGTGGACAAGCTGTTCATCC<br>AGCTGGTGCAGACCTACAACCAGCTGTTCGAGGAAAACCCCATCA<br>ACGCCAGCGGCGTGGACGCCAAGGCCATCCTGTCTGCCAGACTGA<br>GCAAGAGCAGACGGCTGGAAAATCTGATCGCCCAGCTGCCCGGCG<br>AGAAGAAGAATGGCCTGTTCGGCAACCTGATTGCCCTGAGCCTGG<br>GCCTGACCCCCAACTTCAAGAGCAACTTCGACCTGGCCGAGGATG<br>CCAAACTGCAGCTGAGCAAGGACACCTACGACGACGACCTGGACA<br>ACCTGCTGGCCCAGATCGGCGACCAGTACGCCGACCTGTTTCTGGC<br>CGCCAAGAACCTGTCCGACGCCATCCTGCTGAGCGACATCCTGAG<br>AGTGAACACCGAGATCACCAAGGCCCCCCTGAGCGCCTCTATGAT<br>CAAGAGATACGACGAGCACCACCAGGACCTGACCCTGCTGAAAGC<br>TCTCGTGCGGCAGCAGCTGCCTGAGAAGTACAAAGAGATTTTCTTC<br>GACCAGAGCAAGAACGGCTACGCCGGCTACATCGATGGCGGAGCC<br>AGCCAGGAAGAGTTCTACAAGTTCATCAAGCCCATCCTGGAAAAG<br>ATGGACGGCACCGAGGAACTGCTCGTGAAGCTGAACAGAGAGGA<br>CCTGCTGCGGAAGCAGCGGACCTTCGACAACGGCAGCATCCCCCA<br>CCAGATCCACCTGGGAGAGCTGCACGCCATTCTGCGGCGGCAGGA<br>AGATTTTTACCCATTCCTGAAGGACAACCGGGAAAAGATCGAGAA<br>GATCCTGACCTTCCGCATCCCCTACTACGTGGGCCCTCTGGCCAGG<br>GGAAACAGCAGATTCGCCTGGATGACCAGAAAGAGCGAGGAAAC<br>CATCACCCCCTGGAACTTCGAGGAAGTGGTGGACAAGGGCGCCAG<br>CGCCCAGAGCTTCATCGAGCGGATGACCAACTTCGATAAGAACCT<br>GCCCAACGAGAAGGTGCTGCCCAAGCACAGCCTGCTGTACGAGTA<br>CTTCACCGTGTACAACGAGCTGACCAAAGTGAAATACGTGACCGA<br>GGGAATGAGAAAGCCCGCCTTCCTGAGCGGCGAGCAGAAAAAAG<br>CCATCGTGGACCTGCTGTTCAAGACCAACCGGAAAGTGACCGTGA<br>AGCAGCTGAAAGAGGACTACTTCAAGAAAATCGAGTGCTTCGACT<br>CCGTGGAAATCTCCGGCGTGGAAGATCGGTTCAACGCCTCCCTGG<br>GCACATACCACGATCTGCTGAAAATTATCAAGGACAAGGACTTCC<br>TGGACAATGAGGAAAACGAGGACATTCTGGAAGATATCGTGCTGA<br>CCCTGACACTGTTTGAGGACAGAGAGATGATCGAGGAACGGCTGA<br>AAACCTATGCCCACCTGTTCGACGACAAAGTGATGAAGCAGCTGA<br>AGCGGCGGAGATACACCGGCTGGGGCAGGCTGAGCCGGAAGCTG<br>ATCAACGGCATCCGGGACAAGCAGTCCGGCAAGACAATCCTGGAT<br>TTCCTGAAGTCCGACGGCTTCGCCAACAGAAACTTCATGCAGCTGA<br>TCCACGACGACAGCCTGACCTTTAAAGAGGACATCCAGAAAGCCC<br>AGGTGTCCGGCCAGGGCGATAGCCTGCACGAGCACATTGCCAATC<br>TGGCCGGCAGCCCCGCCATTAAGAAGGGCATCCTGCAGACAGTGA<br>AGGTGGTGGACGAGCTCGTGAAAGTGATGGGCCGGCACAAGCCCG<br>AGAACATCGTGATCGAAATGGCCAGAGAGAACCAGACCACCCAG<br>AAGGGACAGAAGAACAGCCGCGAGAGAATGAAGCGGATCGAAGA

	GGGCATCAAAGAGCTGGGCAGCCAGATCCTGAAAGAACACCCCGT<br>GGAAAACACCCAGCTGCAGAACGAGAAGCTGTACCTGTACTACCT<br>GCAGAATGGGCGGGATATGTACGTGGACCAGGAACTGGACATCAA<br>CCGGCTGTCCGACTACGATGTGGACGCTATCGTGCCTCAGAGCTTT<br>CTGGCAGACGACTCCATCGATAACAAAGTGCTGACTCGGAGCGAC<br>AAGAACCGGGGCAAGAGCGACAACGTGCCCTCCGAAGAGGTCGT<br>GAAGAAGATGAAGAACTACTGGCGCCAGCTGCTGAATGCCAAGCT<br>GATTACCCAGAGGAAGTTCGACAATCTGACCAAGGCCGAGAGAGG<br>CGGCCTGAGCGAACTGGATAAGGCCGGCTTCATCAAGAGACAGCT<br>GGTGGAAACCCGGCAGATCACAAAGCACGTGGCACAGATCCTGGA<br>CTCCCGGATGAACACTAAGTACGACGAGAACGACAAACTGATCCG<br>GGAAGTGAAAGTGATCACCCTGAAGTCCAAGCTGGTGTCCGATTT<br>CCGGAAGGATTTCCAGTTTTACAAAGTGCGCGAGATCAACAACTA<br>CCACCACGCCCACGACGCCTACCTGAACGCCGTCGTGGGAACCGC<br>CCTGATCAAAAAGTACCCTGCGCTGGAAAGCGAGTTCGTGTACGG<br>CGACTACAAGGTGTACGACGTGCGGAAGATGATCGCCAAGAGCGA<br>GCAGGAAATCGGCAAGGCTACCGCCAAGTACTTCTTCTACAGCAA<br>CATCATGAACTTTTTCAAGACCGAGATTACCCTGGCCAACGGCGA<br>GATCCGGAAGGCGCCTCTGATCGAGACAAACGGCGAAACAGGCG<br>AGATCGTGTGGGATAAGGGCCGGGACTTTGCCACCGTGCGGAAAG<br>TGCTGTCTATGCCCCAAGTGAATATCGTGAAAAAGACCGAGGTGC<br>AGACAGGCGGCTTCAGCAAAGAGTCTATCCTGCCCAAGAGGAACA<br>GCGACAAGCTGATCGCCAGAAAGAAGGACTGGGACCCTAAGAAG<br>TACGGCGGCTTCGACAGCCCCACCGTGGCCTATTCTGTGCTGGTGG<br>TGGCCAAAGTGGAAAAGGGCAAGTCCAAGAAACTGAAGAGTGTG<br>AAAGAGCTGCTGGGGATCACCATCATGGAAAGAAGCAGCTTCGAG<br>AAGAATCCCATCGACTTTCTGGAAGCCAAGGGCTACAAAGAAGTG<br>AAAAAGGACCTGATCATCAAGCTGCCTAAGTACTCCCTGTTCGAG<br>CTGGAAAACGGCCGGAAGAGAATGCTGGCCTCTGCCGGCGAACTG<br>CAGAAGGGAAACGAACTGGCCCTGCCCTCCAAATATGTGAACTTC<br>CTGTACCTGGCCAGCCACTATGAGAAGCTGAAGGGCTCCCCCGAG<br>GATAATGAGCAGAAACAGCTGTTTGTGGAACAGCACAAACACTAC<br>CTGGACGAGATCATCGAGCAGATCAGCGAGTTCTCCAAGAGAGTG<br>ATCCTGGCCGACGCTAATCTGGACAAGGTGCTGAGCGCCTACAAC<br>AAGCACAGAGACAAGCCTATCAGAGAGCAGGCCGAGAATATCATC<br>CACCTGTTTACCCTGACCAATCTGGGAGCCCCTGCCGCCTTCAAGT<br>ACTTTGACACCACCATCGACCGGAAGAGGTACACCAGCACCAAAG<br>AGGTGCTGGACGCCACCCTGATCCACCAGAGCATCACCGGCCTGT<br>ACGAGACACGGATCGACCTGTCTCAGCTGGGAGGCGACGCCTATC<br>CCTATGACGTGCCCGATTATGCCAGCCTGGGCAGCGGCTCCCCCAA<br>GAAAAAACGCAAGGTGGAAGATCCTAAGAAAAAGCGGAAAGGTT<br>CTAGTGAAACCCCGGGAACAAGTGAGTCGGCCACCCCTGAAGGTG<br>GATCAGGGGGTAGCGGATCCGTTCAGATCCCGCAGAACCCGCTGA<br>TTCTGGTTGACGGATCTAGTTACCTGTACCGTGCTTACCATGCTTTC<br>CCGCCTTTGACCAATTCTGCTGGTGAACCTACGGGAGCTATGTACG<br>GAGTTCTGAATATGTTGCGTTCTTTAATTATGCAGTACAAGCCTAC

	CCACGCTGCTGTTGTTTTCGATGCTAAAGGTAAGACGTTCCGCGAC<br>GAGTTATTCGAGCACTATAAGTCTCACCGTCCTCCGATGCCTGATG<br>ACTTACGCGCTCAGATTGAGCCGCTGCATGCTATGGTGAAGGCTAT<br>GGGTTTACCTCTTTTGGCTGTCAGCGGTGTTGAGGCTGATGATGTC<br>ATTGGCACCTTAGCTCGTGAGGCTGAGAAGGCTGGTCGCCCTGTTT<br>TGATTTCTACCGGTGACAAGGACATGGCTCAATTGGTTACCCCGAA<br>CATCACCCTGATCAACACCATGACCAACACGATTCTGGGTCCTGAG<br>GAAGTTGTTAACAAATATGGTGTTCCTCCGGAGTTGATTATTGACT<br>TTCTTGCTCTTATGGGCGATTCTTCAGACAATATCCCGGGTGTTCC<br>AGGTGTTGGAGAGAAGACTGCTCAAGCTCTGCTTCAGGGTCTGGG<br>TGGTTTGGACACCCTTTACGCTGAACCGGAGAAGATCGCCGGTCTG<br>TCTTTTCGCGGTGCTAAGACCATGGCTGCTAAACTGGAACAGAATA<br>AGGAGGTCGCATACCTGTCTTATCAATTGGCTACCATCAAGACGG<br>ATGTGGAGTTAGAACTTACGTGCGAGCAGCTTGAGGTTCAACAGC<br>CTGCTGCTGAGGAACTGCTGGGTCTTTTTAAGAAATACGAATTTAA<br>GCGTTGGACCGCCGACGTTGAGGCTGGTAAGTGGCTGCAAGCTAA<br>GGGTGCTAAGCCGGCTGCTAAACCGCAAGAAACGAGTGTCGCTGA<br>TGAGGCTCCGGAGGTTACCGCTACCGTTATCTCTTACGATAATTAT<br>GTTACGATTCTGGACGAGGAAACCTTAAAGGCTTGGATCGCTAAA<br>TTAGAGAAGGCTCCTGTTTTCGCTTTCGACACGGAAACGGATTCTC<br>TGGACAATATTAGTGCGAATCTTGTTGGTCTGAGTTTCGCAATTGA<br>ACCGGGTGTTGCTGCTTACATCCCTGTGGCACACGACTACCTGGAC<br>GCTCCGGACCAGATTTCACGTGAACGCGCTCTGGAACTGCTGAAG<br>CCTTTATTAGAGGACGAGAAAGCTTTGAAAGTTGGTCAGAATTTG<br>AAGTATGCTCGTGGAATCTTAGCTAATTATGGTATCGAGTTGCGCG<br>GTATCGCTTTCGACACGATGTTGGAATCTTATATCCTGAACTCTGT<br>CGCTGGTCGCCATGACATGGACTCTCTGGCTGAGCGCTGGCTGAA<br>ACATAAGACGATTACCTTCGAGGAAATCGCAGGAAAGGGTAAGAA<br>CCAGCTCACGTTCAATCAAATCGCTCTGGAGGAAGCTGGTCGCTAT<br>GCTGCTGAGGACGCTGACGTTACTCTGCAACTGCACTTGAAGATGT<br>GGCCTGACTTGCAGAAGCATAAGGGTCCACTGAATGTTTTTGAAA<br>ACATTGAGATGCCTTTGGTTCCAGTTCTGTCTCGTATCGAGCGCAA<br>TGGCGTTAAAATTGACCCAAAGGTTTTACATAACCACTCAGAGGA<br>ACTGACGCTGCGCTTAGCCGAATTGGAGAAAAAGGCTCACGAGAT<br>CGCTGGCGAAGAGTTCAATCTGTCATCTACGAAACAACTGCAGAC<br>TATCCTGTTCGAGAAGCAAGGTATCAAGCCATTAAAAAAGACCCC<br>TGGCGGTGCTCCGTCTACCTCTGAGGAAGTTTTGGAGGAGTTAGCT<br>TTGGATTACCCTCTGCCGAAGGTTATCTTGGAATACCGCGGTTTGG<br>CTAAATTGAAGTCTACTTATACGGATAAACTTCCTTTGATGATTAA<br>TCCAAAGACGGGTCGCGTTCACACGTCGTACCATCAAGCTGTTACC<br>GCTACCGGTCGCCTGTCTTCTACGGATCCGAATTTACAGAATATTC<br>CTGTGCGCAATGAGGAGGGCCGCCGCATTCGTCAAGCTTTTATCGC<br>TCCGGAAGACTACGTTATCGTTTCTGCTGATTATTCTCAAAATGAA<br>TTACGTATCATGGCTCACCTGTCTCGCGATAAGGGTCTGTTGACGG<br>CCTTTGCTGAGGGTAAGGACATTCATCGTGCTACCGCTGCTGAGGT<br>TTACGGCCTGCCGTTGGAAACGGTTACGTCTGAACAGCGTCGCTCT

	GCTAAGCGTATTAATTTCGGCTTAATCTACGGTATGTCTGCGTTTG<br>GCTTAGCTCGTCAGCTGAATATCCCGCGCAAGGAAGCTCAAAAT<br>ATATGGATCTGTATTTTGAGCGTTACCACGGTGTTTTGGAATACAT<br>GGAGCGTACGCGCGCGCAAGCTAAGGAACAAGGTTATGTGGAAAC<br>CTTGGATGGTCGTCGCTTGTACTTGCCTGACATTAAGTCTTCTAAC<br>GGCGCCCGCCGCGCTGCTGCCGAGCGCGCTGCTATCAATGCTCCG<br>ATGCAAGGTACTGCTGCTGATATTATTAAGCGTGCTATGATCGCTG<br>TGGACGCTTGGCTGCAAGCTGAACAGCCTCGCGTTCGCATGATTAT<br>GCAAGTTCATGACGAGTTGGTTTTCGAGGTGCATAAGGACGACGT<br>GGACGCTGTTGCTAAACAAATCCACCAGTTGATGGAGAATTGCAC<br>GCGCTTAGACGTTCCGCTGCTGGTTGAAGTTGGTTCTGGTGAAAAC<br>TGGGACCAGGCTCACTAATAATGGCTGAAAACTGGGACCAGGCTC<br>ACTAACTCAGATCCTACTAGGTTTAATAAACATCTTTATTTTCATTA<br>CATCTGTGTGTTGGTTTTTTGTGTGGTACCCAATTCGCCCTATAGTG<br>AGTCGTATTACTCACTGGCCGTCGTTTTACAACGTCGTGACTGGGA<br>AAACCCTGGCGTTACCCAACTTAATCGCCTTGCAGCACATCCCCCT<br>TTCGCCAGCTGGCGTAATAGCGAAGAGGCCCGCACCGATCGCCCT<br>TCCCAACAGTTGCGCAGCCTGAATGGCGAATGGACGCGCCCTGT<br>AGCGGCGCATTAAGCGCGGCGGGTGTGGTGGTTACGCGCAGCGTG<br>ACCGCTACACTTGCCAGCGCCCTAGCGCCCGCTCCTTTCGCTTTCTT<br>CCCTTCCTTTCTCGCCACGTTCGCCGGCTTTCCCCGTCAAGCTCTAA<br>ATCGGGGGCTCCCTTTAGGGTTCCGATTTAGTGCTTTACGGCACCT<br>CGACCCCAAAAAACTTGATTAGGGTGATGGTTCACGTAGTGGGCC<br>ATCGCCCTGATAGACGGTTTTTCGCCCTTTGACGTTGGAGTCCACG<br>TTCTTTAATAGTGGACTCTTGTTCCAAACTGGAACAACACTCAACC<br>CTATCTCGGTCTATTCTTTTGATTTATAAGGGATTTTGCCGATTTCG<br>GCCTATTGGTTAAAAAATGAGCTGATTTAACAAAAATTTAACGCG<br>AATTTTAACAAAATATTAACGCTTACAATTTAGGTGGCACTTTTCG<br>GGGAAATGTG<br><br>**Ampicillin resistance cassette-ColE1 origin of replication-hU6 promoter-gRNA sequence-SpCas9 gRNA scaffold-CMV promoter-NLS-mCherry-GSGM-enCas9(H840A)-NLS-linker-PolI5M-stop-SV40 polyA**
p.dCas9-PolI5M	CGCGGAACCCCTATTTGTTTATTTTTCTAAATACATTCAAATATGTA<br>TCCGCTCATGAGACAATAACCCTGATAAATGCTTCAATAATATTGA<br>AAAAGGAAGAGTATGAGTATTCAACATTTCCGTGTCGCCCTTATTC<br>CCTTTTTTGCGGCATTTTGCCTTCCTGTTTTTGCTCACCCAGAAACG<br>CTGGTGAAAGTAAAAGATGCTGAAGATCAGTTGGGTGCACGAGTG<br>GGTTACATCGAACTGGATCTCAACAGCGGTAAGATCCTTGAGAGT<br>TTTCGCCCCGAAGAACGTTTTCCAATGATGAGCACTTTTAAAGTTC<br>TGCTATGTGGCGCGGTATTATCCCGTATTGACGCCGGGCAAGAGC<br>AACTCGGTCGCCGCATACACTATTCTCAGAATGACTTGGTTGAGTA<br>CTCACCAGTCACAGAAAAGCATCTTACGGATGGCATGACAGTAAG<br>AGAATTATGCAGTGCTGCCATAACCATGAGTGATAACACTGCGGC

	CAACTTACTTCTGACAACGATCGGAGGACCGAAGGAGCTAACCGC<br>TTTTTTGCACAACATGGGGGATCATGTAACTCGCCTTGATCGTTGG<br>GAACCGGAGCTGAATGAAGCCATACCAAACGACGAGCGTGACACC<br>ACGATGCCTGTAGCAATGGCAACAACGTTGCGCAAACTATTAACT<br>GGCGAACTACTTACTCTAGCTTCCCGGCAACAATTAATAGACTGGA<br>TGGAGGCGGATAAAGTTGCAGGACCACTTCTGCGCTCGGCCCTTCC<br>GGCTGGCTGGTTTATTGCTGATAAATCTGGAGCCGGTGAGCGTGGT<br>TCTCGCGGTATCATTGCAGCACTGGGGCCAGATGGTAAGCCCTCCC<br>GTATCGTAGTTATCTACACGACGGGGAGTCAGGCAACTATGGATG<br>AACGAAATAGACAGATCGCTGAGATAGGTGCCTCACTGATTAAGC<br>ATTGGTAACTGTCAGACCAAGTTTACTCATATATACTTTAGATTGA<br>TTTAAAACTTCATTTTTAATTTAAAAGGATCTAGGTGAAGATCCTT<br>TTTGATAATCTCATGACCAAAATCCCTTAACGTGAGTTTTCGTTCC<br>ACTGAGCGTCAGACCCCGTAGAAAAGATCAAAGGATCTTCTTGAG<br>ATCCTTTTTTTCTGCGCGTAATCTGCTGCTTGCAAACAAAAAAACC<br>ACCGCTACCAGCGGTGGTTTGTTTGCCGGATCAAGAGCTACCAACT<br>CTTTTTCCGAAGGTAACTGGCTTCAGCAGAGCGCAGATACCAAAT<br>ACTGTCCTTCTAGTGTAGCCGTAGTTAGGCCACCACTTCAAGAACT<br>CTGTAGCACCGCCTACATACCTCGCTCTGCTAATCCTGTTACCAGT<br>GGCTGCTGCCAGTGGCGATAAGTCGTGTCTTACCGGGTTGGACTCA<br>AGACGATAGTTACCGGATAAGGCGCAGCGGTCGGGCTGAACGGGG<br>GGTTCGTGCACACAGCCCAGCTTGGAGCGAACGACCTACACCGAA<br>CTGAGATACCTACAGCGTGAGCTATGAGAAAGCGCCACGCTTCCC<br>GAAGGGAGAAAGGCGGACAGGTATCCGGTAAGCGGCAGGGTCGG<br>AACAGGAGAGCGCACGAGGGAGCTTCCAGGGGGAAACGCCTGGT<br>ATCTTTATAGTCCTGTCGGGTTTCGCCACCTCTGACTTGAGCGTCG<br>ATTTTTGTGATGCTCGTCAGGGGGGCGGAGCCTATGGAAAAACGC<br>CAGCAACGCGGCCTTTTTACGGTTCCTGGCCTTTTGCTGGCCTTTTG<br>CTCACATGTTCTTTCCTGCGTTATCCCCTGATTCTGTGGATAACCGT<br>ATTACCGCCTTTGAGTGAGCTGATACCGCTCGCCGCAGCCGAACG<br>ACCGAGCGCAGCGAGTCAGTGAGCGAGGAAGCGGAAGAGCGCCC<br>AATACGCAAACCGCCTCTCCCCGCGCGTTGGCCGATTCATTAATGC<br>AGCTGGCACGACAGGTTTCCCGACTGGAAAGCGGGCAGTGAGCGC<br>AACGCAATTAATGTGAGTTAGCTCACTCATTAGGCACCCCAGGCTT<br>TACACTTTATGCTTCCGGCTCGTATGTTGTGTGGAATTGTGAGCGG<br>ATAACAATTTCACACAGGAAACAGCTATGACCATGATTACGCCAA<br>GCGCGCAATTAACCCTCACTAAAGGGAACAAAAGCTGGAGCTCCA<br>CCGCGGTGGCGGCCGCTCTTAAGGGGTGCAGCGGCCTCCGCGCCG<br>GGTTTTGGCGCCTCGATCCAAGGTCGGGCAGGAAGAGGGCCTATT<br>TCCCATGATTCCTTCATATTTGCATATACGATACAAGGCTGTTAGA<br>GAGATAATTAGAATTAATTTGACTGTAAACACAAAGATATTAGTA<br>CAAAATACGTGACGTAGAAAGTAATAATTTCTTGGGTAGTTTGCA<br>GTTTTAAAATTATGTTTTAAAATGGACTATCATATGCTTACCGTAA<br>CTTGAAAGTATTTCGATTTCTTGGCTTTATATATCTTGTGGAAAGG<br>ACGAAACACC-[gRNA SEQUENCE]-<br>GTTTTAGAGCTAGAAATAGCAAGTTAAAATAAGGCTAGTCCGTTA

	TCAACTTGAAAAAGTGGCACCGAGTCGGTGCTTTTTTCCGCGGCCT<br>CTAGACTCGAGGCGTTGACATTGATTATTGACTAGTTATTAATAGT<br>AATCAATTACGGGGTCATTAGTTCATAGCCCATATATGGAGTTCCG<br>CGTTACATAACTTACGGTAAATGGCCCGCCTGGCTGACCGCCCAAC<br>GACCCCCGCCCATTGACGTCAATAATGACGTATGTTCCCATAGTAA<br>CGCCAATAGGGACTTTCCATTGACGTCAATGGGTGGAGTATTTACG<br>GTAAACTGCCCACTTGGCAGTACATCAAGTGTATCATATGCCAAGT<br>ACGCCCCCTATTGACGTCAATGACGGTAAATGGCCCGCCTGGCATT<br>ATGCCCAGTACATGACCTTATGGGACTTTCCTACTTGGCAGTACAT<br>CTACGTATTAGTCATCGCTATTACCATGGTGATGCGGTTTTGGCAG<br>TACATCAATGGGCGTGGATAGCGGTTTGACTCACGGGGATTTCCA<br>AGTCTCCACCCCATTGACGTCAATGGGAGTTTGTTTTGGCACCAAA<br>ATCAACGGGACTTTCCAAAATGTCGTAACAACTCCGCCCCATTGAC<br>GCAAATGGGCGGTAGGCGTGTACGGTGGGAGGTCTATATAAGCAG<br>AGCTCTCTGGCTAACTACCGGTGCCACCATGGCCCCAAAGAAGAA<br>GCGGAAGGTCGGTATCCACGGAGTCCCAGCAGCCGTGAGCAAGGG<br>CGAGGAGGATAACATGGCCATCATCAAGGAGTTCATGCGCTTCAA<br>GGTGCACATGGAGGGCTCCGTGAACGGCCACGAGTTCGAGATCGA<br>GGGCGAGGGCGAGGGCCGCCCCTACGAGGGCACCCAGACCGCCA<br>AGCTGAAGGTGACCAAGGGTGGCCCCCTGCCCTTCGCCTGGGACA<br>TCCTGTCCCCTCAGTTCATGTACGGCTCCAAGGCCTACGTGAAGCA<br>CCCCGCCGACATCCCCGACTACTTGAAGCTGTCCTTCCCCGAGGGC<br>TTCAAGTGGGAGCGCGTGATGAACTTCGAGGACGGCGGCGTGGTG<br>ACCGTGACCCAGGACTCCTCCCTGCAGGACGGCGAGTTCATCTAC<br>AAGGTGAAGCTGCGCGGCACCAACTTCCCCTCCGACGGCCCCGTA<br>ATGCAGAAGAAGACCATGGGCTGGGAGGCCTCCTCCGAGCGGATG<br>TACCCCGAGGACGGCGCCCTGAAGGGCGAGATCAAGCAGAGGCTG<br>AAGCTGAAGGACGGCGGCCACTACGACGCTGAGGTCAAGACCACC<br>TACAAGGCCAAGAAGCCCGTGCAGCTGCCCGGCGCCTACAACGTC<br>AACATCAAGTTGGACATCACCTCCCACAACGAGGACTACACCATC<br>GTGGAACAGTACGAACGCGCCGAGGGCCGCCACTCCACCGGCGGC<br>ATGGACGAGCTGTACAAGGGATCCGGTATGGACAAGAAGTACAGC<br>ATCGGCCTGGCCATCGGCACCAACTCTGTGGGCTGGGCCGTGATC<br>ACCGACGAGTACAAGGTGCCCAGCAAGAAATTCAAGGTGCTGGGC<br>AACACCGACCGGCACAGCATCAAGAAGAACCTGATCGGCGCCCTG<br>CTGTTCGACAGCGGAGAAACAGCCGAGGCCACCCGGCTGAAGAGA<br>ACCGCCAGAAGAAGATACACCAGACGGAAGAACCGGATCTGCTAT<br>CTGCAAGAGATCTTCAGCAACGAGATGGCCAAGGTGGACGACAGC<br>TTCTTCCACAGACTGGAAGAGTCCTTCCTGGTGGAAGAGGATAAG<br>AAGCACGAGCGGCACCCCATCTTCGGCAACATCGTGGACGAGGTG<br>GCCTACCACGAGAAGTACCCCACCATCTACCACCTGAGAAAGAAA<br>CTGGTGGACAGCACCGACAAGGCCGACCTGCGGCTGATCTATCTG<br>GCCCTGGCCCACATGATCAAGTTCCGGGGCCACTTCCTGATCGAGG<br>GCGACCTGAACCCCGACAACAGCGACGTGGACAAGCTGTTCATCC<br>AGCTGGTGCAGACCTACAACCAGCTGTTCGAGGAAAACCCCATCA<br>ACGCCAGCGGCGTGGACGCCAAGGCCATCCTGTCTGCCAGACTGA

	GCAAGAGCAGACGGCTGGAAAATCTGATCGCCCAGCTGCCCGGCG<br>AGAAGAAGAATGGCCTGTTCGGCAACCTGATTGCCCTGAGCCTGG<br>GCCTGACCCCCAACTTCAAGAGCAACTTCGACCTGGCCGAGGATG<br>CCAAACTGCAGCTGAGCAAGGACACCTACGACGACGACCTGGACA<br>ACCTGCTGGCCCAGATCGGCGACCAGTACGCCGACCTGTTTCTGGC<br>CGCCAAGAACCTGTCCGACGCCATCCTGCTGAGCGACATCCTGAG<br>AGTGAACACCGAGATCACCAAGGCCCCCCTGAGCGCCTCTATGAT<br>CAAGAGATACGACGAGCACCACCAGGACCTGACCCTGCTGAAAGC<br>TCTCGTGCGGCAGCAGCTGCCTGAGAAGTACAAAGAGATTTTCTTC<br>GACCAGAGCAAGAACGGCTACGCCGGCTACATCGATGGCGGAGCC<br>AGCCAGGAAGAGTTCTACAAGTTCATCAAGCCCATCCTGGAAAAG<br>ATGGACGGCACCGAGGAACTGCTCGTGAAGCTGAACAGAGAGGA<br>CCTGCTGCGGAAGCAGCGGACCTTCGACAACGGCAGCATCCCCCA<br>CCAGATCCACCTGGGAGAGCTGCACGCCATTCTGCGGCGGCAGGA<br>AGATTTTTACCCATTCCTGAAGGACAACCGGGAAAAGATCGAGAA<br>GATCCTGACCTTCCGCATCCCCTACTACGTGGGCCCTCTGGCCAGG<br>GGAAACAGCAGATTCGCCTGGATGACCAGAAAGAGCGAGGAAAC<br>CATCACCCCCTGGAACTTCGAGGAAGTGGTGGACAAGGGCGCCAG<br>CGCCCAGAGCTTCATCGAGCGGATGACCAACTTCGATAAGAACCT<br>GCCCAACGAGAAGGTGCTGCCCAAGCACAGCCTGCTGTACGAGTA<br>CTTCACCGTGTACAACGAGCTGACCAAAGTGAAATACGTGACCGA<br>GGGAATGAGAAAGCCCGCCTTCCTGAGCGGCGAGCAGAAAAAAG<br>CCATCGTGGACCTGCTGTTCAAGACCAACCGGAAAGTGACCGTGA<br>AGCAGCTGAAAGAGGACTACTTCAAGAAAATCGAGTGCTTCGACT<br>CCGTGGAAATCTCCGGCGTGGAAGATCGGTTCAACGCCTCCCTGG<br>GCACATACCACGATCTGCTGAAAATTATCAAGGACAAGGACTTCC<br>TGGACAATGAGGAAAACGAGGACATTCTGGAAGATATCGTGCTGA<br>CCCTGACACTGTTTGAGGACAGAGAGATGATCGAGGAACGGCTGA<br>AAACCTATGCCCACCTGTTCGACGACAAAGTGATGAAGCAGCTGA<br>AGCGGCGGAGATACACCGGCTGGGGCAGGCTGAGCCGGAAGCTG<br>ATCAACGGCATCCGGGACAAGCAGTCCGGCAAGACAATCCTGGAT<br>TTCCTGAAGTCCGACGGCTTCGCCAACAGAAACTTCATGCAGCTGA<br>TCCACGACGACAGCCTGACCTTTAAAGAGGACATCCAGAAAGCCC<br>AGGTGTCCGGCCAGGGCGATAGCCTGCACGAGCACATTGCCAATC<br>TGGCCGGCAGCCCCGCCATTAAGAAGGGCATCCTGCAGACAGTGA<br>AGGTGGTGGACGAGCTCGTGAAAGTGATGGGCCGGCACAAGCCCG<br>AGAACATCGTGATCGAAATGGCCAGAGAGAACCAGACCACCCAG<br>AAGGGACAGAAGAACAGCCGCGAGAGAATGAAGCGGATCGAAGA<br>GGGCATCAAAGAGCTGGGCAGCCAGATCCTGAAAGAACACCCCGT<br>GGAAAACACCCAGCTGCAGAACGAGAAGCTGTACCTGTACTACCT<br>GCAGAATGGGCGGGATATGTACGTGGACCAGGAACTGGACATCAA<br>CCGGCTGTCCGACTACGATGTGGACGCTATCGTGCCTCAGAGCTTT<br>CTGAAGGACGACTCCATCGATAACAAAGTGCTGACTCGGAGCGAC<br>AAGAACCGGGGCAAGAGCGACAACGTGCCCTCCGAAGAGGTCGT<br>GAAGAAGATGAAGAACTACTGGCGCCAGCTGCTGAATGCCAAGCT<br>GATTACCCAGAGGAAGTTCGACAATCTGACCAAGGCCGAGAGAGG

		CGGCCTGAGCGAACTGGATAAGGCCGGCTTCATCAAGAGACAGCT<br>GGTGGAAACCCGGCAGATCACAAAGCACGTGGCACAGATCCTGGA<br>CTCCCGGATGAACACTAAGTACGACGAGAACGACAAACTGATCCG<br>GGAAGTGAAAGTGATCACCCTGAAGTCCAAGCTGGTGTCCGATTT<br>CCGGAAGGATTTCCAGTTTTACAAAGTGCGCGAGATCAACAACTA<br>CCACCACGCCCACGACGCCTACCTGAACGCCGTCGTGGGAACCGC<br>CCTGATCAAAAAGTACCCTAAGCTGGAAAGCGAGTTCGTGTACGG<br>CGACTACAAGGTGTACGACGTGCGGAAGATGATCGCCAAGAGCGA<br>GCAGGAAATCGGCAAGGCTACCGCCAAGTACTTCTTCTACAGCAA<br>CATCATGAACTTTTTCAAGACCGAGATTACCCTGGCCAACGGCGA<br>GATCCGGAAGCGGCCTCTGATCGAGACAAACGGCGAAACAGGCG<br>AGATCGTGTGGGATAAGGGCCGGGACTTTGCCACCGTGCGGAAAG<br>TGCTGTCTATGCCCCAAGTGAATATCGTGAAAAAGACCGAGGTGC<br>AGACAGGCGGCTTCAGCAAAGAGTCTATCCTGCCCAAGAGGAACA<br>GCGACAAGCTGATCGCCAGAAAGAAGGACTGGGACCCTAAGAAG<br>TACGGCGGCTTCGACAGCCCCACCGTGGCCTATTCTGTGCTGGTGG<br>TGGCCAAAGTGGAAAAGGGCAAGTCCAAGAAACTGAAGAGTGTG<br>AAAGAGCTGCTGGGGATCACCATCATGGAAAGAAGCAGCTTCGAG<br>AAGAATCCCATCGACTTTCTGGAAGCCAAGGGCTACAAAGAAGTG<br>AAAAAGGACCTGATCATCAAGCTGCCTAAGTACTCCCTGTTCGAG<br>CTGGAAAACGGCCGGAAGAGAATGCTGGCCTCTGCCGGCGAACTG<br>CAGAAGGGAAACGAACTGGCCCTGCCCTCCAAATATGTGAACTTC<br>CTGTACCTGGCCAGCCACTATGAGAAGCTGAAGGGCTCCCCCGAG<br>GATAATGAGCAGAAACAGCTGTTTGTGGAACAGCACAAACACTAC<br>CTGGACGAGATCATCGAGCAGATCAGCGAGTTCTCCAAGAGAGTG<br>ATCCTGGCCGACGCTAATCTGGACAAGGTGCTGAGCGCCTACAAC<br>AAGCACAGAGACAAGCCTATCAGAGAGCAGGCCGAGAATATCATC<br>CACCTGTTTACCCTGACCAATCTGGGAGCCCCTGCCGCCTTCAAGT<br>ACTTTGACACCACCATCGACCGGAAGAGGTACACCAGCACCAAAG<br>AGGTGCTGGACGCCACCCTGATCCACCAGAGCATCACCGGCCTGT<br>ACGAGACACGGATCGACCTGTCTCAGCTGGGAGGCGACGCCTATC<br>CCTATGACGTGCCCGATTATGCCAGCCTGGGCAGCGGCTCCCCCAA<br>GAAAAAACGCAAGGTGGAAGATCCTAAGAAAAAGCGGAAAGGTT<br>CTAGTGAAACCCCGGGAACAAGTGAGTCGGCCACCCCTGAAGGTG<br>GATCAGGGGGTAGCGGATCCGTTCAGATCCCGCAGAACCCGCTGA<br>TTCTGGTTGACGGATCTAGTTACCTGTACCGTGCTTACCATGCTTTC<br>CCGCCTTTGACCAATTCTGCTGGTGAACCTACGGGAGCTATGTACG<br>GAGTTCTGAATATGTTGCGTTCTTTAATTATGCAGTACAAGCCTAC<br>CCACGCTGCTGTTGTTTTCGATGCTAAAGGTAAGACGTTCCGCGAC<br>GAGTTATTCGAGCACTATAAGTCTCACCGTCCTCCGATGCCTGATG<br>ACTTACGCGCTCAGATTGAGCCGCTGCATGCTATGGTGAAGGCTAT<br>GGGTTTACCTCTTTTGGCTGTCAGCGGTGTTGAGGCTGATGATGTC<br>ATTGGCACCTTAGCTCGTGAGGCTGAGAAGGCTGGTCGCCCTGTTT<br>TGATTTCTACCGGTGACAAGGACATGGCTCAATTGGTTACCCCGAA<br>CATCACCCTGATCAACACCATGACCAACACGATTCTGGGTCCTGAG<br>GAAGTTGTTAACAAATATGGTGTTCCTCCGGAGTTGATTATTGACT

	TTCTTGCTCTTATGGGCGATTCTTCAGACAATATCCCGGGTGTTCC<br>AGGTGTTGGAGAGAAGACTGCTCAAGCTCTGCTTCAGGGTCTGGG<br>TGGTTTGGACACCCTTTACGCTGAACCGGAGAAGATCGCCGGTCTG<br>TCTTTTCGCGGTGCTAAGACCATGGCTGCTAAACTGGAACAGAATA<br>AGGAGGTCGCATACCTGTCTTATCAATTGGCTACCATCAAGACGG<br>ATGTGGAGTTAGAACTTACGTGCGAGCAGCTTGAGGTTCAACAGC<br>CTGCTGCTGAGGAACTGCTGGGTCTTTTTAAGAAATACGAATTTAA<br>GCGTTGGACCGCCGACGTTGAGGCTGGTAAGTGGCTGCAAGCTAA<br>GGGTGCTAAGCCGGCTGCTAAACCGCAAGAAACGAGTGTCGCTGA<br>TGAGGCTCCGGAGGTTACCGCTACCGTTATCTCTTACGATAATTAT<br>GTTACGATTCTGGACGAGGAAACCTTAAAGGCTTGGATCGCTAAA<br>TTAGAGAAGGCTCCTGTTTTCGCTTTCGACACGGAAACGGATTCTC<br>TGGACAATATTAGTGCGAATCTTGTTGGTCTGAGTTTCGCAATTGA<br>ACCGGGTGTTGCTGCTTACATCCCTGTGGCACACGACTACCTGGAC<br>GCTCCGGACCAGATTTCACGTGAACGCGCTCTGGAACTGCTGAAG<br>CCTTTATTAGAGGACGAGAAAGCTTTGAAAGTTGGTCAGAATTTG<br>AAGTATGCTCGTGGAATCTTAGCTAATTATGGTATCGAGTTGCGCG<br>GTATCGCTTTCGACACGATGTTGGAATCTTATATCCTGAACTCTGT<br>CGCTGGTCGCCATGACATGGACTCTCTGGCTGAGCGCTGGCTGAA<br>ACATAAGACGATTACCTTCGAGGAAATCGCAGGAAAGGGTAAGAA<br>CCAGCTCACGTTCAATCAAATCGCTCTGGAGGAAGCTGGTCGCTAT<br>GCTGCTGAGGACGCTGACGTTACTCTGCAACTGCACTTGAAGATGT<br>GGCCTGACTTGCAGAAGCATAAGGGTCCACTGAATGTTTTTGAAA<br>ACATTGAGATGCCTTTGGTTCCAGTTCTGTCTCGTATCGAGCGCAA<br>TGGCGTTAAAATTGACCCAAAGGTTTTACATAACCACTCAGAGGA<br>ACTGACGCTGCGCTTAGCCGAATTGGAGAAAAAGGCTCACGAGAT<br>CGCTGGCGAAGAGTTCAATCTGTCATCTACGAAACAACTGCAGAC<br>TATCCTGTTCGAGAAGCAAGGTATCAAGCCATTAAAAAAGACCCC<br>TGGCGGTGCTCCGTCTACCTCTGAGGAAGTTTTGGAGGAGTTAGCT<br>TTGGATTACCCTCTGCCGAAGGTTATCTTGGAATACCGCGGTTTGG<br>CTAAATTGAAGTCTACTTATACGGATAAACTTCCTTTGATGATTAA<br>TCCAAAGACGGGTCGCGTTCACACGTCGTACCATCAAGCTGTTACC<br>GCTACCGGTCGCCTGTCTTCTACGGATCCGAATTTACAGAATATTC<br>CTGTGCGCAATGAGGAGGGCCGCCGCATTCGTCAAGCTTTTATCGC<br>TCCGGAAGACTACGTTATCGTTTCTGCTGATTATTCTCAAAATGAA<br>TTACGTATCATGGCTCACCTGTCTCGCGATAAGGGTCTGTTGACGG<br>CCTTTGCTGAGGGTAAGGACATTCATCGTGCTACCGCTGCTGAGGT<br>TTACGGCCTGCCGTTGGAAACGGTTACGTCTGAACAGCGTCGCTCT<br>GCTAAGCGTATTAATTTCGGCTTAATCTACGGTATGTCTGCGTTTG<br>GCTTAGCTCGTCAGCTGAATATCCCGCGCAAGGAAGCTCAAAAAT<br>ATATGGATCTGTATTTTGAGCGTTACCACGGTGTTTTGGAATACAT<br>GGAGCGTACGCGCGCGCAAGCTAAGGAACAAGGTTATGTGGAAAC<br>CTTGGATGGTCGTCGCTTGTACTTGCCTGACATTAAGTCTTCTAAC<br>GGCGCCCGCCGCGCTGCTGCCGAGCGCGCTGCTATCAATGCTCCG<br>ATGCAAGGTACTGCTGCTGATATTATTAAGCGTGCTATGATCGCTG<br>TGGACGCTTGGCTGCAAGCTGAACAGCCTCGCGTTCGCATGATTAT

	GCAAGTTCATGACGAGTTGGTTTTCGAGGTGCATAAGGACGACGT<br>GGACGCTGTTGCTAAACAAATCCACCAGTTGATGGAGAATTGCAC<br>GCGCTTAGACGTTCCGCTGCTGGTTGAAGTTGGTTCTGGTGAAAAC<br>TGGGACCAGGCTCACTAATAATGGCTGAAAACTGGGACCAGGCTC<br>ACTAACTCAGATCCTACTAGGTTTAATAAACATCTTTATTTTCATTA<br>CATCTGTGTGTTGGTTTTTTGTGTGGTACCCAATTCGCCCTATAGTG<br>AGTCGTATTACTCACTGGCCGTCGTTTTACAACGTCGTGACTGGGA<br>AAACCCTGGCGTTACCCAACTTAATCGCCTTGCAGCACATCCCCCT<br>TTCGCCAGCTGGCGTAATAGCGAAGAGGCCCGCACCGATCGCCCT<br>TCCCAACAGTTGCGCAGCCTGAATGGCGAATGGGACGCGCCCTGT<br>AGCGGCGCATTAAGCGCGGCGGGTGTGGTGGTTACGCGCAGCGTG<br>ACCGCTACACTTGCCAGCGCCCTAGCGCCCGCTCCTTTCGCTTTCTT<br>CCCTTCCTTTCTCGCCACGTTCGCCGGCTTTCCCCGTCAAGCTCTAA<br>ATCGGGGGCTCCCTTTAGGGTTCCGATTTAGTGCTTTACGGCACCT<br>CGACCCCAAAAAACTTGATTAGGGTGATGGTTCACGTAGTGGGCC<br>ATCGCCCTGATAGACGGTTTTTCGCCCTTTGACGTTGGAGTCCACG<br>TTCTTTAATAGTGGACTCTTGTTCCAAACTGGAACAACACTCAACC<br>CTATCTCGGTCTATTCTTTTGATTTATAAGGGATTTTGCCGATTTCG<br>GCCTATTGGTTAAAAAATGAGCTGATTTAACAAAAATTTAACGCG<br>AATTTTAACAAAATATTAACGCTTACAATTTAGGTGGCACTTTTCG<br>GGGAAATGTG<br><br>**Ampicillin resistance cassette-ColE1 origin of replication-hU6 promoter-gRNA sequence-SpCas9 gRNA scaffold-CMV promoter-NLS-mCherry-GSGM-dCas9(D10A, H840A)-NLS-linker-PolI5M-stop-SV40 polyA**
p.enCas9-PolI3M	CGCGGAACCCCTATTTGTTTATTTTTCTAAATACATTCAAATATGTA<br>TCCGCTCATGAGACAATAACCCTGATAAATGCTTCAATAATATTGA<br>AAAAGGAAGAGTATGAGTATTCAACATTTCCGTGTCGCCCTTATTC<br>CCTTTTTTGCGGCATTTTGCCTTCCTGTTTTTGCTCACCCAGAAACG<br>CTGGTGAAAGTAAAAGATGCTGAAGATCAGTTGGGTGCACGAGTG<br>GGTTACATCGAACTGGATCTCAACAGCGGTAAGATCCTTGAGAGT<br>TTTCGCCCCGAAGAACGTTTTCCAATGATGAGCACTTTTAAAGTTC<br>TGCTATGTGGCGCGGTATTATCCCGTATTGACGCCGGGCAAGAGC<br>AACTCGGTCGCCGCATACACTATTCTCAGAATGACTTGGTTGAGTA<br>CTCACCAGTCACAGAAAAGCATCTTACGGATGGCATGACAGTAAG<br>AGAATTATGCAGTGCTGCCATAACCATGAGTGATAACACTGCGGC<br>CAACTTACTTCTGACAACGATCGGAGGACCGAAGGAGCTAACCGC<br>TTTTTTGCACAACATGGGGGATCATGTAACTCGCCTTGATCGTTGG<br>GAACCGGAGCTGAATGAAGCCATACCAAACGACGAGCGTGACACC<br>ACGATGCCTGTAGCAATGGCAACAACGTTGCGCAAACTATTAACT<br>GGCGAACTACTTACTCTAGCTTCCCGGCAACAATTAATAGACTGGA<br>TGGAGGCGGATAAAGTTGCAGGACCACTTCTGCGCTCGGCCCTTCC<br>GGCTGGCTGGTTTATTGCTGATAAATCTGGAGCCGGTGAGCGTGGT<br>TCTCGCGGTATCATTGCAGCACTGGGGCCAGATGGTAAGCCCTCCC

	GTATCGTAGTTATCTACACGACGGGGAGTCAGGCAACTATGGATG<br>AACGAAATAGACAGATCGCTGAGATAGGTGCCTCACTGATTAAGC<br>ATTGGTAACTGTCAGACCAAGTTTACTCATATATACTTTAGATTGA<br>TTTAAAACTTCATTTTTAATTTAAAAGGATCTAGGTGAAGATCCTT<br>TTTGATAATCTCATGACCAAAATCCCTTAACGTGAGTTTTCGTTCC<br>ACTGAGCGTCAGACCCCGTAGAAAAGATCAAAGGATCTTCTTGAG<br>ATCCTTTTTTTCTGCGCGTAATCTGCTGCTTGCAAACAAAAAAACC<br>ACCGCTACCAGCGGTGGTTTGTTTGCCGGATCAAGAGCTACCAACT<br>CTTTTTCCGAAGGTAACTGGCTTCAGCAGAGCGCAGATACCAAAT<br>ACTGTCCTTCTAGTGTAGCCGTAGTTAGGCCACCACTTCAAGAACT<br>CTGTAGCACCGCCTACATACCTCGCTCTGCTAATCCTGTTACCAGT<br>GGCTGCTGCCAGTGGCGATAAGTCGTGTCTTACCGGGTTGGACTCA<br>AGACGATAGTTACCGGATAAGGCGCAGCGGTCGGGCTGAACGGGG<br>GGTTCGTGCACACAGCCCAGCTTGGAGCGAACGACCTACACCGAA<br>CTGAGATACCTACAGCGTGAGCTATGAGAAAGCGCCACGCTTCCC<br>GAAGGGAGAAAGGCGGACAGGTATCCGGTAAGCGGCAGGGTCGG<br>AACAGGAGAGCGCACGAGGGAGCTTCCAGGGGGAAACGCCTGGT<br>ATCTTTATAGTCCTGTCGGGTTTCGCCACCTCTGACTTGAGCGTCG<br>ATTTTTGTGATGCTCGTCAGGGGGGCGGAGCCTATGGAAAAACGC<br>CAGCAACGCGGCCTTTTTACGGTTCCTGGCCTTTTGCTGGCCTTTTG<br>CTCACATGTTCTTTCCTGCGTTATCCCCTGATTCTGTGGATAACCGT<br>ATTACCGCCTTTGAGTGAGCTGATACCGCTCGCCGCAGCCGAACG<br>ACCGAGCGCAGCGAGTCAGTGAGCGAGGAAGCGGAAGAGCGCCC<br>AATACGCAAACCGCCTCTCCCCGCGCGTTGGCCGATTCATTAATGC<br>AGCTGGCACGACAGGTTTCCCGACTGGAAAGCGGGCAGTGAGCGC<br>AACGCAATTAATGTGAGTTAGCTCACTCATTAGGCACCCCAGGCTT<br>TACACTTTATGCTTCCGGCTCGTATGTTGTGTGGAATTGTGAGCGG<br>ATAACAATTTCACACAGGAAACAGCTATGACCATGATTACGCCAA<br>GCGCGCAATTAACCCTCACTAAAGGGAACAAAAGCTGGAGCTCCA<br>CCGCGGTGGCGGCCGCTCTTAAGGGGTGCAGCGGCCTCCGCGCCG<br>GGTTTTGGCGCCTCGATCCAAGGTCGGGCAGGAAGAGGGCCTATT<br>TCCCATGATTCCTTCATATTTGCATATACGATACAAGGCTGTTAGA<br>GAGATAATTAGAATTAATTTGACTGTAAACACAAAGATATTAGTA<br>CAAAATACGTGACGTAGAAAGTAATAATTTCTTGGGTAGTTTGCA<br>GTTTTAAAATTATGTTTTAAAATGGACTATCATATGCTTACCGTAA<br>CTTGAAAGTATTTCGATTTCTTGGCTTTATATATCTTGTGGAAAGG<br>ACGAAACACC-[gRNA SEQUENCE]-<br>GTTTTAGAGCTAGAAATAGCAAGTTAAAATAAGGCTAGTCCGTTA<br>TCAACTTGAAAAAGTGGCACCGAGTCGGTGCTTTTTTCCGCGGCCT<br>CTAGACTCGAGGCGTTGACATTGATTATTGACTAGTTATTAATAGT<br>AATCAATTACGGGGTCATTAGTTCATAGCCCATATATGGAGTTCCG<br>CGTTACATAACTTACGGTAAATGGCCCGCCTGGCTGACCGCCCAAC<br>GACCCCCGCCCATTGACGTCAATAATGACGTATGTTCCCATAGTAA<br>CGCCAATAGGGACTTTCCATTGACGTCAATGGGTGGAGTATTTACG<br>GTAAACTGCCCACTTGGCAGTACATCAAGTGTATCATATGCCAAGT<br>ACGCCCCCTATTGACGTCAATGACGGTAAATGGCCCGCCTGGCATT

	ATGCCCAGTACATGACCTTATGGGACTTTCCTACTTGGCAGTACAT<br>CTACGTATTAGTCATCGCTATTACCATGGTGATGCGGTTTTGGCAG<br>TACATCAATGGGCGTGGATAGCGGTTTGACTCACGGGGATTTCCA<br>AGTCTCCACCCCATTGACGTCAATGGGAGTTTGTTTTGGCACCAAA<br>ATCAACGGGACTTTCCAAAATGTCGTAACAACTCCGCCCCATTGAC<br>GCAAATGGGCGGTAGGCGTGTACGGTGGGAGGTCTATATAAGCAG<br>AGCTCTCTGGCTAACTACCGGTGCCACCATGGCCCCAAAGAAGAA<br>GCGGAAGGTCGGTATCCACGGAGTCCCAGCAGCCGTGAGCAAGGG<br>CGAGGAGGATAACATGGCCATCATCAAGGAGTTCATGCGCTTCAA<br>GGTGCACATGGAGGGCTCCGTGAACGGCCACGAGTTCGAGATCGA<br>GGGCGAGGGCGAGGGCCGCCCCTACGAGGGCACCCAGACCGCCA<br>AGCTGAAGGTGACCAAGGGTGGCCCCCTGCCCTTCGCCTGGGACA<br>TCCTGTCCCCTCAGTTCATGTACGGCTCCAAGGCCTACGTGAAGCA<br>CCCCGCCGACATCCCCGACTACTTGAAGCTGTCCTTCCCCGAGGGC<br>TTCAAGTGGGAGCGCGTGATGAACTTCGAGGACGGCGGCGTGGTG<br>ACCGTGACCCAGGACTCCTCCCTGCAGGACGGCGAGTTCATCTAC<br>AAGGTGAAGCTGCGCGGCACCAACTTCCCCTCCGACGGCCCCGTA<br>ATGCAGAAGAAGACCATGGGCTGGGAGGCCTCCTCCGAGCGGATG<br>TACCCCGAGGACGGCGCCCTGAAGGGCGAGATCAAGCAGAGGCTG<br>AAGCTGAAGGACGGCGGCCACTACGACGCTGAGGTCAAGACCACC<br>TACAAGGCCAAGAAGCCCGTGCAGCTGCCCGGCGCCTACAACGTC<br>AACATCAAGTTGGACATCACCTCCCACAACGAGGACTACACCATC<br>GTGGAACAGTACGAACGCGCCGAGGGCCGCCACTCCACCGGCGGC<br>ATGGACGAGCTGTACAAGGGATCCGGTATGGACAAGAAGTACAGC<br>ATCGGCCTGGCCATCGGCACCAACTCTGTGGGCTGGGCCGTGATC<br>ACCGACGAGTACAAGGTGCCCAGCAAGAAATTCAAGGTGCTGGGC<br>AACACCGACCGGCACAGCATCAAGAAGAACCTGATCGGCGCCCTG<br>CTGTTCGACAGCGGAGAAACAGCCGAGGCCACCCGGCTGAAGAGA<br>ACCGCCAGAAGAAGATACACCAGACGGAAGAACCGGATCTGCTAT<br>CTGCAAGAGATCTTCAGCAACGAGATGGCCAAGGTGGACGACAGC<br>TTCTTCCACAGACTGGAAGAGTCCTTCCTGGTGGAAGAGGATAAG<br>AAGCACGAGCGGCACCCCATCTTCGGCAACATCGTGGACGAGGTG<br>GCCTACCACGAGAAGTACCCCACCATCTACCACCTGAGAAAGAAA<br>CTGGTGGACAGCACCGACAAGGCCGACCTGCGGCTGATCTATCTG<br>GCCCTGGCCCACATGATCAAGTTCCGGGGCCACTTCCTGATCGAGG<br>GCGACCTGAACCCCGACAACAGCGACGTGGACAAGCTGTTCATCC<br>AGCTGGTGCAGACCTACAACCAGCTGTTCGAGGAAAACCCCATCA<br>ACGCCAGCGGCGTGGACGCCAAGGCCATCCTGTCTGCCAGACTGA<br>GCAAGAGCAGACGGCTGGAAAATCTGATCGCCCAGCTGCCCGGCG<br>AGAAGAAGAATGGCCTGTTCGGCAACCTGATTGCCCTGAGCCTGG<br>GCCTGACCCCCAACTTCAAGAGCAACTTCGACCTGGCCGAGGATG<br>CCAAACTGCAGCTGAGCAAGGACACCTACGACGACGACCTGGACA<br>ACCTGCTGGCCCAGATCGGCGACCAGTACGCCGACCTGTTTCTGGC<br>CGCCAAGAACCTGTCCGACGCCATCCTGCTGAGCGACATCCTGAG<br>AGTGAACACCGAGATCACCAAGGCCCCCCTGAGCGCCTCTATGAT<br>CAAGAGATACGACGAGCACCACCAGGACCTGACCCTGCTGAAAGC

	TCTCGTGCGGCAGCAGCTGCCTGAGAAGTACAAAGAGATTTTCTTC<br>GACCAGAGCAAGAACGGCTACGCCGGCTACATCGATGGCGGAGCC<br>AGCCAGGAAGAGTTCTACAAGTTCATCAAGCCCATCCTGGAAAAG<br>ATGGACGGCACCGAGGAACTGCTCGTGAAGCTGAACAGAGAGGA<br>CCTGCTGCGGAAGCAGCGGACCTTCGACAACGGCAGCATCCCCCA<br>CCAGATCCACCTGGGAGAGCTGCACGCCATTCTGCGGCGGCAGGA<br>AGATTTTTACCCATTCCTGAAGGACAACCGGGAAAAGATCGAGAA<br>GATCCTGACCTTCCGCATCCCCTACTACGTGGGCCCTCTGGCCAGG<br>GGAAACAGCAGATTCGCCTGGATGACCAGAAAGAGCGAGGAAAC<br>CATCACCCCCTGGAACTTCGAGGAAGTGGTGGACAAGGGCGCCAG<br>CGCCCAGAGCTTCATCGAGCGGATGACCAACTTCGATAAGAACCT<br>GCCCAACGAGAAGGTGCTGCCCAAGCACAGCCTGCTGTACGAGTA<br>CTTCACCGTGTACAACGAGCTGACCAAAGTGAAATACGTGACCGA<br>GGGAATGAGAAAGCCCGCCTTCCTGAGCGGCGAGCAGAAAAAAG<br>CCATCGTGGACCTGCTGTTCAAGACCAACCGGAAAGTGACCGTGA<br>AGCAGCTGAAAGAGGACTACTTCAAGAAAATCGAGTGCTTCGACT<br>CCGTGGAAATCTCCGGCGTGGAAGATCGGTTCAACGCCTCCCTGG<br>GCACATACCACGATCTGCTGAAAATTATCAAGGACAAGGACTTCC<br>TGGACAATGAGGAAAACGAGGACATTCTGGAAGATATCGTGCTGA<br>CCCTGACACTGTTTGAGGACAGAGAGATGATCGAGGAACGGCTGA<br>AAACCTATGCCCACCTGTTCGACGACAAAGTGATGAAGCAGCTGA<br>AGCGGCGGAGATACACCGGCTGGGGCAGGCTGAGCCGGAAGCTG<br>ATCAACGGCATCCGGGACAAGCAGTCCGGCAAGACAATCCTGGAT<br>TTCCTGAAGTCCGACGGCTTCGCCAACAGAAACTTCATGCAGCTGA<br>TCCACGACGACAGCCTGACCTTTAAAGAGGACATCCAGAAAGCCC<br>AGGTGTCCGGCCAGGGCGATAGCCTGCACGAGCACATTGCCAATC<br>TGGCCGGCAGCCCCGCCATTAAGAAGGGCATCCTGCAGACAGTGA<br>AGGTGGTGGACGAGCTCGTGAAAGTGATGGGCCGGCACAAGCCCG<br>AGAACATCGTGATCGAAATGGCCAGAGAGAACCAGACCACCCAG<br>AAGGGACAGAAGAACAGCCGCGAGAGAATGAAGCGGATCGAAGA<br>GGGCATCAAAGAGCTGGGCAGCCAGATCCTGAAAGAACACCCCGT<br>GGAAAACACCCAGCTGCAGAACGAGAAGCTGTACCTGTACTACCT<br>GCAGAATGGGCGGGATATGTACGTGGACCAGGAACTGGACATCAA<br>CCGGCTGTCCGACTACGATGTGGACCATATCGTGCCTCAGAGCTTT<br>CTGGCAGACGACTCCATCGATAACAAAGTGCTGACTCGGAGCGAC<br>AAGAACCGGGGCAAGAGCGACAACGTGCCCTCCGAAGAGGTCGT<br>GAAGAAGATGAAGAACTACTGGCGCCAGCTGCTGAATGCCAAGCT<br>GATTACCCAGAGGAAGTTCGACAATCTGACCAAGGCCGAGAGAGG<br>CGGCCTGAGCGAACTGGATAAGGCCGGCTTCATCAAGAGACAGCT<br>GGTGGAAACCCGGCAGATCACAAAGCACGTGGCACAGATCCTGGA<br>CTCCCGGATGAACACTAAGTACGACGAGAACGACAAACTGATCCG<br>GGAAGTGAAAGTGATCACCCTGAAGTCCAAGCTGGTGTCCGATTT<br>CCGGAAGGATTTCCAGTTTTACAAAGTGCGCGAGATCAACAACTA<br>CCACCACGCCCACGACGCCTACCTGAACGCCGTCGTGGGAACCGC<br>CCTGATCAAAAAGTACCCTGCGCTGGAAAGCGAGTTCGTGTACGG<br>CGACTACAAGGTGTACGACGTGCGGAAGATGATCGCCAAGAGCGA

	GCAGGAAATCGGCAAGGCTACCGCCAAGTACTTCTTCTACAGCAA<br>CATCATGAACTTTTTCAAGACCGAGATTACCCTGGCCAACGGCGA<br>GATCCGGAAGGCGCCTCTGATCGAGACAAACGGCGAAACAGGCG<br>AGATCGTGTGGGATAAGGGCCGGGACTTTGCCACCGTGCGGAAAG<br>TGCTGTCTATGCCCCAAGTGAATATCGTGAAAAAGACCGAGGTGC<br>AGACAGGCGGCTTCAGCAAAGAGTCTATCCTGCCCAAGAGGAACA<br>GCGACAAGCTGATCGCCAGAAAGAAGGACTGGGACCCTAAGAAG<br>TACGGCGGCTTCGACAGCCCCACCGTGGCCTATTCTGTGCTGGTGG<br>TGGCCAAAGTGGAAAAGGGCAAGTCCAAGAAACTGAAGAGTGTG<br>AAAGAGCTGCTGGGGATCACCATCATGGAAAGAAGCAGCTTCGAG<br>AAGAATCCCATCGACTTTCTGGAAGCCAAGGGCTACAAAGAAGTG<br>AAAAAGGACCTGATCATCAAGCTGCCTAAGTACTCCCTGTTCGAG<br>CTGGAAAACGGCCGGAAGAGAATGCTGGCCTCTGCCGGCGAACTG<br>CAGAAGGGAAACGAACTGGCCCTGCCCTCCAAATATGTGAACTTC<br>CTGTACCTGGCCAGCCACTATGAGAAGCTGAAGGGCTCCCCCGAG<br>GATAATGAGCAGAAACAGCTGTTTGTGGAACAGCACAAACACTAC<br>CTGGACGAGATCATCGAGCAGATCAGCGAGTTCTCCAAGAGAGTG<br>ATCCTGGCCGACGCTAATCTGGACAAGGTGCTGAGCGCCTACAAC<br>AAGCACAGAGACAAGCCTATCAGAGAGCAGGCCGAGAATATCATC<br>CACCTGTTTACCCTGACCAATCTGGGAGCCCCTGCCGCCTTCAAGT<br>ACTTTGACACCACCATCGACCGGAAGAGGTACACCAGCACCAAAG<br>AGGTGCTGGACGCCACCCTGATCCACCAGAGCATCACCGGCCTGT<br>ACGAGACACGGATCGACCTGTCTCAGCTGGGAGGCGACGCCTATC<br>CCTATGACGTGCCCGATTATGCCAGCCTGGGCAGCGGCTCCCCCAA<br>GAAAAAACGCAAGGTGGAAGATCCTAAGAAAAAGCGGAAAGGTT<br>CTAGTGAAACCCCGGGAACAAGTGAGTCGGCCACCCCTGAAGGTG<br>GATCAGGGGGTAGCGGATCCGTTCAGATCCCGCAGAACCCGCTGA<br>TTCTGGTTGACGGATCTAGTTACCTGTACCGTGCTTACCATGCTTTC<br>CCGCCTTTGACCAATTCTGCTGGTGAACCTACGGGAGCTATGTACG<br>GAGTTCTGAATATGTTGCGTTCTTTAATTATGCAGTACAAGCCTAC<br>CCACGCTGCTGTTGTTTTCGATGCTAAAGGTAAGACGTTCCGCGAC<br>GAGTTATTCGAGCACTATAAGTCTCACCGTCCTCCGATGCCTGATG<br>ACTTACGCGCTCAGATTGAGCCGCTGCATGCTATGGTGAAGGCTAT<br>GGGTTTACCTCTTTTGGCTGTCAGCGGTGTTGAGGCTGATGATGTC<br>ATTGGCACCTTAGCTCGTGAGGCTGAGAAGGCTGGTCGCCCTGTTT<br>TGATTTCTACCGGTGACAAGGACATGGCTCAATTGGTTACCCCGAA<br>CATCACCCTGATCAACACCATGACCAACACGATTCTGGGTCCTGAG<br>GAAGTTGTTAACAAATATGGTGTTCCTCCGGAGTTGATTATTGACT<br>TTCTTGCTCTTATGGGCGATTCTTCAGACAATATCCCGGGTGTTCC<br>AGGTGTTGGAGAGAAGACTGCTCAAGCTCTGCTTCAGGGTCTGGG<br>TGGTTTGGACACCCTTTACGCTGAACCGGAGAAGATCGCCGGTCTG<br>TCTTTTCGCGGTGCTAAGACCATGGCTGCTAAACTGGAACAGAATA<br>AGGAGGTCGCATACCTGTCTTATCAATTGGCTACCATCAAGACGG<br>ATGTGGAGTTAGAACTTACGTGCGAGCAGCTTGAGGTTCAACAGC<br>CTGCTGCTGAGGAACTGCTGGGTCTTTTTAAGAAATACGAATTTAA<br>GCGTTGGACCGCCGACGTTGAGGCTGGTAAGTGGCTGCAAGCTAA

	GGGTGCTAAGCCGGCTGCTAAACCGCAAGAAACGAGTGTCGCTGA<br>TGAGGCTCCGGAGGTTACCGCTACCGTTATCTCTTACGATAATTAT<br>GTTACGATTCTGGACGAGGAAACCTTAAAGGCTTGGATCGCTAAA<br>TTAGAGAAGGCTCCTGTTTTCGCTTTCGACACGGAAACGGATTCTC<br>TGGACAATATTAGTGCGAATCTTGTTGGTCTGAGTTTCGCAATTGA<br>ACCGGGTGTTGCTGCTTACATCCCTGTGGCACACGACTACCTGGAC<br>GCTCCGGACCAGATTTCACGTGAACGCGCTCTGGAACTGCTGAAG<br>CCTTTATTAGAGGACGAGAAAGCTTTGAAAGTTGGTCAGAATTTG<br>AAGTATGCTCGTGGAATCTTAGCTAATTATGGTATCGAGTTGCGCG<br>GTATCGCTTTCGACACGATGTTGGAATCTTATATCCTGAACTCTGT<br>CGCTGGTCGCCATGACATGGACTCTCTGGCTGAGCGCTGGCTGAA<br>ACATAAGACGATTACCTTCGAGGAAATCGCAGGAAAGGGTAAGAA<br>CCAGCTCACGTTCAATCAAATCGCTCTGGAGGAAGCTGGTCGCTAT<br>GCTGCTGAGGACGCTGACGTTACTCTGCAACTGCACTTGAAGATGT<br>GGCCTGACTTGCAGAAGCATAAGGGTCCACTGAATGTTTTTGAAA<br>ACATTGAGATGCCTTTGGTTCCAGTTCTGTCTCGTATCGAGCGCAA<br>TGGCGTTAAAATTGACCCAAAGGTTTTACATAACCACTCAGAGGA<br>ACTGACGCTGCGCTTAGCCGAATTGGAGAAAAAGGCTCACGAGAT<br>CGCTGGCGAAGAGTTCAATCTGTCATCTACGAAACAACTGCAGAC<br>TATCCTGTTCGAGAAGCAAGGTATCAAGCCATTAAAAAAGACCCC<br>TGGCGGTGCTCCGTCTACCTCTGAGGAAGTTTTGGAGGAGTTAGCT<br>TTGGATTACCCTCTGCCGAAGGTTATCTTGGAATACCGCGGTTTGG<br>CTAAATTGAAGTCTACTTATACGGATAAACTTCCTTTGATGATTAA<br>TCCAAAGACGGGTCGCGTTCACACGTCGTACCATCAAGCTGTTACC<br>GCTACCGGTCGCCTGTCTTCTACGGATCCGAATTTACAGAATATTC<br>CTGTGCGCAATGAGGAGGGCCGCCGCATTCGTCAAGCTTTTATCGC<br>TCCGGAAGACTACGTTATCGTTTCTGCTGATTATTCTCAAAATGAA<br>TTACGTATCATGGCTCACCTGTCTCGCGATAAGGGTCTGTTGACGG<br>CCTTTGCTGAGGGTAAGGACATTCATCGTGCTACCGCTGCTGAGGT<br>TTTCGGCCTGCCGTTGGAAACGGTTACGTCTGAACAGCGTCGCTCT<br>GCTAAGCGTATTAATTTCGGCTTAATCTACGGTATGTCTGCGTTTG<br>GCTTAGCTCGTCAGCTGAATATCCCGCGCAAGGAAGCTCAAAAAT<br>ATATGGATCTGTATTTTGAGCGTTACCCGGGTGTTTTGGAATACAT<br>GGAGCGTACGCGCGCGCAAGCTAAGGAACAAGGTTATGTGGAAAC<br>CTTGGATGGTCGTCGCTTGTACTTGCCTGACATTAAGTCTTCTAAC<br>GGCGCCCGCCGCGCTGCTGCCGAGCGCGCTGCTATCAATGCTCCG<br>ATGCAAGGTACTGCTGCTGATATTATTAAGCGTGCTATGATCGCTG<br>TGGACGCTTGGCTGCAAGCTGAACAGCCTCGCGTTCGCATGATTAT<br>GCAAGTTCATGACGAGTTGGTTTTCGAGGTGCATAAGGACGACGT<br>GGACGCTGTTGCTAAACAAATCCACCAGTTGATGGAGAATTGCAC<br>GCGCTTAGACGTTCCGCTGCTGGTTGAAGTTGGTTCTGGTGAAAAC<br>TGGGACCAGGCTCACTAATAATGGCTGAAAACTGGGACCAGGCTC<br>ACTAACTCAGATCCTACTAGGTTTAATAAACATCTTTATTTTCATTA<br>CATCTGTGTGTTGGTTTTTTGTGTGGTACCCAATTCGCCCTATAGTG<br>AGTCGTATTACTCACTGGCCGTCGTTTTACAACGTCGTGACTGGGA<br>AAACCCTGGCGTTACCCAACTTAATCGCCTTGCAGCACATCCCCCT

	TTCGCCAGCTGGCGTAATAGCGAAGAGGCCCGCACCGATCGCCCT<br>TCCCAACAGTTGCGCAGCCTGAATGGCGAATGGGACGCGCCCTGT<br>AGCGGCGCATTAAGCGCGGCGGGTGTGGTGGTTACGCGCAGCGTG<br>ACCGCTACACTTGCCAGCGCCCTAGCGCCCGCTCCTTTCGCTTTCTT<br>CCCTTCCTTTCTCGCCACGTTCGCCGGCTTTCCCCGTCAAGCTCTAA<br>ATCGGGGGCTCCCTTTAGGGTTCCGATTTAGTGCTTTACGGCACCT<br>CGACCCCAAAAAACTTGATTAGGGTGATGGTTCACGTAGTGGGCC<br>ATCGCCCTGATAGACGGTTTTTCGCCCTTTGACGTTGGAGTCCACG<br>TTCTTTAATAGTGGACTCTTGTTCCAAACTGGAACAACACTCAACC<br>CTATCTCGGTCTATTCTTTTGATTTATAAGGGATTTTGCCGATTTCG<br>GCCTATTGGTTAAAAAATGAGCTGATTTAACAAAAATTTAACGCG<br>AATTTTAACAAAATATTAACGCTTACAATTTAGGTGGCACTTTTCG<br>GGGAAATGTG<br><br>**Ampicillin resistance cassette-ColE1 origin of replication-hU6 promoter-gRNA sequence-SpCas9 gRNA scaffold-CMV promoter-NLS-mCherry-GSGM- enCas9(D10A)-NLS-linker-PolI3M-stop-SV40 polyA**
p.enCas9-PolI5M	CGCGGAACCCCTATTTGTTTATTTTTCTAAATACATTCAAATATGTA<br>TCCGCTCATGAGACAATAACCCTGATAAATGCTTCAATAATATTGA<br>AAAAGGAAGAGTATGAGTATTCAACATTTCCGTGTCGCCCTTATTC<br>CCTTTTTTGCGGCATTTTGCCTTCCTGTTTTTGCTCACCCAGAAACG<br>CTGGTGAAAGTAAAAGATGCTGAAGATCAGTTGGGTGCACGAGTG<br>GGTTACATCGAACTGGATCTCAACAGCGGTAAGATCCTTGAGAGT<br>TTTCGCCCCGAAGAACGTTTTCCAATGATGAGCACTTTTAAAGTTC<br>TGCTATGTGGCGCGGTATTATCCCGTATTGACGCCGGGCAAGAGC<br>AACTCGGTCGCCGCATACACTATTCTCAGAATGACTTGGTTGAGTA<br>CTCACCAGTCACAGAAAAGCATCTTACGGATGGCATGACAGTAAG<br>AGAATTATGCAGTGCTGCCATAACCATGAGTGATAACACTGCGGC<br>CAACTTACTTCTGACAACGATCGGAGGACCGAAGGAGCTAACCGC<br>TTTTTTGCACAACATGGGGGATCATGTAACTCGCCTTGATCGTTGG<br>GAACCGGAGCTGAATGAAGCCATACCAAACGACGAGCGTGACACC<br>ACGATGCCTGTAGCAATGGCAACAACGTTGCGCAAACTATTAACT<br>GGCGAACTACTTACTCTAGCTTCCCGGCAACAATTAATAGACTGGA<br>TGGAGGCGGATAAAGTTGCAGGACCACTTCTGCGCTCGGCCCTTCC<br>GGCTGGCTGGTTTATTGCTGATAAATCTGGAGCCGGTGAGCGTGGT<br>TCTCGCGGTATCATTGCAGCACTGGGGCCAGATGGTAAGCCCTCCC<br>GTATCGTAGTTATCTACACGACGGGGAGTCAGGCAACTATGGATG<br>AACGAAATAGACAGATCGCTGAGATAGGTGCCTCACTGATTAAGC<br>ATTGGTAACTGTCAGACCAAGTTTACTCATATATACTTTAGATTGA<br>TTTAAAACTTCATTTTTAATTTAAAAGGATCTAGGTGAAGATCCTT<br>TTTGATAATCTCATGACCAAAATCCCTTAACGTGAGTTTTCGTTCC<br>ACTGAGCGTCAGACCCCGTAGAAAAGATCAAAGGATCTTCTTGAG<br>ATCCTTTTTTTCTGCGCGTAATCTGCTGCTTGCAAACAAAAAAACC<br>ACCGCTACCAGCGGTGGTTTGTTTGCCGGATCAAGAGCTACCAACT

	CTTTTTCCGAAGGTAACTGGCTTCAGCAGAGCGCAGATACCAAAT<br>ACTGTCCTTCTAGTGTAGCCGTAGTTAGGCCACCACTTCAAGAACT<br>CTGTAGCACCGCCTACATACCTCGCTCTGCTAATCCTGTTACCAGT<br>GGCTGCTGCCAGTGGCGATAAGTCGTGTCTTACCGGGTTGGACTCA<br>AGACGATAGTTACCGGATAAGGCGCAGCGGTCGGGCTGAACGGGG<br>GGTTCGTGCACACAGCCCAGCTTGGAGCGAACGACCTACACCGAA<br>CTGAGATACCTACAGCGTGAGCTATGAGAAAGCGCCACGCTTCCC<br>GAAGGGAGAAAGGCGGACAGGTATCCGGTAAGCGGCAGGGTCGG<br>AACAGGAGAGCGCACGAGGGAGCTTCCAGGGGGAAACGCCTGGT<br>ATCTTTATAGTCCTGTCGGGTTTCGCCACCTCTGACTTGAGCGTCG<br>ATTTTTGTGATGCTCGTCAGGGGGGCGGAGCCTATGGAAAAACGC<br>CAGCAACGCGGCCTTTTTACGGTTCCTGGCCTTTTGCTGGCCTTTTG<br>CTCACATGTTCTTTCCTGCGTTATCCCCTGATTCTGTGGATAACCGT<br>ATTACCGCCTTTGAGTGAGCTGATACCGCTCGCCGCAGCCGAACG<br>ACCGAGCGCAGCGAGTCAGTGAGCGAGGAAGCGGAAGAGCGCCC<br>AATACGCAAACCGCCTCTCCCCGCGCGTTGGCCGATTCATTAATGC<br>AGCTGGCACGACAGGTTTCCCGACTGGAAAGCGGGCAGTGAGCGC<br>AACGCAATTAATGTGAGTTAGCTCACTCATTAGGCACCCCAGGCTT<br>TACACTTTATGCTTCCGGCTCGTATGTTGTGTGGAATTGTGAGCGG<br>ATAACAATTTCACACAGGAAACAGCTATGACCATGATTACGCCAA<br>GCGCGCAATTAACCCTCACTAAAGGGAACAAAAGCTGGAGCTCCA<br>CCGCGGTGGCGGCCGCTCTTAAGGGGTGCAGCGGCCTCCGCGCCG<br>GGTTTTGGCGCCTCGATCCAAGGTCGGGCAGGAAGAGGGCCTATT<br>TCCCATGATTCCTTCATATTTGCATATACGATACAAGGCTGTTAGA<br>GAGATAATTAGAATTAATTTGACTGTAAACACAAAGATATTAGTA<br>CAAAATACGTGACGTAGAAAGTAATAATTTCTTGGGTAGTTTGCA<br>GTTTTAAAATTATGTTTTAAAATGGACTATCATATGCTTACCGTAA<br>CTTGAAAGTATTTCGATTTCTTGGCTTTATATATCTTGTGGAAAGG<br>ACGAAACACC-[gRNA SEQUENCE]-<br>GTTTTAGAGCTAGAAATAGCAAGTTAAAATAAGGCTAGTCCGTTA<br>TCAACTTGAAAAAGTGGCACCGAGTCGGTGCTTTTTTCCGCGGCCT<br>CTAGACTCGAGGCGTTGACATTGATTATTGACTAGTTATTAATAGT<br>AATCAATTACGGGGTCATTAGTTCATAGCCCATATATGGAGTTCCG<br>CGTTACATAACTTACGGTAAATGGCCCGCCTGGCTGACCGCCCAAC<br>GACCCCCGCCCATTGACGTCAATAATGACGTATGTTCCCATAGTAA<br>CGCCAATAGGGACTTTCCATTGACGTCAATGGGTGGAGTATTTACG<br>GTAAACTGCCCACTTGGCAGTACATCAAGTGTATCATATGCCAAGT<br>ACGCCCCCTATTGACGTCAATGACGGTAAATGGCCCGCCTGGCATT<br>ATGCCCAGTACATGACCTTATGGGACTTTCCTACTTGGCAGTACAT<br>CTACGTATTAGTCATCGCTATTACCATGGTGATGCGGTTTTGGCAG<br>TACATCAATGGGCGTGGATAGCGGTTTGACTCACGGGGATTTCCA<br>AGTCTCCACCCCATTGACGTCAATGGGAGTTTGTTTTGGCACCAAA<br>ATCAACGGGACTTTCCAAAATGTCGTAACAACTCCGCCCCATTGAC<br>GCAAATGGGCGGTAGGCGTGTACGGTGGGAGGTCTATATAAGCAG<br>AGCTCTCTGGCTAACTACCGGTGCCACCATGGCCCCAAAGAAGAA<br>GCGGAAGGTCGGTATCCACGGAGTCCCAGCAGCCGTGAGCAAGGG

	CGAGGAGGATAACATGGCCATCATCAAGGAGTTCATGCGCTTCAA<br>GGTGCACATGGAGGGCTCCGTGAACGGCCACGAGTTCGAGATCGA<br>GGGCGAGGGCGAGGGCCGCCCCTACGAGGGCACCCAGACCGCCA<br>AGCTGAAGGTGACCAAGGGTGGCCCCCTGCCCTTCGCCTGGGACA<br>TCCTGTCCCCTCAGTTCATGTACGGCTCCAAGGCCTACGTGAAGCA<br>CCCCGCCGACATCCCCGACTACTTGAAGCTGTCCTTCCCCGAGGGC<br>TTCAAGTGGGAGCGCGTGATGAACTTCGAGGACGGCGGCGTGGTG<br>ACCGTGACCCAGGACTCCTCCCTGCAGGACGGCGAGTTCATCTAC<br>AAGGTGAAGCTGCGCGGCACCAACTTCCCCTCCGACGGCCCCGTA<br>ATGCAGAAGAAGACCATGGGCTGGGAGGCCTCCTCCGAGCGGATG<br>TACCCCGAGGACGGCGCCCTGAAGGGCGAGATCAAGCAGAGGCTG<br>AAGCTGAAGGACGGCGGCCACTACGACGCTGAGGTCAAGACCACC<br>TACAAGGCCAAGAAGCCCGTGCAGCTGCCCGGCGCCTACAACGTC<br>AACATCAAGTTGGACATCACCTCCCACAACGAGGACTACACCATC<br>GTGGAACAGTACGAACGCGCCGAGGGCCGCCACTCCACCGGCGGC<br>ATGGACGAGCTGTACAAGGGATCCGGTATGGACAAGAAGTACAGC<br>ATCGGCCTGGCCATCGGCACCAACTCTGTGGGCTGGGCCGTGATC<br>ACCGACGAGTACAAGGTGCCCAGCAAGAAATTCAAGGTGCTGGGC<br>AACACCGACCGGCACAGCATCAAGAAGAACCTGATCGGCGCCCTG<br>CTGTTCGACAGCGGAGAAACAGCCGAGGCCACCCGGCTGAAGAGA<br>ACCGCCAGAAGAAGATACACCAGACGGAAGAACCGGATCTGCTAT<br>CTGCAAGAGATCTTCAGCAACGAGATGGCCAAGGTGGACGACAGC<br>TTCTTCCACAGACTGGAAGAGTCCTTCCTGGTGGAAGAGGATAAG<br>AAGCACGAGCGGCACCCCATCTTCGGCAACATCGTGGACGAGGTG<br>GCCTACCACGAGAAGTACCCCACCATCTACCACCTGAGAAAGAAA<br>CTGGTGGACAGCACCGACAAGGCCGACCTGCGGCTGATCTATCTG<br>GCCCTGGCCCACATGATCAAGTTCCGGGGCCACTTCCTGATCGAGG<br>GCGACCTGAACCCCGACAACAGCGACGTGGACAAGCTGTTCATCC<br>AGCTGGTGCAGACCTACAACCAGCTGTTCGAGGAAAACCCCATCA<br>ACGCCAGCGGCGTGGACGCCAAGGCCATCCTGTCTGCCAGACTGA<br>GCAAGAGCAGACGGCTGGAAAATCTGATCGCCCAGCTGCCCGGCG<br>AGAAGAAGAATGGCCTGTTCGGCAACCTGATTGCCCTGAGCCTGG<br>GCCTGACCCCCAACTTCAAGAGCAACTTCGACCTGGCCGAGGATG<br>CCAAACTGCAGCTGAGCAAGGACACCTACGACGACGACCTGGACA<br>ACCTGCTGGCCCAGATCGGCGACCAGTACGCCGACCTGTTTCTGGC<br>CGCCAAGAACCTGTCCGACGCCATCCTGCTGAGCGACATCCTGAG<br>AGTGAACACCGAGATCACCAAGGCCCCCCTGAGCGCCTCTATGAT<br>CAAGAGATACGACGAGCACCACCAGGACCTGACCCTGCTGAAAGC<br>TCTCGTGCGGCAGCAGCTGCCTGAGAAGTACAAAGAGATTTTCTTC<br>GACCAGAGCAAGAACGGCTACGCCGGCTACATCGATGGCGGAGCC<br>AGCCAGGAAGAGTTCTACAAGTTCATCAAGCCCATCCTGGAAAAG<br>ATGGACGGCACCGAGGAACTGCTCGTGAAGCTGAACAGAGAGGA<br>CCTGCTGCGGAAGCAGCGGACCTTCGACAACGGCAGCATCCCCCA<br>CCAGATCCACCTGGGAGAGCTGCACGCCATTCTGCGGCGGCAGGA<br>AGATTTTTACCCATTCCTGAAGGACAACCGGGAAAAGATCGAGAA<br>GATCCTGACCTTCCGCATCCCCTACTACGTGGGCCCTCTGGCCAGG

	GGAAACAGCAGATTCGCCTGGATGACCAGAAAGAGCGAGGAAAC<br>CATCACCCCCTGGAACTTCGAGGAAGTGGTGGACAAGGGCGCCAG<br>CGCCCAGAGCTTCATCGAGCGGATGACCAACTTCGATAAGAACCT<br>GCCCAACGAGAAGGTGCTGCCCAAGCACAGCCTGCTGTACGAGTA<br>CTTCACCGTGTACAACGAGCTGACCAAAGTGAAATACGTGACCGA<br>GGGAATGAGAAAGCCCGCCTTCCTGAGCGGCGAGCAGAAAAAAG<br>CCATCGTGGACCTGCTGTTCAAGACCAACCGGAAAGTGACCGTGA<br>AGCAGCTGAAAGAGGACTACTTCAAGAAAATCGAGTGCTTCGACT<br>CCGTGGAAATCTCCGGCGTGGAAGATCGGTTCAACGCCTCCCTGG<br>GCACATACCACGATCTGCTGAAAATTATCAAGGACAAGGACTTCC<br>TGGACAATGAGGAAAACGAGGACATTCTGGAAGATATCGTGCTGA<br>CCCTGACACTGTTTGAGGACAGAGAGATGATCGAGGAACGGCTGA<br>AAACCTATGCCCACCTGTTCGACGACAAAGTGATGAAGCAGCTGA<br>AGCGGCGGAGATACACCGGCTGGGGCAGGCTGAGCCGGAAGCTG<br>ATCAACGGCATCCGGGACAAGCAGTCCGGCAAGACAATCCTGGAT<br>TTCCTGAAGTCCGACGGCTTCGCCAACAGAAACTTCATGCAGCTGA<br>TCCACGACGACAGCCTGACCTTTAAAGAGGACATCCAGAAAGCCC<br>AGGTGTCCGGCCAGGGCGATAGCCTGCACGAGCACATTGCCAATC<br>TGGCCGGCAGCCCCGCCATTAAGAAGGGCATCCTGCAGACAGTGA<br>AGGTGGTGGACGAGCTCGTGAAAGTGATGGGCCGGCACAAGCCCG<br>AGAACATCGTGATCGAAATGGCCAGAGAGAACCAGACCACCCAG<br>AAGGGACAGAAGAACAGCCGCGAGAGAATGAAGCGGATCGAAGA<br>GGGCATCAAAGAGCTGGGCAGCCAGATCCTGAAAGAACACCCCGT<br>GGAAAACACCCAGCTGCAGAACGAGAAGCTGTACCTGTACTACCT<br>GCAGAATGGGCGGGATATGTACGTGGACCAGGAACTGGACATCAA<br>CCGGCTGTCCGACTACGATGTGGACCATATCGTGCCTCAGAGCTTT<br>CTGGCAGACGACTCCATCGATAACAAAGTGCTGACTCGGAGCGAC<br>AAGAACCGGGGCAAGAGCGACAACGTGCCCTCCGAAGAGGTCGT<br>GAAGAAGATGAAGAACTACTGGCGCCAGCTGCTGAATGCCAAGCT<br>GATTACCCAGAGGAAGTTCGACAATCTGACCAAGGCCGAGAGAGG<br>CGGCCTGAGCGAACTGGATAAGGCCGGCTTCATCAAGAGACAGCT<br>GGTGGAAACCCGGCAGATCACAAAGCACGTGGCACAGATCCTGGA<br>CTCCCGGATGAACACTAAGTACGACGAGAACGACAAACTGATCCG<br>GGAAGTGAAAGTGATCACCCTGAAGTCCAAGCTGGTGTCCGATTT<br>CCGGAAGGATTTCCAGTTTTACAAAGTGCGCGAGATCAACAACTA<br>CCACCACGCCCACGACGCCTACCTGAACGCCGTCGTGGGAACCGC<br>CCTGATCAAAAAGTACCCTGCGCTGGAAAGCGAGTTCGTGTACGG<br>CGACTACAAGGTGTACGACGTGCGGAAGATGATCGCCAAGAGCGA<br>GCAGGAAATCGGCAAGGCTACCGCCAAGTACTTCTTCTACAGCAA<br>CATCATGAACTTTTTCAAGACCGAGATTACCCTGGCCAACGGCGA<br>GATCCGGAAGGCGCCTCTGATCGAGACAAACGGCGAAACAGGCG<br>AGATCGTGTGGGATAAGGGCCGGGACTTTGCCACCGTGCGGAAAG<br>TGCTGTCTATGCCCCAAGTGAATATCGTGAAAAAGACCGAGGTGC<br>AGACAGGCGGCTTCAGCAAAGAGTCTATCCTGCCCAAGAGGAACA<br>GCGACAAGCTGATCGCCAGAAAGAAGGACTGGGACCCTAAGAAG<br>TACGGCGGCTTCGACAGCCCCACCGTGGCCTATTCTGTGCTGGTGG

	TGGCCAAAGTGGAAAAGGGCAAGTCCAAGAAACTGAAGAGTGTG<br>AAAGAGCTGCTGGGGATCACCATCATGGAAAGAAGCAGCTTCGAG<br>AAGAATCCCATCGACTTTCTGGAAGCCAAGGGCTACAAAGAAGTG<br>AAAAAGGACCTGATCATCAAGCTGCCTAAGTACTCCCTGTTCGAG<br>CTGGAAAACGGCCGGAAGAGAATGCTGGCCTCTGCCGGCGAACTG<br>CAGAAGGGAAACGAACTGGCCCTGCCCTCCAAATATGTGAACTTC<br>CTGTACCTGGCCAGCCACTATGAGAAGCTGAAGGGCTCCCCCGAG<br>GATAATGAGCAGAAACAGCTGTTTGTGGAACAGCACAAACACTAC<br>CTGGACGAGATCATCGAGCAGATCAGCGAGTTCTCCAAGAGAGTG<br>ATCCTGGCCGACGCTAATCTGGACAAGGTGCTGAGCGCCTACAAC<br>AAGCACAGAGACAAGCCTATCAGAGAGCAGGCCGAGAATATCATC<br>CACCTGTTTACCCTGACCAATCTGGGAGCCCCTGCCGCCTTCAAGT<br>ACTTTGACACCACCATCGACCGGAAGAGGTACACCAGCACCAAAG<br>AGGTGCTGGACGCCACCCTGATCCACCAGAGCATCACCGGCCTGT<br>ACGAGACACGGATCGACCTGTCTCAGCTGGGAGGCGACGCCTATC<br>CCTATGACGTGCCCGATTATGCCAGCCTGGGCAGCGGCTCCCCCAA<br>GAAAAAACGCAAGGTGGAAGATCCTAAGAAAAAGCGGAAAGTGG<br>ACAGTGAAACCCCGGGAACAAGTGAGTCGGCCACCCCTGAAGGTG<br>GATCAGGGGGTAGCGGATCCGTTCAGATCCCGCAGAACCCGCTGA<br>TTCTGGTTGACGGATCTAGTTACCTGTACCGTGCTTACCATGCTTTC<br>CCGCCTTTGACCAATTCTGCTGGTGAACCTACGGGAGCTATGTACG<br>GAGTTCTGAATATGTTGCGTTCTTTAATTATGCAGTACAAGCCTAC<br>CCACGCTGCTGTTGTTTTCGATGCTAAAGGTAAGACGTTCCGCGAC<br>GAGTTATTCGAGCACTATAAGTCTCACCGTCCTCCGATGCCTGATG<br>ACTTACGCGCTCAGATTGAGCCGCTGCATGCTATGGTGAAGGCTAT<br>GGGTTTACCTCTTTTGGCTGTCAGCGGTGTTGAGGCTGATGATGTC<br>ATTGGCACCTTAGCTCGTGAGGCTGAGAAGGCTGGTCGCCCTGTTT<br>TGATTTCTACCGGTGACAAGGACATGGCTCAATTGGTTACCCCGAA<br>CATCACCCTGATCAACACCATGACCAACACGATTCTGGGTCCTGAG<br>GAAGTTGTTAACAAATATGGTGTTCCTCCGGAGTTGATTATTGACT<br>TTCTTGCTCTTATGGGCGATTCTTCAGACAATATCCCGGGTGTTCC<br>AGGTGTTGGAGAGAAGACTGCTCAAGCTCTGCTTCAGGGTCTGGG<br>TGGTTTGGACACCCTTTACGCTGAACCGGAGAAGATCGCCGGTCTG<br>TCTTTTCGCGGTGCTAAGACCATGGCTGCTAAACTGGAACAGAATA<br>AGGAGGTCGCATACCTGTCTTATCAATTGGCTACCATCAAGACGG<br>ATGTGGAGTTAGAACTTACGTGCGAGCAGCTTGAGGTTCAACAGC<br>CTGCTGCTGAGGAACTGCTGGGTCTTTTTAAGAAATACGAATTTAA<br>GCGTTGGACCGCCGACGTTGAGGCTGGTAAGTGGCTGCAAGCTAA<br>GGGTGCTAAGCCGGCTGCTAAACCGCAAGAAACGAGTGTCGCTGA<br>TGAGGCTCCGGAGGTTACCGCTACCGTTATCTCTTACGATAATTAT<br>GTTACGATTCTGGACGAGGAAACCTTAAAGGCTTGGATCGCTAAA<br>TTAGAGAAGGCTCCTGTTTTCGCTTTCGACACGGAAACGGATTCTC<br>TGGACAATATTAGTGCGAATCTTGTTGGTCTGAGTTTCGCAATTGA<br>ACCGGGTGTTGCTGCTTACATCCCTGTGGCACACGACTACCTGGAC<br>GCTCCGGACCAGATTTCACGTGAACGCGCTCTGGAACTGCTGAAG<br>CCTTTATTAGAGGACGAGAAAGCTTTGAAAGTTGGTCAGAATTTG

	AAGTATGCTCGTGGAATCTTAGCTAATTATGGTATCGAGTTGCGCG<br>GTATCGCTTTCGACACGATGTTGGAATCTTATATCCTGAACTCTGT<br>CGCTGGTCGCCATGACATGGACTCTCTGGCTGAGCGCTGGCTGAA<br>ACATAAGACGATTACCTTCGAGGAAATCGCAGGAAAGGGTAAGAA<br>CCAGCTCACGTTCAATCAAATCGCTCTGGAGGAAGCTGGTCGCTAT<br>GCTGCTGAGGACGCTGACGTTACTCTGCAACTGCACTTGAAGATGT<br>GGCCTGACTTGCAGAAGCATAAGGGTCCACTGAATGTTTTTGAAA<br>ACATTGAGATGCCTTTGGTTCCAGTTCTGTCTCGTATCGAGCGCAA<br>TGGCGTTAAAATTGACCCAAAGGTTTTACATAACCACTCAGAGGA<br>ACTGACGCTGCGCTTAGCCGAATTGGAGAAAAAGGCTCACGAGAT<br>CGCTGGCGAAGAGTTCAATCTGTCATCTACGAAACAACTGCAGAC<br>TATCCTGTTCGAGAAGCAAGGTATCAAGCCATTAAAAAAGACCCC<br>TGGCGGTGCTCCGTCTACCTCTGAGGAAGTTTTGGAGGAGTTAGCT<br>TTGGATTACCCTCTGCCGAAGGTTATCTTGGAATACCGCGGTTTGG<br>CTAAATTGAAGTCTACTTATACGGATAAACTTCCTTTGATGATTAA<br>TCCAAAGACGGGTCGCGTTCACACGTCGTACCATCAAGCTGTTACC<br>GCTACCGGTCGCCTGTCTTCTACGGATCCGAATTTACAGAATATTC<br>CTGTGCGCAATGAGGAGGGCCGCCGCATTCGTCAAGCTTTTATCGC<br>TCCGGAAGACTACGTTATCGTTTCTGCTGATTATTCTCAAAATGAA<br>TTACGTATCATGGCTCACCTGTCTCGCGATAAGGGTCTGTTGACGG<br>CCTTTGCTGAGGGTAAGGACATTCATCGTGCTACCGCTGCTGAGGT<br>TTACGGCCTGCCGTTGGAAACGGTTACGTCTGAACAGCGTCGCTCT<br>GCTAAGCGTATTAATTTCGGCTTAATCTACGGTATGTCTGCGTTTG<br>GCTTAGCTCGTCAGCTGAATATCCCGCGCAAGGAAGCTCAAAAAT<br>ATATGGATCTGTATTTTGAGCGTTACCACGGTGTTTTGGAATACAT<br>GGAGCGTACGCGCGCGCAAGCTAAGGAACAAGGTTATGTGGAAAC<br>CTTGGATGGTCGTCGCTTGTACTTGCCTGACATTAAGTCTTCTAAC<br>GGCGCCCGCCGCGCTGCTGCCGAGCGCGCTGCTATCAATGCTCCG<br>ATGCAAGGTACTGCTGCTGATATTATTAAGCGTGCTATGATCGCTG<br>TGGACGCTTGGCTGCAAGCTGAACAGCCTCGCGTTCGCATGATTAT<br>GCAAGTTCATGACGAGTTGGTTTTCGAGGTGCATAAGGACGACGT<br>GGACGCTGTTGCTAAACAAATCCACCAGTTGATGGAGAATTGCAC<br>GCGCTTAGACGTTCCGCTGCTGGTTGAAGTTGGTTCTGGTGAAAAC<br>TGGGACCAGGCTCACTAATAATGGCTGAAAACTGGGACCAGGCTC<br>ACTAACTCAGATCCTACTAGGTTTAATAAACATCTTTATTTTCATTA<br>CATCTGTGTGTTGGTTTTTTGTGTGGTACCCAATTCGCCCTATAGTG<br>AGTCGTATTACTCACTGGCCGTCGTTTTACAACGTCGTGACTGGGA<br>AAACCCTGGCGTTACCCAACTTAATCGCCTTGCAGCACATCCCCCT<br>TTCGCCAGCTGGCGTAATAGCGAAGAGGCCCGCACCGATCGCCCT<br>TCCCAACAGTTGCGCAGCCTGAATGGCGAATGGGACGCGCCCTGT<br>AGCGGCGCATTAAGCGCGGCGGGTGTGGTGGTTACGCGCAGCGTG<br>ACCGCTACACTTGCCAGCGCCCTAGCGCCCGCTCCTTTCGCTTTCTT<br>CCCTTCCTTTCTCGCCACGTTCGCCGGCTTTCCCCGTCAAGCTCTAA<br>ATCGGGGGCTCCCTTTAGGGTTCCGATTTAGTGCTTTACGGCACCT<br>CGACCCCAAAAAACTTGATTAGGGTGATGGTTCACGTAGTGGGCC<br>ATCGCCCTGATAGACGGTTTTTCGCCCTTTGACGTTGGAGTCCACG

	TTCTTTAATAGTGGACTCTTGTTCCAAACTGGAACAACACTCAACC<br>CTATCTCGGTCTATTCTTTTGATTTATAAGGGATTTTGCCGATTTCG<br>GCCTATTGGTTAAAAAATGAGCTGATTTAACAAAAATTTAACGCG<br>AATTTTAACAAAATATTAACGCTTACAATTTAGGTGGCACTTTTCG<br>GGGAAATGTG<br><br>**Ampicillin resistance cassette-ColE1 origin of replication-hU6 promoter-gRNA sequence-SpCas9 gRNA scaffold-CMV promoter-NLS-mCherry-GSGM- enCas9(D10A)-NLS-linker-PolI5M-stop-SV40 polyA**
p.enCas9-PolI5MΔ	CGCGGAACCCCTATTTGTTTATTTTTCTAAATACATTCAAATATGTA<br>TCCGCTCATGAGACAATAACCCTGATAAATGCTTCAATAATATTGA<br>AAAAGGAAGAGTATGAGTATTCAACATTTCCGTGTCGCCCTTATTC<br>CCTTTTTTGCGGCATTTTGCCTTCCTGTTTTTGCTCACCCAGAAACG<br>CTGGTGAAAGTAAAAGATGCTGAAGATCAGTTGGGTGCACGAGTG<br>GGTTACATCGAACTGGATCTCAACAGCGGTAAGATCCTTGAGAGT<br>TTTCGCCCCGAAGAACGTTTTCCAATGATGAGCACTTTTAAAGTTC<br>TGCTATGTGGCGCGGTATTATCCCGTATTGACGCCGGGCAAGAGC<br>AACTCGGTCGCCGCATACACTATTCTCAGAATGACTTGGTTGAGTA<br>CTCACCAGTCACAGAAAAGCATCTTACGGATGGCATGACAGTAAG<br>AGAATTATGCAGTGCTGCCATAACCATGAGTGATAACACTGCGGC<br>CAACTTACTTCTGACAACGATCGGAGGACCGAAGGAGCTAACCGC<br>TTTTTTGCACAACATGGGGGATCATGTAACTCGCCTTGATCGTTGG<br>GAACCGGAGCTGAATGAAGCCATACCAAACGACGAGCGTGACACC<br>ACGATGCCTGTAGCAATGGCAACAACGTTGCGCAAACTATTAACT<br>GGCGAACTACTTACTCTAGCTTCCCGGCAACAATTAATAGACTGGA<br>TGGAGGCGGATAAAGTTGCAGGACCACTTCTGCGCTCGGCCCTTCC<br>GGCTGGCTGGTTTATTGCTGATAAATCTGGAGCCGGTGAGCGTGGT<br>TCTCGCGGTATCATTGCAGCACTGGGGCCAGATGGTAAGCCCTCCC<br>GTATCGTAGTTATCTACACGACGGGGAGTCAGGCAACTATGGATG<br>AACGAAATAGACAGATCGCTGAGATAGGTGCCTCACTGATTAAGC<br>ATTGGTAACTGTCAGACCAAGTTTACTCATATATACTTTAGATTGA<br>TTTAAAACTTCATTTTTAATTTAAAAGGATCTAGGTGAAGATCCTT<br>TTTGATAATCTCATGACCAAAATCCCTTAACGTGAGTTTTCGTTCC<br>ACTGAGCGTCAGACCCCGTAGAAAAGATCAAAGGATCTTCTTGAG<br>ATCCTTTTTTTCTGCGCGTAATCTGCTGCTTGCAAACAAAAAAACC<br>ACCGCTACCAGCGGTGGTTTGTTTGCCGGATCAAGAGCTACCAACT<br>CTTTTTCCGAAGGTAACTGGCTTCAGCAGAGCGCAGATACCAAAT<br>ACTGTCCTTCTAGTGTAGCCGTAGTTAGGCCACCACTTCAAGAACT<br>CTGTAGCACCGCCTACATACCTCGCTCTGCTAATCCTGTTACCAGT<br>GGCTGCTGCCAGTGGCGATAAGTCGTGTCTTACCGGGTTGGACTCA<br>AGACGATAGTTACCGGATAAGGCGCAGCGGTCGGGCTGAACGGGG<br>GGTTCGTGCACACAGCCCAGCTTGGAGCGAACGACCTACACCGAA<br>CTGAGATACCTACAGCGTGAGCTATGAGAAAGCGCCACGCTTCCC<br>GAAGGGAGAAAGGCGGACAGGTATCCGGTAAGCGGCAGGGTCGG

	AACAGGAGAGCGCACGAGGGAGCTTCCAGGGGGAAACGCCTGGT<br>ATCTTTATAGTCCTGTCGGGTTTCGCCACCTCTGACTTGAGCGTCG<br>ATTTTTGTGATGCTCGTCAGGGGGGCGGAGCCTATGGAAAAACGC<br>CAGCAACGCGGCCTTTTTACGGTTCCTGGCCTTTTGCTGGCCTTTTG<br>CTCACATGTTCTTTCCTGCGTTATCCCCTGATTCTGTGGATAACCGT<br>ATTACCGCCTTTGAGTGAGCTGATACCGCTCGCCGCAGCCGAACG<br>ACCGAGCGCAGCGAGTCAGTGAGCGAGGAAGCGGAAGAGCGCCC<br>AATACGCAAACCGCCTCTCCCCGCGCGTTGGCCGATTCATTAATGC<br>AGCTGGCACGACAGGTTTCCCGACTGGAAAGCGGGCAGTGAGCGC<br>AACGCAATTAATGTGAGTTAGCTCACTCATTAGGCACCCCAGGCTT<br>TACACTTTATGCTTCCGGCTCGTATGTTGTGTGGAATTGTGAGCGG<br>ATAACAATTTCACACAGGAAACAGCTATGACCATGATTACGCCAA<br>GCGCGCAATTAACCCTCACTAAAGGGAACAAAAGCTGGAGCTCCA<br>CCGCGGTGGCGGCCGCTCTTAAGGGGTGCAGCGGCCTCCGCGCCG<br>GGTTTTGGCGCCTCGATCCAAGGTCGGGCAGGAAGAGGGCCTATT<br>TCCCATGATTCCTTCATATTTGCATATACGATACAAGGCTGTTAGA<br>GAGATAATTAGAATTAATTTGACTGTAAACACAAAGATATTAGTA<br>CAAAATACGTGACGTAGAAAGTAATAATTTCTTGGGTAGTTTGCA<br>GTTTTAAAATTATGTTTTAAAATGGACTATCATATGCTTACCGTAA<br>CTTGAAAGTATTTCGATTTCTTGGCTTTATATATCTTGTGGAAAGG<br>ACGAAACACC-[gRNA SEQUENCE]-<br>GTTTTAGAGCTAGAAATAGCAAGTTAAAATAAGGCTAGTCCGTTA<br>TCAACTTGAAAAAGTGGCACCGAGTCGGTGCTTTTTTCCGCGGCCT<br>CTAGACTCGAGGCGTTGACATTGATTATTGACTAGTTATTAATAGT<br>AATCAATTACGGGGTCATTAGTTCATAGCCCATATATGGAGTTCCG<br>CGTTACATAACTTACGGTAAATGGCCCGCCTGGCTGACCGCCCAAC<br>GACCCCCGCCCATTGACGTCAATAATGACGTATGTTCCCATAGTAA<br>CGCCAATAGGGACTTTCCATTGACGTCAATGGGTGGAGTATTTACG<br>GTAAACTGCCCACTTGGCAGTACATCAAGTGTATCATATGCCAAGT<br>ACGCCCCCTATTGACGTCAATGACGGTAAATGGCCCGCCTGGCATT<br>ATGCCCAGTACATGACCTTATGGGACTTTCCTACTTGGCAGTACAT<br>CTACGTATTAGTCATCGCTATTACCATGGTGATGCGGTTTTGGCAG<br>TACATCAATGGGCGTGGATAGCGGTTTGACTCACGGGGATTTCCA<br>AGTCTCCACCCCATTGACGTCAATGGGAGTTTGTTTTGGCACCAAA<br>ATCAACGGGACTTTCCAAAATGTCGTAACAACTCCGCCCCATTGAC<br>GCAAATGGGCGGTAGGCGTGTACGGTGGGAGGTCTATATAAGCAG<br>AGCTCTCTGGCTAACTACCGGTGCCACCATGGCCCCAAAGAAGAA<br>GCGGAAGGTCGGTATCCACGGAGTCCCAGCAGCCGTGAGCAAGGG<br>CGAGGAGGATAACATGGCCATCATCAAGGAGTTCATGCGCTTCAA<br>GGTGCACATGGAGGGCTCCGTGAACGGCCACGAGTTCGAGATCGA<br>GGGCGAGGGCGAGGGCCGCCCCTACGAGGGCACCCAGACCGCCA<br>AGCTGAAGGTGACCAAGGGTGGCCCCCTGCCCTTCGCCTGGGACA<br>TCCTGTCCCCTCAGTTCATGTACGGCTCCAAGGCCTACGTGAAGCA<br>CCCCGCCGACATCCCCGACTACTTGAAGCTGTCCTTCCCCGAGGGC<br>TTCAAGTGGGAGCGCGTGATGAACTTCGAGGACGGCGGCGTGGTG<br>ACCGTGACCCAGGACTCCTCCCTGCAGGACGGCGAGTTCATCTAC

		AAGGTGAAGCTGCGCGGCACCAACTTCCCCTCCGACGGCCCCGTA<br>ATGCAGAAGAAGACCATGGGCTGGGAGGCCTCCTCCGAGCGGATG<br>TACCCCGAGGACGGCGCCCTGAAGGGCGAGATCAAGCAGAGGCTG<br>AAGCTGAAGGACGGCGGCCACTACGACGCTGAGGTCAAGACCACC<br>TACAAGGCCAAGAAGCCCGTGCAGCTGCCCGGCGCCTACAACGTC<br>AACATCAAGTTGGACATCACCTCCCACAACGAGGACTACACCATC<br>GTGGAACAGTACGAACGCGCCGAGGGCCGCCACTCCACCGGCGGC<br>ATGGACGAGCTGTACAAGGGATCCGGTATGGACAAGAAGTACAGC<br>ATCGGCCTGGCCATCGGCACCAACTCTGTGGGCTGGGCCGTGATC<br>ACCGACGAGTACAAGGTGCCCAGCAAGAAATTCAAGGTGCTGGGC<br>AACACCGACCGGCACAGCATCAAGAAGAACCTGATCGGCGCCCTG<br>CTGTTCGACAGCGGAGAAACAGCCGAGGCCACCCGGCTGAAGAGA<br>ACCGCCAGAAGAAGATACACCAGACGGAAGAACCGGATCTGCTAT<br>CTGCAAGAGATCTTCAGCAACGAGATGGCCAAGGTGGACGACAGC<br>TTCTTCCACAGACTGGAAGAGTCCTTCCTGGTGGAAGAGGATAAG<br>AAGCACGAGCGGCACCCCATCTTCGGCAACATCGTGGACGAGGTG<br>GCCTACCACGAGAAGTACCCCACCATCTACCACCTGAGAAAGAAA<br>CTGGTGGACAGCACCGACAAGGCCGACCTGCGGCTGATCTATCTG<br>GCCCTGGCCCACATGATCAAGTTCCGGGGCCACTTCCTGATCGAGG<br>GCGACCTGAACCCCGACAACAGCGACGTGGACAAGCTGTTCATCC<br>AGCTGGTGCAGACCTACAACCAGCTGTTCGAGGAAAACCCCATCA<br>ACGCCAGCGGCGTGGACGCCAAGGCCATCCTGTCTGCCAGACTGA<br>GCAAGAGCAGACGGCTGGAAAATCTGATCGCCCAGCTGCCCGGCG<br>AGAAGAAGAATGGCCTGTTCGGCAACCTGATTGCCCTGAGCCTGG<br>GCCTGACCCCCAACTTCAAGAGCAACTTCGACCTGGCCGAGGATG<br>CCAAACTGCAGCTGAGCAAGGACACCTACGACGACGACCTGGACA<br>ACCTGCTGGCCCAGATCGGCGACCAGTACGCCGACCTGTTTCTGGC<br>CGCCAAGAACCTGTCCGACGCCATCCTGCTGAGCGACATCCTGAG<br>AGTGAACACCGAGATCACCAAGGCCCCCCTGAGCGCCTCTATGAT<br>CAAGAGATACGACGAGCACCACCAGGACCTGACCCTGCTGAAAGC<br>TCTCGTGCGGCAGCAGCTGCCTGAGAAGTACAAAGAGATTTTCTTC<br>GACCAGAGCAAGAACGGCTACGCCGGCTACATCGATGGCGGAGCC<br>AGCCAGGAAGAGTTCTACAAGTTCATCAAGCCCATCCTGGAAAAG<br>ATGGACGGCACCGAGGAACTGCTCGTGAAGCTGAACAGAGAGGA<br>CCTGCTGCGGAAGCAGCGGACCTTCGACAACGGCAGCATCCCCCA<br>CCAGATCCACCTGGGAGAGCTGCACGCCATTCTGCGGCGGCAGGA<br>AGATTTTTACCCATTCCTGAAGGACAACCGGGAAAAGATCGAGAA<br>GATCCTGACCTTCCGCATCCCCTACTACGTGGGCCCTCTGGCCAGG<br>GGAAACAGCAGATTCGCCTGGATGACCAGAAAGAGCGAGGAAAC<br>CATCACCCCCTGGAACTTCGAGGAAGTGGTGGACAAGGGCGCCAG<br>CGCCCAGAGCTTCATCGAGCGGATGACCAACTTCGATAAGAACCT<br>GCCCAACGAGAAGGTGCTGCCCAAGCACAGCCTGCTGTACGAGTA<br>CTTCACCGTGTACAACGAGCTGACCAAAGTGAAATACGTGACCGA<br>GGGAATGAGAAAGCCCGCCTTCCTGAGCGGCGAGCAGAAAAAAG<br>CCATCGTGGACCTGCTGTTCAAGACCAACCGGAAAGTGACCGTGA<br>AGCAGCTGAAAGAGGACTACTTCAAGAAAATCGAGTGCTTCGACT

		CCGTGGAAATCTCCGGCGTGGAAGATCGGTTCAACGCCTCCCTGG<br>GCACATACCACGATCTGCTGAAAATTATCAAGGACAAGGACTTCC<br>TGGACAATGAGGAAAACGAGGACATTCTGGAAGATATCGTGCTGA<br>CCCTGACACTGTTTGAGGACAGAGAGATGATCGAGGAACGGCTGA<br>AAACCTATGCCCACCTGTTCGACGACAAAGTGATGAAGCAGCTGA<br>AGCGGCGGAGATACACCGGCTGGGGCAGGCTGAGCCGGAAGCTG<br>ATCAACGGCATCCGGGACAAGCAGTCCGGCAAGACAATCCTGGAT<br>TTCCTGAAGTCCGACGGCTTCGCCAACAGAAACTTCATGCAGCTGA<br>TCCACGACGACAGCCTGACCTTTAAAGAGGACATCCAGAAAGCCC<br>AGGTGTCCGGCCAGGGCGATAGCCTGCACGAGCACATTGCCAATC<br>TGGCCGGCAGCCCCGCCATTAAGAAGGGCATCCTGCAGACAGTGA<br>AGGTGGTGGACGAGCTCGTGAAAGTGATGGGCCGGCACAAGCCCG<br>AGAACATCGTGATCGAAATGGCCAGAGAGAACCAGACCACCCAG<br>AAGGGACAGAAGAACAGCCGCGAGAGAATGAAGCGGATCGAAGA<br>GGGCATCAAAGAGCTGGGCAGCCAGATCCTGAAAGAACACCCCGT<br>GGAAAACACCCAGCTGCAGAACGAGAAGCTGTACCTGTACTACCT<br>GCAGAATGGGCGGGATATGTACGTGGACCAGGAACTGGACATCAA<br>CCGGCTGTCCGACTACGATGTGGACCATATCGTGCCTCAGAGCTTT<br>CTGGCAGACGACTCCATCGATAACAAAGTGCTGACTCGGAGCGAC<br>AAGAACCGGGGCAAGAGCGACAACGTGCCCTCCGAAGAGGTCGT<br>GAAGAAGATGAAGAACTACTGGCGCCAGCTGCTGAATGCCAAGCT<br>GATTACCCAGAGGAAGTTCGACAATCTGACCAAGGCCGAGAGAGG<br>CGGCCTGAGCGAACTGGATAAGGCCGGCTTCATCAAGAGACAGCT<br>GGTGGAAACCCGGCAGATCACAAAGCACGTGGCACAGATCCTGGA<br>CTCCCGGATGAACACTAAGTACGACGAGAACGACAAACTGATCCG<br>GGAAGTGAAAGTGATCACCCTGAAGTCCAAGCTGGTGTCCGATTT<br>CCGGAAGGATTTCCAGTTTTACAAAGTGCGCGAGATCAACAACTA<br>CCACCACGCCCACGACGCCTACCTGAACGCCGTCGTGGGAACCGC<br>CCTGATCAAAAAGTACCCTGCGCTGGAAAGCGAGTTCGTGTACGG<br>CGACTACAAGGTGTACGACGTGCGGAAGATGATCGCCAAGAGCGA<br>GCAGGAAATCGGCAAGGCTACCGCCAAGTACTTCTTCTACAGCAA<br>CATCATGAACTTTTTCAAGACCGAGATTACCCTGGCCAACGGCGA<br>GATCCGGAAGGCGCCTCTGATCGAGACAAACGGCGAAACAGGCG<br>AGATCGTGTGGGATAAGGGCCGGGACTTTGCCACCGTGCGGAAAG<br>TGCTGTCTATGCCCCAAGTGAATATCGTGAAAAAGACCGAGGTGC<br>AGACAGGCGGCTTCAGCAAAGAGTCTATCCTGCCCAAGAGGAACA<br>GCGACAAGCTGATCGCCAGAAAGAAGGACTGGGACCCTAAGAAG<br>TACGGCGGCTTCGACAGCCCCACCGTGGCCTATTCTGTGCTGGTGG<br>TGGCCAAAGTGGAAAAGGGCAAGTCCAAGAAACTGAAGAGTGTG<br>AAAGAGCTGCTGGGGATCACCATCATGGAAAGAAGCAGCTTCGAG<br>AAGAATCCCATCGACTTTCTGGAAGCCAAGGGCTACAAAGAAGTG<br>AAAAAGGACCTGATCATCAAGCTGCCTAAGTACTCCCTGTTCGAG<br>CTGGAAAACGGCCGGAAGAGAATGCTGGCCTCTGCCGGCGAACTG<br>CAGAAGGGAAACGAACTGGCCCTGCCCTCCAAATATGTGAACTTC<br>CTGTACCTGGCCAGCCACTATGAGAAGCTGAAGGGCTCCCCCGAG<br>GATAATGAGCAGAAACAGCTGTTTGTGGAACAGCACAAACACTAC

	CTGGACGAGATCATCGAGCAGATCAGCGAGTTCTCCAAGAGAGTG<br>ATCCTGGCCGACGCTAATCTGGACAAGGTGCTGAGCGCCTACAAC<br>AAGCACAGAGACAAGCCTATCAGAGAGCAGGCCGAGAATATCATC<br>CACCTGTTTACCCTGACCAATCTGGGAGCCCCTGCCGCCTTCAAGT<br>ACTTTGACACCACCATCGACCGGAAGAGGTACACCAGCACCAAAG<br>AGGTGCTGGACGCCACCCTGATCCACCAGAGCATCACCGGCCTGT<br>ACGAGACACGGATCGACCTGTCTCAGCTGGGAGGCGACGCCTATC<br>CCTATGACGTGCCCGATTATGCCAGCCTGGGCAGCGGCTCCCCCAA<br>GAAAAAACGCAAGGTGGAAGATCCTAAGAAAAAGCGGAAAGGTT<br>CTAGTGAAACCCCGGGAACAAGTGAGTCGGCCACCCCTGAAGGTG<br>GATCAGGGGGTAGCGGATCCTCTTACGATAATTATGTTACGATTCT<br>GGACGAGGAAACCTTAAAGGCTTGGATCGCTAAATTAGAGAAGGC<br>TCCTGTTTTCGCTTTCGACACGGAAACGGATTCTCTGGACAATATT<br>AGTGCGAATCTTGTTGGTCTGAGTTTCGCAATTGAACCGGGTGTTG<br>CTGCTTACATCCCTGTGGCACACGACTACCTGGACGCTCCGGACCA<br>GATTTCACGTGAACGCGCTCTGGAACTGCTGAAGCCTTTATTAGAG<br>GACGAGAAAGCTTTGAAAGTTGGTCAGAATTTGAAGTATGCTCGT<br>GGAATCTTAGCTAATTATGGTATCGAGTTGCGCGGTATCGCTTTCG<br>ACACGATGTTGGAATCTTATATCCTGAACTCTGTCGCTGGTCGCCA<br>TGACATGGACTCTCTGGCTGAGCGCTGGCTGAAACATAAGACGAT<br>TACCTTCGAGGAAATCGCAGGAAAGGGTAAGAACCAGCTCACGTT<br>CAATCAAATCGCTCTGGAGGAAGCTGGTCGCTATGCTGCTGAGGA<br>CGCTGACGTTACTCTGCAACTGCACTTGAAGATGTGGCCTGACTTG<br>CAGAAGCATAAGGGTCCACTGAATGTTTTTGAAAACATTGAGATG<br>CCTTTGGTTCCAGTTCTGTCTCGTATCGAGCGCAATGGCGTTAAAA<br>TTGACCCAAAGGTTTTACATAACCACTCAGAGGAACTGACGCTGC<br>GCTTAGCCGAATTGGAGAAAAAGGCTCACGAGATCGCTGGCGAAG<br>AGTTCAATCTGTCATCTACGAAACAACTGCAGACTATCCTGTTCGA<br>GAAGCAAGGTATCAAGCCATTAAAAAAGACCCCTGGCGGTGCTCC<br>GTCTACCTCTGAGGAAGTTTTGGAGGAGTTAGCTTTGGATTACCCT<br>CTGCCGAAGGTTATCTTGGAATACCGCGGTTTGGCTAAATTGAAGT<br>CTACTTATACGGATAAACTTCCTTTGATGATTAATCCAAAGACGGG<br>TCGCGTTCACACGTCGTACCATCAAGCTGTTACCGCTACCGGTCGC<br>CTGTCTTCTACGGATCCGAATTTACAGAATATTCCTGTGCGCAATG<br>AGGAGGGCCGCCGCATTCGTCAAGCTTTTATCGCTCCGGAAGACT<br>ACGTTATCGTTTCTGCTGATTATTCTCAAAATGAATTACGTATCAT<br>GGCTCACCTGTCTCGCGATAAGGGTCTGTTGACGGCCTTTGCTGAG<br>GGTAAGGACATTCATCGTGCTACCGCTGCTGAGGTTTACGGCCTGC<br>CGTTGGAAACGGTTACGTCTGAACAGCGTCGCTCTGCTAAGCGTAT<br>TAATTTCGGCTTAATCTACGGTATGTCTGCGTTTGGCTTAGCTCGTC<br>AGCTGAATATCCCGCGCAAGGAAGCTCAAAAATATATGGATCTGT<br>ATTTTGAGCGTTACCACGGTGTTTTGGAATACATGGAGCGTACGCG<br>CGCGCAAGCTAAGGAACAAGGTTATGTGGAAACCTTGGATGGTCG<br>TCGCTTGTACTTGCCTGACATTAAGTCTTCTAACGGCGCCCGCCGC<br>GCTGCTGCCGAGCGCGCTGCTATCAATGCTCCGATGCAAGGTACTG<br>CTGCTGATATTATTAAGCGTGCTATGATCGCTGTGGACGCTTGGCT

	GCAAGCTGAACAGCCTCGCGTTCGCATGATTATGCAAGTTCATGAC<br>GAGTTGGTTTTCGAGGTGCATAAGGACGACGTGGACGCTGTTGCT<br>AAACAAATCCACCAGTTGATGGAGAATTGCACGCGCTTAGACGTT<br>CCGCTGCTGGTTGAAGTTGGTTCTGGTGAAAACTGGGACCAGGCTC<br>ACTAATGGCTGAAAACTGGGACCAGGCTCACTAACTCAGATCCTA<br>CTAGGTTTAATAAACATCTTTATTTTCATTACATCTGTGTGTTGGTT<br>TTTTGTGTGGTACCCAATTCGCCCTATAGTGAGTCGTATTACTCACT<br>GGCCGTCGTTTTACAACGTCGTGACTGGGAAAACCCTGGCGTTACC<br>CAACTTAATCGCCTTGCAGCACATCCCCCTTTCGCCAGCTGGCGTA<br>ATAGCGAAGAGGCCCGCACCGATCGCCCTTCCCAACAGTTGCGCA<br>GCCTGAATGGCGAATGGGACGCGCCCTGTAGCGGCGCATTAAGCG<br>CGGCGGGTGTGGTGGTTACGCGCAGCGTGACCGCTACACTTGCCA<br>GCGCCCTAGCGCCCGCTCCTTTCGCTTTCTTCCCTTCCTTTCTCGCC<br>ACGTTCGCCGGCTTTCCCCGTCAAGCTCTAAATCGGGGGCTCCCTT<br>TAGGGTTCCGATTTAGTGCTTTACGGCACCTCGACCCCAAAAAACT<br>TGATTAGGGTGATGGTTCACGTAGTGGGCCATCGCCCTGATAGAC<br>GGTTTTTCGCCCTTTGACGTTGGAGTCCACGTTCTTTAATAGTGGA<br>CTCTTGTTCCAAACTGGAACAACACTCAACCCTATCTCGGTCTATT<br>CTTTTGATTTATAAGGGATTTTGCCGATTTCGGCCTATTGGTTAAA<br>AAATGAGCTGATTTAACAAAAATTTAACGCGAATTTTAACAAAAT<br>ATTAACGCTTACAATTTAGGTGGCACTTTTCGGGGAAATGTG<br><br>**Ampicillin resistance cassette-ColE1 origin of replication-hU6 promoter-gRNA sequence-SpCas9 gRNA scaffold-CMV promoter-NLS-mCherry-GSGM- enCas9(D10A)-NLS-linker-PolI5MΔ-stop-SV40 polyA**
p.SuperFi-nCas9-PolI5MΔ	CGCGGAACCCCTATTTGTTTATTTTTCTAAATACATTCAAATATGTA<br>TCCGCTCATGAGACAATAACCCTGATAAATGCTTCAATAATATTGA<br>AAAAGGAAGAGTATGAGTATTCAACATTTCCGTGTCGCCCTTATTC<br>CCTTTTTTGCGGCATTTTGCCTTCCTGTTTTTGCTCACCCAGAAACG<br>CTGGTGAAAGTAAAAGATGCTGAAGATCAGTTGGGTGCACGAGTG<br>GGTTACATCGAACTGGATCTCAACAGCGGTAAGATCCTTGAGAGT<br>TTTCGCCCCGAAGAACGTTTTCCAATGATGAGCACTTTTAAAGTTC<br>TGCTATGTGGCGCGGTATTATCCCGTATTGACGCCGGGCAAGAGC<br>AACTCGGTCGCCGCATACACTATTCTCAGAATGACTTGGTTGAGTA<br>CTCACCAGTCACAGAAAAGCATCTTACGGATGGCATGACAGTAAG<br>AGAATTATGCAGTGCTGCCATAACCATGAGTGATAACACTGCGGC<br>CAACTTACTTCTGACAACGATCGGAGGACCGAAGGAGCTAACCGC<br>TTTTTTGCACAACATGGGGGATCATGTAACTCGCCTTGATCGTTGG<br>GAACCGGAGCTGAATGAAGCCATACCAAACGACGAGCGTGACACC<br>ACGATGCCTGTAGCAATGGCAACAACGTTGCGCAAACTATTAACT<br>GGCGAACTACTTACTCTAGCTTCCCGGCAACAATTAATAGACTGGA<br>TGGAGGCGGATAAAGTTGCAGGACCACTTCTGCGCTCGGCCCTTCC<br>GGCTGGCTGGTTTATTGCTGATAAATCTGGAGCCGGTGAGCGTGGT<br>TCTCGCGGTATCATTGCAGCACTGGGGCCAGATGGTAAGCCCTCCC

	GTATCGTAGTTATCTACACGACGGGGAGTCAGGCAACTATGGATG<br>AACGAAATAGACAGATCGCTGAGATAGGTGCCTCACTGATTAAGC<br>ATTGGTAACTGTCAGACCAAGTTTACTCATATATACTTTAGATTGA<br>TTTAAAACTTCATTTTTAATTTAAAAGGATCTAGGTGAAGATCCTT<br>TTTGATAATCTCATGACCAAAATCCCTTAACGTGAGTTTTCGTTCC<br>ACTGAGCGTCAGACCCCGTAGAAAAGATCAAAGGATCTTCTTGAG<br>ATCCTTTTTTTCTGCGCGTAATCTGCTGCTTGCAAACAAAAAAACC<br>ACCGCTACCAGCGGTGGTTTGTTTGCCGGATCAAGAGCTACCAACT<br>CTTTTTCCGAAGGTAACTGGCTTCAGCAGAGCGCAGATACCAAAT<br>ACTGTCCTTCTAGTGTAGCCGTAGTTAGGCCACCACTTCAAGAACT<br>CTGTAGCACCGCCTACATACCTCGCTCTGCTAATCCTGTTACCAGT<br>GGCTGCTGCCAGTGGCGATAAGTCGTGTCTTACCGGGTTGGACTCA<br>AGACGATAGTTACCGGATAAGGCGCAGCGGTCGGGCTGAACGGGG<br>GGTTCGTGCACACAGCCCAGCTTGGAGCGAACGACCTACACCGAA<br>CTGAGATACCTACAGCGTGAGCTATGAGAAAGCGCCACGCTTCCC<br>GAAGGGAGAAAGGCGGACAGGTATCCGGTAAGCGGCAGGGTCGG<br>AACAGGAGAGCGCACGAGGGAGCTTCCAGGGGGAAACGCCTGGT<br>ATCTTTATAGTCCTGTCGGGTTTCGCCACCTCTGACTTGAGCGTCG<br>ATTTTTGTGATGCTCGTCAGGGGGGCGGAGCCTATGGAAAAACGC<br>CAGCAACGCGGCCTTTTTACGGTTCCTGGCCTTTTGCTGGCCTTTTG<br>CTCACATGTTCTTTCCTGCGTTATCCCCTGATTCTGTGGATAACCGT<br>ATTACCGCCTTTGAGTGAGCTGATACCGCTCGCCGCAGCCGAACG<br>ACCGAGCGCAGCGAGTCAGTGAGCGAGGAAGCGGAAGAGCGCCC<br>AATACGCAAACCGCCTCTCCCCGCGCGTTGGCCGATTCATTAATGC<br>AGCTGGCACGACAGGTTTCCCGACTGGAAAGCGGGCAGTGAGCGC<br>AACGCAATTAATGTGAGTTAGCTCACTCATTAGGCACCCCAGGCTT<br>TACACTTTATGCTTCCGGCTCGTATGTTGTGTGGAATTGTGAGCGG<br>ATAACAATTTCACACAGGAAACAGCTATGACCATGATTACGCCAA<br>GCGCGCAATTAACCCTCACTAAAGGGAACAAAAGCTGGAGCTCCA<br>CCGCGGTGGCGGCCGCTCTTAAGGGGTGCAGCGGCCTCCGCGCCG<br>GGTTTTGGCGCCTCGATCCAAGGTCGGGCAGGAAGAGGGCCTATT<br>TCCCATGATTCCTTCATATTTGCATATACGATACAAGGCTGTTAGA<br>GAGATAATTAGAATTAATTTGACTGTAAACACAAAGATATTAGTA<br>CAAAATACGTGACGTAGAAAGTAATAATTTCTTGGGTAGTTTGCA<br>GTTTTAAAATTATGTTTTAAAATGGACTATCATATGCTTACCGTAA<br>CTTGAAAGTATTTCGATTTCTTGGCTTTATATATCTTGTGGAAAGG<br>ACGAAACACC-[gRNA SEQUENCE]-<br>GTTTTAGAGCTAGAAATAGCAAGTTAAAATAAGGCTAGTCCGTTA<br>TCAACTTGAAAAAGTGGCACCGAGTCGGTGCTTTTTTCCGCGGCCT<br>CTAGACTCGAGGCGTTGACATTGATTATTGACTAGTTATTAATAGT<br>AATCAATTACGGGGTCATTAGTTCATAGCCCATATATGGAGTTCCG<br>CGTTACATAACTTACGGTAAATGGCCCGCCTGGCTGACCGCCCAAC<br>GACCCCCGCCCATTGACGTCAATAATGACGTATGTTCCCATAGTAA<br>CGCCAATAGGGACTTTCCATTGACGTCAATGGGTGGAGTATTTACG<br>GTAAACTGCCCACTTGGCAGTACATCAAGTGTATCATATGCCAAGT<br>ACGCCCCCTATTGACGTCAATGACGGTAAATGGCCCGCCTGGCATT

	ATGCCCAGTACATGACCTTATGGGACTTTCCTACTTGGCAGTACAT<br>CTACGTATTAGTCATCGCTATTACCATGGTGATGCGGTTTTGGCAG<br>TACATCAATGGGCGTGGATAGCGGTTTGACTCACGGGGATTTCCA<br>AGTCTCCACCCCATTGACGTCAATGGGAGTTTGTTTTGGCACCAAA<br>ATCAACGGGACTTTCCAAAATGTCGTAACAACTCCGCCCCATTGAC<br>GCAAATGGGCGGTAGGCGTGTACGGTGGGAGGTCTATATAAGCAG<br>AGCTCTCTGGCTAACTACCGGTGCCACCATGGCCCCAAAGAAGAA<br>GCGGAAGGTCGGTATCCACGGAGTCCCAGCAGCCGTGAGCAAGGG<br>CGAGGAGGATAACATGGCCATCATCAAGGAGTTCATGCGCTTCAA<br>GGTGCACATGGAGGGCTCCGTGAACGGCCACGAGTTCGAGATCGA<br>GGGCGAGGGCGAGGGCCGCCCCTACGAGGGCACCCAGACCGCCA<br>AGCTGAAGGTGACCAAGGGTGGCCCCCTGCCCTTCGCCTGGGACA<br>TCCTGTCCCCTCAGTTCATGTACGGCTCCAAGGCCTACGTGAAGCA<br>CCCCGCCGACATCCCCGACTACTTGAAGCTGTCCTTCCCCGAGGGC<br>TTCAAGTGGGAGCGCGTGATGAACTTCGAGGACGGCGGCGTGGTG<br>ACCGTGACCCAGGACTCCTCCCTGCAGGACGGCGAGTTCATCTAC<br>AAGGTGAAGCTGCGCGGCACCAACTTCCCCTCCGACGGCCCCGTA<br>ATGCAGAAGAAGACCATGGGCTGGGAGGCCTCCTCCGAGCGGATG<br>TACCCCGAGGACGGCGCCCTGAAGGGCGAGATCAAGCAGAGGCTG<br>AAGCTGAAGGACGGCGGCCACTACGACGCTGAGGTCAAGACCACC<br>TACAAGGCCAAGAAGCCCGTGCAGCTGCCCGGCGCCTACAACGTC<br>AACATCAAGTTGGACATCACCTCCCACAACGAGGACTACACCATC<br>GTGGAACAGTACGAACGCGCCGAGGGCCGCCACTCCACCGGCGGC<br>ATGGACGAGCTGTACAAGGGATCCGGTATGGACAAGAAGTACAGC<br>ATCGGCCTGGCCATCGGCACCAACTCTGTGGGCTGGGCCGTGATC<br>ACCGACGAGTACAAGGTGCCCAGCAAGAAATTCAAGGTGCTGGGC<br>AACACCGACCGGCACAGCATCAAGAAGAACCTGATCGGCGCCCTG<br>CTGTTCGACAGCGGAGAAACAGCCGAGGCCACCCGGCTGAAGAGA<br>ACCGCCAGAAGAAGATACACCAGACGGAAGAACCGGATCTGCTAT<br>CTGCAAGAGATCTTCAGCAACGAGATGGCCAAGGTGGACGACAGC<br>TTCTTCCACAGACTGGAAGAGTCCTTCCTGGTGGAAGAGGATAAG<br>AAGCACGAGCGGCACCCCATCTTCGGCAACATCGTGGACGAGGTG<br>GCCTACCACGAGAAGTACCCCACCATCTACCACCTGAGAAAGAAA<br>CTGGTGGACAGCACCGACAAGGCCGACCTGCGGCTGATCTATCTG<br>GCCCTGGCCCACATGATCAAGTTCCGGGGCCACTTCCTGATCGAGG<br>GCGACCTGAACCCCGACAACAGCGACGTGGACAAGCTGTTCATCC<br>AGCTGGTGCAGACCTACAACCAGCTGTTCGAGGAAAACCCCATCA<br>ACGCCAGCGGCGTGGACGCCAAGGCCATCCTGTCTGCCAGACTGA<br>GCAAGAGCAGACGGCTGGAAAATCTGATCGCCCAGCTGCCCGGCG<br>AGAAGAAGAATGGCCTGTTCGGCAACCTGATTGCCCTGAGCCTGG<br>GCCTGACCCCCAACTTCAAGAGCAACTTCGACCTGGCCGAGGATG<br>CCAAACTGCAGCTGAGCAAGGACACCTACGACGACGACCTGGACA<br>ACCTGCTGGCCCAGATCGGCGACCAGTACGCCGACCTGTTTCTGGC<br>CGCCAAGAACCTGTCCGACGCCATCCTGCTGAGCGACATCCTGAG<br>AGTGAACACCGAGATCACCAAGGCCCCCCTGAGCGCCTCTATGAT<br>CAAGAGATACGACGAGCACCACCAGGACCTGACCCTGCTGAAAGC

	TCTCGTGCGGCAGCAGCTGCCTGAGAAGTACAAAGAGATTTTCTTC<br>GACCAGAGCAAGAACGGCTACGCCGGCTACATCGATGGCGGAGCC<br>AGCCAGGAAGAGTTCTACAAGTTCATCAAGCCCATCCTGGAAAAG<br>ATGGACGGCACCGAGGAACTGCTCGTGAAGCTGAACAGAGAGGA<br>CCTGCTGCGGAAGCAGCGGACCTTCGACAACGGCAGCATCCCCCA<br>CCAGATCCACCTGGGAGAGCTGCACGCCATTCTGCGGCGGCAGGA<br>AGATTTTTACCCATTCCTGAAGGACAACCGGGAAAAGATCGAGAA<br>GATCCTGACCTTCCGCATCCCCTACTACGTGGGCCCTCTGGCCAGG<br>GGAAACAGCAGATTCGCCTGGATGACCAGAAAGAGCGAGGAAAC<br>CATCACCCCCTGGAACTTCGAGGAAGTGGTGGACAAGGGCGCCAG<br>CGCCCAGAGCTTCATCGAGCGGATGACCAACTTCGATAAGAACCT<br>GCCCAACGAGAAGGTGCTGCCCAAGCACAGCCTGCTGTACGAGTA<br>CTTCACCGTGTACAACGAGCTGACCAAAGTGAAATACGTGACCGA<br>GGGAATGAGAAAGCCCGCCTTCCTGAGCGGCGAGCAGAAAAAAG<br>CCATCGTGGACCTGCTGTTCAAGACCAACCGGAAAGTGACCGTGA<br>AGCAGCTGAAAGAGGACTACTTCAAGAAAATCGAGTGCTTCGACT<br>CCGTGGAAATCTCCGGCGTGGAAGATCGGTTCAACGCCTCCCTGG<br>GCACATACCACGATCTGCTGAAAATTATCAAGGACAAGGACTTCC<br>TGGACAATGAGGAAAACGAGGACATTCTGGAAGATATCGTGCTGA<br>CCCTGACACTGTTTGAGGACAGAGAGATGATCGAGGAACGGCTGA<br>AAACCTATGCCCACCTGTTCGACGACAAAGTGATGAAGCAGCTGA<br>AGCGGCGGAGATACACCGGCTGGGGCAGGCTGAGCCGGAAGCTG<br>ATCAACGGCATCCGGGACAAGCAGTCCGGCAAGACAATCCTGGAT<br>TTCCTGAAGTCCGACGGCTTCGCCAACAGAAACTTCATGCAGCTGA<br>TCCACGACGACAGCCTGACCTTTAAAGAGGACATCCAGAAAGCCC<br>AGGTGTCCGGCCAGGGCGATAGCCTGCACGAGCACATTGCCAATC<br>TGGCCGGCAGCCCCGCCATTAAGAAGGGCATCCTGCAGACAGTGA<br>AGGTGGTGGACGAGCTCGTGAAAGTGATGGGCCGGCACAAGCCCG<br>AGAACATCGTGATCGAAATGGCCAGAGAGAACCAGACCACCCAG<br>AAGGGACAGAAGAACAGCCGCGAGAGAATGAAGCGGATCGAAGA<br>GGGCATCAAAGAGCTGGGCAGCCAGATCCTGAAAGAACACCCCGT<br>GGAAAACACCCAGCTGCAGAACGAGAAGCTGTACCTGTACTACCT<br>GCAGAATGGGCGGGATATGTACGTGGACCAGGAACTGGACATCAA<br>CCGGCTGTCCGACTACGATGTGGACCATATCGTGCCTCAGAGCTTT<br>CTGAAGGACGACTCCATCGATAACAAAGTGCTGACTCGGAGCGAC<br>AAGAACCGGGGCAAGAGCGACAACGTGCCCTCCGAAGAGGTCGT<br>GAAGAAGATGAAGAACTACTGGCGCCAGCTGCTGAATGCCAAGCT<br>GATTACCCAGAGGAAGTTCGACAATCTGACCAAGGCCGAGAGAGG<br>CGGCCTGAGCGAACTGGATAAGGCCGGCTTCATCAAGAGACAGCT<br>GGTGGAAACCCGGCAGATCACAAAGCACGTGGCACAGATCCTGGA<br>CTCCCGGATGAACACTAAGTACGACGAGAACGACAAACTGATCCG<br>GGAAGTGAAAGTGATCACCCTGAAGTCCAAGCTGGTGTCCGATTT<br>CCGGAAGGATTTCCAGTTTTACAAAGTGCGCGAGATCAACAACTA<br>CCACCACGCCCACGACGCCTACCTGAACGCCGTCGTGGGAACCGC<br>CCTGATCAAAAAGTACCCTAAGCTGGAAAGCGAGTTCGTGGACGG<br>CGACGACAAGGTGGACGACGACGACAAGATGATCGCCAAGAGCG

	AGGACGAAATCGGCGACGCTACCGCCAAGTACTTCTTCTACAGCA<br>ACATCATGAACTTTTTCAAGACCGAGATTACCCTGGCCAACGGCG<br>AGATCCGGAAGCGGCCTCTGATCGAGACAAACGGCGAAACAGGC<br>GAGATCGTGTGGGATAAGGGCCGGGACTTTGCCACCGTGCGGAAA<br>GTGCTGTCTATGCCCCAAGTGAATATCGTGAAAAAGACCGAGGTG<br>CAGACAGGCGGCTTCAGCAAAGAGTCTATCCTGCCCAAGAGGAAC<br>AGCGACAAGCTGATCGCCAGAAAGAAGGACTGGGACCCTAAGAA<br>GTACGGCGGCTTCGACAGCCCCACCGTGGCCTATTCTGTGCTGGTG<br>GTGGCCAAAGTGGAAAAGGGCAAGTCCAAGAAACTGAAGAGTGT<br>GAAAGAGCTGCTGGGGATCACCATCATGGAAAGAAGCAGCTTCGA<br>GAAGAATCCCATCGACTTTCTGGAAGCCAAGGGCTACAAAGAAGT<br>GAAAAAGGACCTGATCATCAAGCTGCCTAAGTACTCCCTGTTCGA<br>GCTGGAAAACGGCCGGAAGAGAATGCTGGCCTCTGCCGGCGAACT<br>GCAGAAGGGAAACGAACTGGCCCTGCCCTCCAAATATGTGAACTT<br>CCTGTACCTGGCCAGCCACTATGAGAAGCTGAAGGGCTCCCCCGA<br>GGATAATGAGCAGAAACAGCTGTTTGTGGAACAGCACAAACACTA<br>CCTGGACGAGATCATCGAGCAGATCAGCGAGTTCTCCAAGAGAGT<br>GATCCTGGCCGACGCTAATCTGGACAAGGTGCTGAGCGCCTACAA<br>CAAGCACAGAGACAAGCCTATCAGAGAGCAGGCCGAGAATATCAT<br>CCACCTGTTTACCCTGACCAATCTGGGAGCCCCTGCCGCCTTCAAG<br>TACTTTGACACCACCATCGACCGGAAGAGGTACACCAGCACCAAA<br>GAGGTGCTGGACGCCACCCTGATCCACCAGAGCATCACCGGCCTG<br>TACGAGACACGGATCGACCTGTCTCAGCTGGGAGGCGACGCCTAT<br>CCCTATGACGTGCCCGATTATGCCAGCCTGGGCAGCGGCTCCCCCA<br>AGAAAAAACGCAAGGTGGAAGATCCTAAGAAAAAGCGGAAAGGT<br>TCTAGTGAAACCCCGGGAACAAGTGAGTCGGCCACCCCTGAAGGT<br>GGATCAGGGGGTAGCGGATCCTCTTACGATAATTATGTTACGATTC<br>TGGACGAGGAAACCTTAAAGGCTTGGATCGCTAAATTAGAGAAGG<br>CTCCTGTTTTCGCTTTCGACACGGAAACGGATTCTCTGGACAATAT<br>TAGTGCGAATCTTGTTGGTCTGAGTTTCGCAATTGAACCGGGTGTT<br>GCTGCTTACATCCCTGTGGCACACGACTACCTGGACGCTCCGGACC<br>AGATTTCACGTGAACGCGCTCTGGAACTGCTGAAGCCTTTATTAGA<br>GGACGAGAAAGCTTTGAAAGTTGGTCAGAATTTGAAGTATGCTCG<br>TGGAATCTTAGCTAATTATGGTATCGAGTTGCGCGGTATCGCTTTC<br>GACACGATGTTGGAATCTTATATCCTGAACTCTGTCGCTGGTCGCC<br>ATGACATGGACTCTCTGGCTGAGCGCTGGCTGAAACATAAGACGA<br>TTACCTTCGAGGAAATCGCAGGAAAGGGTAAGAACCAGCTCACGT<br>TCAATCAAATCGCTCTGGAGGAAGCTGGTCGCTATGCTGCTGAGG<br>ACGCTGACGTTACTCTGCAACTGCACTTGAAGATGTGGCCTGACTT<br>GCAGAAGCATAAGGGTCCACTGAATGTTTTTGAAAACATTGAGAT<br>GCCTTTGGTTCCAGTTCTGTCTCGTATCGAGCGCAATGGCGTTAAA<br>ATTGACCCAAAGGTTTTACATAACCACTCAGAGGAACTGACGCTG<br>CGCTTAGCCGAATTGGAGAAAAAGGCTCACGAGATCGCTGGCGAA<br>GAGTTCAATCTGTCATCTACGAAACAACTGCAGACTATCCTGTTCG<br>AGAAGCAAGGTATCAAGCCATTAAAAAAGACCCCTGGCGGTGCTC<br>CGTCTACCTCTGAGGAAGTTTTGGAGGAGTTAGCTTTGGATTACCC

	TCTGCCGAAGGTTATCTTGGAATACCGCGGTTTGGCTAAATTGAAG TCTACTTATACGGATAAACTTCCTTTGATGATTAATCCAAAGACGG GTCGCGTTCACACGTCGTACCATCAAGCTGTTACCGCTACCGGTCG CCTGTCTTCTACGGATCCGAATTTACAGAATATTCCTGTGCGCAAT GAGGAGGGCCGCCGCATTCGTCAAGCTTTTATCGCTCCGGAAGAC TACGTTATCGTTTCTGCTGATTATTCTCAAAATGAATTACGTATCAT GGCTCACCTGTCTCGCGATAAGGGTCTGTTGACGGCCTTTGCTGAG GGTAAGGACATTCATCGTGCTACCGCTGCTGAGGTTTACGGCCTGC CGTTGGAAACGGTTACGTCTGAACAGCGTCGCTCTGCTAAGCGTAT TAATTTCGGCTTAATCTACGGTATGTCTGCGTTTGGCTTAGCTCGTC AGCTGAATATCCCGCGCAAGGAAGCTCAAAAATATATGGATCTGT ATTTTGAGCGTTACCACGGTGTTTTGGAATACATGGAGCGTACGCG CGCGCAAGCTAAGGAACAAGGTTATGTGGAAACCTTGGATGGTCG TCGCTTGTACTTGCCTGACATTAAGTCTTCTAACGGCGCCCGCCGC GCTGCTGCCGAGCGCGCTGCTATCAATGCTCCGATGCAAGGTACTG CTGCTGATATTATTAAGCGTGCTATGATCGCTGTGGACGCTTGGCT GCAAGCTGAACAGCCTCGCGTTCGCATGATTATGCAAGTTCATGAC GAGTTGGTTTTCGAGGTGCATAAGGACGACGTGGACGCTGTTGCT AAACAAATCCACCAGTTGATGGAGAATTGCACGCGCTTAGACGTT CCGCTGCTGGTTGAAGTTGGTTCTGGTGAAAACTGGGACCAGGCTC ACTAATGGCTGAAAACTGGGACCAGGCTCACTAACTCAGATCCTA CTAGGTTTAATAAACATCTTTATTTTCATTACATCTGTGTGTTGGTT TTTTGTGTGGTACCCAATTCGCCCTATAGTGAGTCGTATTACTCACT GGCCGTCGTTTTACAACGTCGTGACTGGGAAAACCCTGGCGTTACC CAACTTAATCGCCTTGCAGCACATCCCCCTTTCGCCAGCTGGCGTA ATAGCGAAGAGGCCCGCACCGATCGCCCTTCCCAACAGTTGCGCA GCCTGAATGGCGAATGGGACGCGCCCTGTAGCGGCGCATTAAGCG CGGCGGGTGTGGTGGTTACGCGCAGCGTGACCGCTACACTTGCCA GCGCCCTAGCGCCCGCTCCTTTCGCTTTCTTCCCTTCCTTTCTCGCC ACGTTCGCCGGCTTTCCCCGTCAAGCTCTAAATCGGGGGCTCCCTT TAGGGTTCCGATTTAGTGCTTTACGGCACCTCGACCCCAAAAAACT TGATTAGGGTGATGGTTCACGTAGTGGGCCATCGCCCTGATAGAC GGTTTTTCGCCCTTTGACGTTGGAGTCCACGTTCTTTAATAGTGGA CTCTTGTTCCAAACTGGAACAACACTCAACCCTATCTCGGTCTATT CTTTTGATTTATAAGGGATTTTGCCGATTTCGGCCTATTGGTTAAA AAATGAGCTGATTTAACAAAAATTTAACGCGAATTTTAACAAAAT ATTAACGCTTACAATTTAGGTGGCACTTTTCGGGGAAATGTG<br><br>**Ampicillin resistance cassette-ColE1 origin of replication-hU6 promoter-gRNA sequence-SpCas9 gRNA scaffold-CMV promoter-NLS-mCherry-GSGM- SuperFi-nCas9(D10A)-NLS-linker-PolI5MΔ-stop-SV40 polyA**
p.Sniper-nCas9-PolI5MΔ	CGCGGAACCCCTATTTGTTTATTTTTCTAAATACATTCAAATATGTA TCCGCTCATGAGACAATAACCCTGATAAATGCTTCAATAATATTGA AAAAGGAAGAGTATGAGTATTCAACATTTCCGTGTCGCCCTTATTC

	CCTTTTTTGCGGCATTTTGCCTTCCTGTTTTTGCTCACCCAGAAACG<br>CTGGTGAAAGTAAAAGATGCTGAAGATCAGTTGGGTGCACGAGTG<br>GGTTACATCGAACTGGATCTCAACAGCGGTAAGATCCTTGAGAGT<br>TTTCGCCCCGAAGAACGTTTTCCAATGATGAGCACTTTTAAAGTTC<br>TGCTATGTGGCGCGGTATTATCCCGTATTGACGCCGGGCAAGAGC<br>AACTCGGTCGCCGCATACACTATTCTCAGAATGACTTGGTTGAGTA<br>CTCACCAGTCACAGAAAAGCATCTTACGGATGGCATGACAGTAAG<br>AGAATTATGCAGTGCTGCCATAACCATGAGTGATAACACTGCGGC<br>CAACTTACTTCTGACAACGATCGGAGGACCGAAGGAGCTAACCGC<br>TTTTTTGCACAACATGGGGGATCATGTAACTCGCCTTGATCGTTGG<br>GAACCGGAGCTGAATGAAGCCATACCAAACGACGAGCGTGACACC<br>ACGATGCCTGTAGCAATGGCAACAACGTTGCGCAAACTATTAACT<br>GGCGAACTACTTACTCTAGCTTCCCGGCAACAATTAATAGACTGGA<br>TGGAGGCGGATAAAGTTGCAGGACCACTTCTGCGCTCGGCCCTTCC<br>GGCTGGCTGGTTTATTGCTGATAAATCTGGAGCCGGTGAGCGTGGT<br>TCTCGCGGTATCATTGCAGCACTGGGGCCAGATGGTAAGCCCTCCC<br>GTATCGTAGTTATCTACACGACGGGGAGTCAGGCAACTATGGATG<br>AACGAAATAGACAGATCGCTGAGATAGGTGCCTCACTGATTAAGC<br>ATTGGTAACTGTCAGACCAAGTTTACTCATATATACTTTAGATTGA<br>TTTAAAACTTCATTTTTAATTTAAAAGGATCTAGGTGAAGATCCTT<br>TTTGATAATCTCATGACCAAAATCCCTTAACGTGAGTTTTCGTTCC<br>ACTGAGCGTCAGACCCCGTAGAAAAGATCAAAGGATCTTCTTGAG<br>ATCCTTTTTTTCTGCGCGTAATCTGCTGCTTGCAAACAAAAAAACC<br>ACCGCTACCAGCGGTGGTTTGTTTGCCGGATCAAGAGCTACCAACT<br>CTTTTTCCGAAGGTAACTGGCTTCAGCAGAGCGCAGATACCAAAT<br>ACTGTCCTTCTAGTGTAGCCGTAGTTAGGCCACCACTTCAAGAACT<br>CTGTAGCACCGCCTACATACCTCGCTCTGCTAATCCTGTTACCAGT<br>GGCTGCTGCCAGTGGCGATAAGTCGTGTCTTACCGGGTTGGACTCA<br>AGACGATAGTTACCGGATAAGGCGCAGCGGTCGGGCTGAACGGGG<br>GGTTCGTGCACACAGCCCAGCTTGGAGCGAACGACCTACACCGAA<br>CTGAGATACCTACAGCGTGAGCTATGAGAAAGCGCCACGCTTCCC<br>GAAGGGAGAAAGGCGGACAGGTATCCGGTAAGCGGCAGGGTCGG<br>AACAGGAGAGCGCACGAGGGAGCTTCCAGGGGGAAACGCCTGGT<br>ATCTTTATAGTCCTGTCGGGTTTCGCCACCTCTGACTTGAGCGTCG<br>ATTTTTGTGATGCTCGTCAGGGGGGCGGAGCCTATGGAAAAACGC<br>CAGCAACGCGGCCTTTTTACGGTTCCTGGCCTTTTGCTGGCCTTTTG<br>CTCACATGTTCTTTCCTGCGTTATCCCCTGATTCTGTGGATAACCGT<br>ATTACCGCCTTTGAGTGAGCTGATACCGCTCGCCGCAGCCGAACG<br>ACCGAGCGCAGCGAGTCAGTGAGCGAGGAAGCGGAAGAGCGCCC<br>AATACGCAAACCGCCTCTCCCCGCGCGTTGGCCGATTCATTAATGC<br>AGCTGGCACGACAGGTTTCCCGACTGGAAAGCGGGCAGTGAGCGC<br>AACGCAATTAATGTGAGTTAGCTCACTCATTAGGCACCCCAGGCTT<br>TACACTTTATGCTTCCGGCTCGTATGTTGTGTGGAATTGTGAGCGG<br>ATAACAATTTCACACAGGAAACAGCTATGACCATGATTACGCCAA<br>GCGCGCAATTAACCCTCACTAAAGGGAACAAAAGCTGGAGCTCCA<br>CCGCGGTGGCGGCCGCTCTTAAGGGGTGCAGCGGCCTCCGCGCCG

	GGTTTTGGCGCCTCGATCCAAGGTCGGCAGGAAGAGGGCCTATT<br>TCCCATGATTCCTTCATATTTGCATATACGATACAAGGCTGTTAGA<br>GAGATAATTAGAATTAATTTGACTGTAAACACAAAGATATTAGTA<br>CAAAATACGTGACGTAGAAAGTAATAATTTCTTGGGTAGTTTGCA<br>GTTTTAAAATTATGTTTTAAAATGGACTATCATATGCTTACCGTAA<br>CTTGAAAGTATTTCGATTTCTTGGCTTTATATATCTTGTGGAAAGG<br>ACGAAACACC-[gRNA SEQUENCE]-<br>GTTTTAGAGCTAGAAATAGCAAGTTAAAATAAGGCTAGTCCGTTA<br>TCAACTTGAAAAAGTGGCACCGAGTCGGTGCTTTTTTCCGCGGCCT<br>CTAGACTCGAGGCGTTGACATTGATTATTGACTAGTTATTAATAGT<br>AATCAATTACGGGGTCATTAGTTCATAGCCCATATATGGAGTTCCG<br>CGTTACATAACTTACGGTAAATGGCCCGCCTGGCTGACCGCCCAAC<br>GACCCCCGCCCATTGACGTCAATAATGACGTATGTTCCCATAGTAA<br>CGCCAATAGGGACTTTCCATTGACGTCAATGGGTGGAGTATTTACG<br>GTAAACTGCCCACTTGGCAGTACATCAAGTGTATCATATGCCAAGT<br>ACGCCCCCTATTGACGTCAATGACGGTAAATGGCCCGCCTGGCATT<br>ATGCCCAGTACATGACCTTATGGGACTTTCCTACTTGGCAGTACAT<br>CTACGTATTAGTCATCGCTATTACCATGGTGATGCGGTTTTGGCAG<br>TACATCAATGGGCGTGGATAGCGGTTTGACTCACGGGGATTTCCA<br>AGTCTCCACCCCATTGACGTCAATGGGAGTTTGTTTTGGCACCAAA<br>ATCAACGGGACTTTCCAAAATGTCGTAACAACTCCGCCCCATTGAC<br>GCAAATGGGCGGTAGGCGTGTACGGTGGGAGGTCTATATAAGCAG<br>AGCTCTCTGGCTAACTACCGGTGCCACCATGGCCCCAAAGAAGAA<br>GCGGAAGGTCGGTATCCACGGAGTCCCAGCAGCCGTGAGCAAGGG<br>CGAGGAGGATAACATGGCCATCATCAAGGAGTTCATGCGCTTCAA<br>GGTGCACATGGAGGGCTCCGTGAACGGCCACGAGTTCGAGATCGA<br>GGGCGAGGGCGAGGGCCGCCCCTACGAGGGCACCCAGACCGCCA<br>AGCTGAAGGTGACCAAGGGTGGCCCCCTGCCCTTCGCCTGGGACA<br>TCCTGTCCCCTCAGTTCATGTACGGCTCCAAGGCCTACGTGAAGCA<br>CCCCGCCGACATCCCCGACTACTTGAAGCTGTCCTTCCCCGAGGGC<br>TTCAAGTGGGAGCGCGTGATGAACTTCGAGGACGGCGGCGTGGTG<br>ACCGTGACCCAGGACTCCTCCCTGCAGGACGGCGAGTTCATCTAC<br>AAGGTGAAGCTGCGCGGCACCAACTTCCCCTCCGACGGCCCCGTA<br>ATGCAGAAGAAGACCATGGGCTGGGAGGCCTCCTCCGAGCGGATG<br>TACCCCGAGGACGGCGCCCTGAAGGGCGAGATCAAGCAGAGGCTG<br>AAGCTGAAGGACGGCGGCCACTACGACGCTGAGGTCAAGACCACC<br>TACAAGGCCAAGAAGCCCGTGCAGCTGCCCGGCGCCTACAACGTC<br>AACATCAAGTTGGACATCACCTCCCACAACGAGGACTACACCATC<br>GTGGAACAGTACGAACGCGCCGAGGGCCGCCACTCCACCGGCGGC<br>ATGGACGAGCTGTACAAGGGATCCGGTATGGACAAGAAGTACAGC<br>ATCGGCCTGGCCATCGGCACCAACTCTGTGGGCTGGGCCGTGATC<br>ACCGACGAGTACAAGGTGCCCAGCAAGAAATTCAAGGTGCTGGGC<br>AACACCGACCGGCACAGCATCAAGAAGAACCTGATCGGCGCCCTG<br>CTGTTCGACAGCGGAGAAACAGCCGAGGCCACCCGGCTGAAGAGA<br>ACCGCCAGAAGAAGATACACCAGACGGAAGAACCGGATCTGCTAT<br>CTGCAAGAGATCTTCAGCAACGAGATGGCCAAGGTGGACGACAGC

	TTCTTCCACAGACTGGAAGAGTCCTTCCTGGTGGAAGAGGATAAG<br>AAGCACGAGCGGCACCCCATCTTCGGCAACATCGTGGACGAGGTG<br>GCCTACCACGAGAAGTACCCCACCATCTACCACCTGAGAAAGAAA<br>CTGGTGGACAGCACCGACAAGGCCGACCTGCGGCTGATCTATCTG<br>GCCCTGGCCCACATGATCAAGTTCCGGGGCCACTTCCTGATCGAGG<br>GCGACCTGAACCCCGACAACAGCGACGTGGACAAGCTGTTCATCC<br>AGCTGGTGCAGACCTACAACCAGCTGTTCGAGGAAAACCCCATCA<br>ACGaCAGCGGCGTGGACGCCAAGGCCATCCTGTCTGCCAGACTGA<br>GCAAGAGCAGACGGCTGGAAAATCTGATCGCCCAGCTGCCCGGCG<br>AGAAGAAGAATGGCCTGTTCGGCAACCTGATTGCCCTGAGCCTGG<br>GCCTGACCCCCAACTTCAAGAGCAACTTCGACCTGGCCGAGGATG<br>CCAAACTGCAGCTGAGCAAGGACACCTACGACGACGACCTGGACA<br>ACCTGCTGGCCCAGATCGGCGACCAGTACGCCGACCTGTTTCTGGC<br>CGCCAAGAACCTGTCCGACGCCATCCTGCTGAGCGACATCCTGAG<br>AGTGAACACCGAGATCACCAAGGCCCCCCTGAGCGCCTCTATGAT<br>CAAGAGATACGACGAGCACCACCAGGACCTGACCCTGCTGAAAGC<br>TCTCGTGCGGCAGCAGCTGCCTGAGAAGTACAAAGAGATTTTCTTC<br>GACCAGAGCAAGAACGGCTACGCCGGCTACATCGATGGCGGAGCC<br>AGCCAGGAAGAGTTCTACAAGTTCATCAAGCCCATCCTGGAAAAG<br>ATGGACGGCACCGAGGAACTGCTCGTGAAGCTGAACAGAGAGGA<br>CCTGCTGCGGAAGCAGCGGACCTTCGACAACGGCAGCATCCCCCA<br>CCAGATCCACCTGGGAGAGCTGCACGCCATTCTGCGGCGGCAGGA<br>AGATTTTTACCCATTCCTGAAGGACAACCGGGAAAAGATCGAGAA<br>GATCCTGACCTTCCGCATCCCCTACTACGTGGGCCCTCTGGCCAGG<br>GGAAACAGCAGATTCGCCTGGATGACCAGAAAGAGCGAGGAAAC<br>CATCACCCCCTGGAACTTCGAGGAAGTGGTGGACAAGGGCGCCAG<br>CGCCCAGAGCTTCATCGAGCGGATGACCAACTTCGATAAGAACCT<br>GCCCAACGAGAAGGTGCTGCCCAAGCACAGCCTGCTGTACGAGTA<br>CTTCACCGTGTACAACGAGCTGACCAAAGTGAAATACGTGACCGA<br>GGGAATGAGAAAGCCCGCCTTCCTGAGCGGCGAGCAGAAAAAAG<br>CCATCGTGGACCTGCTGTTCAAGACCAACCGGAAAGTGACCGTGA<br>AGCAGCTGAAAGAGGACTACTTCAAGAAAATCGAGTGCTTCGACT<br>CCGTGGAAATCTCCGGCGTGGAAGATCGGTTCAACGCCTCCCTGG<br>GCACATACCACGATCTGCTGAAAATTATCAAGGACAAGGACTTCC<br>TGGACAATGAGGAAAACGAGGACATTCTGGAAGATATCGTGCTGA<br>CCCTGACACTGTTTGAGGACAGAGAGATGATCGAGGAACGGCTGA<br>AAACCTATGCCCACCTGTTCGACGACAAAGTGATGAAGCAGCTGA<br>AGCGGCGGAGATACACCGGCTGGGGCAGGCTGAGCCGGAAGCTG<br>ATCAACGGCATCCGGGACAAGCAGTCCGGCAAGACAATCCTGGAT<br>TTCCTGAAGTCCGACGGCTTCGCCAACAGAAACTTCATGCAGCTGA<br>TCCACGACGACAGCCTGACCTTTAAAGAGGACATCCAGAAAGCCC<br>AGGTGTCCGGCCAGGGCGATAGCCTGCACGAGCACATTGCCAATC<br>TGGCCGGCAGCCCCGCCATTAAGAAGGGCATCCTGCAGACAGTGA<br>AGGTGGTGGACGAGCTCGTGAAAGTGATGGGCCGGCACAAGCCCG<br>AGAACATCGTGATCGAAATGGCCAGAGAGAACCAGACCACCCAG<br>AAGGGACAGAAGAACAGCCGCGAGAGAATGAAGCGGATCGAAGA

		GGGCATCAAAGAGCTGGGCAGCCAGATCCTGAAAGAACACCCCGT<br>GGAAAACACCCAGCTGCAGAACGAGAAGCTGTACCTGTACTACCT<br>GCAGAATGGGCGGGATATGTACGTGGACCAGGAACTGGACATCAA<br>CCGGCTGTCCGACTACGATGTGGACCATATCGTGCCTCAGAGCTTT<br>CTGAAGGACGACTCCATCGATAACAAAGTGCTGACTCGGAGCGAC<br>AAGAACCGGGGCAAGAGCGACAACGTGCCCTCCGAAGAGGTCGT<br>GAAGAAGATGAAGAACTACTGGCGCCAGCTGCTGAATGCCAAGCT<br>GATTACCCAGAGGAAGTTCGACAATCTGACCAAGGCCGAGAGAGG<br>CGGCCTGAGCGAACTGGATAAGGCCGGCTTCATCAAGAGACAGCT<br>GGTGGAAACCCGGCAGATCACAAAGCACGTGGCACAGATCCTGGA<br>CTCCCGGATGAACACTAAGTACGACGAGAACGACAAACTGATCCG<br>GGAAGTGAAAGTGATCACCCTGAAGTCCAAGCTGGTGTCCGATTT<br>CCGGAAGGATTTCCAGTTTTACAAAGTGCGCGAGATCAACAACTA<br>CCACCACGCCCACGACGCCTACCTGAACGCCGTCGTGGGAACCGC<br>CCTGATCAAAAAGTACCCTAAGCTGGAAAGCGAGTTCGTGTACGG<br>CGACTACAAGGTGTACGACGTGCGGAAGATGATCGCCAAGAGCGA<br>GCAGGAAATCGGCAAGGCTACCGCCAAGTACTTCTTCTACAGCAA<br>CATCATGAACTTTTTCAAGACCGAGATTACCCTGGCCAACGGCGA<br>GATCCGGAAGCGGCCTCTGATCGAGACAAACGGCGAAACAGGCG<br>AGATCGTGTGGGATAAGGGCCGGGACTTTGCCACCGTGCGGAAAG<br>TGCTGTCTATGCCCCAAGTGAATATCGTGAAAAAGACCGAGGTGC<br>AGACAGGCGGCTTCAGCAAAGAGTCTATCCTGCCCAAGAGGAACA<br>GCGACAAGCTGATCGCCAGAAAGAAGGACTGGGACCCTAAGAAG<br>TACGGCGGCTTCGACAGCCCCACCGTGGCCTATTCTGTGCTGGTGG<br>TGGCCAAAGTGGAAAAGGGCAAGTCCAAGAAACTGAAGAGTGTG<br>AAAGAGCTGCTGGGGATCACCATCATGGAAAGAAGCAGCTTCGAG<br>AAGAATCCCATCGACTTTCTGGAAGCCAAGGGCTACAAAGAAGTG<br>AAAAAGGACCTGATCATCAAGCTGCCTAAGTACTCCCTGTTCGAG<br>CTGGAAAACGGCCGGAAGAGAATGCTGGCCTCTGCCGGCGAACTG<br>CAGAAGGGAAACGAACTGGCCCTGCCCTCCAAATATGTGAACTTC<br>CTGTACCTGGCCAGCCACTATGAGAAGCTGAAGGGCTCCCCCGAG<br>GATAATGAGCAGAAACAGCTGTTTGTGGAACAGCACAAACACTAC<br>CTGGACGAGATCATCGAGCAGATCAGCGAGTTCTCCAAGAGAGTG<br>ATCCTGGCCGACGCTAATCTGGACAAGGTGCTGAGCGCCTACAAC<br>AAGCACAGAGACAAGCCTATCAGAGAGCAGGCCGAGAATATCATC<br>CACCTGTTTACCCTGACCAATCTGGGAGCCCCTGCCGCCTTCAAGT<br>ACTTTGACACCACCATCGACCGGAAGAGGTACACCAGCACCAAAG<br>AGGTGCTGGACGCCACCCTGATCCACCAGAGCATCACCGGCCTGT<br>ACGAGACACGGATCGACCTGTCTCAGCTGGGAGGCGACGCCTATC<br>CCTATGACGTGCCCGATTATGCCAGCCTGGGCAGCGGCTCCCCCAA<br>GAAAAAACGCAAGGTGGAAGATCCTAAGAAAAAGCGGAAAGGTT<br>CTAGTGAAACCCCGGGAACAAGTGAGTCGGCCACCCCTGAAGGTG<br>GATCAGGGGGTAGCGGATCCTCTTACGATAATTATGTTACGATTCT<br>GGACGAGGAAACCTTAAAGGCTTGGATCGCTAAATTAGAGAAGGC<br>TCCTGTTTTCGCTTTCGACACGGAAACGGATTCTCTGGACAATATT<br>AGTGCGAATCTTGTTGGTCTGAGTTTCGCAATTGAACCGGGTGTTG

	CTGCTTACATCCCTGTGGCACACGACTACCTGGACGCTCCGGACCA<br>GATTTCACGTGAACGCGCTCTGGAACTGCTGAAGCCTTTATTAGAG<br>GACGAGAAAGCTTTGAAAGTTGGTCAGAATTTGAAGTATGCTCGT<br>GGAATCTTAGCTAATTATGGTATCGAGTTGCGCGGTATCGCTTTCG<br>ACACGATGTTGGAATCTTATATCCTGAACTCTGTCGCTGGTCGCCA<br>TGACATGGACTCTCTGGCTGAGCGCTGGCTGAAACATAAGACGAT<br>TACCTTCGAGGAAATCGCAGGAAAGGGTAAGAACCAGCTCACGTT<br>CAATCAAATCGCTCTGGAGGAAGCTGGTCGCTATGCTGCTGAGGA<br>CGCTGACGTTACTCTGCAACTGCACTTGAAGATGTGGCCTGACTTG<br>CAGAAGCATAAGGGTCCACTGAATGTTTTTGAAAACATTGAGATG<br>CCTTTGGTTCCAGTTCTGTCTCGTATCGAGCGCAATGGCGTTAAAA<br>TTGACCCAAAGGTTTTACATAACCACTCAGAGGAACTGACGCTGC<br>GCTTAGCCGAATTGGAGAAAAAGGCTCACGAGATCGCTGGCGAAG<br>AGTTCAATCTGTCATCTACGAAACAACTGCAGACTATCCTGTTCGA<br>GAAGCAAGGTATCAAGCCATTAAAAAAGACCCCTGGCGGTGCTCC<br>GTCTACCTCTGAGGAAGTTTTGGAGGAGTTAGCTTTGGATTACCCT<br>CTGCCGAAGGTTATCTTGGAATACCGCGGTTTGGCTAAATTGAAGT<br>CTACTTATACGGATAAACTTCCTTTGATGATTAATCCAAAGACGGG<br>TCGCGTTCACACGTCGTACCATCAAGCTGTTACCGCTACCGGTCGC<br>CTGTCTTCTACGGATCCGAATTTACAGAATATTCCTGTGCGCAATG<br>AGGAGGGCCGCCGCATTCGTCAAGCTTTTATCGCTCCGGAAGACT<br>ACGTTATCGTTTCTGCTGATTATTCTCAAAATGAATTACGTATCAT<br>GGCTCACCTGTCTCGCGATAAGGGTCTGTTGACGGCCTTTGCTGAG<br>GGTAAGGACATTCATCGTGCTACCGCTGCTGAGGTTTACGGCCTGC<br>CGTTGGAAACGGTTACGTCTGAACAGCGTCGCTCTGCTAAGCGTAT<br>TAATTTCGGCTTAATCTACGGTATGTCTGCGTTTGGCTTAGCTCGTC<br>AGCTGAATATCCCGCGCAAGGAAGCTCAAAAATATATGGATCTGT<br>ATTTTGAGCGTTACCACGGTGTTTTGGAATACATGGAGCGTACGCG<br>CGCGCAAGCTAAGGAACAAGGTTATGTGGAAACCTTGGATGGTCG<br>TCGCTTGTACTTGCCTGACATTAAGTCTTCTAACGGCGCCCGCCGC<br>GCTGCTGCCGAGCGCGCTGCTATCAATGCTCCGATGCAAGGTACTG<br>CTGCTGATATTATTAAGCGTGCTATGATCGCTGTGGACGCTTGGCT<br>GCAAGCTGAACAGCCTCGCGTTCGCATGATTATGCAAGTTCATGAC<br>GAGTTGGTTTTCGAGGTGCATAAGGACGACGTGGACGCTGTTGCT<br>AAACAAATCCACCAGTTGATGGAGAATTGCACGCGCTTAGACGTT<br>CCGCTGCTGGTTGAAGTTGGTTCTGGTGAAAACTGGGACCAGGCTC<br>ACTAATGGCTGAAAACTGGGACCAGGCTCACTAACTCAGATCCTA<br>CTAGGTTTAATAAACATCTTTATTTTCATTACATCTGTGTGTTGGTT<br>TTTTGTGTGGTACCCAATTCGCCCTATAGTGAGTCGTATTACTCACT<br>GGCCGTCGTTTTACAACGTCGTGACTGGGAAAACCCTGGCGTTACC<br>CAACTTAATCGCCTTGCAGCACATCCCCCTTTCGCCAGCTGGCGTA<br>ATAGCGAAGAGGCCCGCACCGATCGCCCTTCCCAACAGTTGCGCA<br>GCCTGAATGGCGAATGGGACGCGCCCTGTAGCGGCGCATTAAGCG<br>CGGCGGGTGTGGTGGTTACGCGCAGCGTGACCGCTACACTTGCCA<br>GCGCCCTAGCGCCCGCTCCTTTCGCTTTCTTCCCTTCCTTTCTCGCC<br>ACGTTCGCCGGCTTTCCCCGTCAAGCTCTAAATCGGGGGCTCCCTT

	TAGGGTTCCGATTTAGTGCTTTACGGCACCTCGACCCCAAAAAACT<br>TGATTAGGGTGATGGTTCACGTAGTGGGCCATCGCCCTGATAGAC<br>GGTTTTTCGCCCTTTGACGTTGGAGTCCACGTTCTTTAATAGTGGA<br>CTCTTGTTCCAAACTGGAACAACACTCAACCCTATCTCGGTCTATT<br>CTTTTGATTTATAAGGGATTTTGCCGATTTCGGCCTATTGGTTAAA<br>AAATGAGCTGATTTAACAAAAATTTAACGCGAATTTTAACAAAAT<br>ATTAACGCTTACAATTTAGGTGGCACTTTTCGGGGAAATGTG<br><br>**Ampicillin resistance cassette-ColE1 origin of replication-hU6 promoter-gRNA sequence-SpCas9 gRNA scaffold-CMV promoter-NLS-mCherry-GSGM- Sniper-nCas9(D10A)-NLS-linker-PolI5MΔ-stop-SV40 polyA**
p.HSC1.2-nCas9-PolI5MΔ	CGCGGAACCCCTATTTGTTTATTTTTCTAAATACATTCAAATATGTA<br>TCCGCTCATGAGACAATAACCCTGATAAATGCTTCAATAATATTGA<br>AAAAGGAAGAGTATGAGTATTCAACATTTCCGTGTCGCCCTTATTC<br>CCTTTTTTGCGGCATTTTGCCTTCCTGTTTTTGCTCACCCAGAAACG<br>CTGGTGAAAGTAAAAGATGCTGAAGATCAGTTGGGTGCACGAGTG<br>GGTTACATCGAACTGGATCTCAACAGCGGTAAGATCCTTGAGAGT<br>TTTCGCCCCGAAGAACGTTTTCCAATGATGAGCACTTTTAAAGTTC<br>TGCTATGTGGCGCGGTATTATCCCGTATTGACGCCGGGCAAGAGC<br>AACTCGGTCGCCGCATACACTATTCTCAGAATGACTTGGTTGAGTA<br>CTCACCAGTCACAGAAAAGCATCTTACGGATGGCATGACAGTAAG<br>AGAATTATGCAGTGCTGCCATAACCATGAGTGATAACACTGCGGC<br>CAACTTACTTCTGACAACGATCGGAGGACCGAAGGAGCTAACCGC<br>TTTTTTGCACAACATGGGGGATCATGTAACTCGCCTTGATCGTTGG<br>GAACCGGAGCTGAATGAAGCCATACCAAACGACGAGCGTGACACC<br>ACGATGCCTGTAGCAATGGCAACAACGTTGCGCAAACTATTAACT<br>GGCGAACTACTTACTCTAGCTTCCCGGCAACAATTAATAGACTGGA<br>TGGAGGCGGATAAAGTTGCAGGACCACTTCTGCGCTCGGCCCTTCC<br>GGCTGGCTGGTTTATTGCTGATAAATCTGGAGCCGGTGAGCGTGGT<br>TCTCGCGGTATCATTGCAGCACTGGGGCCAGATGGTAAGCCCTCCC<br>GTATCGTAGTTATCTACACGACGGGGAGTCAGGCAACTATGGATG<br>AACGAAATAGACAGATCGCTGAGATAGGTGCCTCACTGATTAAGC<br>ATTGGTAACTGTCAGACCAAGTTTACTCATATATACTTTAGATTGA<br>TTTAAAACTTCATTTTTAATTTAAAAGGATCTAGGTGAAGATCCTT<br>TTTGATAATCTCATGACCAAAATCCCTTAACGTGAGTTTTCGTTCC<br>ACTGAGCGTCAGACCCCGTAGAAAAGATCAAAGGATCTTCTTGAG<br>ATCCTTTTTTTCTGCGCGTAATCTGCTGCTTGCAAACAAAAAAACC<br>ACCGCTACCAGCGGTGGTTTGTTTGCCGGATCAAGAGCTACCAACT<br>CTTTTTCCGAAGGTAACTGGCTTCAGCAGAGCGCAGATACCAAAT<br>ACTGTCCTTCTAGTGTAGCCGTAGTTAGGCCACCACTTCAAGAACT<br>CTGTAGCACCGCCTACATACCTCGCTCTGCTAATCCTGTTACCAGT<br>GGCTGCTGCCAGTGGCGATAAGTCGTGTCTTACCGGGTTGGACTCA<br>AGACGATAGTTACCGGATAAGGCGCAGCGGTCGGGCTGAACGGGG<br>GGTTCGTGCACACAGCCCAGCTTGGAGCGAACGACCTACACCGAA

	CTGAGATACCTACAGCGTGAGCTATGAGAAAGCGCCACGCTTCCC<br>GAAGGGAGAAAGGCGGACAGGTATCCGGTAAGCGGCAGGGTCGG<br>AACAGGAGAGCGCACGAGGGAGCTTCCAGGGGGAAACGCCTGGT<br>ATCTTTATAGTCCTGTCGGGTTTCGCCACCTCTGACTTGAGCGTCG<br>ATTTTTGTGATGCTCGTCAGGGGGGCGGAGCCTATGGAAAAACGC<br>CAGCAACGCGGCCTTTTTACGGTTCCTGGCCTTTTGCTGGCCTTTTG<br>CTCACATGTTCTTTCCTGCGTTATCCCCTGATTCTGTGGATAACCGT<br>ATTACCGCCTTTGAGTGAGCTGATACCGCTCGCCGCAGCCGAACG<br>ACCGAGCGCAGCGAGTCAGTGAGCGAGGAAGCGGAAGAGCGCCC<br>AATACGCAAACCGCCTCTCCCCGCGCGTTGGCCGATTCATTAATGC<br>AGCTGGCACGACAGGTTTCCCGACTGGAAAGCGGGCAGTGAGCGC<br>AACGCAATTAATGTGAGTTAGCTCACTCATTAGGCACCCCAGGCTT<br>TACACTTTATGCTTCCGGCTCGTATGTTGTGTGGAATTGTGAGCGG<br>ATAACAATTTCACACAGGAAACAGCTATGACCATGATTACGCCAA<br>GCGCGCAATTAACCCTCACTAAAGGGAACAAAAGCTGGAGCTCCA<br>CCGCGGTGGCGGCCGCTCTTAAGGGGTGCAGCGGCCTCCGCGCCG<br>GGTTTTGGCGCCTCGATCCAAGGTCGGGCAGGAAGAGGGCCTATT<br>TCCCATGATTCCTTCATATTTGCATATACGATACAAGGCTGTTAGA<br>GAGATAATTAGAATTAATTTGACTGTAAACACAAAGATATTAGTA<br>CAAAATACGTGACGTAGAAAGTAATAATTTCTTGGGTAGTTTGCA<br>GTTTTAAAATTATGTTTTAAAATGGACTATCATATGCTTACCGTAA<br>CTTGAAAGTATTTCGATTTCTTGGCTTTATATATCTTGTGGAAAGG<br>ACGAAACACC-[gRNA SEQUENCE]-<br>GTTTTAGAGCTAGAAATAGCAAGTTAAAATAAGGCTAGTCCGTTA<br>TCAACTTGAAAAAGTGGCACCGAGTCGGTGCTTTTTTCCGCGGCCT<br>CTAGACTCGAGGCGTTGACATTGATTATTGACTAGTTATTAATAGT<br>AATCAATTACGGGGTCATTAGTTCATAGCCCATATATGGAGTTCCG<br>CGTTACATAACTTACGGTAAATGGCCCGCCTGGCTGACCGCCCAAC<br>GACCCCCGCCCATTGACGTCAATAATGACGTATGTTCCCATAGTAA<br>CGCCAATAGGGACTTTCCATTGACGTCAATGGGTGGAGTATTTACG<br>GTAAACTGCCCACTTGGCAGTACATCAAGTGTATCATATGCCAAGT<br>ACGCCCCCTATTGACGTCAATGACGGTAAATGGCCCGCCTGGCATT<br>ATGCCCAGTACATGACCTTATGGGACTTTCCTACTTGGCAGTACAT<br>CTACGTATTAGTCATCGCTATTACCATGGTGATGCGGTTTTGGCAG<br>TACATCAATGGGCGTGGATAGCGGTTTGACTCACGGGGATTTCCA<br>AGTCTCCACCCCATTGACGTCAATGGGAGTTTGTTTTGGCACCAAA<br>ATCAACGGGACTTTCCAAAATGTCGTAACAACTCCGCCCCATTGAC<br>GCAAATGGGCGGTAGGCGTGTACGGTGGGAGGTCTATATAAGCAG<br>AGCTCTCTGGCTAACTACCGGTGCCACCATGGCCCCAAAGAAGAA<br>GCGGAAGGTCGGTATCCACGGAGTCCCAGCAGCCGTGAGCAAGGG<br>CGAGGAGGATAACATGGCCATCATCAAGGAGTTCATGCGCTTCAA<br>GGTGCACATGGAGGGCTCCGTGAACGGCCACGAGTTCGAGATCGA<br>GGGCGAGGGCGAGGGCCGCCCCTACGAGGGCACCCAGACCGCCA<br>AGCTGAAGGTGACCAAGGGTGGCCCCCTGCCCTTCGCCTGGGACA<br>TCCTGTCCCCTCAGTTCATGTACGGCTCCAAGGCCTACGTGAAGCA<br>CCCCGCCGACATCCCCGACTACTTGAAGCTGTCCTTCCCCGAGGGC

		TTCAAGTGGGAGCGCGTGATGAACTTCGAGGACGGCGGCGTGGTG<br>ACCGTGACCCAGGACTCCTCCCTGCAGGACGGCGAGTTCATCTAC<br>AAGGTGAAGCTGCGCGGCACCAACTTCCCCTCCGACGGCCCCGTA<br>ATGCAGAAGAAGACCATGGGCTGGGAGGCCTCCTCCGAGCGGATG<br>TACCCCGAGGACGGCGCCCTGAAGGGCGAGATCAAGCAGAGGCTG<br>AAGCTGAAGGACGGCGGCCACTACGACGCTGAGGTCAAGACCACC<br>TACAAGGCCAAGAAGCCCGTGCAGCTGCCCGGCGCCTACAACGTC<br>AACATCAAGTTGGACATCACCTCCCACAACGAGGACTACACCATC<br>GTGGAACAGTACGAACGCGCCGAGGGCCGCCACTCCACCGGCGGC<br>ATGGACGAGCTGTACAAGGGATCCGGTATGGACAAGAAGTACAGC<br>ATCGGCCTGGCCATCGGCACCGCCTCTGTGGGCTGGGCCGTGATCA<br>CCGACGAGTACAAGGTGCCCAGCAAGAAATTCAAGGTGCTGGGCA<br>ACACCGACCGGCACAGCATCAAGAAGAACCTGATCGGCGCCCTGC<br>TGTTCGACAGCGGAGAAACAGCCGAGGCCACCCGGCTGAAGAGA<br>ACCGCCAGAAGAAGATACACCAGACGGAAGAACCGGATCTGCTAT<br>CTGCAAGAGATCTTCAGCAACGAGATGGCCAAGGTGGACGACAGC<br>TTCTTCCACAGACTGGAAGAGTCCTTCCTGGTGGAAGAGGATAAG<br>AAGCACGAGCGGCACCCCATCTTCGGCAACATCGTGGACGAGGTG<br>GCCTACCACGAGAAGTACCCCACCATCTACCACCTGAGAAAGAAA<br>CTGGTGGACAGCACCGACAAGGCCGACCTGCGGCTGATCTATCTG<br>GCCCTGGCCCACATGATCAAGTTCCGGGGCCACTTCCTGATCGAGG<br>GCGACCTGAACCCCGACAACAGCGACGTGGACAAGCTGTTCATCC<br>AGCTGGTGCAGACCTACAACCAGCTGTTCGAGGAAAACCCCATCA<br>ACGCCAGCGGCGTGGACGCCAAGGCCATCCTGTCTGCCAGACTGA<br>GCAAGAGCAGACGGCTGGAAAATCTGATCGCCCAGCTGCCCGGCG<br>AGAAGAAGAATGGCCTGTTCGGCAACCTGATTGCCCTGAGCCTGG<br>GCCTGACCCCCAACTTCAAGAGCAACTTCGACCTGGCCGAGGATG<br>CCAAACTGCAGCTGAGCAAGGACACCTACGACGACGACCTGGACA<br>ACCTGCTGGCCCAGATCGGCGACCAGTACGCCGACCTGTTTCTGGC<br>CGCCAAGAACCTGTCCGACGCCATCCTGCTGAGCGACATCCTGAG<br>AGTGAACACCGAGATCACCAAGGCCCCCCTGAGCGCCTCTATGAT<br>CAAGAGATACGACGAGCACCACCAGGACCTGACCCTGCTGAAAGC<br>TCTCGTGCGGCAGCAGCTGCCTGAGAAGTACAAAGAGATTTTCTTC<br>GACCAGAGCAAGAACGGCTACGCCGGCTACATCGATGGCGGAGCC<br>AGCCAGGAAGAGTTCTACAAGTTCATCAAGCCCATCCTGGAAAAG<br>ATGGACGGCACCGAGGAACTGCTCGTGAAGCTGAACAGAGAGGA<br>CCTGCTGCGGAAGCAGCGGACCTTCGACAACGGCAGCATCCCCCA<br>CCAGATCCACCTGGGAGAGCTGCACGCCATTCTGCGGCGGCAGGA<br>AGATTTTTACCCATTCCTGAAGGACAACCGGGAAAAGATCGAGAA<br>GATCCTGACCTTCGCCATCCCCTACTACGTGGGCCCTCTGGCCAGG<br>GGAAACAGCAGATTCGCCTGGATGACCAGAAAGAGCGAGGAAAC<br>CATCACCCCCTGGAACTTCGAGGAAGTGGTGGACAAGGGCGCCAG<br>CGCCCAGAGCTTCATCGAGCGGATGACCAACTTCGATAAGAACCT<br>GCCCAACGAGAAGGTGCTGCCCAAGCACAGCCTGCTGTACGAGTA<br>CTTCACCGTGTACAACGAGCTGACCAAAGTGAAATACGTGACCGA<br>GGGAATGAGAAAGCCCGCCTTCCTGAGCGGCGAGCAGAAAAAG

	CCATCGTGGACCTGCTGTTCAAGACCAACCGGAAAGTGACCGTGA<br>AGCAGCTGAAAGAGGACTACTTCAAGAAAATCGAGTGCTTCGACT<br>CCGTGGAAATCTCCGGCGTGGAAGATCGGTTCAACGCCTCCCTGG<br>GCACATACCACGATCTGCTGAAAATTATCAAGGACAAGGACTTCC<br>TGGACAATGAGGAAAACGAGGACATTCTGGAAGATATCGTGCTGA<br>CCCTGACACTGTTTGAGGACAGAGAGATGATCGAGGAACGGCTGA<br>AAACCTATGCCCACCTGTTCGACGACAAAGTGATGAAGCAGCTGA<br>AGCGGCGGAGATACACCGGCTGGGGCAGGCTGAGCCGGAAGCTG<br>ATCAACGGCATCCGGGACAAGCAGTCCGGCAAGACAATCCTGGAT<br>TTCCTGAAGTCCGACGGCTTCGCCAACAGAAACTTCATGCAGCTGA<br>TCCACGACGACAGCCTGACCTTTAAAGAGGACATCCAGAAAGCCC<br>AGGTGTCCGGCCAGGGCGATAGCCTGCACGAGCACATTGCCAATC<br>TGGCCGGCAGCCCCGCCATTAAGAAGGGCATCCTGCAGACAGTGA<br>AGGTGGTGGACGAGCTCGTGAAAGTGATGGGCCGGCACAAGCCCG<br>AGAACATCGTGATCGAAATGGCCGCCGAGAACCAGACCACCCAGA<br>AGGGACAGAAGAACAGCCGCGAGAGAATGAAGCGGATCGAAGAG<br>GGCATCAAAGAGCTGGGCAGCCAGATCCTGAAAGAACACCCCGTG<br>GAAAACACCCAGCTGCAGAACGAGAAGCTGTACCTGTACTACCTG<br>CAGAATGGGCGGGATATGTACGTGGACCAGGAACTGGACATCAAC<br>CGGCTGTCCGACTACGATGTGGACCATATCGTGCCTCAGGCCTTTC<br>TGAAGGACGACTCCATCGATAACAAAGTGCTGACTCGGAGCGACA<br>AGAACCGGGGCAAGAGCGACAACGTGCCCTCCGAAGAGGTCGTG<br>AAGAAGATGAAGAACTACTGGCGCCAGCTGCTGAATGCCAAGCTG<br>ATTACCCAGAGGAAGTTCGACAATCTGACCAAGGCCGAGAGAGGC<br>GGCCTGAGCGAACTGGATAAGGCCGGCTTCATCAAGAGACAGCTG<br>GTGGAAACCCGGCAGATCACAAAGCACGTGGCACAGATCCTGGAC<br>TCCCGGATGAACACTAAGTACGACGAGAACGACAAACTGATCCGG<br>GAAGTGAAAGTGATCACCCTGAAGTCCAAGCTGGTGTCCGATTTC<br>CGGAAGGATTTCCAGTTTTACAAAGTGCGCGAGATCAACAACTAC<br>CACCACGCCCACGACGCCTACCTGAACGCCGTCGTGGGAACCGCC<br>CTGATCAAAAAGTACCCTAAGCTGGAAAGCGAGTTCGTGTACGGC<br>GACTACAAGGTGTACGACGTGCGGAAGATGATCGCCAAGAGCGAG<br>CAGGAAATCGGCAAGGCTACCGCCAAGTACTTCTTCTACAGCAAC<br>ATCATGAACTTTTTCAAGACCGAGATTACCCTGGCCAACGGCGAG<br>ATCCGGAAGCGGCCTCTGATCGAGACAAACGGCGAAACAGGCGA<br>GATCGTGTGGGATAAGGGCCGGGACTTTGCCACCGTGCGGAAAGT<br>GCTGTCTATGCCCCAAGTGAATATCGTGAAAAAGACCGAGGTGCA<br>GACAGGCGGCTTCAGCAAAGAGTCTATCCTGCCCAAGAGGAACAG<br>CGACAAGCTGATCGCCAGAAAGAAGGACTGGGACCCTAAGAAGT<br>ACGGCGGCTTCGACAGCCCCACCGTGGCCTATTCTGTGCTGGTGGT<br>GGCCAAAGTGGAAAAGGGCAAGTCCAAGAAACTGAAGAGTGTGA<br>AAGAGCTGCTGGGGATCACCATCATGGAAAGAAGCAGCTTCGAGA<br>AGAATCCCATCGACTTTCTGGAAGCCAAGGGCTACAAAGAAGTGA<br>AAAAGGACCTGATCATCAAGCTGCCTAAGTACTCCCTGTTCGAGCT<br>GGAAAACGGCCGGAAGAGAATGCTGGCCTCTGCCGGCGAACTGCA<br>GAAGGGAAACGAACTGGCCCTGCCCTCCAAATATGTGAACTTCCT

	GTACCTGGCCAGCCACTATGAGAAGCTGAAGGGCTCCCCCGAGGA<br>TAATGAGCAGAAACAGCTGTTTGTGGAACAGCACAAACACTACCT<br>GGACGAGATCATCGAGCAGATCAGCGAGTTCTCCAAGAGAGTGAT<br>CCTGGCCGACGCTAATCTGGACAAGGTGCTGAGCGCCTACAACAA<br>GCACAGAGACAAGCCTATCAGAGAGCAGGCCGAGAATATCATCCA<br>CCTGTTTACCCTGACCAATCTGGGAGCCCCTGCCGCCTTCAAGTAC<br>TTTGACACCACCATCGACCGGAAGAGGTACACCAGCACCAAAGAG<br>GTGCTGGACGCCACCCTGATCCACCAGAGCATCACCGGCCTGTAC<br>GAGACACGGATCGACCTGTCTCAGCTGGGAGGCGACGCCTATCCC<br>TATGACGTGCCCGATTATGCCAGCCTGGGCAGCGGCTCCCCCAAG<br>AAAAAACGCAAGGTGGAAGATCCTAAGAAAAAGCGGAAAGGTTC<br>TAGTGAAACCCCGGGAACAAGTGAGTCGGCCACCCCTGAAGGTGG<br>ATCAGGGGGTAGCGGATCCTCTTACGATAATTATGTTACGATTCTG<br>GACGAGGAAACCTTAAAGGCTTGGATCGCTAAATTAGAGAAGGCT<br>CCTGTTTTCGCTTTCGACACGGAAACGGATTCTCTGGACAATATTA<br>GTGCGAATCTTGTTGGTCTGAGTTTCGCAATTGAACCGGGTGTTGC<br>TGCTTACATCCCTGTGGCACACGACTACCTGGACGCTCCGGACCAG<br>ATTTCACGTGAACGCGCTCTGGAACTGCTGAAGCCTTTATTAGAGG<br>ACGAGAAAGCTTTGAAAGTTGGTCAGAATTTGAAGTATGCTCGTG<br>GAATCTTAGCTAATTATGGTATCGAGTTGCGCGGTATCGCTTTCGA<br>CACGATGTTGGAATCTTATATCCTGAACTCTGTCGCTGGTCGCCAT<br>GACATGGACTCTCTGGCTGAGCGCTGGCTGAAACATAAGACGATT<br>ACCTTCGAGGAAATCGCAGGAAAGGGTAAGAACCAGCTCACGTTC<br>AATCAAATCGCTCTGGAGGAAGCTGGTCGCTATGCTGCTGAGGAC<br>GCTGACGTTACTCTGCAACTGCACTTGAAGATGTGGCCTGACTTGC<br>AGAAGCATAAGGGTCCACTGAATGTTTTTGAAAACATTGAGATGC<br>CTTTGGTTCCAGTTCTGTCTCGTATCGAGCGCAATGGCGTTAAAAT<br>TGACCCAAAGGTTTTACATAACCACTCAGAGGAACTGACGCTGCG<br>CTTAGCCGAATTGGAGAAAAAGGCTCACGAGATCGCTGGCGAAGA<br>GTTCAATCTGTCATCTACGAAACAACTGCAGACTATCCTGTTCGAG<br>AAGCAAGGTATCAAGCCATTAAAAAAGACCCCTGGCGGTGCTCCG<br>TCTACCTCTGAGGAAGTTTTGGAGGAGTTAGCTTTGGATTACCCTC<br>TGCCGAAGGTTATCTTGGAATACCGCGGTTTGGCTAAATTGAAGTC<br>TACTTATACGGATAAACTTCCTTTGATGATTAATCCAAAGACGGGT<br>CGCGTTCACACGTCGTACCATCAAGCTGTTACCGCTACCGGTCGCC<br>TGTCTTCTACGGATCCGAATTTACAGAATATTCCTGTGCGCAATGA<br>GGAGGGCCGCCGCATTCGTCAAGCTTTTATCGCTCCGGAAGACTAC<br>GTTATCGTTTCTGCTGATTATTCTCAAAATGAATTACGTATCATGG<br>CTCACCTGTCTCGCGATAAGGGTCTGTTGACGGCCTTTGCTGAGGG<br>TAAGGACATTCATCGTGCTACCGCTGCTGAGGTTTACGGCCTGCCG<br>TTGGAAACGGTTACGTCTGAACAGCGTCGCTCTGCTAAGCGTATTA<br>ATTTCGGCTTAATCTACGGTATGTCTGCGTTTGGCTTAGCTCGTCA<br>GCTGAATATCCCGCGCAAGGAAGCTCAAAAATATATGGATCTGTA<br>TTTTGAGCGTTACCACGGTGTTTTGGAATACATGGAGCGTACGCGC<br>GCGCAAGCTAAGGAACAAGGTTATGTGGAAACCTTGGATGGTCGT<br>CGCTTGTACTTGCCTGACATTAAGTCTTCTAACGGCGCCCGCCGCG

	CTGCTGCCGAGCGCGCTGCTATCAATGCTCCGATGCAAGGTACTGC<br>TGCTGATATTATTAAGCGTGCTATGATCGCTGTGGACGCTTGGCTG<br>CAAGCTGAACAGCCTCGCGTTCGCATGATTATGCAAGTTCATGACG<br>AGTTGGTTTTCGAGGTGCATAAGGACGACGTGGACGCTGTTGCTA<br>AACAAATCCACCAGTTGATGGAGAATTGCACGCGCTTAGACGTTC<br>CGCTGCTGGTTGAAGTTGGTTCTGGTGAAAACTGGGACCAGGCTC<br>ACTAATGGCTGAAAACTGGGACCAGGCTCACTAACTCAGATCCTA<br>CTAGGTTTAATAAACATCTTTATTTTCATTACATCTGTGTGTTGGTT<br>TTTTGTGTGGTACCCAATTCGCCCTATAGTGAGTCGTATTACTCACT<br>GGCCGTCGTTTTACAACGTCGTGACTGGGAAAACCCTGGCGTTACC<br>CAACTTAATCGCCTTGCAGCACATCCCCCTTTCGCCAGCTGGCGTA<br>ATAGCGAAGAGGCCCGCACCGATCGCCCTTCCCAACAGTTGCGCA<br>GCCTGAATGGCGAATGGGACGCGCCCTGTAGCGGCGCATTAAGCG<br>CGGCGGGTGTGGTGGTTACGCGCAGCGTGACCGCTACACTTGCCA<br>GCGCCCTAGCGCCCGCTCCTTTCGCTTTCTTCCCTTCCTTTCTCGCC<br>ACGTTCGCCGGCTTTCCCCGTCAAGCTCTAAATCGGGGGCTCCCTT<br>TAGGGTTCCGATTTAGTGCTTTACGGCACCTCGACCCCAAAAAACT<br>TGATTAGGGTGATGGTTCACGTAGTGGGCCATCGCCCTGATAGAC<br>GGTTTTTCGCCCTTTGACGTTGGAGTCCACGTTCTTTAATAGTGGA<br>CTCTTGTTCCAAACTGGAACAACACTCAACCCTATCTCGGTCTATT<br>CTTTTGATTTATAAGGGATTTTGCCGATTTCGGCCTATTGGTTAAA<br>AAATGAGCTGATTTAACAAAAATTTAACGCGAATTTTAACAAAAT<br>ATTAACGCTTACAATTTAGGTGGCACTTTTCGGGGAAATGTG<br><br>**Ampicillin resistance cassette-ColE1 origin of replication-hU6 promoter-gRNA sequence-SpCas9 gRNA scaffold-CMV promoter-NLS-mCherry-GSGM- HSC1.2-nCas9(D10A)-NLS-linker-PolI5MΔ-stop-SV40 polyA**
p.LZ3-nCas9-PolI5MΔ	CGCGGAACCCCTATTTGTTTATTTTTCTAAATACATTCAAATATGTA<br>TCCGCTCATGAGACAATAACCCTGATAAATGCTTCAATAATATTGA<br>AAAAGGAAGAGTATGAGTATTCAACATTTCCGTGTCGCCCTTATTC<br>CCTTTTTTGCGGCATTTTGCCTTCCTGTTTTTGCTCACCCAGAAACG<br>CTGGTGAAAGTAAAAGATGCTGAAGATCAGTTGGGTGCACGAGTG<br>GGTTACATCGAACTGGATCTCAACAGCGGTAAGATCCTTGAGAGT<br>TTTCGCCCCGAAGAACGTTTTCCAATGATGAGCACTTTTAAAGTTC<br>TGCTATGTGGCGCGGTATTATCCCGTATTGACGCCGGGCAAGAGC<br>AACTCGGTCGCCGCATACACTATTCTCAGAATGACTTGGTTGAGTA<br>CTCACCAGTCACAGAAAAGCATCTTACGGATGGCATGACAGTAAG<br>AGAATTATGCAGTGCTGCCATAACCATGAGTGATAACACTGCGGC<br>CAACTTACTTCTGACAACGATCGGAGGACCGAAGGAGCTAACCGC<br>TTTTTTGCACAACATGGGGGATCATGTAACTCGCCTTGATCGTTGG<br>GAACCGGAGCTGAATGAAGCCATACCAAACGACGAGCGTGACACC<br>ACGATGCCTGTAGCAATGGCAACAACGTTGCGCAAACTATTAACT<br>GGCGAACTACTTACTCTAGCTTCCCGGCAACAATTAATAGACTGGA<br>TGGAGGCGGATAAAGTTGCAGGACCACTTCTGCGCTCGGCCCTTCC

	GGCTGGCTGGTTTATTGCTGATAAATCTGGAGCCGGTGAGCGTGGT TCTCGCGGTATCATTGCAGCACTGGGGCCAGATGGTAAGCCCTCCC GTATCGTAGTTATCTACACGACGGGGAGTCAGGCAACTATGGATG AACGAAATAGACAGATCGCTGAGATAGGTGCCTCACTGATTAAGC ATTGGTAACTGTCAGACCAAGTTTACTCATATATACTTTAGATTGA TTTAAAACTTCATTTTTAATTTAAAAGGATCTAGGTGAAGATCCTT TTTGATAATCTCATGACCAAAATCCCTTAACGTGAGTTTTCGTTCC ACTGAGCGTCAGACCCCGTAGAAAAGATCAAAGGATCTTCTTGAG ATCCTTTTTTTCTGCGCGTAATCTGCTGCTTGCAAACAAAAAAACC ACCGCTACCAGCGGTGGTTTGTTTGCCGGATCAAGAGCTACCAACT CTTTTTCCGAAGGTAACTGGCTTCAGCAGAGCGCAGATACCAAAT ACTGTCCTTCTAGTGTAGCCGTAGTTAGGCCACCACTTCAAGAACT CTGTAGCACCGCCTACATACCTCGCTCTGCTAATCCTGTTACCAGT GGCTGCTGCCAGTGGCGATAAGTCGTGTCTTACCGGGTTGGACTCA AGACGATAGTTACCGGATAAGGCGCAGCGGTCGGGCTGAACGGGG GGTTCGTGCACACAGCCCAGCTTGGAGCGAACGACCTACACCGAA CTGAGATACCTACAGCGTGAGCTATGAGAAAGCGCCACGCTTCCC GAAGGGAGAAAGGCGGACAGGTATCCGGTAAGCGGCAGGGTCGG AACAGGAGAGCGCACGAGGGAGCTTCCAGGGGGAAACGCCTGGT ATCTTTATAGTCCTGTCGGGTTTCGCCACCTCTGACTTGAGCGTCG ATTTTTGTGATGCTCGTCAGGGGGGCGGAGCCTATGGAAAAACGC CAGCAACGCGGCCTTTTTACGGTTCCTGGCCTTTTGCTGGCCTTTTG CTCACATGTTCTTTCCTGCGTTATCCCCTGATTCTGTGGATAACCGT ATTACCGCCTTTGAGTGAGCTGATACCGCTCGCCGCAGCCGAACG ACCGAGCGCAGCGAGTCAGTGAGCGAGGAAGCGGAAGAGCGCCC AATACGCAAACCGCCTCTCCCCGCGCGTTGGCCGATTCATTAATGC AGCTGGCACGACAGGTTTCCCGACTGGAAAGCGGGCAGTGAGCGC AACGCAATTAATGTGAGTTAGCTCACTCATTAGGCACCCCAGGCTT TACACTTTATGCTTCCGGCTCGTATGTTGTGTGGAATTGTGAGCGG ATAACAATTTCACACAGGAAACAGCTATGACCATGATTACGCCAA GCGCGCAATTAACCCTCACTAAAGGGAACAAAAGCTGGAGCTCCA CCGCGGTGGCGGCCGCTCTTAAGGGGTGCAGCGGCCTCCGCGCCG GGTTTTGGCGCCTCGATCCAAGGTCGGGCAGGAAGAGGGCCTATT TCCCATGATTCCTTCATATTTGCATATACGATACAAGGCTGTTAGA GAGATAATTAGAATTAATTTGACTGTAAACACAAAGATATTAGTA CAAAATACGTGACGTAGAAAGTAATAATTTCTTGGGTAGTTTGCA GTTTTAAAATTATGTTTTAAAATGGACTATCATATGCTTACCGTAA CTTGAAAGTATTTCGATTTCTTGGCTTTATATATCTTGTGGAAAGG ACGAAACACC-[gRNA SEQUENCE]- GTTTTAGAGCTAGAAATAGCAAGTTAAAATAAGGCTAGTCCGTTA TCAACTTGAAAAAGTGGCACCGAGTCGGTGCTTTTTTCCGCGGCCT CTAGACTCGAGGCGTTGACATTGATTATTGACTAGTTATTAATAGT AATCAATTACGGGGTCATTAGTTCATAGCCCATATATGGAGTTCCG CGTTACATAACTTACGGTAAATGGCCCGCCTGGCTGACCGCCCAAC GACCCCCGCCCATTGACGTCAATAATGACGTATGTTCCCATAGTAA CGCCAATAGGGACTTTCCATTGACGTCAATGGGTGGAGTATTTACG

	GTAAACTGCCCACTTGGCAGTACATCAAGTGTATCATATGCCAAGT<br>ACGCCCCCTATTGACGTCAATGACGGTAAATGGCCCGCCTGGCATT<br>ATGCCCAGTACATGACCTTATGGGACTTTCCTACTTGGCAGTACAT<br>CTACGTATTAGTCATCGCTATTACCATGGTGATGCGGTTTTGGCAG<br>TACATCAATGGGCGTGGATAGCGGTTTGACTCACGGGGATTTCCA<br>AGTCTCCACCCCATTGACGTCAATGGGAGTTTGTTTTGGCACCAAA<br>ATCAACGGGACTTTCCAAAATGTCGTAACAACTCCGCCCCATTGAC<br>GCAAATGGGCGGTAGGCGTGTACGGTGGGAGGTCTATATAAGCAG<br>AGCTCTCTGGCTAACTACCGGTGCCACCATGGCCCCAAAGAAGAA<br>GCGGAAGGTCGGTATCCACGGAGTCCCAGCAGCCGTGAGCAAGGG<br>CGAGGAGGATAACATGGCCATCATCAAGGAGTTCATGCGCTTCAA<br>GGTGCACATGGAGGGCTCCGTGAACGGCCACGAGTTCGAGATCGA<br>GGGCGAGGGCGAGGGCCGCCCCTACGAGGGCACCCAGACCGCCA<br>AGCTGAAGGTGACCAAGGGTGGCCCCCTGCCCTTCGCCTGGGACA<br>TCCTGTCCCCTCAGTTCATGTACGGCTCCAAGGCCTACGTGAAGCA<br>CCCCGCCGACATCCCCGACTACTTGAAGCTGTCCTTCCCCGAGGGC<br>TTCAAGTGGGAGCGCGTGATGAACTTCGAGGACGGCGGCGTGGTG<br>ACCGTGACCCAGGACTCCTCCCTGCAGGACGGCGAGTTCATCTAC<br>AAGGTGAAGCTGCGCGGCACCAACTTCCCCTCCGACGGCCCCGTA<br>ATGCAGAAGAAGACCATGGGCTGGGAGGCCTCCTCCGAGCGGATG<br>TACCCCGAGGACGGCGCCCTGAAGGGCGAGATCAAGCAGAGGCTG<br>AAGCTGAAGGACGGCGGCCACTACGACGCTGAGGTCAAGACCACC<br>TACAAGGCCAAGAAGCCCGTGCAGCTGCCCGGCGCCTACAACGTC<br>AACATCAAGTTGGACATCACCTCCCACAACGAGGACTACACCATC<br>GTGGAACAGTACGAACGCGCCGAGGGCCGCCACTCCACCGGCGGC<br>ATGGACGAGCTGTACAAGGGATCCGGTATGGACAAGAAGTACAGC<br>ATCGGCCTGGCCATCGGCACCAACTCTGTGGGCTGGGCCGTGATC<br>ACCGACGAGTACAAGGTGCCCAGCAAGAAATTCAAGGTGCTGGGC<br>AACACCGACCGGCACAGCATCAAGAAGAACCTGATCGGCGCCCTG<br>CTGTTCGACAGCGGAGAAACAGCCGAGGCCACCCGGCTGAAGAGA<br>ACCGCCAGAAGAAGATACACCAGACGGAAGAACCGGATCTGCTAT<br>CTGCAAGAGATCTTCAGCAACGAGATGGCCAAGGTGGACGACAGC<br>TTCTTCCACAGACTGGAAGAGTCCTTCCTGGTGGAAGAGGATAAG<br>AAGCACGAGCGGCACCCCATCTTCGGCAACATCGTGGACGAGGTG<br>GCCTACCACGAGAAGTACCCCACCATCTACCACCTGAGAAAGAAA<br>CTGGTGGACAGCACCGACAAGGCCGACCTGCGGCTGATCTATCTG<br>GCCCTGGCCCACATGATCAAGTTCCGGGGCCACTTCCTGATCGAGG<br>GCGACCTGAACCCCGACAACAGCGACGTGGACAAGCTGTTCATCC<br>AGCTGGTGCAGACCTACAACCAGCTGTTCGAGGAAAACCCCATCA<br>ACGCCAGCGGCGTGGACGCCAAGGCCATCCTGTCTGCCAGACTGA<br>GCAAGAGCAGACGGCTGGAAAATCTGATCGCCCAGCTGCCCGGCG<br>AGAAGAAGAATGGCCTGTTCGGCAACCTGATTGCCCTGAGCCTGG<br>GCCTGACCCCCAACTTCAAGAGCAACTTCGACCTGGCCGAGGATG<br>CCAAACTGCAGCTGAGCAAGGACACCTACGACGACGACCTGGACA<br>ACCTGCTGGCCCAGATCGGCGACCAGTACGCCGACCTGTTTCTGGC<br>CGCCAAGAACCTGTCCGACGCCATCCTGCTGAGCGACATCCTGAG

		AGTGAACACCGAGATCACCAAGGCCCCCCTGAGCGCCTCTATGAT<br>CAAGAGATACGACGAGCACCACCAGGACCTGACCCTGCTGAAAGC<br>TCTCGTGCGGCAGCAGCTGCCTGAGAAGTACAAAGAGATTTTCTTC<br>GACCAGAGCAAGAACGGCTACGCCGGCTACATCGATGGCGGAGCC<br>AGCCAGGAAGAGTTCTACAAGTTCATCAAGCCCATCCTGGAAAAG<br>ATGGACGGCACCGAGGAACTGCTCGTGAAGCTGAACAGAGAGGA<br>CCTGCTGCGGAAGCAGCGGACCTTCGACAACGGCAGCATCCCCCA<br>CCAGATCCACCTGGGAGAGCTGCACGCCATTCTGCGGCGGCAGGA<br>AGATTTTTACCCATTCCTGAAGGACAACCGGGAAAAGATCGAGAA<br>GATCCTGACCTTCCGCATCCCCTACTACGTGGGCCCTCTGGCCAGG<br>GGAAACAGCAGATTCGCCTGGATGACCAGAAAGAGCGAGGAAAC<br>CATCACCCCCTGGAACTTCGAGGAAGTGGTGGACAAGGGCGCCAG<br>CGCCCAGAGCTTCATCGAGCGGATGACCAACTTCGATAAGAACCT<br>GCCCAACGAGAAGGTGCTGCCCAAGCACAGCCTGCTGTACGAGTA<br>CTTCACCGTGTACAACGAGCTGACCAAAGTGAAATACGTGACCGA<br>GGGAATGAGAAAGCCCGCCTTCCTGAGCGGCGAGCAGAAAAAAG<br>CCATCGTGGACCTGCTGTTCAAGACCAACCGGAAAGTGACCGTGA<br>AGCAGCTGAAAGAGGACTACTTCAAGAAAATCGAGTGCTTCGACT<br>CCGTGGAAATCTCCGGCGTGGAAGATCGGTTCAACGCCTCCCTGG<br>GCACATACCACGATCTGCTGAAAATTATCAAGGACAAGGACTTCC<br>TGGACAATGAGGAAAACGAGGACATTCTGGAAGATATCGTGCTGA<br>CCCTGACACTGTTTGAGGACAGAGAGATGATCGAGGAACGGCTGA<br>AAACCTATGCCCACCTGTTCGACGACAAAGTGATGAAGCAGCTGA<br>AGCGGCGGAGATACACCGGCTGGGGCAGGCTGAGCCGGAAGCTG<br>ATCAACGGCATCCGGGACAAGCAGTCCGGCAAGACAATCCTGGAT<br>TTCCTGAAGTCCGACGGCTTCGCCTGCAGAAACTTCATGCAGCTGA<br>TCCACGACGACAGCCTGACCTTTAAAGAGGACATCCAGAAAGCCC<br>AGGTGTCCGGCCAGGGCGATAGCCTGCACGAGCACATTGCCAATC<br>TGGCCGGCAGCCCCGCCATTAAGAAGGGCATCCTGCAGACAGTGA<br>AGGTGGTGGACGAGCTCGTGAAAGTGATGGGCCGGCACAAGCCCG<br>AGAACATCGTGATCGAAATGGCCAGAGAGAACCAGATCACCCAGA<br>AGGGACAGAAGAACAGCCGCGAGAGAATGAAGCGGATCGAAGAG<br>GGCATCAAAGAGCTGGGCAGCCAGATCCTGAAAGAACACCCCGTG<br>GAAAACACCCAGCTGCAGAACGAGAAGCTGTACCTGTACTACCTG<br>CAGAATGGGCGGGATATGTACGTGGACCAGGAACTGGACATCAAC<br>CGGCTGTCCGACTACGATGTGGACCATATCGTGCCTCAGAGCTTTC<br>TGAAGGACGACTCCATCGATAACAAAGTGCTGACTCGGAGCGACA<br>AGAACCGGGGCAAGAGCGACAACGTGCCCTCCGAAGAGGTCGTG<br>AAGAAGATGAAGAACTACTGGCGCCAGCTGCTGAATGCCAAGCTG<br>ATTACCCAGAGGAAGTTCGACAATCTGACCAAGGCCGAGAGAGGC<br>GGCCTGAGCGAACTGGATAAGGCCATGTTCATCAAGAGACAGCTG<br>GTGGAAACCCGGCAGATCACAAAGCACGTGGCACAGATCCTGGAC<br>TCCCGGATGAACACTAAGTACGACGAGAACGACAAACTGATCCGG<br>GAAGTGAAAGTGATCACCCTGAAGTCCAAGCTGGTGTCCGATTTC<br>CGGAAGGATTTCCAGTTTTACAAAGTGCGCGAGATCAACAAGTAC<br>CACCACGCCCACGACGCCTACCTGAACGCCGTCGTGGAACCGCC

	CTGATCAAAAAGTACCCTAAGCTGGAAAGCGAGTTCGTGTACGGC<br>GACTACAAGGTGTACGACGTGCGGAAGATGATCGCCAAGAGCGAG<br>CAGGAAATCGGCAAGGCTACCGCCAAGTACTTCTTCTACAGCAAC<br>ATCATGAACTTTTTCAAGACCGAGATTACCCTGGCCAACGGCGAG<br>ATCCGGAAGCGGCCTCTGATCGAGACAAACGGCGAAACAGGCGA<br>GATCGTGTGGGATAAGGGCCGGGACTTTGCCACCGTGCGGAAAGT<br>GCTGTCTATGCCCCAAGTGAATATCGTGAAAAAGACCGAGGTGCA<br>GACAGGCGGCTTCAGCAAAGAGTCTATCCTGCCCAAGAGGAACAG<br>CGACAAGCTGATCGCCAGAAAGAAGGACTGGGACCCTAAGAAGT<br>ACGGCGGCTTCGACAGCCCCACCGTGGCCTATTCTGTGCTGGTGGT<br>GGCCAAAGTGGAAAAGGGCAAGTCCAAGAAACTGAAGAGTGTGA<br>AAGAGCTGCTGGGGATCACCATCATGGAAAGAAGCAGCTTCGAGA<br>AGAATCCCATCGACTTTCTGGAAGCCAAGGGCTACAAAGAAGTGA<br>AAAAGGACCTGATCATCAAGCTGCCTAAGTACTCCCTGTTCGAGCT<br>GGAAAACGGCCGGAAGAGAATGCTGGCCTCTGCCGGCGAACTGCA<br>GAAGGGAAACGAACTGGCCCTGCCCTCCAAATATGTGAACTTCCT<br>GTACCTGGCCAGCCACTATGAGAAGCTGAAGGGCTCCCCCGAGGA<br>TAATGAGCAGAAACAGCTGTTTGTGGAACAGCACAAACACTACCT<br>GGACGAGATCATCGAGCAGATCAGCGAGTTCTCCAAGAGAGTGAT<br>CCTGGCCGACGCTAATCTGGACAAGGTGCTGAGCGCCTACAACAA<br>GCACAGAGACAAGCCTATCAGAGAGCAGGCCGAGAATATCATCCA<br>CCTGTTTACCCTGACCAATCTGGGAGCCCCTGCCGCCTTCAAGTAC<br>TTTGACACCACCATCGACCGGAAGAGGTACACCAGCACCAAAGAG<br>GTGCTGGACGCCACCCTGATCCACCAGAGCATCACCGGCCTGTAC<br>GAGACACGGATCGACCTGTCTCAGCTGGGAGGCGACGCCTATCCC<br>TATGACGTGCCCGATTATGCCAGCCTGGGCAGCGGCTCCCCCAAG<br>AAAAAACGCAAGGTGGAAGATCCTAAGAAAAAGCGGAAAGGTTC<br>TAGTGAAACCCCGGGAACAAGTGAGTCGGCCACCCCTGAAGGTGG<br>ATCAGGGGGTAGCGGATCCTCTTACGATAATTATGTTACGATTCTG<br>GACGAGGAAACCTTAAAGGCTTGGATCGCTAAATTAGAGAAGGCT<br>CCTGTTTTCGCTTTCGACACGGAAACGGATTCTCTGGACAATATTA<br>GTGCGAATCTTGTTGGTCTGAGTTTCGCAATTGAACCGGGTGTTGC<br>TGCTTACATCCCTGTGGCACACGACTACCTGGACGCTCCGGACCAG<br>ATTTCACGTGAACGCGCTCTGGAACTGCTGAAGCCTTTATTAGAGG<br>ACGAGAAAGCTTTGAAAGTTGGTCAGAATTTGAAGTATGCTCGTG<br>GAATCTTAGCTAATTATGGTATCGAGTTGCGCGGTATCGCTTTCGA<br>CACGATGTTGGAATCTTATATCCTGAACTCTGTCGCTGGTCGCCAT<br>GACATGGACTCTCTGGCTGAGCGCTGGCTGAAACATAAGACGATT<br>ACCTTCGAGGAAATCGCAGGAAAGGGTAAGAACCAGCTCACGTTC<br>AATCAAATCGCTCTGGAGGAAGCTGGTCGCTATGCTGCTGAGGAC<br>GCTGACGTTACTCTGCAACTGCACTTGAAGATGTGGCCTGACTTGC<br>AGAAGCATAAGGGTCCACTGAATGTTTTTGAAAACATTGAGATGC<br>CTTTGGTTCCAGTTCTGTCTCGTATCGAGCGCAATGGCGTTAAAAT<br>TGACCCAAAGGTTTTACATAACCACTCAGAGGAACTGACGCTGCG<br>CTTAGCCGAATTGGAGAAAAAGGCTCACGAGATCGCTGGCGAAGA<br>GTTCAATCTGTCATCTACGAAACAACTGCAGACTATCCTGTTCGAG

	AAGCAAGGTATCAAGCCATTAAAAAAGACCCCTGGCGGTGCTCCG<br>TCTACCTCTGAGGAAGTTTTGGAGGAGTTAGCTTTGGATTACCCTC<br>TGCCGAAGGTTATCTTGGAATACCGCGGTTTGGCTAAATTGAAGTC<br>TACTTATACGGATAAACTTCCTTTGATGATTAATCCAAAGACGGGT<br>CGCGTTCACACGTCGTACCATCAAGCTGTTACCGCTACCGGTCGCC<br>TGTCTTCTACGGATCCGAATTTACAGAATATTCCTGTGCGCAATGA<br>GGAGGGCCGCCGCATTCGTCAAGCTTTTATCGCTCCGGAAGACTAC<br>GTTATCGTTTCTGCTGATTATTCTCAAAATGAATTACGTATCATGG<br>CTCACCTGTCTCGCGATAAGGGTCTGTTGACGGCCTTTGCTGAGGG<br>TAAGGACATTCATCGTGCTACCGCTGCTGAGGTTTACGGCCTGCCG<br>TTGGAAACGGTTACGTCTGAACAGCGTCGCTCTGCTAAGCGTATTA<br>ATTTCGGCTTAATCTACGGTATGTCTGCGTTTGGCTTAGCTCGTCA<br>GCTGAATATCCCGCGCAAGGAAGCTCAAAAATATATGGATCTGTA<br>TTTTGAGCGTTACCACGGTGTTTTGGAATACATGGAGCGTACGCGC<br>GCGCAAGCTAAGGAACAAGGTTATGTGGAAACCTTGGATGGTCGT<br>CGCTTGTACTTGCCTGACATTAAGTCTTCTAACGGCGCCCGCCGCG<br>CTGCTGCCGAGCGCGCTGCTATCAATGCTCCGATGCAAGGTACTGC<br>TGCTGATATTATTAAGCGTGCTATGATCGCTGTGGACGCTTGGCTG<br>CAAGCTGAACAGCCTCGCGTTCGCATGATTATGCAAGTTCATGACG<br>AGTTGGTTTTCGAGGTGCATAAGGACGACGTGGACGCTGTTGCTA<br>AACAAATCCACCAGTTGATGGAGAATTGCACGCGCTTAGACGTTC<br>CGCTGCTGGTTGAAGTTGGTTCTGGTGAAAACTGGGACCAGGCTC<br>ACTAATGGCTGAAAACTGGGACCAGGCTCACTAACTCAGATCCTA<br>CTAGGTTTAATAAACATCTTTATTTTCATTACATCTGTGTGTTGGTT<br>TTTTGTGTGGTACCCAATTCGCCCTATAGTGAGTCGTATTACTCACT<br>GGCCGTCGTTTTACAACGTCGTGACTGGGAAAACCCTGGCGTTACC<br>CAACTTAATCGCCTTGCAGCACATCCCCCTTTCGCCAGCTGGCGTA<br>ATAGCGAAGAGGCCCGCACCGATCGCCCTTCCCAACAGTTGCGCA<br>GCCTGAATGGCGAATGGACGCGCCCTGTAGCGGCGCATTAAGCG<br>CGGCGGGTGTGGTGGTTACGCGCAGCGTGACCGCTACACTTGCCA<br>GCGCCCTAGCGCCCGCTCCTTTCGCTTTCTTCCCTTCCTTTCTCGCC<br>ACGTTCGCCGGCTTTCCCCGTCAAGCTCTAAATCGGGGGCTCCCTT<br>TAGGGTTCCGATTTAGTGCTTTACGGCACCTCGACCCCAAAAAACT<br>TGATTAGGGTGATGGTTCACGTAGTGGGCCATCGCCCTGATAGAC<br>GGTTTTTCGCCCTTTGACGTTGGAGTCCACGTTCTTTAATAGTGGA<br>CTCTTGTTCCAAACTGGAACAACACTCAACCCTATCTCGGTCTATT<br>CTTTTGATTTATAAGGGATTTTGCCGATTTCGGCCTATTGGTTAAA<br>AAATGAGCTGATTTAACAAAAATTTAACGCGAATTTTAACAAAAT<br>ATTAACGCTTACAATTTAGGTGGCACTTTTCGGGGAAATGTG<br><br>**Ampicillin resistance cassette-ColE1 origin of replication-hU6 promoter-gRNA sequence-SpCas9 gRNA scaffold-CMV promoter-NLS-mCherry-GSGM- LZ3-nCas9(D10A)-NLS-PolI5MΔ-stop-SV40 polyA**

p.SpRY-<br>nCas9-<br>PolI5MΔ	CGCGGAACCCCTATTTGTTTATTTTTCTAAATACATTCAAATATGTA<br>TCCGCTCATGAGACAATAACCCTGATAAATGCTTCAATAATATTGA<br>AAAAGGAAGAGTATGAGTATTCAACATTTCCGTGTCGCCCTTATTC<br>CCTTTTTTGCGGCATTTTGCCTTCCTGTTTTTGCTCACCCAGAAACG<br>CTGGTGAAAGTAAAAGATGCTGAAGATCAGTTGGGTGCACGAGTG<br>GGTTACATCGAACTGGATCTCAACAGCGGTAAGATCCTTGAGAGT<br>TTTCGCCCCGAAGAACGTTTTCCAATGATGAGCACTTTTAAAGTTC<br>TGCTATGTGGCGCGGTATTATCCCGTATTGACGCCGGGCAAGAGC<br>AACTCGGTCGCCGCATACACTATTCTCAGAATGACTTGGTTGAGTA<br>CTCACCAGTCACAGAAAAGCATCTTACGGATGGCATGACAGTAAG<br>AGAATTATGCAGTGCTGCCATAACCATGAGTGATAACACTGCGGC<br>CAACTTACTTCTGACAACGATCGGAGGACCGAAGGAGCTAACCGC<br>TTTTTTGCACAACATGGGGGATCATGTAACTCGCCTTGATCGTTGG<br>GAACCGGAGCTGAATGAAGCCATACCAAACGACGAGCGTGACACC<br>ACGATGCCTGTAGCAATGGCAACAACGTTGCGCAAACTATTAACT<br>GGCGAACTACTTACTCTAGCTTCCCGGCAACAATTAATAGACTGGA<br>TGGAGGCGGATAAAGTTGCAGGACCACTTCTGCGCTCGGCCCTTCC<br>GGCTGGCTGGTTTATTGCTGATAAATCTGGAGCCGGTGAGCGTGGT<br>TCTCGCGGTATCATTGCAGCACTGGGGCCAGATGGTAAGCCCTCCC<br>GTATCGTAGTTATCTACACGACGGGGAGTCAGGCAACTATGGATG<br>AACGAAATAGACAGATCGCTGAGATAGGTGCCTCACTGATTAAGC<br>ATTGGTAACTGTCAGACCAAGTTTACTCATATATACTTTAGATTGA<br>TTTAAAACTTCATTTTTAATTTAAAAGGATCTAGGTGAAGATCCTT<br>TTTGATAATCTCATGACCAAAATCCCTTAACGTGAGTTTTCGTTCC<br>ACTGAGCGTCAGACCCCGTAGAAAAGATCAAAGGATCTTCTTGAG<br>ATCCTTTTTTTCTGCGCGTAATCTGCTGCTTGCAAACAAAAAAACC<br>ACCGCTACCAGCGGTGGTTTGTTTGCCGGATCAAGAGCTACCAACT<br>CTTTTTCCGAAGGTAACTGGCTTCAGCAGAGCGCAGATACCAAAT<br>ACTGTCCTTCTAGTGTAGCCGTAGTTAGGCCACCACTTCAAGAACT<br>CTGTAGCACCGCCTACATACCTCGCTCTGCTAATCCTGTTACCAGT<br>GGCTGCTGCCAGTGGCGATAAGTCGTGTCTTACCGGGTTGGACTCA<br>AGACGATAGTTACCGGATAAGGCGCAGCGGTCGGGCTGAACGGGG<br>GGTTCGTGCACACAGCCCAGCTTGGAGCGAACGACCTACACCGAA<br>CTGAGATACCTACAGCGTGAGCTATGAGAAAGCGCCACGCTTCCC<br>GAAGGGAGAAAGGCGGACAGGTATCCGGTAAGCGGCAGGGTCGG<br>AACAGGAGAGCGCACGAGGGAGCTTCCAGGGGGAAACGCCTGGT<br>ATCTTTATAGTCCTGTCGGGTTTCGCCACCTCTGACTTGAGCGTCG<br>ATTTTTGTGATGCTCGTCAGGGGGGCGGAGCCTATGGAAAAACGC<br>CAGCAACGCGGCCTTTTTACGGTTCCTGGCCTTTTGCTGGCCTTTTG<br>CTCACATGTTCTTTCCTGCGTTATCCCCTGATTCTGTGGATAACCGT<br>ATTACCGCCTTTGAGTGAGCTGATACCGCTCGCCGCAGCCGAACG<br>ACCGAGCGCAGCGAGTCAGTGAGCGAGGAAGCGGAAGAGCGCCC<br>AATACGCAAACCGCCTCTCCCCGCGCGTTGGCCGATTCATTAATGC<br>AGCTGGCACGACAGGTTTCCCGACTGGAAAGCGGGCAGTGAGCGC<br>AACGCAATTAATGTGAGTTAGCTCACTCATTAGGCACCCCAGGCTT

	TACACTTTATGCTTCCGGCTCGTATGTTGTGTGGAATTGTGAGCGG<br>ATAACAATTTCACACAGGAAACAGCTATGACCATGATTACGCCAA<br>GCGCGCAATTAACCCTCACTAAAGGGAACAAAAGCTGGAGCTCCA<br>CCGCGGTGGCGGCCGCTCTTAAGGGGTGCAGCGGCCTCCGCGCCG<br>GGTTTTGGCGCCTCGATCCAAGGTCGGGCAGGAAGAGGGCCTATT<br>TCCCATGATTCCTTCATATTTGCATATACGATACAAGGCTGTTAGA<br>GAGATAATTAGAATTAATTTGACTGTAAACACAAAGATATTAGTA<br>CAAAATACGTGACGTAGAAAGTAATAATTTCTTGGGTAGTTTGCA<br>GTTTTAAAATTATGTTTTAAAATGGACTATCATATGCTTACCGTAA<br>CTTGAAAGTATTTCGATTTCTTGGCTTTATATATCTTGTGGAAAGG<br>ACGAAACACC-[gRNA SEQUENCE]-<br>GTTTTAGAGCTAGAAATAGCAAGTTAAAATAAGGCTAGTCCGTTA<br>TCAACTTGAAAAAGTGGCACCGAGTCGGTGCTTTTTTCCGCGGCCT<br>CTAGACTCGAGGCGTTGACATTGATTATTGACTAGTTATTAATAGT<br>AATCAATTACGGGGTCATTAGTTCATAGCCCATATATGGAGTTCCG<br>CGTTACATAACTTACGGTAAATGGCCCGCCTGGCTGACCGCCCAAC<br>GACCCCCGCCCATTGACGTCAATAATGACGTATGTTCCCATAGTAA<br>CGCCAATAGGGACTTTCCATTGACGTCAATGGGTGGAGTATTTACG<br>GTAAACTGCCCACTTGGCAGTACATCAAGTGTATCATATGCCAAGT<br>ACGCCCCCTATTGACGTCAATGACGGTAAATGGCCCGCCTGGCATT<br>ATGCCCAGTACATGACCTTATGGGACTTTCCTACTTGGCAGTACAT<br>CTACGTATTAGTCATCGCTATTACCATGGTGATGCGGTTTTGGCAG<br>TACATCAATGGGCGTGGATAGCGGTTTGACTCACGGGGATTTCCA<br>AGTCTCCACCCCATTGACGTCAATGGGAGTTTGTTTTGGCACCAAA<br>ATCAACGGGACTTTCCAAAATGTCGTAACAACTCCGCCCCATTGAC<br>GCAAATGGGCGGTAGGCGTGTACGGTGGGAGGTCTATATAAGCAG<br>AGCTCTCTGGCTAACTACCGGTGCCACCATGGCCCCAAAGAAGAA<br>GCGGAAGGTCGGTATCCACGGAGTCCCAGCAGCCGTGAGCAAGGG<br>CGAGGAGGATAACATGGCCATCATCAAGGAGTTCATGCGCTTCAA<br>GGTGCACATGGAGGGCTCCGTGAACGGCCACGAGTTCGAGATCGA<br>GGGCGAGGGCGAGGGCCGCCCCTACGAGGGCACCCAGACCGCCA<br>AGCTGAAGGTGACCAAGGGTGGCCCCCTGCCCTTCGCCTGGGACA<br>TCCTGTCCCCTCAGTTCATGTACGGCTCCAAGGCCTACGTGAAGCA<br>CCCCGCCGACATCCCCGACTACTTGAAGCTGTCCTTCCCCGAGGGC<br>TTCAAGTGGGAGCGCGTGATGAACTTCGAGGACGGCGGCGTGGTG<br>ACCGTGACCCAGGACTCCTCCCTGCAGGACGGCGAGTTCATCTAC<br>AAGGTGAAGCTGCGCGGCACCAACTTCCCCTCCGACGGCCCCGTA<br>ATGCAGAAGAAGACCATGGGCTGGGAGGCCTCCTCCGAGCGGATG<br>TACCCCGAGGACGGCGCCCTGAAGGGCGAGATCAAGCAGAGGCTG<br>AAGCTGAAGGACGGCGGCCACTACGACGCTGAGGTCAAGACCACC<br>TACAAGGCCAAGAAGCCCGTGCAGCTGCCCGGCGCCTACAACGTC<br>AACATCAAGTTGGACATCACCTCCCACAACGAGGACTACACCATC<br>GTGGAACAGTACGAACGCGCCGAGGGCCGCCACTCCACCGGCGGC<br>ATGGACGAGCTGTACAAGGGATCCGGTATGGACAAGAAGTACAGC<br>ATCGGCCTGGCCATCGGCACCAACTCTGTGGGCTGGGCCGTGATC<br>ACCGACGAGTACAAGGTGCCCAGCAAGAAATTCAAGGTGCTGGGC

	AACACCGACCGGCACAGCATCAAGAAGAACCTGATCGGAGCCCTG<br>CTGTTCGACAGCGGCGAAACAGCCGAGAGAACCCGGCTGAAGAG<br>AACCGCCAGAAGAAGATACACCAGACGGAAGAACCGGATCTGCT<br>ATCTGCAAGAGATCTTCAGCAACGAGATGGCCAAGGTGGACGACA<br>GCTTCTTCCACAGACTGGAAGAGTCCTTCCTGGTGGAAGAGGATA<br>AGAAGCACGAGCGGCACCCCATCTTCGGCAACATCGTGGACGAGG<br>TGGCCTACCACGAGAAGTACCCCACCATCTACCACCTGAGAAAGA<br>AACTGGTGGACAGCACCGACAAGGCCGACCTGCGGCTGATCTATC<br>TGGCCCTGGCCCACATGATCAAGTTCCGGGGCCACTTCCTGATCGA<br>GGGCGACCTGAACCCCGACAACAGCGACGTGGACAAGCTGTTCAT<br>CCAGCTGGTGCAGACCTACAACCAGCTGTTCGAGGAAAACCCCAT<br>CAACGCCAGCGGCGTGGACGCCAAGGCCATCCTGTCTGCCAGACT<br>GAGCAAGAGCAGACGGCTGGAAAATCTGATCGCCCAGCTGCCCGG<br>CGAGAAGAAGAATGGCCTGTTCGGAAACCTGATTGCCCTGAGCCT<br>GGGCCTGACCCCCAACTTCAAGAGCAACTTCGACCTGGCCGAGGA<br>TGCCAAACTGCAGCTGAGCAAGGACACCTACGACGACGACCTGGA<br>CAACCTGCTGGCCCAGATCGGCGACCAGTACGCCGACCTGTTTCTG<br>GCCGCCAAGAACCTGTCCGACGCCATCCTGCTGAGCGACATCCTG<br>AGAGTGAACACCGAGATCACCAAGGCCCCCCTGAGCGCCTCTATG<br>ATCAAGAGATACGACGAGCACCACCAGGACCTGACCCTGCTGAAA<br>GCTCTCGTGCGGCAGCAGCTGCCTGAGAAGTACAAAGAGATTTTC<br>TTCGACCAGAGCAAGAACGGCTACGCCGGCTACATTGACGGCGGA<br>GCCAGCCAGGAAGAGTTCTACAAGTTCATCAAGCCCATCCTGGAA<br>AAGATGGACGGCACCGAGGAACTGCTCGTGAAGCTGAACAGAGA<br>GGACCTGCTGCGGAAGCAGCGGACCTTCGACAACGGCAGCATCCC<br>CCACCAGATCCACCTGGGAGAGCTGCACGCCATTCTGCGGCGGCA<br>GGAAGATTTTTACCCATTCCTGAAGGACAACCGGGAAAAGATCGA<br>GAAGATCCTGACCTTCCGCATCCCCTACTACGTGGGCCCTCTGGCC<br>AGGGGAAACAGCAGATTCGCCTGGATGACCAGAAAGAGCGAGGA<br>AACCATCACCCCCTGGAACTTCGAGGAAGTGGTGGACAAGGGCGC<br>TTCCGCCCAGAGCTTCATCGAGCGGATGACCAACTTCGATAAGAA<br>CCTGCCCAACGAGAAGGTGCTGCCCAAGCACAGCCTGCTGTACGA<br>GTACTTCACCGTGTATAACGAGCTGACCAAAGTGAAATACGTGAC<br>CGAGGGAATGAGAAAGCCCGCCTTCCTGAGCGGCGAGCAGAAAA<br>AGGCCATCGTGGACCTGCTGTTCAAGACCAACCGGAAAGTGACCG<br>TGAAGCAGCTGAAAGAGGACTACTTCAAGAAAATCGAGTGCTTCG<br>ACTCCGTGGAAATCTCCGGCGTGGAAGATCGGTTCAACGCCTCCCT<br>GGGCACATACCACGATCTGCTGAAAATTATCAAGGACAAGGACTT<br>CCTGGACAATGAGGAAAACGAGGACATTCTGGAAGATATCGTGCT<br>GACCCTGACACTGTTTGAGGACAGAGAGATGATCGAGGAACGGCT<br>GAAAACCTATGCCCACCTGTTCGACGACAAAGTGATGAAGCAGCT<br>GAAGCGGCGGAGATACACCGGCTGGGGCAGGCTGAGCCGGAAGC<br>TGATCAACGGCATCCGGGACAAGCAGTCCGGCAAGACAATCCTGG<br>ATTTCCTGAAGTCCGACGGCTTCGCCAACAGAAACTTCATGCAGCT<br>GATCCACGACGACAGCCTGACCTTTAAAGAGGACATCCAGAAAGC<br>CCAGGTGTCCGGCCAGGGCGATAGCCTGCACGAGCACATTGCCAA

	TCTGGCCGGCAGCCCCGCCATTAAGAAGGGCATCCTGCAGACAGT<br>GAAGGTGGTGGACGAGCTCGTGAAAGTGATGGGCCGGCACAAGCC<br>CGAGAACATCGTGATCGAAATGGCCAGAGAGAACCAGACCACCCA<br>GAAGGGACAGAAGAACAGCCGCGAGAGAATGAAGCGGATCGAAG<br>AGGGCATCAAAGAGCTGGGCAGCCAGATCCTGAAAGAACACCCCG<br>TGGAAAACACCCAGCTGCAGAACGAGAAGCTGTACCTGTACTACC<br>TGCAGAATGGGCGGGATATGTACGTGGACCAGGAACTGGACATCA<br>ACCGGCTGTCCGACTACGATGTGGACCATATCGTGCCTCAGAGCTT<br>TCTGAAGGACGACTCCATCGACAACAAGGTGCTGACCAGAAGCGA<br>CAAGAACCGGGGCAAGAGCGACAACGTGCCCTCCGAAGAGGTCGT<br>GAAGAAGATGAAGAACTACTGGCGGCAGCTGCTGAACGCCAAGCT<br>GATTACCCAGAGAAAGTTCGACAATCTGACCAAGGCCGAGAGAGG<br>CGGCCTGAGCGAACTGGATAAGGCCGGCTTCATCAAGAGACAGCT<br>GGTGGAAACCCGGCAGATCACAAAGCACGTGGCACAGATCCTGGA<br>CTCCCGGATGAACACTAAGTACGACGAGAATGACAAGCTGATCCG<br>GGAAGTGAAAGTGATCACCCTGAAGTCCAAGCTGGTGTCCGATTT<br>CCGGAAGGATTTCCAGTTTTACAAAGTGCGCGAGATCAACAACTA<br>CCACCACGCCCACGACGCCTACCTGAACGCCGTCGTGGGAACCGC<br>CCTGATCAAAAAGTACCCTAAGCTGGAAAGCGAGTTCGTGTACGG<br>CGACTACAAGGTGTACGACGTGCGGAAGATGATCGCCAAGAGCGA<br>GCAGGAAATCGGCAAGGCTACCGCCAAGTACTTCTTCTACAGCAA<br>CATCATGAACTTTTTCAAGACCGAGATTACCCTGGCCAACGGCGA<br>GATCCGGAAGCGGCCTCTGATCGAGACAAACGGCGAAACCGGGG<br>AGATCGTGTGGGATAAGGGCCGGGATTTTGCCACCGTGCGGAAAG<br>TGCTGAGCATGCCCCAAGTGAATATCGTGAAAAAGACCGAGGTGC<br>AGACAGGCGGCTTCAGCAAAGAGTCTATCAGACCCAAGAGGAACA<br>GCGATAAGCTGATCGCCAGAAAGAAGGACTGGGACCCTAAGAAGT<br>ACGGCGGCTTCCTGTGGCCCACCGTGGCCTATTCTGTGCTGGTGGT<br>GGCCAAAGTGGAAAAGGGCAAGTCCAAGAAACTGAAGAGTGTGA<br>AAGAGCTGCTGGGGATCACCATCATGGAAAGAAGCAGCTTCGAGA<br>AGAATCCCATCGACTTTCTGGAAGCCAAGGGCTACAAAGAAGTGA<br>AAAAGGACCTGATCATCAAGCTGCCTAAGTACTCCCTGTTCGAGCT<br>GGAAAACGGCCGGAAGAGAATGCTGGCCTCTGCCAAGCAGCTGCA<br>GAAGGGAAACGAACTGGCCCTGCCCTCCAAATATGTGAACTTCCT<br>GTACCTGGCCAGCCACTATGAGAAGCTGAAGGGCTCCCCCGAGGA<br>TAATGAGCAGAAACAGCTGTTTGTGGAACAGCACAAGCACTACCT<br>GGACGAGATCATCGAGCAGATCAGCGAGTTCTCCAAGAGAGTGAT<br>CCTGGCCGACGCTAATCTGGACAAAGTGCTGTCCGCCTACAACAA<br>GCACCGGGATAAGCCCATCAGAGAGCAGGCCGAGAATATCATCCA<br>CCTGTTTACCCTGACCAGACTGGGAGCCCCTAGAGCCTTCAAGTAC<br>TTTGACACCACCATCGACCCCAAGCAGTACAGAAGCACCAAAGAG<br>GTGCTGGACGCCACCCTGATCCACCAGAGCATCACCGGCCTGTAC<br>GAGACACGGATCGACCTGTCTCAGCTGGGAGGCGACGCCTATCCC<br>TATGACGTGCCCGATTATGCCAGCCTGGGCAGCGGCTCCCCCAAG<br>AAAAAACGCAAGGTGGAAGATCCTAAGAAAAAGCGGAAAGGTTC<br>TAGTGAAACCCCGGGAACAAGTGAGTCGGCCACCCCTGAAGGTGG

	ATCAGGGGGTAGCGGATCCTCTTACGATAATTATGTTACGATTCTG<br>GACGAGGAAACCTTAAAGGCTTGGATCGCTAAATTAGAGAAGGCT<br>CCTGTTTTCGCTTTCGACACGGAAACGGATTCTCTGGACAATATTA<br>GTGCGAATCTTGTTGGTCTGAGTTTCGCAATTGAACCGGGTGTTGC<br>TGCTTACATCCCTGTGGCACACGACTACCTGGACGCTCCGGACCAG<br>ATTTCACGTGAACGCGCTCTGGAACTGCTGAAGCCTTTATTAGAGG<br>ACGAGAAAGCTTTGAAAGTTGGTCAGAATTTGAAGTATGCTCGTG<br>GAATCTTAGCTAATTATGGTATCGAGTTGCGCGGTATCGCTTTCGA<br>CACGATGTTGGAATCTTATATCCTGAACTCTGTCGCTGGTCGCCAT<br>GACATGGACTCTCTGGCTGAGCGCTGGCTGAAACATAAGACGATT<br>ACCTTCGAGGAAATCGCAGGAAAGGGTAAGAACCAGCTCACGTTC<br>AATCAAATCGCTCTGGAGGAAGCTGGTCGCTATGCTGCTGAGGAC<br>GCTGACGTTACTCTGCAACTGCACTTGAAGATGTGGCCTGACTTGC<br>AGAAGCATAAGGGTCCACTGAATGTTTTTGAAAACATTGAGATGC<br>CTTTGGTTCCAGTTCTGTCTCGTATCGAGCGCAATGGCGTTAAAAT<br>TGACCCAAAGGTTTTACATAACCACTCAGAGGAACTGACGCTGCG<br>CTTAGCCGAATTGGAGAAAAAGGCTCACGAGATCGCTGGCGAAGA<br>GTTCAATCTGTCATCTACGAAACAACTGCAGACTATCCTGTTCGAG<br>AAGCAAGGTATCAAGCCATTAAAAAAGACCCCTGGCGGTGCTCCG<br>TCTACCTCTGAGGAAGTTTTGGAGGAGTTAGCTTTGGATTACCCTC<br>TGCCGAAGGTTATCTTGGAATACCGCGGTTTGGCTAAATTGAAGTC<br>TACTTATACGGATAAACTTCCTTTGATGATTAATCCAAAGACGGGT<br>CGCGTTCACACGTCGTACCATCAAGCTGTTACCGCTACCGGTCGCC<br>TGTCTTCTACGGATCCGAATTTACAGAATATTCCTGTGCGCAATGA<br>GGAGGGCCGCCGCATTCGTCAAGCTTTTATCGCTCCGGAAGACTAC<br>GTTATCGTTTCTGCTGATTATTCTCAAAATGAATTACGTATCATGG<br>CTCACCTGTCTCGCGATAAGGGTCTGTTGACGGCCTTTGCTGAGGG<br>TAAGGACATTCATCGTGCTACCGCTGCTGAGGTTTACGGCCTGCCG<br>TTGGAAACGGTTACGTCTGAACAGCGTCGCTCTGCTAAGCGTATTA<br>ATTTCGGCTTAATCTACGGTATGTCTGCGTTTGGCTTAGCTCGTCA<br>GCTGAATATCCCGCGCAAGGAAGCTCAAAAATATATGGATCTGTA<br>TTTTGAGCGTTACCACGGTGTTTTGGAATACATGGAGCGTACGCGC<br>GCGCAAGCTAAGGAACAAGGTTATGTGGAAACCTTGGATGGTCGT<br>CGCTTGTACTTGCCTGACATTAAGTCTTCTAACGGCGCCCGCCGCG<br>CTGCTGCCGAGCGCGCTGCTATCAATGCTCCGATGCAAGGTACTGC<br>TGCTGATATTATTAAGCGTGCTATGATCGCTGTGGACGCTTGGCTG<br>CAAGCTGAACAGCCTCGCGTTCGCATGATTATGCAAGTTCATGACG<br>AGTTGGTTTTCGAGGTGCATAAGGACGACGTGGACGCTGTTGCTA<br>AACAAATCCACCAGTTGATGGAGAATTGCACGCGCTTAGACGTTC<br>CGCTGCTGGTTGAAGTTGGTTCTGGTGAAAACTGGGACCAGGCTC<br>ACTAATGGCTGAAAACTGGGACCAGGCTCACTAACTCAGATCCTA<br>CTAGGTTTAATAAACATCTTTATTTTCATTACATCTGTGTGTTGGTT<br>TTTTGTGTGGTACCCAATTCGCCCTATAGTGAGTCGTATTACTCACT<br>GGCCGTCGTTTTACAACGTCGTGACTGGGAAAACCCTGGCGTTACC<br>CAACTTAATCGCCTTGCAGCACATCCCCCTTTCGCCAGCTGGCGTA<br>ATAGCGAAGAGGCCCGCACCGATCGCCCTTCCCAACAGTTGCGCA

	GCCTGAATGGCGAATGGGACGCGCCCTGTAGCGGCGCATTAAGCG CGGCGGGTGTGGTGGTTACGCGCAGCGTGACCGCTACACTTGCCA GCGCCCTAGCGCCCGCTCCTTTCGCTTTCTTCCCTTCCTTTCTCGCC ACGTTCGCCGGCTTTCCCCGTCAAGCTCTAAATCGGGGGCTCCCTT TAGGGTTCCGATTTAGTGCTTTACGGCACCTCGACCCCAAAAAACT TGATTAGGGTGATGGTTCACGTAGTGGGCCATCGCCCTGATAGAC GGTTTTTCGCCCTTTGACGTTGGAGTCCACGTTCTTTAATAGTGGA CTCTTGTTCCAAACTGGAACAACACTCAACCCTATCTCGGTCTATT CTTTTGATTTATAAGGGATTTTGCCGATTTCGGCCTATTGGTTAAA AAATGAGCTGATTTAACAAAAATTTAACGCGAATTTTAACAAAAT ATTAACGCTTACAATTTAGGTGGCACTTTTCGGGGAAATGTG<br><br>**Ampicillin resistance cassette-ColE1 origin of replication-hU6 promoter-gRNA sequence-SpCas9 gRNA scaffold-CMV promoter-NLS-mCherry-GSGM- SpRY-nCas9(D10A)-NLS-PolI5MΔ-stop-SV40 polyA**
p.HF1-nCas9-PolI5MΔ	CGCGGAACCCCTATTTGTTTATTTTTCTAAATACATTCAAATATGTA TCCGCTCATGAGACAATAACCCTGATAAATGCTTCAATAATATTGA AAAAGGAAGAGTATGAGTATTCAACATTTCCGTGTCGCCCTTATTC CCTTTTTTGCGGCATTTTGCCTTCCTGTTTTTGCTCACCCAGAAACG CTGGTGAAAGTAAAAGATGCTGAAGATCAGTTGGGTGCACGAGTG GGTTACATCGAACTGGATCTCAACAGCGGTAAGATCCTTGAGAGT TTTCGCCCCGAAGAACGTTTTCCAATGATGAGCACTTTTAAAGTTC TGCTATGTGGCGCGGTATTATCCCGTATTGACGCCGGGCAAGAGC AACTCGGTCGCCGCATACACTATTCTCAGAATGACTTGGTTGAGTA CTCACCAGTCACAGAAAAGCATCTTACGGATGGCATGACAGTAAG AGAATTATGCAGTGCTGCCATAACCATGAGTGATAACACTGCGGC CAACTTACTTCTGACAACGATCGGAGGACCGAAGGAGCTAACCGC TTTTTTGCACAACATGGGGGATCATGTAACTCGCCTTGATCGTTGG GAACCGGAGCTGAATGAAGCCATACCAAACGACGAGCGTGACACC ACGATGCCTGTAGCAATGGCAACAACGTTGCGCAAACTATTAACT GGCGAACTACTTACTCTAGCTTCCCGGCAACAATTAATAGACTGGA TGGAGGCGGATAAAGTTGCAGGACCACTTCTGCGCTCGGCCCTTCC GGCTGGCTGGTTTATTGCTGATAAATCTGGAGCCGGTGAGCGTGGT TCTCGCGGTATCATTGCAGCACTGGGGCCAGATGGTAAGCCCTCCC GTATCGTAGTTATCTACACGACGGGGAGTCAGGCAACTATGGATG AACGAAATAGACAGATCGCTGAGATAGGTGCCTCACTGATTAAGC ATTGGTAACTGTCAGACCAAGTTTACTCATATATACTTTAGATTGA TTTAAAACTTCATTTTTAATTTAAAAGGATCTAGGTGAAGATCCTT TTTGATAATCTCATGACCAAAATCCCTTAACGTGAGTTTTCGTTCC ACTGAGCGTCAGACCCCGTAGAAAAGATCAAAGGATCTTCTTGAG ATCCTTTTTTTCTGCGCGTAATCTGCTGCTTGCAAACAAAAAAACC ACCGCTACCAGCGGTGGTTTGTTTGCCGGATCAAGAGCTACCAACT CTTTTTCCGAAGGTAACTGGCTTCAGCAGAGCGCAGATACCAAAT ACTGTCCTTCTAGTGTAGCCGTAGTTAGGCCACCACTTCAAGAACT

	CTGTAGCACCGCCTACATACCTCGCTCTGCTAATCCTGTTACCAGT<br>GGCTGCTGCCAGTGGCGATAAGTCGTGTCTTACCGGGTTGGACTCA<br>AGACGATAGTTACCGGATAAGGCGCAGCGGTCGGGCTGAACGGGG<br>GGTTCGTGCACACAGCCCAGCTTGGAGCGAACGACCTACACCGAA<br>CTGAGATACCTACAGCGTGAGCTATGAGAAAGCGCCACGCTTCCC<br>GAAGGGAGAAAGGCGGACAGGTATCCGGTAAGCGGCAGGGTCGG<br>AACAGGAGAGCGCACGAGGGAGCTTCCAGGGGGAAACGCCTGGT<br>ATCTTTATAGTCCTGTCGGGTTTCGCCACCTCTGACTTGAGCGTCG<br>ATTTTTGTGATGCTCGTCAGGGGGGCGGAGCCTATGGAAAAACGC<br>CAGCAACGCGGCCTTTTTACGGTTCCTGGCCTTTTGCTGGCCTTTTG<br>CTCACATGTTCTTTCCTGCGTTATCCCCTGATTCTGTGGATAACCGT<br>ATTACCGCCTTTGAGTGAGCTGATACCGCTCGCCGCAGCCGAACG<br>ACCGAGCGCAGCGAGTCAGTGAGCGAGGAAGCGGAAGAGCGCCC<br>AATACGCAAACCGCCTCTCCCCGCGCGTTGGCCGATTCATTAATGC<br>AGCTGGCACGACAGGTTTCCCGACTGGAAAGCGGGCAGTGAGCGC<br>AACGCAATTAATGTGAGTTAGCTCACTCATTAGGCACCCCAGGCTT<br>TACACTTTATGCTTCCGGCTCGTATGTTGTGTGGAATTGTGAGCGG<br>ATAACAATTTCACACAGGAAACAGCTATGACCATGATTACGCCAA<br>GCGCGCAATTAACCCTCACTAAAGGGAACAAAAGCTGGAGCTCCA<br>CCGCGGTGGCGGCCGCTCTTAAGGGGTGCAGCGGCCTCCGCGCCG<br>GGTTTTGGCGCCTCGATCCAAGGTCGGGCAGGAAGAGGGCCTATT<br>TCCCATGATTCCTTCATATTTGCATATACGATACAAGGCTGTTAGA<br>GAGATAATTAGAATTAATTTGACTGTAAACACAAAGATATTAGTA<br>CAAAATACGTGACGTAGAAAGTAATAATTTCTTGGGTAGTTTGCA<br>GTTTTAAAATTATGTTTTAAAATGGACTATCATATGCTTACCGTAA<br>CTTGAAAGTATTTCGATTTCTTGGCTTTATATATCTTGTGGAAAGG<br>ACGAAACACC-[gRNA SEQUENCE]-<br>GTTTTAGAGCTAGAAATAGCAAGTTAAAATAAGGCTAGTCCGTTA<br>TCAACTTGAAAAAGTGGCACCGAGTCGGTGCTTTTTTCCGCGGCCT<br>CTAGACTCGAGGCGTTGACATTGATTATTGACTAGTTATTAATAGT<br>AATCAATTACGGGGTCATTAGTTCATAGCCCATATATGGAGTTCCG<br>CGTTACATAACTTACGGTAAATGGCCCGCCTGGCTGACCGCCCAAC<br>GACCCCCGCCCATTGACGTCAATAATGACGTATGTTCCCATAGTAA<br>CGCCAATAGGGACTTTCCATTGACGTCAATGGGTGGAGTATTTACG<br>GTAAACTGCCCACTTGGCAGTACATCAAGTGTATCATATGCCAAGT<br>ACGCCCCCTATTGACGTCAATGACGGTAAATGGCCCGCCTGGCATT<br>ATGCCCAGTACATGACCTTATGGGACTTTCCTACTTGGCAGTACAT<br>CTACGTATTAGTCATCGCTATTACCATGGTGATGCGGTTTTGGCAG<br>TACATCAATGGGCGTGGATAGCGGTTTGACTCACGGGGATTTCCA<br>AGTCTCCACCCCATTGACGTCAATGGGAGTTTGTTTTGGCACCAAA<br>ATCAACGGGACTTTCCAAAATGTCGTAACAACTCCGCCCCATTGAC<br>GCAAATGGGCGGTAGGCGTGTACGGTGGGAGGTCTATATAAGCAG<br>AGCTCTCTGGCTAACTACCGGTGCCACCATGGCCCCAAAGAAGAA<br>GCGGAAGGTCGGTATCCACGGAGTCCCAGCAGCCGTGAGCAAGGG<br>CGAGGAGGATAACATGGCCATCATCAAGGAGTTCATGCGCTTCAA<br>GGTGCACATGGAGGGCTCCGTGAACGGCCACGAGTTCGAGATCGA

	GGGCGAGGGCGAGGGCCGCCCCTACGAGGGCACCCAGACCGCCA<br>AGCTGAAGGTGACCAAGGGTGGCCCCCTGCCCTTCGCCTGGGACA<br>TCCTGTCCCCTCAGTTCATGTACGGCTCCAAGGCCTACGTGAAGCA<br>CCCCGCCGACATCCCCGACTACTTGAAGCTGTCCTTCCCCGAGGGC<br>TTCAAGTGGGAGCGCGTGATGAACTTCGAGGACGGCGGCGTGGTG<br>ACCGTGACCCAGGACTCCTCCCTGCAGGACGGCGAGTTCATCTAC<br>AAGGTGAAGCTGCGCGGCACCAACTTCCCCTCCGACGGCCCCGTA<br>ATGCAGAAGAAGACCATGGGCTGGGAGGCCTCCTCCGAGCGGATG<br>TACCCCGAGGACGGCGCCCTGAAGGGCGAGATCAAGCAGAGGCTG<br>AAGCTGAAGGACGGCGGCCACTACGACGCTGAGGTCAAGACCACC<br>TACAAGGCCAAGAAGCCCGTGCAGCTGCCCGGCGCCTACAACGTC<br>AACATCAAGTTGGACATCACCTCCCACAACGAGGACTACACCATC<br>GTGGAACAGTACGAACGCGCCGAGGGCCGCCACTCCACCGGCGGC<br>ATGGACGAGCTGTACAAGGGATCCGGTATGGACAAGAAGTACAGC<br>ATCGGCCTGGCCATCGGCACCAACTCTGTGGGCTGGGCCGTGATC<br>ACCGACGAGTACAAGGTGCCCAGCAAGAAATTCAAGGTGCTGGGC<br>AACACCGACCGGCACAGCATCAAGAAGAACCTGATCGGCGCCCTG<br>CTGTTCGACAGCGGAGAAACAGCCGAGGCCACCCGGCTGAAGAGA<br>ACCGCCAGAAGAAGATACACCAGACGGAAGAACCGGATCTGCTAT<br>CTGCAAGAGATCTTCAGCAACGAGATGGCCAAGGTGGACGACAGC<br>TTCTTCCACAGACTGGAAGAGTCCTTCCTGGTGGAAGAGGATAAG<br>AAGCACGAGCGGCACCCCATCTTCGGCAACATCGTGGACGAGGTG<br>GCCTACCACGAGAAGTACCCCACCATCTACCACCTGAGAAAGAAA<br>CTGGTGGACAGCACCGACAAGGCCGACCTGCGGCTGATCTATCTG<br>GCCCTGGCCCACATGATCAAGTTCCGGGGCCACTTCCTGATCGAGG<br>GCGACCTGAACCCCGACAACAGCGACGTGGACAAGCTGTTCATCC<br>AGCTGGTGCAGACCTACAACCAGCTGTTCGAGGAAAACCCCATCA<br>ACGCCAGCGGCGTGGACGCCAAGGCCATCCTGTCTGCCAGACTGA<br>GCAAGAGCAGACGGCTGGAAAATCTGATCGCCCAGCTGCCCGGCG<br>AGAAGAAGAATGGCCTGTTCGGCAACCTGATTGCCCTGAGCCTGG<br>GCCTGACCCCCAACTTCAAGAGCAACTTCGACCTGGCCGAGGATG<br>CCAAACTGCAGCTGAGCAAGGACACCTACGACGACGACCTGGACA<br>ACCTGCTGGCCCAGATCGGCGACCAGTACGCCGACCTGTTTCTGGC<br>CGCCAAGAACCTGTCCGACGCCATCCTGCTGAGCGACATCCTGAG<br>AGTGAACACCGAGATCACCAAGGCCCCCCTGAGCGCCTCTATGAT<br>CAAGAGATACGACGAGCACCACCAGGACCTGACCCTGCTGAAAGC<br>TCTCGTGCGGCAGCAGCTGCCTGAGAAGTACAAAGAGATTTTCTTC<br>GACCAGAGCAAGAACGGCTACGCCGGCTACATCGATGGCGGAGCC<br>AGCCAGGAAGAGTTCTACAAGTTCATCAAGCCCATCCTGGAAAAG<br>ATGGACGGCACCGAGGAACTGCTCGTGAAGCTGAACAGAGAGGA<br>CCTGCTGCGGAAGCAGCGGACCTTCGACAACGGCAGCATCCCCCA<br>CCAGATCCACCTGGGAGAGCTGCACGCCATTCTGCGGCGGCAGGA<br>AGATTTTTACCCATTCCTGAAGGACAACCGGGAAAAGATCGAGAA<br>GATCCTGACCTTCCGCATCCCCTACTACGTGGGCCCTCTGGCCAGG<br>GGAAACAGCAGATTCGCCTGGATGACCAGAAAGAGCGAGGAAAC<br>CATCACCCCCTGGAACTTCGAGGAAGTGGTGGACAAGGGCGCCAG

	CGCCCAGAGCTTCATCGAGCGGATGACCGCCTTCGATAAGAACCT<br>GCCCAACGAGAAGGTGCTGCCCAAGCACAGCCTGCTGTACGAGTA<br>CTTCACCGTGTACAACGAGCTGACCAAAGTGAAATACGTGACCGA<br>GGGAATGAGAAAGCCCGCCTTCCTGAGCGGCGAGCAGAAAAAAG<br>CCATCGTGGACCTGCTGTTCAAGACCAACCGGAAAGTGACCGTGA<br>AGCAGCTGAAAGAGGACTACTTCAAGAAAATCGAGTGCTTCGACT<br>CCGTGGAAATCTCCGGCGTGGAAGATCGGTTCAACGCCTCCCTGG<br>GCACATACCACGATCTGCTGAAAATTATCAAGGACAAGGACTTCC<br>TGGACAATGAGGAAAACGAGGACATTCTGGAAGATATCGTGCTGA<br>CCCTGACACTGTTTGAGGACAGAGAGATGATCGAGGAACGGCTGA<br>AAACCTATGCCCACCTGTTCGACGACAAAGTGATGAAGCAGCTGA<br>AGCGGCGGAGATACACCGGCTGGGGCGCCCTGAGCCGGAAGCTGA<br>TCAACGGCATCCGGGACAAGCAGTCCGGCAAGACAATCCTGGATT<br>TCCTGAAGTCCGACGGCTTCGCCAACAGAAACTTCATGGCCCTGAT<br>CCACGACGACAGCCTGACCTTTAAAGAGGACATCCAGAAAGCCCA<br>GGTGTCCGGCCAGGGCGATAGCCTGCACGAGCACATTGCCAATCT<br>GGCCGGCAGCCCCGCCATTAAGAAGGGCATCCTGCAGACAGTGAA<br>GGTGGTGGACGAGCTCGTGAAAGTGATGGGCCGGCACAAGCCCGA<br>GAACATCGTGATCGAAATGGCCAGAGAGAACCAGACCACCCAGA<br>AGGGACAGAAGAACAGCCGCGAGAGAATGAAGCGGATCGAAGAG<br>GGCATCAAAGAGCTGGGCAGCCAGATCCTGAAAGAACACCCCGTG<br>GAAAACACCCAGCTGCAGAACGAGAAGCTGTACCTGTACTACCTG<br>CAGAATGGGCGGGATATGTACGTGGACCAGGAACTGGACATCAAC<br>CGGCTGTCCGACTACGATGTGGACCATATCGTGCCTCAGAGCTTTC<br>TGAAGGACGACTCCATCGATAACAAAGTGCTGACTCGGAGCGACA<br>AGAACCGGGGCAAGAGCGACAACGTGCCCTCCGAAGAGGTCGTG<br>AAGAAGATGAAGAACTACTGGCGCCAGCTGCTGAATGCCAAGCTG<br>ATTACCCAGAGGAAGTTCGACAATCTGACCAAGGCCGAGAGAGGC<br>GGCCTGAGCGAACTGGATAAGGCCGGCTTCATCAAGAGACAGCTG<br>GTGGAAACCCGGGCCATCACAAAGCACGTGGCACAGATCCTGGAC<br>TCCCGGATGAACACTAAGTACGACGAGAACGACAAACTGATCCGG<br>GAAGTGAAAGTGATCACCCTGAAGTCCAAGCTGGTGTCCGATTTC<br>CGGAAGGATTTCCAGTTTTACAAAGTGCGCGAGATCAACAACTAC<br>CACCACGCCCACGACGCCTACCTGAACGCCGTCGTGGGAACCGCC<br>CTGATCAAAAAGTACCCTAAGCTGGAAAGCGAGTTCGTGTACGGC<br>GACTACAAGGTGTACGACGTGCGGAAGATGATCGCCAAGAGCGAG<br>CAGGAAATCGGCAAGGCTACCGCCAAGTACTTCTTCTACAGCAAC<br>ATCATGAACTTTTTCAAGACCGAGATTACCCTGGCCAACGGCGAG<br>ATCCGGAAGCGGCCTCTGATCGAGACAAACGGCGAAACAGGCGA<br>GATCGTGTGGGATAAGGGCCGGGACTTTGCCACCGTGCGGAAAGT<br>GCTGTCTATGCCCCAAGTGAATATCGTGAAAAAGACCGAGGTGCA<br>GACAGGCGGCTTCAGCAAAGAGTCTATCCTGCCCAAGAGGAACAG<br>CGACAAGCTGATCGCCAGAAAGAAGGACTGGGACCCTAAGAAGT<br>ACGGCGGCTTCGACAGCCCCACCGTGGCCTATTCTGTGCTGGTGGT<br>GGCCAAAGTGGAAAAGGGCAAGTCCAAGAAACTGAAGAGTGTGA<br>AAGAGCTGCTGGGGATCACCATCATGGAAAGAAGCAGCTTCGAGA

	AGAATCCCATCGACTTTCTGGAAGCCAAGGGCTACAAAGAAGTGA<br>AAAAGGACCTGATCATCAAGCTGCCTAAGTACTCCCTGTTCGAGCT<br>GGAAAACGGCCGGAAGAGAATGCTGGCCTCTGCCGGCGAACTGCA<br>GAAGGGAAACGAACTGGCCCTGCCCTCCAAATATGTGAACTTCCT<br>GTACCTGGCCAGCCACTATGAGAAGCTGAAGGGCTCCCCCGAGGA<br>TAATGAGCAGAAACAGCTGTTTGTGGAACAGCACAAACACTACCT<br>GGACGAGATCATCGAGCAGATCAGCGAGTTCTCCAAGAGAGTGAT<br>CCTGGCCGACGCTAATCTGGACAAGGTGCTGAGCGCCTACAACAA<br>GCACAGAGACAAGCCTATCAGAGAGCAGGCCGAGAATATCATCCA<br>CCTGTTTACCCTGACCAATCTGGGAGCCCCTGCCGCCTTCAAGTAC<br>TTTGACACCACCATCGACCGGAAGAGGTACACCAGCACCAAAGAG<br>GTGCTGGACGCCACCCTGATCCACCAGAGCATCACCGGCCTGTAC<br>GAGACACGGATCGACCTGTCTCAGCTGGGAGGCGACGCCTATCCC<br>TATGACGTGCCCGATTATGCCAGCCTGGGCAGCGGCTCCCCCAAG<br>AAAAAACGCAAGGTGGAAGATCCTAAGAAAAAGCGGAAAGGTTC<br>TAGTGAAACCCCGGGAACAAGTGAGTCGGCCACCCCTGAAGGTGG<br>ATCAGGGGGTAGCGGATCCTCTTACGATAATTATGTTACGATTCTG<br>GACGAGGAAACCTTAAAGGCTTGGATCGCTAAATTAGAGAAGGCT<br>CCTGTTTTCGCTTTCGACACGGAAACGGATTCTCTGGACAATATTA<br>GTGCGAATCTTGTTGGTCTGAGTTTCGCAATTGAACCGGGTGTTGC<br>TGCTTACATCCCTGTGGCACACGACTACCTGGACGCTCCGGACCAG<br>ATTTCACGTGAACGCGCTCTGGAACTGCTGAAGCCTTTATTAGAGG<br>ACGAGAAAGCTTTGAAAGTTGGTCAGAATTTGAAGTATGCTCGTG<br>GAATCTTAGCTAATTATGGTATCGAGTTGCGCGGTATCGCTTTCGA<br>CACGATGTTGGAATCTTATATCCTGAACTCTGTCGCTGGTCGCCAT<br>GACATGGACTCTCTGGCTGAGCGCTGGCTGAAACATAAGACGATT<br>ACCTTCGAGGAAATCGCAGGAAAGGGTAAGAACCAGCTCACGTTC<br>AATCAAATCGCTCTGGAGGAAGCTGGTCGCTATGCTGCTGAGGAC<br>GCTGACGTTACTCTGCAACTGCACTTGAAGATGTGGCCTGACTTGC<br>AGAAGCATAAGGGTCCACTGAATGTTTTTGAAAACATTGAGATGC<br>CTTTGGTTCCAGTTCTGTCTCGTATCGAGCGCAATGGCGTTAAAAT<br>TGACCCAAAGGTTTTACATAACCACTCAGAGGAACTGACGCTGCG<br>CTTAGCCGAATTGGAGAAAAAGGCTCACGAGATCGCTGGCGAAGA<br>GTTCAATCTGTCATCTACGAAACAACTGCAGACTATCCTGTTCGAG<br>AAGCAAGGTATCAAGCCATTAAAAAAGACCCCTGGCGGTGCTCCG<br>TCTACCTCTGAGGAAGTTTTGGAGGAGTTAGCTTTGGATTACCCTC<br>TGCCGAAGGTTATCTTGGAATACCGCGGTTTGGCTAAATTGAAGTC<br>TACTTATACGGATAAACTTCCTTTGATGATTAATCCAAAGACGGGT<br>CGCGTTCACACGTCGTACCATCAAGCTGTTACCGCTACCGGTCGCC<br>TGTCTTCTACGGATCCGAATTTACAGAATATTCCTGTGCGCAATGA<br>GGAGGGCCGCCGCATTCGTCAAGCTTTTATCGCTCCGGAAGACTAC<br>GTTATCGTTTCTGCTGATTATTCTCAAAATGAATTACGTATCATGG<br>CTCACCTGTCTCGCGATAAGGGTCTGTTGACGGCCTTTGCTGAGGG<br>TAAGGACATTCATCGTGCTACCGCTGCTGAGGTTTACGGCCTGCCG<br>TTGGAAACGGTTACGTCTGAACAGCGTCGCTCTGCTAAGCGTATTA<br>ATTTCGGCTTAATCTACGGTATGTCTGCGTTTGGCTTAGCTCGTCA

	GCTGAATATCCCGCGCAAGGAAGCTCAAAAATATATGGATCTGTA<br>TTTTGAGCGTTACCACGGTGTTTTGGAATACATGGAGCGTACGCGC<br>GCGCAAGCTAAGGAACAAGGTTATGTGGAAACCTTGGATGGTCGT<br>CGCTTGTACTTGCCTGACATTAAGTCTTCTAACGGCGCCCGCCGCG<br>CTGCTGCCGAGCGCGCTGCTATCAATGCTCCGATGCAAGGTACTGC<br>TGCTGATATTATTAAGCGTGCTATGATCGCTGTGGACGCTTGGCTG<br>CAAGCTGAACAGCCTCGCGTTCGCATGATTATGCAAGTTCATGACG<br>AGTTGGTTTTCGAGGTGCATAAGGACGACGTGGACGCTGTTGCTA<br>AACAAATCCACCAGTTGATGGAGAATTGCACGCGCTTAGACGTTC<br>CGCTGCTGGTTGAAGTTGGTTCTGGTGAAAACTGGGACCAGGCTC<br>ACTAATGGCTGAAAACTGGGACCAGGCTCACTAACTCAGATCCTA<br>CTAGGTTTAATAAACATCTTTATTTTCATTACATCTGTGTGTTGGTT<br>TTTTGTGTGGTACCCAATTCGCCCTATAGTGAGTCGTATTACTCACT<br>GGCCGTCGTTTTACAACGTCGTGACTGGGAAAACCCTGGCGTTACC<br>CAACTTAATCGCCTTGCAGCACATCCCCCTTTCGCCAGCTGGCGTA<br>ATAGCGAAGAGGCCCGCACCGATCGCCCTTCCCAACAGTTGCGCA<br>GCCTGAATGGCGAATGGGACGCGCCCTGTAGCGGCGCATTAAGCG<br>CGGCGGGTGTGGTGGTTACGCGCAGCGTGACCGCTACACTTGCCA<br>GCGCCCTAGCGCCCGCTCCTTTCGCTTTCTTCCCTTCCTTTCTCGCC<br>ACGTTCGCCGGCTTTCCCCGTCAAGCTCTAAATCGGGGGCTCCCTT<br>TAGGGTTCCGATTTAGTGCTTTACGGCACCTCGACCCCAAAAAACT<br>TGATTAGGGTGATGGTTCACGTAGTGGGCCATCGCCCTGATAGAC<br>GGTTTTTCGCCCTTTGACGTTGGAGTCCACGTTCTTTAATAGTGGA<br>CTCTTGTTCCAAACTGGAACAACACTCAACCCTATCTCGGTCTATT<br>CTTTTGATTTATAAGGGATTTTGCCGATTTCGGCCTATTGGTTAAA<br>AAATGAGCTGATTTAACAAAAATTTAACGCGAATTTTAACAAAAT<br>ATTAACGCTTACAATTTAGGTGGCACTTTTCGGGGAAATGTG<br><br>**Ampicillin resistance cassette-ColE1 origin of replication-hU6 promoter-gRNA sequence-SpCas9 gRNA scaffold-CMV promoter-NLS-mCherry-GSGM- HFI-nCas9(D10A)-NLS-PolI5MΔ-stop-SV40 polyA**
p.Hypa-nCas9-PolI5MΔ	CGCGGAACCCCTATTTGTTTATTTTTCTAAATACATTCAAATATGTA<br>TCCGCTCATGAGACAATAACCCTGATAAATGCTTCAATAATATTGA<br>AAAAGGAAGAGTATGAGTATTCAACATTTCCGTGTCGCCCTTATTC<br>CCTTTTTTGCGGCATTTTGCCTTCCTGTTTTTGCTCACCCAGAAACG<br>CTGGTGAAAGTAAAAGATGCTGAAGATCAGTTGGGTGCACGAGTG<br>GGTTACATCGAACTGGATCTCAACAGCGGTAAGATCCTTGAGAGT<br>TTTCGCCCCGAAGAACGTTTTCCAATGATGAGCACTTTTAAAGTTC<br>TGCTATGTGGCGCGGTATTATCCCGTATTGACGCCGGGCAAGAGC<br>AACTCGGTCGCCGCATACACTATTCTCAGAATGACTTGGTTGAGTA<br>CTCACCAGTCACAGAAAAGCATCTTACGGATGGCATGACAGTAAG<br>AGAATTATGCAGTGCTGCCATAACCATGAGTGATAACACTGCGGC<br>CAACTTACTTCTGACAACGATCGGAGGACCGAAGGAGCTAACCGC<br>TTTTTTGCACAACATGGGGGATCATGTAACTCGCCTTGATCGTTGG

	GAACCGGAGCTGAATGAAGCCATACCAAACGACGAGCGTGACACC<br>ACGATGCCTGTAGCAATGGCAACAACGTTGCGCAAACTATTAACT<br>GGCGAACTACTTACTCTAGCTTCCCGGCAACAATTAATAGACTGGA<br>TGGAGGCGGATAAAGTTGCAGGACCACTTCTGCGCTCGGCCCTTCC<br>GGCTGGCTGGTTTATTGCTGATAAATCTGGAGCCGGTGAGCGTGGT<br>TCTCGCGGTATCATTGCAGCACTGGGGCCAGATGGTAAGCCCTCCC<br>GTATCGTAGTTATCTACACGACGGGGAGTCAGGCAACTATGGATG<br>AACGAAATAGACAGATCGCTGAGATAGGTGCCTCACTGATTAAGC<br>ATTGGTAACTGTCAGACCAAGTTTACTCATATATACTTTAGATTGA<br>TTTAAAACTTCATTTTTAATTTAAAAGGATCTAGGTGAAGATCCTT<br>TTTGATAATCTCATGACCAAAATCCCTTAACGTGAGTTTTCGTTCC<br>ACTGAGCGTCAGACCCCGTAGAAAAGATCAAAGGATCTTCTTGAG<br>ATCCTTTTTTTCTGCGCGTAATCTGCTGCTTGCAAACAAAAAAACC<br>ACCGCTACCAGCGGTGGTTTGTTTGCCGGATCAAGAGCTACCAACT<br>CTTTTTCCGAAGGTAACTGGCTTCAGCAGAGCGCAGATACCAAAT<br>ACTGTCCTTCTAGTGTAGCCGTAGTTAGGCCACCACTTCAAGAACT<br>CTGTAGCACCGCCTACATACCTCGCTCTGCTAATCCTGTTACCAGT<br>GGCTGCTGCCAGTGGCGATAAGTCGTGTCTTACCGGGTTGGACTCA<br>AGACGATAGTTACCGGATAAGGCGCAGCGGTCGGGCTGAACGGGG<br>GGTTCGTGCACACAGCCCAGCTTGGAGCGAACGACCTACACCGAA<br>CTGAGATACCTACAGCGTGAGCTATGAGAAAGCGCCACGCTTCCC<br>GAAGGGAGAAAGGCGGACAGGTATCCGGTAAGCGGCAGGGTCGG<br>AACAGGAGAGCGCACGAGGGAGCTTCCAGGGGGAAACGCCTGGT<br>ATCTTTATAGTCCTGTCGGGTTTCGCCACCTCTGACTTGAGCGTCG<br>ATTTTTGTGATGCTCGTCAGGGGGGCGGAGCCTATGGAAAAACGC<br>CAGCAACGCGGCCTTTTTACGGTTCCTGGCCTTTTGCTGGCCTTTTG<br>CTCACATGTTCTTTCCTGCGTTATCCCCTGATTCTGTGGATAACCGT<br>ATTACCGCCTTTGAGTGAGCTGATACCGCTCGCCGCAGCCGAACG<br>ACCGAGCGCAGCGAGTCAGTGAGCGAGGAAGCGGAAGAGCGCCC<br>AATACGCAAACCGCCTCTCCCCGCGCGTTGGCCGATTCATTAATGC<br>AGCTGGCACGACAGGTTTCCCGACTGGAAAGCGGGCAGTGAGCGC<br>AACGCAATTAATGTGAGTTAGCTCACTCATTAGGCACCCCAGGCTT<br>TACACTTTATGCTTCCGGCTCGTATGTTGTGTGGAATTGTGAGCGG<br>ATAACAATTTCACACAGGAAACAGCTATGACCATGATTACGCCAA<br>GCGCGCAATTAACCCTCACTAAAGGGAACAAAAGCTGGAGCTCCA<br>CCGCGGTGGCGGCCGCTCTTAAGGGGTGCAGCGGCCTCCGCGCCG<br>GGTTTTGGCGCCTCGATCCAAGGTCGGGCAGGAAGAGGGCCTATT<br>TCCCATGATTCCTTCATATTTGCATATACGATACAAGGCTGTTAGA<br>GAGATAATTAGAATTAATTTGACTGTAAACACAAAGATATTAGTA<br>CAAAATACGTGACGTAGAAAGTAATAATTTCTTGGGTAGTTTGCA<br>GTTTTAAAATTATGTTTTAAAATGGACTATCATATGCTTACCGTAA<br>CTTGAAAGTATTTCGATTTCTTGGCTTTATATATCTTGTGGAAAGG<br>ACGAAACACC-[gRNA SEQUENCE]-<br>GTTTTAGAGCTAGAAATAGCAAGTTAAAATAAGGCTAGTCCGTTA<br>TCAACTTGAAAAAGTGGCACCGAGTCGGTGCTTTTTTCCGCGGCCT<br>CTAGACTCGAGGCGTTGACATTGATTATTGACTAGTTATTAATAGT

	AATCAATTACGGGGTCATTAGTTCATAGCCCATATATGGAGTTCCG<br>CGTTACATAACTTACGGTAAATGGCCCGCCTGGCTGACCGCCCAAC<br>GACCCCCGCCCATTGACGTCAATAATGACGTATGTTCCCATAGTAA<br>CGCCAATAGGGACTTTCCATTGACGTCAATGGGTGGAGTATTTACG<br>GTAAACTGCCCACTTGGCAGTACATCAAGTGTATCATATGCCAAGT<br>ACGCCCCCTATTGACGTCAATGACGGTAAATGGCCCGCCTGGCATT<br>ATGCCCAGTACATGACCTTATGGGACTTTCCTACTTGGCAGTACAT<br>CTACGTATTAGTCATCGCTATTACCATGGTGATGCGGTTTTGGCAG<br>TACATCAATGGGCGTGGATAGCGGTTTGACTCACGGGGATTTCCA<br>AGTCTCCACCCCATTGACGTCAATGGGAGTTTGTTTTGGCACCAAA<br>ATCAACGGGACTTTCCAAAATGTCGTAACAACTCCGCCCCATTGAC<br>GCAAATGGGCGGTAGGCGTGTACGGTGGGAGGTCTATATAAGCAG<br>AGCTCTCTGGCTAACTACCGGTGCCACCATGGCCCCAAAGAAGAA<br>GCGGAAGGTCGGTATCCACGGAGTCCCAGCAGCCGTGAGCAAGGG<br>CGAGGAGGATAACATGGCCATCATCAAGGAGTTCATGCGCTTCAA<br>GGTGCACATGGAGGGCTCCGTGAACGGCCACGAGTTCGAGATCGA<br>GGGCGAGGGCGAGGGCCGCCCCTACGAGGGCACCCAGACCGCCA<br>AGCTGAAGGTGACCAAGGGTGGCCCCCTGCCCTTCGCCTGGGACA<br>TCCTGTCCCCTCAGTTCATGTACGGCTCCAAGGCCTACGTGAAGCA<br>CCCCGCCGACATCCCCGACTACTTGAAGCTGTCCTTCCCCGAGGGC<br>TTCAAGTGGGAGCGCGTGATGAACTTCGAGGACGGCGGCGTGGTG<br>ACCGTGACCCAGGACTCCTCCCTGCAGGACGGCGAGTTCATCTAC<br>AAGGTGAAGCTGCGCGGCACCAACTTCCCCTCCGACGGCCCCGTA<br>ATGCAGAAGAAGACCATGGGCTGGGAGGCCTCCTCCGAGCGGATG<br>TACCCCGAGGACGGCGCCCTGAAGGGCGAGATCAAGCAGAGGCTG<br>AAGCTGAAGGACGGCGGCCACTACGACGCTGAGGTCAAGACCACC<br>TACAAGGCCAAGAAGCCCGTGCAGCTGCCCGGCGCCTACAACGTC<br>AACATCAAGTTGGACATCACCTCCCACAACGAGGACTACACCATC<br>GTGGAACAGTACGAACGCGCCGAGGGCCGCCACTCCACCGGCGGC<br>ATGGACGAGCTGTACAAGGGATCCGGTATGGACAAGAAGTACAGC<br>ATCGGCCTGGCCATCGGCACCAACTCTGTGGGCTGGGCCGTGATC<br>ACCGACGAGTACAAGGTGCCCAGCAAGAAATTCAAGGTGCTGGGC<br>AACACCGACCGGCACAGCATCAAGAAGAACCTGATCGGCGCCCTG<br>CTGTTCGACAGCGGAGAAACAGCCGAGGCCACCCGGCTGAAGAGA<br>ACCGCCAGAAGAAGATACACCAGACGGAAGAACCGGATCTGCTAT<br>CTGCAAGAGATCTTCAGCAACGAGATGGCCAAGGTGGACGACAGC<br>TTCTTCCACAGACTGGAAGAGTCCTTCCTGGTGGAAGAGGATAAG<br>AAGCACGAGCGGCACCCCATCTTCGGCAACATCGTGGACGAGGTG<br>GCCTACCACGAGAAGTACCCCACCATCTACCACCTGAGAAAGAAA<br>CTGGTGGACAGCACCGACAAGGCCGACCTGCGGCTGATCTATCTG<br>GCCCTGGCCCACATGATCAAGTTCCGGGGCCACTTCCTGATCGAGG<br>GCGACCTGAACCCCGACAACAGCGACGTGGACAAGCTGTTCATCC<br>AGCTGGTGCAGACCTACAACCAGCTGTTCGAGGAAAACCCCATCA<br>ACGCCAGCGGCGTGGACGCCAAGGCCATCCTGTCTGCCAGACTGA<br>GCAAGAGCAGACGGCTGGAAAATCTGATCGCCCAGCTGCCCGGCG<br>AGAAGAAGAATGGCCTGTTCGGCAACCTGATTGCCCTGAGCCTGG

	GCCTGACCCCCAACTTCAAGAGCAACTTCGACCTGGCCGAGGATG<br>CCAAACTGCAGCTGAGCAAGGACACCTACGACGACGACCTGGACA<br>ACCTGCTGGCCCAGATCGGCGACCAGTACGCCGACCTGTTTCTGGC<br>CGCCAAGAACCTGTCCGACGCCATCCTGCTGAGCGACATCCTGAG<br>AGTGAACACCGAGATCACCAAGGCCCCCCTGAGCGCCTCTATGAT<br>CAAGAGATACGACGAGCACCACCAGGACCTGACCCTGCTGAAAGC<br>TCTCGTGCGGCAGCAGCTGCCTGAGAAGTACAAAGAGATTTTCTTC<br>GACCAGAGCAAGAACGGCTACGCCGGCTACATCGATGGCGGAGCC<br>AGCCAGGAAGAGTTCTACAAGTTCATCAAGCCCATCCTGGAAAAG<br>ATGGACGGCACCGAGGAACTGCTCGTGAAGCTGAACAGAGAGGA<br>CCTGCTGCGGAAGCAGCGGACCTTCGACAACGGCAGCATCCCCCA<br>CCAGATCCACCTGGGAGAGCTGCACGCCATTCTGCGGCGGCAGGA<br>AGATTTTTACCCATTCCTGAAGGACAACCGGGAAAAGATCGAGAA<br>GATCCTGACCTTCCGCATCCCCTACTACGTGGGCCCTCTGGCCAGG<br>GGAAACAGCAGATTCGCCTGGATGACCAGAAAGAGCGAGGAAAC<br>CATCACCCCCTGGAACTTCGAGGAAGTGGTGGACAAGGGCGCCAG<br>CGCCCAGAGCTTCATCGAGCGGATGACCAACTTCGATAAGAACCT<br>GCCCAACGAGAAGGTGCTGCCCAAGCACAGCCTGCTGTACGAGTA<br>CTTCACCGTGTACAACGAGCTGACCAAAGTGAAATACGTGACCGA<br>GGGAATGAGAAAGCCCGCCTTCCTGAGCGGCGAGCAGAAAAAAG<br>CCATCGTGGACCTGCTGTTCAAGACCAACCGGAAAGTGACCGTGA<br>AGCAGCTGAAAGAGGACTACTTCAAGAAAATCGAGTGCTTCGACT<br>CCGTGGAAATCTCCGGCGTGGAAGATCGGTTCAACGCCTCCCTGG<br>GCACATACCACGATCTGCTGAAAATTATCAAGGACAAGGACTTCC<br>TGGACAATGAGGAAAACGAGGACATTCTGGAAGATATCGTGCTGA<br>CCCTGACACTGTTTGAGGACAGAGAGATGATCGAGGAACGGCTGA<br>AAACCTATGCCCACCTGTTCGACGACAAAGTGATGAAGCAGCTGA<br>AGCGGCGGAGATACACCGGCTGGGGCAGGCTGAGCCGGAAGCTG<br>ATCAACGGCATCCGGGACAAGCAGTCCGGCAAGACAATCCTGGAT<br>TTCCTGAAGTCCGACGGCTTCGCCAACAGAGCCTTCGCCGCCCTGA<br>TCGCCGACGACAGCCTGACCTTTAAAGAGGACATCCAGAAAGCCC<br>AGGTGTCCGGCCAGGGCGATAGCCTGCACGAGCACATTGCCAATC<br>TGGCCGGCAGCCCCGCCATTAAGAAGGGCATCCTGCAGACAGTGA<br>AGGTGGTGGACGAGCTCGTGAAAGTGATGGGCCGGCACAAGCCCG<br>AGAACATCGTGATCGAAATGGCCAGAGAGAACCAGACCACCCAG<br>AAGGGACAGAAGAACAGCCGCGAGAGAATGAAGCGGATCGAAGA<br>GGGCATCAAAGAGCTGGGCAGCCAGATCCTGAAAGAACACCCCGT<br>GGAAAACACCCAGCTGCAGAACGAGAAGCTGTACCTGTACTACCT<br>GCAGAATGGGCGGGATATGTACGTGGACCAGGAACTGGACATCAA<br>CCGGCTGTCCGACTACGATGTGGACCATATCGTGCCTCAGAGCTTT<br>CTGAAGGACGACTCCATCGATAACAAAGTGCTGACTCGGAGCGAC<br>AAGAACCGGGGCAAGAGCGACAACGTGCCCTCCGAAGAGGTCGT<br>GAAGAAGATGAAGAACTACTGGCGCCAGCTGCTGAATGCCAAGCT<br>GATTACCCAGAGGAAGTTCGACAATCTGACCAAGGCCGAGAGAGG<br>CGGCCTGAGCGAACTGGATAAGGCCGGCTTCATCAAGAGACAGCT<br>GGTGGAAACCCGGCAGATCACAAAGCACGTGGCACAGATCCTGGA

	CTCCCGGATGAACACTAAGTACGACGAGAACGACAAACTGATCCG<br>GGAAGTGAAAGTGATCACCCTGAAGTCCAAGCTGGTGTCCGATTT<br>CCGGAAGGATTTCCAGTTTTACAAAGTGCGCGAGATCAACAACTA<br>CCACCACGCCCACGACGCCTACCTGAACGCCGTCGTGGGAACCGC<br>CCTGATCAAAAAGTACCCTAAGCTGGAAAGCGAGTTCGTGTACGG<br>CGACTACAAGGTGTACGACGTGCGGAAGATGATCGCCAAGAGCGA<br>GCAGGAAATCGGCAAGGCTACCGCCAAGTACTTCTTCTACAGCAA<br>CATCATGAACTTTTTCAAGACCGAGATTACCCTGGCCAACGGCGA<br>GATCCGGAAGCGGCCTCTGATCGAGACAAACGGCGAAACAGGCG<br>AGATCGTGTGGGATAAGGGCCGGGACTTTGCCACCGTGCGGAAAG<br>TGCTGTCTATGCCCCAAGTGAATATCGTGAAAAAGACCGAGGTGC<br>AGACAGGCGGCTTCAGCAAAGAGTCTATCCTGCCCAAGAGGAACA<br>GCGACAAGCTGATCGCCAGAAAGAAGGACTGGGACCCTAAGAAG<br>TACGGCGGCTTCGACAGCCCCACCGTGGCCTATTCTGTGCTGGTGG<br>TGGCCAAAGTGGAAAAGGGCAAGTCCAAGAAACTGAAGAGTGTG<br>AAAGAGCTGCTGGGGATCACCATCATGGAAAGAAGCAGCTTCGAG<br>AAGAATCCCATCGACTTTCTGGAAGCCAAGGGCTACAAAGAAGTG<br>AAAAAGGACCTGATCATCAAGCTGCCTAAGTACTCCCTGTTCGAG<br>CTGGAAAACGGCCGGAAGAGAATGCTGGCCTCTGCCGGCGAACTG<br>CAGAAGGGAAACGAACTGGCCCTGCCCTCCAAATATGTGAACTTC<br>CTGTACCTGGCCAGCCACTATGAGAAGCTGAAGGGCTCCCCCGAG<br>GATAATGAGCAGAAACAGCTGTTTGTGGAACAGCACAAACACTAC<br>CTGGACGAGATCATCGAGCAGATCAGCGAGTTCTCCAAGAGAGTG<br>ATCCTGGCCGACGCTAATCTGGACAAGGTGCTGAGCGCCTACAAC<br>AAGCACAGAGACAAGCCTATCAGAGAGCAGGCCGAGAATATCATC<br>CACCTGTTTACCCTGACCAATCTGGGAGCCCCTGCCGCCTTCAAGT<br>ACTTTGACACCACCATCGACCGGAAGAGGTACACCAGCACCAAAG<br>AGGTGCTGGACGCCACCCTGATCCACCAGAGCATCACCGGCCTGT<br>ACGAGACACGGATCGACCTGTCTCAGCTGGGAGGCGACGCCTATC<br>CCTATGACGTGCCCGATTATGCCAGCCTGGGCAGCGGCTCCCCCAA<br>GAAAAAACGCAAGGTGGAAGATCCTAAGAAAAAGCGGAAAGGTT<br>CTAGTGAAACCCCGGGAACAAGTGAGTCGGCCACCCCTGAAGGTG<br>GATCAGGGGGTAGCGGATCCTCTTACGATAATTATGTTACGATTCT<br>GGACGAGGAAACCTTAAAGGCTTGGATCGCTAAATTAGAGAAGGC<br>TCCTGTTTTCGCTTTCGACACGGAAACGGATTCTCTGGACAATATT<br>AGTGCGAATCTTGTTGGTCTGAGTTTCGCAATTGAACCGGGTGTTG<br>CTGCTTACATCCCTGTGGCACACGACTACCTGGACGCTCCGGACCA<br>GATTTCACGTGAACGCGCTCTGGAACTGCTGAAGCCTTTATTAGAG<br>GACGAGAAAGCTTTGAAAGTTGGTCAGAATTTGAAGTATGCTCGT<br>GGAATCTTAGCTAATTATGGTATCGAGTTGCGCGGTATCGCTTTCG<br>ACACGATGTTGGAATCTTATATCCTGAACTCTGTCGCTGGTCGCCA<br>TGACATGGACTCTCTGGCTGAGCGCTGGCTGAAACATAAGACGAT<br>TACCTTCGAGGAAATCGCAGGAAAGGGTAAGAACCAGCTCACGTT<br>CAATCAAATCGCTCTGGAGGAAGCTGGTCGCTATGCTGCTGAGGA<br>CGCTGACGTTACTCTGCAACTGCACTTGAAGATGTGGCCTGACTTG<br>CAGAAGCATAAGGGTCCACTGAATGTTTTTGAAAACATTGAGATG

	CCTTTGGTTCCAGTTCTGTCTCGTATCGAGCGCAATGGCGTTAAAA<br>TTGACCCAAAGGTTTTACATAACCACTCAGAGGAACTGACGCTGC<br>GCTTAGCCGAATTGGAGAAAAAGGCTCACGAGATCGCTGGCGAAG<br>AGTTCAATCTGTCATCTACGAAACAACTGCAGACTATCCTGTTCGA<br>GAAGCAAGGTATCAAGCCATTAAAAAAGACCCCTGGCGGTGCTCC<br>GTCTACCTCTGAGGAAGTTTTGGAGGAGTTAGCTTTGGATTACCCT<br>CTGCCGAAGGTTATCTTGGAATACCGCGGTTTGGCTAAATTGAAGT<br>CTACTTATACGGATAAACTTCCTTTGATGATTAATCCAAAGACGGG<br>TCGCGTTCACACGTCGTACCATCAAGCTGTTACCGCTACCGGTCGC<br>CTGTCTTCTACGGATCCGAATTTACAGAATATTCCTGTGCGCAATG<br>AGGAGGGCCGCCGCATTCGTCAAGCTTTTATCGCTCCGGAAGACT<br>ACGTTATCGTTTCTGCTGATTATTCTCAAAATGAATTACGTATCAT<br>GGCTCACCTGTCTCGCGATAAGGGTCTGTTGACGGCCTTTGCTGAG<br>GGTAAGGACATTCATCGTGCTACCGCTGCTGAGGTTTACGGCCTGC<br>CGTTGGAAACGGTTACGTCTGAACAGCGTCGCTCTGCTAAGCGTAT<br>TAATTTCGGCTTAATCTACGGTATGTCTGCGTTTGGCTTAGCTCGTC<br>AGCTGAATATCCCGCGCAAGGAAGCTCAAAAATATATGGATCTGT<br>ATTTTGAGCGTTACCACGGTGTTTTGGAATACATGGAGCGTACGCG<br>CGCGCAAGCTAAGGAACAAGGTTATGTGGAAACCTTGGATGGTCG<br>TCGCTTGTACTTGCCTGACATTAAGTCTTCTAACGGCGCCCGCCGC<br>GCTGCTGCCGAGCGCGCTGCTATCAATGCTCCGATGCAAGGTACTG<br>CTGCTGATATTATTAAGCGTGCTATGATCGCTGTGGACGCTTGGCT<br>GCAAGCTGAACAGCCTCGCGTTCGCATGATTATGCAAGTTCATGAC<br>GAGTTGGTTTTCGAGGTGCATAAGGACGACGTGGACGCTGTTGCT<br>AAACAAATCCACCAGTTGATGGAGAATTGCACGCGCTTAGACGTT<br>CCGCTGCTGGTTGAAGTTGGTTCTGGTGAAAACTGGGACCAGGCTC<br>ACTAATGGCTGAAAACTGGGACCAGGCTCACTAACTCAGATCCTA<br>CTAGGTTTAATAAACATCTTTATTTTCATTACATCTGTGTGTTGGTT<br>TTTTGTGTGGTACCCAATTCGCCCTATAGTGAGTCGTATTACTCACT<br>GGCCGTCGTTTTACAACGTCGTGACTGGGAAAACCCTGGCGTTACC<br>CAACTTAATCGCCTTGCAGCACATCCCCCTTTCGCCAGCTGGCGTA<br>ATAGCGAAGAGGCCCGCACCGATCGCCCTTCCCAACAGTTGCGCA<br>GCCTGAATGGCGAATGGGACGCGCCCTGTAGCGGCGCATTAAGCG<br>CGGCGGGTGTGGTGGTTACGCGCAGCGTGACCGCTACACTTGCCA<br>GCGCCCTAGCGCCCGCTCCTTTCGCTTTCTTCCCTTCCTTTCTCGCC<br>ACGTTCGCCGGCTTTCCCCGTCAAGCTCTAAATCGGGGGCTCCCTT<br>TAGGGTTCCGATTTAGTGCTTTACGGCACCTCGACCCCAAAAAACT<br>TGATTAGGGTGATGGTTCACGTAGTGGGCCATCGCCCTGATAGAC<br>GGTTTTTCGCCCTTTGACGTTGGAGTCCACGTTCTTTAATAGTGGA<br>CTCTTGTTCCAAACTGGAACAACACTCAACCCTATCTCGGTCTATT<br>CTTTTGATTTATAAGGGATTTTGCCGATTTCGGCCTATTGGTTAAA<br>AAATGAGCTGATTTAACAAAAATTTAACGCGAATTTTAACAAAAT<br>ATTAACGCTTACAATTTAGGTGGCACTTTTCGGGGAAATGTG

	**Ampicillin resistance cassette-ColE1 origin of replication-hU6 promoter-gRNA sequence-SpCas9 gRNA scaffold-CMV promoter-NLS-mCherry-GSGM- Hypa-nCas9(D10A)-NLS-PolI5MΔ-stop-SV40 polyA**
p.Slug-nCas9-PolI5MΔ	CGCGGAACCCCTATTTGTTTATTTTTCTAAATACATTCAAATATGTA<br>TCCGCTCATGAGACAATAACCCTGATAAATGCTTCAATAATATTGA<br>AAAAGGAAGAGTATGAGTATTCAACATTTCCGTGTCGCCCTTATTC<br>CCTTTTTTGCGGCATTTTGCCTTCCTGTTTTTGCTCACCCAGAAACG<br>CTGGTGAAAGTAAAAGATGCTGAAGATCAGTTGGGTGCACGAGTG<br>GGTTACATCGAACTGGATCTCAACAGCGGTAAGATCCTTGAGAGT<br>TTTCGCCCCGAAGAACGTTTTCCAATGATGAGCACTTTTAAAGTTC<br>TGCTATGTGGCGCGGTATTATCCCGTATTGACGCCGGGCAAGAGC<br>AACTCGGTCGCCGCATACACTATTCTCAGAATGACTTGGTTGAGTA<br>CTCACCAGTCACAGAAAAGCATCTTACGGATGGCATGACAGTAAG<br>AGAATTATGCAGTGCTGCCATAACCATGAGTGATAACACTGCGGC<br>CAACTTACTTCTGACAACGATCGGAGGACCGAAGGAGCTAACCGC<br>TTTTTTGCACAACATGGGGGATCATGTAACTCGCCTTGATCGTTGG<br>GAACCGGAGCTGAATGAAGCCATACCAAACGACGAGCGTGACACC<br>ACGATGCCTGTAGCAATGGCAACAACGTTGCGCAAACTATTAACT<br>GGCGAACTACTTACTCTAGCTTCCCGGCAACAATTAATAGACTGGA<br>TGGAGGCGGATAAAGTTGCAGGACCACTTCTGCGCTCGGCCCTTCC<br>GGCTGGCTGGTTTATTGCTGATAAATCTGGAGCCGGTGAGCGTGGT<br>TCTCGCGGTATCATTGCAGCACTGGGGCCAGATGGTAAGCCCTCCC<br>GTATCGTAGTTATCTACACGACGGGGAGTCAGGCAACTATGGATG<br>AACGAAATAGACAGATCGCTGAGATAGGTGCCTCACTGATTAAGC<br>ATTGGTAACTGTCAGACCAAGTTTACTCATATATACTTTAGATTGA<br>TTTAAAACTTCATTTTTAATTTAAAAGGATCTAGGTGAAGATCCTT<br>TTTGATAATCTCATGACCAAAATCCCTTAACGTGAGTTTTCGTTCC<br>ACTGAGCGTCAGACCCCGTAGAAAAGATCAAAGGATCTTCTTGAG<br>ATCCTTTTTTTCTGCGCGTAATCTGCTGCTTGCAAACAAAAAAACC<br>ACCGCTACCAGCGGTGGTTTGTTTGCCGGATCAAGAGCTACCAACT<br>CTTTTTCCGAAGGTAACTGGCTTCAGCAGAGCGCAGATACCAAAT<br>ACTGTCCTTCTAGTGTAGCCGTAGTTAGGCCACCACTTCAAGAACT<br>CTGTAGCACCGCCTACATACCTCGCTCTGCTAATCCTGTTACCAGT<br>GGCTGCTGCCAGTGGCGATAAGTCGTGTCTTACCGGGTTGGACTCA<br>AGACGATAGTTACCGGATAAGGCGCAGCGGTCGGGCTGAACGGGG<br>GGTTCGTGCACACAGCCCAGCTTGGAGCGAACGACCTACACCGAA<br>CTGAGATACCTACAGCGTGAGCTATGAGAAAGCGCCACGCTTCCC<br>GAAGGGAGAAAGGCGGACAGGTATCCGGTAAGCGGCAGGGTCGG<br>AACAGGAGAGCGCACGAGGGAGCTTCCAGGGGGAAACGCCTGGT<br>ATCTTTATAGTCCTGTCGGGTTTCGCCACCTCTGACTTGAGCGTCG<br>ATTTTTGTGATGCTCGTCAGGGGGGCGGAGCCTATGGAAAAACGC<br>CAGCAACGCGGCCTTTTTACGGTTCCTGGCCTTTTGCTGGCCTTTTG<br>CTCACATGTTCTTTCCTGCGTTATCCCCTGATTCTGTGGATAACCGT<br>ATTACCGCCTTTGAGTGAGCTGATACCGCTCGCCGCAGCCGAACG

	ACCGAGCGCAGCGAGTCAGTGAGCGAGGAAGCGGAAGAGCGCCC<br>AATACGCAAACCGCCTCTCCCCGCGCGTTGGCCGATTCATTAATGC<br>AGCTGGCACGACAGGTTTCCCGACTGGAAAGCGGGCAGTGAGCGC<br>AACGCAATTAATGTGAGTTAGCTCACTCATTAGGCACCCCAGGCTT<br>TACACTTTATGCTTCCGGCTCGTATGTTGTGTGGAATTGTGAGCGG<br>ATAACAATTTCACACAGGAAACAGCTATGACCATGATTACGCCAA<br>GCGCGCAATTAACCCTCACTAAAGGGAACAAAAGCTGGAGCTCCA<br>CCGCGGTGGCGGCCGCTCTTAAGGGGTGCAGCGGCCTCCGCGCCG<br>GGTTTTGGCGCCTCGATCCAAGGTCGGGCAGGAAGAGGGCCTATT<br>TCCCATGATTCCTTCATATTTGCATATACGATACAAGGCTGTTAGA<br>GAGATAATTAGAATTAATTTGACTGTAAACACAAAGATATTAGTA<br>CAAAATACGTGACGTAGAAAGTAATAATTTCTTGGGTAGTTTGCA<br>GTTTTAAAATTATGTTTTAAAATGGACTATCATATGCTTACCGTAA<br>CTTGAAAGTATTTCGATTTCTTGGCTTTATATATCTTGTGGAAAGG<br>ACGAAACACC-[gRNA SEQUENCE]-<br>GTTTTAGTACTCTGGAAACAGAATCTACTGAAACAAGACAATATG<br>TCGTGTTTATCCCATCAATTTATTGGTGGGATTTTTCCGCGGCCTCT<br>AGACTCGAGGCGTTGACATTGATTATTGACTAGTTATTAATAGTAA<br>TCAATTACGGGGTCATTAGTTCATAGCCCATATATGGAGTTCCGCG<br>TTACATAACTTACGGTAAATGGCCCGCCTGGCTGACCGCCCAACG<br>ACCCCCGCCCATTGACGTCAATAATGACGTATGTTCCCATAGTAAC<br>GCCAATAGGGACTTTCCATTGACGTCAATGGGTGGAGTATTTACGG<br>TAAACTGCCCACTTGGCAGTACATCAAGTGTATCATATGCCAAGTA<br>CGCCCCCTATTGACGTCAATGACGGTAAATGGCCCGCCTGGCATTA<br>TGCCCAGTACATGACCTTATGGGACTTTCCTACTTGGCAGTACATC<br>TACGTATTAGTCATCGCTATTACCATGGTGATGCGGTTTTGGCAGT<br>ACATCAATGGGCGTGGATAGCGGTTTGACTCACGGGGATTTCCAA<br>GTCTCCACCCCATTGACGTCAATGGGAGTTTGTTTTGGCACCAAAA<br>TCAACGGGACTTTCCAAAATGTCGTAACAACTCCGCCCCATTGACG<br>CAAATGGGCGGTAGGCGTGTACGGTGGGAGGTCTATATAAGCAGA<br>GCTCTCTGGCTAACTACCGGTGCCACCATGGCCCCAAAGAAGAAG<br>CGGAAGGTCGGTATCCACGGAGTCCCAGCAGCCGTGAGCAAGGGC<br>GAGGAGGATAACATGGCCATCATCAAGGAGTTCATGCGCTTCAAG<br>GTGCACATGGAGGGCTCCGTGAACGGCCACGAGTTCGAGATCGAG<br>GGCGAGGGCGAGGGCCGCCCCTACGAGGGCACCCAGACCGCCAA<br>GCTGAAGGTGACCAAGGGTGGCCCCCTGCCCTTCGCCTGGGACAT<br>CCTGTCCCCTCAGTTCATGTACGGCTCCAAGGCCTACGTGAAGCAC<br>CCCGCCGACATCCCCGACTACTTGAAGCTGTCCTTCCCCGAGGGCT<br>TCAAGTGGGAGCGCGTGATGAACTTCGAGGACGGCGGCGTGGTGA<br>CCGTGACCCAGGACTCCTCCCTGCAGGACGGCGAGTTCATCTACA<br>AGGTGAAGCTGCGCGGCACCAACTTCCCCTCCGACGGCCCCGTAA<br>TGCAGAAGAAGACCATGGGCTGGGAGGCCTCCTCCGAGCGGATGT<br>ACCCCGAGGACGGCGCCCTGAAGGGCGAGATCAAGCAGAGGCTG<br>AAGCTGAAGGACGGCGGCCACTACGACGCTGAGGTCAAGACCACC<br>TACAAGGCCAAGAAGCCCGTGCAGCTGCCCGGCGCCTACAACGTC<br>AACATCAAGTTGGACATCACCTCCCACAACGAGGACTACACCATC

	GTGGAACAGTACGAACGCGCCGAGGGCCGCCACTCCACCGGCGGC<br>ATGGACGAGCTGTACAAGGGATCCGGTATGAACCAAAAATTCATA<br>CTGGGACTGGCCATCGGAATCACCAGCGTGGGCTACGGCCTGATC<br>GACTACGAGACAAAGAATATCATCGATGCCGGCGTTAGACTGTTC<br>CCCGAGGCCAACGTGGAAAACAACGAGGGAAGAAGGTCCAAACG<br>TGGAAGCAGAAGACTGAAGCGACGCCGCATTCACAGACTTGAACG<br>GGTGAAGAAGCTGCTCGAGGATTATAATCTGCTGGATCAGTCCCA<br>GATTCCTCAGTCTACAAACCCCTACGCCATCCGCGTGAAGGGCCTG<br>TCTGAAGCCCTGAGCAAGGACGAACTCGTGATTGCCCTGCTCCATA<br>TCGCCAAGAGAAGAGGCATCCACAAGATCGACGTGATCGACAGCA<br>ACGACGACGTGGGGAACGAGCTCAGCACCAAGGAACAGCTGAAT<br>AAGAACAGCAAGCTGCTGAAAGACAAATTTGTGTGCCAGATCCAG<br>CTGGAAAGAATGAATGAGGGCCAGGTGCGGGGAGAGAAAAACCG<br>GTTCAAGACCGCTGATATCATCAAGGAAATCATCCAGCTGCTGAA<br>TGTGCAGAAGAACTTCCACCAGCTGGACGAGAACTTCATCAACAA<br>GTACATCGAACTGGTTGAGATGAGGCGGGAATACTTCGAGGGCCC<br>CGGCAAGGGCAGTCCATATGGCTGGGAAGGCGACCCTAAGGCTTG<br>GTACGAGACACTGATGGGCCACTGCACCTACTTCCCAGATGAGCT<br>GAGAAGCGTGAAATACGCCTACAGTGCCGACCTGTTCAACGCTCT<br>GAACGACCTGAACAACCTGGTCATCCAAAGAGATGGACTGTCTAA<br>GCTCGAGTATCATGAGAAGTATCACATCATCGAGAACGTGTTCAA<br>GCAGAAGAAGAAACCTACACTGAAGCAGATCGCCAATGAGATCA<br>ATGTCAACCCTGAAGATATCAAGGGCTACAGAATCACAAAGTCTG<br>GCAAGCCCCAGTTTACCGAGTTTAAGCTCTACCACGACCTGAAAA<br>GCGTGCTGTTTGACCAGAGCATCCTGGAGAACGAAGACGTGCTGG<br>ACCAGATCGCTGAGATCCTGACCATCTACCAGGACAAGGATAGCA<br>TCAAATCTAAGCTGACGGAACTGGACATCCTGCTGAACGAGGAAG<br>ATAAGGAAAACATCGCCCAGCTGACTGGCTACACCGGGACCCACC<br>GGCTCAGCCTGAAATGCATCCGGCTGGTCCTGGAAGAGCAGTGGT<br>ATTCTAGCCGGAATCAGATGGAAATCTTCACACACCTGAACATTA<br>AGCCTAAGAAGATCAACCTGACAGCCGCCAACAAGATCCCGAAGG<br>CTATGATCGACGAGTTCATCCTGAGCCCTGTGGTGAAGAGGACCTT<br>CGGCCAGGCCATTAACCTTATTAACAAGATCATAGAAAAGTACGG<br>CGTGCCTGAAGATATCATCATCGAGCTGGCCAGAGAAAATAATAG<br>CAAGGACAAGCAGAAGTTCATCAATGAGATGCAGAAAAAGAACG<br>AGAACACCAGAAAGAGAATTAACGAAATCATCGGCAAGTATGGC<br>AACCAGAACGCCAAGAGACTGGTCGAGAAGATTAGACTGCACGAC<br>GAGCAGGAGGGCAAGTGCCTGTACTCACTGGAAAGCATCCCTCTG<br>GAGGACCTGCTGAACAACCCCAACCACTACGAGGTGGACCACATC<br>ATTCCAAGATCTGTGTCCTTCGACAACTCTTACCACAACAAAGTGC<br>TCGTGAAGCAGAGCGAGAACTCCAAAAAATCCAACCTGACCCCTT<br>ACCAGTACTTTAACAGCGGCAAGTCCAAGCTCTCTTACAACCAGTT<br>TAAACAACACATCCTGAACCTGAGCAAGTCCCAGGATAGAATCAG<br>CAAAAAAAAGAAAGAGTATCTGCTGGAAGAACGGGACATCAACA<br>AGTTCGAGGTGCAAAAAGAGTTCATCAATAGAAACCTGGTGGATA<br>CCCGGTACGCCACAAGAGAGCTGACAAACTACCTGAAGGCCTACT

	TCAGCGCCAACAATATGAACGTGAAGGTGAAAACGATCAACGGCA<br>GCTTCACCGATTACCTGCGGAAAGTGTGGAAGTTTAAGAAGGAAC<br>GGAACCACGGCTACAAGCACCACGCCGAGGACGCCCTGATTATCG<br>CTAATGCCGATTTCCTGTTCAAAGAGAACAAGAAGCTGAAAGCCG<br>TGAACTCTGTGCTGGAAAAACCTGAGATCGAGAGCAAGCAGCTGG<br>ATATCCAGGTGGATAGCGAGGATAACTACAGCGAAATGTTCATCA<br>TCCCTAAGCAGGTCCAGGACATCAAGGACTTCAGAAACTTCAAGT<br>ACAGCCACAGAGTGGACAAGAAGCCTAACAGACAGCTGATCAAC<br>GATACACTGTACAGCACCCGGAAGAAGGACAACTCCACCTACATC<br>GTGCAGACCATCAAAGATATCTATGCCAAAGATAATACCACCCTG<br>AAGAAGCAGTTTGACAAGTCACCCGAGAAGTTCCTCATGTACCAA<br>CACGATCCGCGGACCTTCGAGAAGTTGGAAGTGATCATGAAGCAG<br>TACGCTAATGAGAAGAATCCTCTGGCCAAGTACCACGAGGAAACA<br>GGCGAGTACCTGACCAAATACAGCAAAAAAAACAACGGCCCTATC<br>GTGAAAAGCCTGAAGTACATTGGAAACAAGCTGGGCAGCCACCTA<br>GATGTGACCCACCAGTTCAAGAGCAGCACCAAGAAGTTGGTGAAG<br>CTGAGCATCAAGCCTTATAGATTCGACGTCTACCTGACCGACAAG<br>GGATATAAGTTCATCACCATCAGCTACCTGGACGTGCTGAAGAAA<br>GACAATTACTACTACATACCCGAACAGAAGTACGACAAGCTCAAA<br>CTGGGCAAGGCCATCGACAAAAACGCCAAGTTTATCGCTAGCTTC<br>TACAAGAATGATCTGATCAAGCTGGACGGCGAGATCTACAAGATC<br>ATCGGCGTGAATAGCGACACCAGAAACATGATCGAACTGGATCTG<br>CCTGACATCAGATACAAAGAATACTGCGAGCTGAACAATATCAAG<br>GGCGAACCTAGAATCAAAAAGACCATCGGCAAAAAGGTGAATAG<br>CATCGAAAAACTGACAACCGACGTGCTGGGCAACGTGTTCACCAA<br>CACCCAGTACACAAAACCTCAGCTGCTGTTCAAGCGAGGAAATGC<br>CTATCCCTATGACGTGCCCGATTATGCCAGCCTGGGCAGCGGCTCC<br>CCCAAGAAAAAACGCAAGGTGGAAGATCCTAAGAAAAAGCGGAA<br>AGGTTCTAGTGAAACCCCGGGAACAAGTGAGTCGGCCACCCCTGA<br>AGGTGGATCAGGGGGTAGCGGATCCTCTTACGATAATTATGTTAC<br>GATTCTGGACGAGGAAACCTTAAAGGCTTGGATCGCTAAATTAGA<br>GAAGGCTCCTGTTTTCGCTTTCGACACGGAAACGGATTCTCTGGAC<br>AATATTAGTGCGAATCTTGTTGGTCTGAGTTTCGCAATTGAACCGG<br>GTGTTGCTGCTTACATCCCTGTGGCACACGACTACCTGGACGCTCC<br>GGACCAGATTTCACGTGAACGCGCTCTGGAACTGCTGAAGCCTTTA<br>TTAGAGGACGAGAAAGCTTTGAAAGTTGGTCAGAATTTGAAGTAT<br>GCTCGTGGAATCTTAGCTAATTATGGTATCGAGTTGCGCGGTATCG<br>CTTTCGACACGATGTTGGAATCTTATATCCTGAACTCTGTCGCTGG<br>TCGCCATGACATGGACTCTCTGGCTGAGCGCTGGCTGAAACATAA<br>GACGATTACCTTCGAGGAAATCGCAGGAAAGGGTAAGAACCAGCT<br>CACGTTCAATCAAATCGCTCTGGAGGAAGCTGGTCGCTATGCTGCT<br>GAGGACGCTGACGTTACTCTGCAACTGCACTTGAAGATGTGGCCT<br>GACTTGCAGAAGCATAAGGGTCCACTGAATGTTTTTGAAAACATT<br>GAGATGCCTTTGGTTCCAGTTCTGTCTCGTATCGAGCGCAATGGCG<br>TTAAAATTGACCCAAAGGTTTTACATAACCACTCAGAGGAACTGA<br>CGCTGCGCTTAGCCGAATTGGAGAAAAAGGCTCACGAGATCGCTG

	GCGAAGAGTTCAATCTGTCATCTACGAAACAACTGCAGACTATCCT<br>GTTCGAGAAGCAAGGTATCAAGCCATTAAAAAAGACCCCTGGCGG<br>TGCTCCGTCTACCTCTGAGGAAGTTTTGGAGGAGTTAGCTTTGGAT<br>TACCCTCTGCCGAAGGTTATCTTGGAATACCGCGGTTTGGCTAAAT<br>TGAAGTCTACTTATACGGATAAACTTCCTTTGATGATTAATCCAAA<br>GACGGGTCGCGTTCACACGTCGTACCATCAAGCTGTTACCGCTACC<br>GGTCGCCTGTCTTCTACGGATCCGAATTTACAGAATATTCCTGTGC<br>GCAATGAGGAGGGCCGCCGCATTCGTCAAGCTTTTATCGCTCCGG<br>AAGACTACGTTATCGTTTCTGCTGATTATTCTCAAAATGAATTACG<br>TATCATGGCTCACCTGTCTCGCGATAAGGGTCTGTTGACGGCCTTT<br>GCTGAGGGTAAGGACATTCATCGTGCTACCGCTGCTGAGGTTTACG<br>GCCTGCCGTTGGAAACGGTTACGTCTGAACAGCGTCGCTCTGCTAA<br>GCGTATTAATTTCGGCTTAATCTACGGTATGTCTGCGTTTGGCTTA<br>GCTCGTCAGCTGAATATCCCGCGCAAGGAAGCTCAAAAATATATG<br>GATCTGTATTTTGAGCGTTACCACGGTGTTTTGGAATACATGGAGC<br>GTACGCGCGCGCAAGCTAAGGAACAAGGTTATGTGGAAACCTTGG<br>ATGGTCGTCGCTTGTACTTGCCTGACATTAAGTCTTCTAACGGCGC<br>CCGCCGCGCTGCTGCCGAGCGCGCTGCTATCAATGCTCCGATGCAA<br>GGTACTGCTGCTGATATTATTAAGCGTGCTATGATCGCTGTGGACG<br>CTTGGCTGCAAGCTGAACAGCCTCGCGTTCGCATGATTATGCAAGT<br>TCATGACGAGTTGGTTTTCGAGGTGCATAAGGACGACGTGGACGC<br>TGTTGCTAAACAAATCCACCAGTTGATGGAGAATTGCACGCGCTTA<br>GACGTTCCGCTGCTGGTTGAAGTTGGTTCTGGTGAAAACTGGGACC<br>AGGCTCACTAATGGCTGAAAACTGGGACCAGGCTCACTAACTCAG<br>ATCCTACTAGGTTTAATAAACATCTTTATTTTCATTACATCTGTGTG<br>TTGGTTTTTTGTGTGGTACCCAATTCGCCCTATAGTGAGTCGTATTA<br>CTCACTGGCCGTCGTTTTACAACGTCGTGACTGGGAAAACCCTGGC<br>GTTACCCAACTTAATCGCCTTGCAGCACATCCCCCTTTCGCCAGCT<br>GGCGTAATAGCGAAGAGGCCCGCACCGATCGCCCTTCCCAACAGT<br>TGCGCAGCCTGAATGGCGAATGGGACGCGCCCTGTAGCGGCGCAT<br>TAAGCGCGGCGGGTGTGGTGGTTACGCGCAGCGTGACCGCTACAC<br>TTGCCAGCGCCCTAGCGCCCGCTCCTTTCGCTTTCTTCCCTTCCTTT<br>CTCGCCACGTTCGCCGGCTTTCCCCGTCAAGCTCTAAATCGGGGGC<br>TCCCTTTAGGGTTCCGATTTAGTGCTTTACGGCACCTCGACCCCAA<br>AAAACTTGATTAGGGTGATGGTTCACGTAGTGGGCCATCGCCCTG<br>ATAGACGGTTTTTCGCCCTTTGACGTTGGAGTCCACGTTCTTTAAT<br>AGTGGACTCTTGTTCCAAACTGGAACAACACTCAACCCTATCTCGG<br>TCTATTCTTTTGATTTATAAGGGATTTTGCCGATTTCGGCCTATTGG<br>TTAAAAAATGAGCTGATTTAACAAAAATTTAACGCGAATTTTAAC<br>AAAATATTAACGCTTACAATTTAGGTGGCACTTTTCGGGGAAATGT<br>G<br><br>**Ampicillin resistance cassette-ColE1 origin of replication-hU6 promoter-gRNA sequence-SlugCas9 gRNA scaffold-CMV promoter-NLS-mCherry-GSGM- Slug-nCas9(D10A)-NLS-PolI5MΔ-stop-SV40 polyA**

p.eNme2.C.NR-nCas9-PolI5MΔ	CGCGGAACCCCTATTTGTTTATTTTTCTAAATACATTCAAATATGTA<br>TCCGCTCATGAGACAATAACCCTGATAAATGCTTCAATAATATTGA<br>AAAAGGAAGAGTATGAGTATTCAACATTTCCGTGTCGCCCTTATTC<br>CCTTTTTTGCGGCATTTTGCCTTCCTGTTTTTGCTCACCCAGAAACG<br>CTGGTGAAAGTAAAAGATGCTGAAGATCAGTTGGGTGCACGAGTG<br>GGTTACATCGAACTGGATCTCAACAGCGGTAAGATCCTTGAGAGT<br>TTTCGCCCCGAAGAACGTTTTCCAATGATGAGCACTTTTAAAGTTC<br>TGCTATGTGGCGCGGTATTATCCCGTATTGACGCCGGGCAAGAGC<br>AACTCGGTCGCCGCATACACTATTCTCAGAATGACTTGGTTGAGTA<br>CTCACCAGTCACAGAAAAGCATCTTACGGATGGCATGACAGTAAG<br>AGAATTATGCAGTGCTGCCATAACCATGAGTGATAACACTGCGGC<br>CAACTTACTTCTGACAACGATCGGAGGACCGAAGGAGCTAACCGC<br>TTTTTTGCACAACATGGGGGATCATGTAACTCGCCTTGATCGTTGG<br>GAACCGGAGCTGAATGAAGCCATACCAAACGACGAGCGTGACACC<br>ACGATGCCTGTAGCAATGGCAACAACGTTGCGCAAACTATTAACT<br>GGCGAACTACTTACTCTAGCTTCCCGGCAACAATTAATAGACTGGA<br>TGGAGGCGGATAAAGTTGCAGGACCACTTCTGCGCTCGGCCCTTCC<br>GGCTGGCTGGTTTATTGCTGATAAATCTGGAGCCGGTGAGCGTGGT<br>TCTCGCGGTATCATTGCAGCACTGGGGCCAGATGGTAAGCCCTCCC<br>GTATCGTAGTTATCTACACGACGGGGAGTCAGGCAACTATGGATG<br>AACGAAATAGACAGATCGCTGAGATAGGTGCCTCACTGATTAAGC<br>ATTGGTAACTGTCAGACCAAGTTTACTCATATATACTTTAGATTGA<br>TTTAAAACTTCATTTTTAATTTAAAAGGATCTAGGTGAAGATCCTT<br>TTTGATAATCTCATGACCAAAATCCCTTAACGTGAGTTTTCGTTCC<br>ACTGAGCGTCAGACCCCGTAGAAAAGATCAAAGGATCTTCTTGAG<br>ATCCTTTTTTTCTGCGCGTAATCTGCTGCTTGCAAACAAAAAAACC<br>ACCGCTACCAGCGGTGGTTTGTTTGCCGGATCAAGAGCTACCAACT<br>CTTTTTCCGAAGGTAACTGGCTTCAGCAGAGCGCAGATACCAAAT<br>ACTGTCCTTCTAGTGTAGCCGTAGTTAGGCCACCACTTCAAGAACT<br>CTGTAGCACCGCCTACATACCTCGCTCTGCTAATCCTGTTACCAGT<br>GGCTGCTGCCAGTGGCGATAAGTCGTGTCTTACCGGGTTGGACTCA<br>AGACGATAGTTACCGGATAAGGCGCAGCGGTCGGGCTGAACGGGG<br>GGTTCGTGCACACAGCCCAGCTTGGAGCGAACGACCTACACCGAA<br>CTGAGATACCTACAGCGTGAGCTATGAGAAAGCGCCACGCTTCCC<br>GAAGGGAGAAAGGCGGACAGGTATCCGGTAAGCGGCAGGGTCGG<br>AACAGGAGAGCGCACGAGGGAGCTTCCAGGGGGAAACGCCTGGT<br>ATCTTTATAGTCCTGTCGGGTTTCGCCACCTCTGACTTGAGCGTCG<br>ATTTTTGTGATGCTCGTCAGGGGGGCGGAGCCTATGGAAAAACGC<br>CAGCAACGCGGCCTTTTTACGGTTCCTGGCCTTTTGCTGGCCTTTTG<br>CTCACATGTTCTTTCCTGCGTTATCCCCTGATTCTGTGGATAACCGT<br>ATTACCGCCTTTGAGTGAGCTGATACCGCTCGCCGCAGCCGAACG<br>ACCGAGCGCAGCGAGTCAGTGAGCGAGGAAGCGGAAGAGCGCCC<br>AATACGCAAACCGCCTCTCCCCGCGCGTTGGCCGATTCATTAATGC<br>AGCTGGCACGACAGGTTTCCCGACTGGAAAGCGGGCAGTGAGCGC<br>AACGCAATTAATGTGAGTTAGCTCACTCATTAGGCACCCCAGGCTT

	TACACTTTATGCTTCCGGCTCGTATGTTGTGTGGAATTGTGAGCGG<br>ATAACAATTTCACACAGGAAACAGCTATGACCATGATTACGCCAA<br>GCGCGCAATTAACCCTCACTAAAGGGAACAAAAGCTGGAGCTCCA<br>CCGCGGTGGCGGCCGCTCTTAAGGGGTGCAGCGGCCTCCGCGCCG<br>GGTTTTGGCGCCTCGATCCAAGGTCGGGCAGGAAGAGGGCCTATT<br>TCCCATGATTCCTTCATATTTGCATATACGATACAAGGCTGTTAGA<br>GAGATAATTAGAATTAATTTGACTGTAAACACAAAGATATTAGTA<br>CAAAATACGTGACGTAGAAAGTAATAATTTCTTGGGTAGTTTGCA<br>GTTTTAAAATTATGTTTTAAAATGGACTATCATATGCTTACCGTAA<br>CTTGAAAGTATTTCGATTTCTTGGCTTTATATATCTTGTGGAAAGG<br>ACGAAACACC-[gRNA SEQUENCE]-<br>GTTGTAGCTCCCTTTCTCATTTCGGAAACGAAATGAGAACCGTTGC<br>TACAATAAGGCCGTCTGAAAAGATGTGCCGCAACGCTCTGCCCCTT<br>AAAGCTTCTGCTTTAAGGGGCATCGTTTATTTTCCGCGGCCTCTAG<br>ACTCGAGGCGTTGACATTGATTATTGACTAGTTATTAATAGTAATC<br>AATTACGGGGTCATTAGTTCATAGCCCATATATGGAGTTCCGCGTT<br>ACATAACTTACGGTAAATGGCCCGCCTGGCTGACCGCCCAACGAC<br>CCCCGCCCATTGACGTCAATAATGACGTATGTTCCCATAGTAACGC<br>CAATAGGGACTTTCCATTGACGTCAATGGGTGGAGTATTTACGGTA<br>AACTGCCCACTTGGCAGTACATCAAGTGTATCATATGCCAAGTACG<br>CCCCCTATTGACGTCAATGACGGTAAATGGCCCGCCTGGCATTATG<br>CCCAGTACATGACCTTATGGGACTTTCCTACTTGGCAGTACATCTA<br>CGTATTAGTCATCGCTATTACCATGGTGATGCGGTTTTGGCAGTAC<br>ATCAATGGGCGTGGATAGCGGTTTGACTCACGGGGATTTCCAAGT<br>CTCCACCCCATTGACGTCAATGGGAGTTTGTTTTGGCACCAAAATC<br>AACGGGACTTTCCAAAATGTCGTAACAACTCCGCCCCATTGACGC<br>AAATGGGCGGTAGGCGTGTACGGTGGGAGGTCTATATAAGCAGAG<br>CTCTCTGGCTAACTACCGGTGCCACCATGGCCCCAAAGAAGAAGC<br>GGAAGGTCGGTATCCACGGAGTCCCAGCAGCCGTGAGCAAGGGCG<br>AGGAGGATAACATGGCCATCATCAAGGAGTTCATGCGCTTCAAGG<br>TGCACATGGAGGGCTCCGTGAACGGCCACGAGTTCGAGATCGAGG<br>GCGAGGGCGAGGGCCGCCCCTACGAGGGCACCCAGACCGCCAAG<br>CTGAAGGTGACCAAGGGTGGCCCCCTGCCCTTCGCCTGGGACATC<br>CTGTCCCCTCAGTTCATGTACGGCTCCAAGGCCTACGTGAAGCACC<br>CCGCCGACATCCCCGACTACTTGAAGCTGTCCTTCCCCGAGGGCTT<br>CAAGTGGGAGCGCGTGATGAACTTCGAGGACGGCGGCGTGGTGAC<br>CGTGACCCAGGACTCCTCCCTGCAGGACGGCGAGTTCATCTACAA<br>GGTGAAGCTGCGCGGCACCAACTTCCCCTCCGACGGCCCCGTAAT<br>GCAGAAGAAGACCATGGGCTGGGAGGCCTCCTCCGAGCGGATGTA<br>CCCCGAGGACGGCGCCCTGAAGGGCGAGATCAAGCAGAGGCTGA<br>AGCTGAAGGACGGCGGCCACTACGACGCTGAGGTCAAGACCACCT<br>ACAAGGCCAAGAAGCCCGTGCAGCTGCCCGGCGCCTACAACGTCA<br>ACATCAAGTTGGACATCACCTCCCACAACGAGGACTACACCATCG<br>TGGAACAGTACGAACGCGCCGAGGGCCGCCACTCCACCGGCGGCA<br>TGGACGAGCTGTACAAGGGATCCGGTATGGCCGCCTTCAAGCCTA<br>ACCCAATCAATTACATCCTGGGACTGGCAATCGGAATCGCATCCGT

	GGGATGGGCTATGGTGGAGATCGACGAGGAGGAGAATCCTATCCG<br>GCTGATCGATCTGGGCGTGAGAGTGTTTGAGAGGGCCGAGGTGCC<br>AAAGACCGGCGATTCTCTGGCTATGGCCCGGAGACTGGCACGGAG<br>CGTGAGGCGCCTGACACGGAGAAGGGCACACAGGCTGCTGAGGG<br>CACGCCGGCTGCTGAAGAGAGAGGGCGTGCTGCAGGCAGCAGACT<br>TCGATGAGAATGGCCTGATCACAAGCCTGCCAAACACCCCCTGGC<br>AGCTGAGAGCAGCCGCCCTGGACAGGAAGCTGACACCACTGGAGT<br>GGTCTGCCGTGCTGCTGCACCTGATCAAGCACCGCGGCTACCTGAG<br>CCAGCGGAAGAACGAGGGAGAGACAGCAGCCAAGGAGCTGGGCG<br>CCCTGCTGAAGGGAGTGGCCAACAATGCCCACGCCCTGCAGACCG<br>GCGATTTCAGGACACCTGCCGAGCTGGCCCTGAATAAGTTTGAGA<br>AGGAGTCCGGCCACATCAGAAACCAGAGGGGCGACTATAGCCACA<br>CCTTCTCCCGCAAGGATCTGCAGGCCGAGCTGATCCTGCTGTTCGA<br>GAAGCAGAAGGAGTTTGGCAATCCACACGTGAGCGGAGGCCTGAA<br>GGAGGGAATCGAAACCCTGCTGATGACACAGAGGCCTGCCCTGTC<br>CGGCGACGCAGTGCAGAAGATGCTGGGACACTGCACCCTGGAGCC<br>TACCGAGCCAAAGGCCGCCAAGAACACCTACACAGCCGAGCGGTT<br>TATCTGGCTGACAAAGCTGAACAATCTGAGAATCCTGGAGCAGGG<br>ATCCGAGAGGCCACTGACCGACACAGAGAGGTCCACCCTGATGGA<br>TGAGCCTTACCGGAAGTCTAAGCTGACATATGCCCAGGCCAGAAA<br>GCTGCTGGGCCTGGAGGACACCGCCTTCTTTAAGGGCCTGAGATA<br>CGGCAAGGATAATGCCGAGGCCTCCACACTGATGGAGATGAAGGC<br>CTATCACGCCATCTCTCGCGCCCTGGAGAAGGAGGGCCTGAAGGA<br>CAAGAAGTCCCCCCTGAACCTGAGCTCCGAGCTGCAGGATGAGAT<br>CGGCACCGCCTTCTCTCTGTTTAAGACCGACGAGGATATCACAGGC<br>CGCCTGAAGGACAGGGTGCAGCCTGAGATCCTGGAGGCCCTGCTG<br>AAGCACATCTCTTTCGATAAGTTTGTGCAGATCAGCCTGAAGGCCC<br>TGAGAAGGATCGTGCCACTGATGGAGCAGGGCAAGCGGTACGACG<br>AGGCCTGCGCCGAGATCTACGGCGTGCACTATGGCAAGAAGAACA<br>CAGAGGAGAAGATCTATCTGCCCCCTATCCCTGCCGACGAGATCA<br>GAAATCCTGTGGTGCTGAGGGCCCTGTCCCAGGCAAGAAAAGTGA<br>TCAACGGAGTGGTGCGCCGGTACGGATCTCCAGCCCGGATCCACA<br>TCGAAACCGCCAGAGAAGTGGGCAAGAGCTTCAAGGACCGGAAG<br>GAGATCGAGAAGAGACAGGAGGAGAATCGCAAGGATCGGGAGAA<br>GGCCGCCGCCAAGTTTAGGGAGTACTTCCCTAACTTTGTGGGCGAG<br>CCAAAGTCTAAGGACATCCTGAAGCTGCGCCTGTACGAGCAGCAG<br>CACGGCAAGTGTCTGTATAGCGGCAAGGAGATCAATCTGGTGCGG<br>CTGAACGAGAAGGGCTATGTGGAGATCGATCACGCCCTGCCTTTCT<br>CCAGAACCTGGGACGATTCTTTTAACAATAAGGTGCTGGTGCTGG<br>GCAGCGAGAACCAGAATAAGGGCAATCAGACACCATACGAGTATT<br>TCAATGGCAAGGACAACTCCAGGGAGTGGCAGGAGTTCAAGGCCC<br>GCGTGGAAACCTCTAGATTTCCCAGGAGCAAGAAGCAGCGGATCC<br>TGCTGCAGAAGTTCGACGAGGATGGCTTTAAGGAGTGCAACCTGA<br>ATGACACCAGATACGTGAACCGGTTCCTGTGCCAGTTTGTGGCCGA<br>TCACATCCTGCTGACCGGCAAGGGCAAGAGAAGGGTGTTCGCCTC<br>TAATGGCCAGATCACAAACCTGCTGAGGGGATTTTGGGACTGAG

	GAAGGTGCGGGCAGAGAATGACAGACACCACGCACTGGATGCAG<br>TGGTGGTGGCATGCAGCACCGTGGCAATGCAGCAGAAGATCACAA<br>GATTCGTGAGGTATAAGGAGATGAACGCCTTTGACGGCAAGACCA<br>TCGATAAGGAGACAGGCAAGGTGCTGCACCAGAAGACCCACTTCC<br>CCCAGCCTTGGGAGTTCTTTGCCCAGGAAGTGATGATCCGGGTGTT<br>CGGCAAGCCAGACGGCAAGCCTGAGTTTGAGGAGGCCGATACCCC<br>AGAGAAGCTGAGGACACTGCTGGCAGAGAAGCTGTCTAGCAGGCC<br>AGAGGCAGTGCACGAGTACGTGACCCCACTGTTCGTGTCCAGGGC<br>ACCCAATCGGAAGATGTCTGGCGCCCACAAGGACACACTGAGAAG<br>CGCCAAGAGGTTTGTGAAGCACAACGAGAAGATCTCCGTGAAGAG<br>AGTGTGGCTGACCGAGATCAAGCTGGCCGATCTGGAGAACATGGT<br>GAATTACAAGAACGGCAGGGAGATCGAGCTGTATGAGGCCCTGAA<br>GGCAAGGCTGGAGGCCTACGGAGGAAATGCCAAGCAGGCCTTCGA<br>CCCAAAGGATAACCCCTTTTATAAGAAGGGAGGACAGCTGGTGAA<br>GGCCGTGCGGGTGGAGAAGACCCAGAAGAGCGGCGTGCTGCTGA<br>ATAAGAAGAACGCCTACACAATCGCCGACAATGGCGATATGGTGA<br>GAGTGGACGTGTTCTGTAAGGTGGATAAGAAGGGCAAGAATCAGT<br>ACTTTATCGTGCCTATCTATGCCTGGCAGGTGGCCGAGAACATCCT<br>GCCAGACATCGATTGCAAGGGCTACAGAATCGACGATAGCTATAC<br>ATTCTGTTTTTCCCTGCACAAGTATGACCTGATCGCCTTCCAGAAG<br>GATGAGAAGTCCAAGGTGGAGTTTGCCTACTATATCAATTGCGACT<br>CCTCTAGCGGCGGCTTCTACCTGGCCTGGCACGATAAGGGCAGCC<br>GCGAGCAGCGCTTTCGCATCTCCACCCAGAATCTGGCCCTGATCCA<br>GAAGTATCAGGTGAACGAGCTGGGCAAGGAGATCAGGCCATGTCG<br>GCTGAAGAAGCGCCCACCCGTGCGGGCCTATCCCTATGACGTGCC<br>CGATTATGCCAGCCTGGGCAGCGGCTCCCCCAAGAAAAAACGCAA<br>GGTGGAAGATCCTAAGAAAAAGCGGAAAGGTTCTAGTGAAACCCC<br>GGGAACAAGTGAGTCGGCCACCCCTGAAGGTGGATCAGGGGGTAG<br>CGGATCCTCTTACGATAATTATGTTACGATTCTGGACGAGGAAACC<br>TTAAAGGCTTGGATCGCTAAATTAGAGAAGGCTCCTGTTTTCGCTT<br>TCGACACGGAAACGGATTCTCTGGACAATATTAGTGCGAATCTTGT<br>TGGTCTGAGTTTCGCAATTGAACCGGGTGTTGCTGCTTACATCCCT<br>GTGGCACACGACTACCTGGACGCTCCGGACCAGATTTCACGTGAA<br>CGCGCTCTGGAACTGCTGAAGCCTTTATTAGAGGACGAGAAAGCT<br>TTGAAAGTTGGTCAGAATTTGAAGTATGCTCGTGGAATCTTAGCTA<br>ATTATGGTATCGAGTTGCGCGGTATCGCTTTCGACACGATGTTGGA<br>ATCTTATATCCTGAACTCTGTCGCTGGTCGCCATGACATGGACTCT<br>CTGGCTGAGCGCTGGCTGAAACATAAGACGATTACCTTCGAGGAA<br>ATCGCAGGAAAGGGTAAGAACCAGCTCACGTTCAATCAAATCGCT<br>CTGGAGGAAGCTGGTCGCTATGCTGCTGAGGACGCTGACGTTACT<br>CTGCAACTGCACTTGAAGATGTGGCCTGACTTGCAGAAGCATAAG<br>GGTCCACTGAATGTTTTTGAAAACATTGAGATGCCTTTGGTTCCAG<br>TTCTGTCTCGTATCGAGCGCAATGGCGTTAAAATTGACCCAAAGGT<br>TTTACATAACCACTCAGAGGAACTGACGCTGCGCTTAGCCGAATTG<br>GAGAAAAAGGCTCACGAGATCGCTGGCGAAGAGTTCAATCTGTCA<br>TCTACGAAACAACTGCAGACTATCCTGTTCGAGAAGCAAGGTATC

	AAGCCATTAAAAAAGACCCCTGGCGGTGCTCCGTCTACCTCTGAG<br>GAAGTTTTGGAGGAGTTAGCTTTGGATTACCCTCTGCCGAAGGTTA<br>TCTTGGAATACCGCGGTTTGGCTAAATTGAAGTCTACTTATACGGA<br>TAAACTTCCTTTGATGATTAATCCAAAGACGGGTCGCGTTCACACG<br>TCGTACCATCAAGCTGTTACCGCTACCGGTCGCCTGTCTTCTACGG<br>ATCCGAATTTACAGAATATTCCTGTGCGCAATGAGGAGGGCCGCC<br>GCATTCGTCAAGCTTTTATCGCTCCGGAAGACTACGTTATCGTTTC<br>TGCTGATTATTCTCAAAATGAATTACGTATCATGGCTCACCTGTCT<br>CGCGATAAGGGTCTGTTGACGGCCTTTGCTGAGGGTAAGGACATT<br>CATCGTGCTACCGCTGCTGAGGTTTACGGCCTGCCGTTGGAAACGG<br>TTACGTCTGAACAGCGTCGCTCTGCTAAGCGTATTAATTTCGGCTT<br>AATCTACGGTATGTCTGCGTTTGGCTTAGCTCGTCAGCTGAATATC<br>CCGCGCAAGGAAGCTCAAAAATATATGGATCTGTATTTTGAGCGTT<br>ACCACGGTGTTTTGGAATACATGGAGCGTACGCGCGCGCAAGCTA<br>AGGAACAAGGTTATGTGGAAACCTTGGATGGTCGTCGCTTGTACTT<br>GCCTGACATTAAGTCTTCTAACGGCGCCCGCCGCGCTGCTGCCGAG<br>CGCGCTGCTATCAATGCTCCGATGCAAGGTACTGCTGCTGATATTA<br>TTAAGCGTGCTATGATCGCTGTGGACGCTTGGCTGCAAGCTGAACA<br>GCCTCGCGTTCGCATGATTATGCAAGTTCATGACGAGTTGGTTTTC<br>GAGGTGCATAAGGACGACGTGGACGCTGTTGCTAAACAAATCCAC<br>CAGTTGATGGAGAATTGCACGCGCTTAGACGTTCCGCTGCTGGTTG<br>AAGTTGGTTCTGGTGAAAACTGGGACCAGGCTCACTAATGGCTGA<br>AAACTGGGACCAGGCTCACTAACTCAGATCCTACTAGGTTTAATA<br>AACATCTTTATTTTCATTACATCTGTGTGTTGGTTTTTTGTGTGGTA<br>CCCAATTCGCCCTATAGTGAGTCGTATTACTCACTGGCCGTCGTTT<br>TACAACGTCGTGACTGGGAAAACCCTGGCGTTACCCAACTTAATC<br>GCCTTGCAGCACATCCCCCTTTCGCCAGCTGGCGTAATAGCGAAGA<br>GGCCCGCACCGATCGCCCTTCCCAACAGTTGCGCAGCCTGAATGG<br>CGAATGGGACGCGCCCTGTAGCGGCGCATTAAGCGCGGCGGGTGT<br>GGTGGTTACGCGCAGCGTGACCGCTACACTTGCCAGCGCCCTAGC<br>GCCCGCTCCTTTCGCTTTCTTCCCTTCCTTTCTCGCCACGTTCGCCG<br>GCTTTCCCCGTCAAGCTCTAAATCGGGGGCTCCCTTTAGGGTTCCG<br>ATTTAGTGCTTTACGGCACCTCGACCCCAAAAAACTTGATTAGGGT<br>GATGGTTCACGTAGTGGGCCATCGCCCTGATAGACGGTTTTTCGCC<br>CTTTGACGTTGGAGTCCACGTTCTTTAATAGTGGACTCTTGTTCCA<br>AACTGGAACAACACTCAACCCTATCTCGGTCTATTCTTTTGATTTA<br>TAAGGGATTTTGCCGATTTCGGCCTATTGGTTAAAAAATGAGCTGA<br>TTTAACAAAAATTTAACGCGAATTTTAACAAAATATTAACGCTTAC<br>AATTTAGGTGGCACTTTTCGGGGAAATGTG<br><br>**Ampicillin resistance cassette-ColE1 origin of replication-hU6 promoter-gRNA sequence-Nme2Cas9 gRNA scaffold-CMV promoter-NLS-mCherry-GSGM- eNme2.NR-nCas9(D16A)-NLS-PolI5MΔ-stop-SV40 polyA**

p.NNGSlug-nCas9-PolI5MΔ	CGCGGAACCCCTATTTGTTTATTTTTCTAAATACATTCAAATATGTATCCGCTCATGAGACAATAACCCTGATAAATGCTTCAATAATATTGAAAAAGGAAGAGTATGAGTATTCAACATTTCCGTGTCGCCCTTATTCCCTTTTTTGCGGCATTTTGCCTTCCTGTTTTTGCTCACCCAGAAACGCTGGTGAAAGTAAAAGATGCTGAAGATCAGTTGGGTGCACGAGTGGGTTACATCGAACTGGATCTCAACAGCGGTAAGATCCTTGAGAGTTTTCGCCCCGAAGAACGTTTTCCAATGATGAGCACTTTTAAAGTTCTGCTATGTGGCGCGGTATTATCCCGTATTGACGCCGGGCAAGAGCAACTCGGTCGCCGCATACACTATTCTCAGAATGACTTGGTTGAGTACTCACCAGTCACAGAAAAGCATCTTACGGATGGCATGACAGTAAGAGAATTATGCAGTGCTGCCATAACCATGAGTGATAACACTGCGGCCAACTTACTTCTGACAACGATCGGAGGACCGAAGGAGCTAACCGCTTTTTTGCACAACATGGGGGATCATGTAACTCGCCTTGATCGTTGGGAACCGGAGCTGAATGAAGCCATACCAAACGACGAGCGTGACACCACGATGCCTGTAGCAATGGCAACAACGTTGCGCAAACTATTAACTGGCGAACTACTTACTCTAGCTTCCCGGCAACAATTAATAGACTGGATGGAGGCGGATAAAGTTGCAGGACCACTTCTGCGCTCGGCCCTTCCGGCTGGCTGGTTTATTGCTGATAAATCTGGAGCCGGTGAGCGTGGTTCTCGCGGTATCATTGCAGCACTGGGGCCAGATGGTAAGCCCTCCCGTATCGTAGTTATCTACACGACGGGGAGTCAGGCAACTATGGATGAACGAAATAGACAGATCGCTGAGATAGGTGCCTCACTGATTAAGCATTGGTAACTGTCAGACCAAGTTTACTCATATATACTTTAGATTGATTTAAAACTTCATTTTTAATTTAAAAGGATCTAGGTGAAGATCCTTTTTGATAATCTCATGACCAAAATCCCTTAACGTGAGTTTTCGTTCCACTGAGCGTCAGACCCCGTAGAAAAGATCAAAGGATCTTCTTGAGATCCTTTTTTTCTGCGCGTAATCTGCTGCTTGCAAACAAAAAAACCACCGCTACCAGCGGTGGTTTGTTTGCCGGATCAAGAGCTACCAACTCTTTTTCCGAAGGTAACTGGCTTCAGCAGAGCGCAGATACCAAATACTGTCCTTCTAGTGTAGCCGTAGTTAGGCCACCACTTCAAGAACTCTGTAGCACCGCCTACATACCTCGCTCTGCTAATCCTGTTACCAGTGGCTGCTGCCAGTGGCGATAAGTCGTGTCTTACCGGGTTGGACTCAAGACGATAGTTACCGGATAAGGCGCAGCGGTCGGGCTGAACGGGGGGTTCGTGCACACAGCCCAGCTTGGAGCGAACGACCTACACCGAACTGAGATACCTACAGCGTGAGCTATGAGAAAGCGCCACGCTTCCCGAAGGGAGAAAGGCGGACAGGTATCCGGTAAGCGGCAGGGTCGGAACAGGAGAGCGCACGAGGGAGCTTCCAGGGGGAAACGCCTGGTATCTTTATAGTCCTGTCGGGTTTCGCCACCTCTGACTTGAGCGTCGATTTTTGTGATGCTCGTCAGGGGGGCGGAGCCTATGGAAAAACGCCAGCAACGCGGCCTTTTTACGGTTCCTGGCCTTTTGCTGGCCTTTTGCTCACATGTTCTTTCCTGCGTTATCCCCTGATTCTGTGGATAACCGTATTACCGCCTTTGAGTGAGCTGATACCGCTCGCCGCAGCCGAACGACCGAGCGCAGCGAGTCAGTGAGCGAGGAAGCGGAAGAGCGCCCAATACGCAAACCGCCTCTCCCCGCGCGTTGGCCGATTCATTAATGCAGCTGGCACGACAGGTTTCCCGACTGGAAAGCGGGCAGTGAGCGCAACGCAATTAATGTGAGTTAGCTCACTCATTAGGCACCCCAGGCTT

	TACACTTTATGCTTCCGGCTCGTATGTTGTGTGGAATTGTGAGCGG<br>ATAACAATTTCACACAGGAAACAGCTATGACCATGATTACGCCAA<br>GCGCGCAATTAACCCTCACTAAAGGGAACAAAAGCTGGAGCTCCA<br>CCGCGGTGGCGGCCGCTCTTAAGGGGTGCAGCGGCCTCCGCGCCG<br>GGTTTTGGCGCCTCGATCCAAGGTCGGCAGGAAGAGGGCCTATT<br>TCCCATGATTCCTTCATATTTGCATATACGATACAAGGCTGTTAGA<br>GAGATAATTAGAATTAATTTGACTGTAAACACAAAGATATTAGTA<br>CAAAATACGTGACGTAGAAAGTAATAATTTCTTGGGTAGTTTGCA<br>GTTTTAAAATTATGTTTTAAAATGGACTATCATATGCTTACCGTAA<br>CTTGAAAGTATTTCGATTTCTTGGCTTTATATATCTTGTGGAAAGG<br>ACGAAACACC-[gRNA SEQUENCE]-<br>GTTTTAGTACTCTGGAAACAGAATCTACTGAAACAAGACAATATG<br>TCGTGTTTATCCCATCAATTTATTGGTGGGATTTTTCCGCGGCCTCT<br>AGACTCGAGGCGTTGACATTGATTATTGACTAGTTATTAATAGTAA<br>TCAATTACGGGGTCATTAGTTCATAGCCCATATATGGAGTTCCGCG<br>TTACATAACTTACGGTAAATGGCCCGCCTGGCTGACCGCCCAACG<br>ACCCCCGCCCATTGACGTCAATAATGACGTATGTTCCCATAGTAAC<br>GCCAATAGGGACTTTCCATTGACGTCAATGGGTGGAGTATTTACGG<br>TAAACTGCCCACTTGGCAGTACATCAAGTGTATCATATGCCAAGTA<br>CGCCCCCTATTGACGTCAATGACGGTAAATGGCCCGCCTGGCATTA<br>TGCCCAGTACATGACCTTATGGGACTTTCCTACTTGGCAGTACATC<br>TACGTATTAGTCATCGCTATTACCATGGTGATGCGGTTTTGGCAGT<br>ACATCAATGGGCGTGGATAGCGGTTTGACTCACGGGGATTTCCAA<br>GTCTCCACCCCATTGACGTCAATGGGAGTTTGTTTTGGCACCAAAA<br>TCAACGGGACTTTCCAAAATGTCGTAACAACTCCGCCCCATTGACG<br>CAAATGGGCGGTAGGCGTGTACGGTGGGAGGTCTATATAAGCAGA<br>GCTCTCTGGCTAACTACCGGTGCCACCATGGCCCCAAAGAAGAAG<br>CGGAAGGTCGGTATCCACGGAGTCCCAGCAGCCGTGAGCAAGGGC<br>GAGGAGGATAACATGGCCATCATCAAGGAGTTCATGCGCTTCAAG<br>GTGCACATGGAGGGCTCCGTGAACGGCCACGAGTTCGAGATCGAG<br>GGCGAGGGCGAGGGCCGCCCCTACGAGGGCACCCAGACCGCCAA<br>GCTGAAGGTGACCAAGGGTGGCCCCCTGCCCTTCGCCTGGGACAT<br>CCTGTCCCCTCAGTTCATGTACGGCTCCAAGGCCTACGTGAAGCAC<br>CCCGCCGACATCCCCGACTACTTGAAGCTGTCCTTCCCCGAGGGCT<br>TCAAGTGGGAGCGCGTGATGAACTTCGAGGACGGCGGCGTGGTGA<br>CCGTGACCCAGGACTCCTCCCTGCAGGACGGCGAGTTCATCTACA<br>AGGTGAAGCTGCGCGGCACCAACTTCCCCTCCGACGGCCCCGTAA<br>TGCAGAAGAAGACCATGGGCTGGGAGGCCTCCTCCGAGCGGATGT<br>ACCCCGAGGACGGCGCCCTGAAGGGCGAGATCAAGCAGAGGCTG<br>AAGCTGAAGGACGGCGGCCACTACGACGCTGAGGTCAAGACCACC<br>TACAAGGCCAAGAAGCCCGTGCAGCTGCCCGGCGCCTACAACGTC<br>AACATCAAGTTGGACATCACCTCCCACAACGAGGACTACACCATC<br>GTGGAACAGTACGAACGCGCCGAGGGCCGCCACTCCACCGGCGGC<br>ATGGACGAGCTGTACAAGGGATCCGGTATGAACCAAAAATTCATA<br>CTGGGACTGGCCATCGGAATCACCAGCGTGGGCTACGGCCTGATC<br>GACTACGAGACAAAGAATATCATCGATGCCGGCGTTAGACTGTTC

	CCCGAGGCCAACGTGGAAAACAACGAGGGAAGAAGGTCCAAACG<br>TGGAAGCAGAAGACTGAAGCGACGCCGCATTCACAGACTTGAACG<br>GGTGAAGAAGCTGCTCGAGGATTATAATCTGCTGGATCAGTCCCA<br>GATTCCTCAGTCTACAAACCCCTACGCCATCCGCGTGAAGGGCCTG<br>TCTGAAGCCCTGAGCAAGGACGAACTCGTGATTGCCCTGCTCCATA<br>TCGCCAAGAGAAGAGGCATCCACAAGATCGACGTGATCGACAGCA<br>ACGACGACGTGGGGAACGAGCTCAGCACCAAGGAACAGCTGAAT<br>AAGAACAGCAAGCTGCTGAAAGACAAATTTGTGTGCCAGATCCAG<br>CTGGAAAGAATGAATGAGGGCCAGGTGCGGGGAGAGAAAAACCG<br>GTTCAAGACCGCTGATATCATCAAGGAAATCATCCAGCTGCTGAA<br>TGTGCAGAAGAACTTCCACCAGCTGGACGAGAACTTCATCAACAA<br>GTACATCGAACTGGTTGAGATGAGGCGGGAATACTTCGAGGGCCC<br>CGGCAAGGGCAGTCCATATGGCTGGGAAGGCGACCCTAAGGCTTG<br>GTACGAGACACTGATGGGCCACTGCACCTACTTCCCAGATGAGCT<br>GAGAAGCGTGAAATACGCCTACAGTGCCGACCTGTTCAACGCTCT<br>GAACGACCTGAACAACCTGGTCATCCAAAGAGATGGACTGTCTAA<br>GCTCGAGTATCATGAGAAGTATCACATCATCGAGAACGTGTTCAA<br>GCAGAAGAAGAAACCTACACTGAAGCAGATCGCCAATGAGATCA<br>ATGTCAACCCTGAAGATATCAAGGGCTACAGAATCACAAAGTCTG<br>GCAAGCCCCAGTTTACCGAGTTTAAGCTCTACCACGACCTGAAAA<br>GCGTGCTGTTTGACCAGAGCATCCTGGAGAACGAAGACGTGCTGG<br>ACCAGATCGCTGAGATCCTGACCATCTACCAGGACAAGGATAGCA<br>TCAAATCTAAGCTGACGGAACTGGACATCCTGCTGAACGAGGAAG<br>ATAAGGAAAACATCGCCCAGCTGACTGGCTACACCGGGACCCACC<br>GGCTCAGCCTGAAATGCATCCGGCTGGTCCTGGAAGAGCAGTGGT<br>ATTCTAGCCGGAATCAGATGGAAATCTTCACACACCTGAACATTA<br>AGCCTAAGAAGATCAACCTGACAGCCGCCAACAAGATCCCGAAGG<br>CTATGATCGACGAGTTCATCCTGAGCCCTGTGGTGAAGAGGACCTT<br>CGGCCAGGCCATTAACCTTATTAACAAGATCATAGAAAAGTACGG<br>CGTGCCTGAAGATATCATCATCGAGCTGGCCAGAGAAAATAATAG<br>CAAGGACAAGCAGAAGTTCATCAATGAGATGCAGAAAAAGAACG<br>AGAACACCAGAAAGAGAATTAACGAAATCATCGGCAAGTATGGC<br>AACCAGAACGCCAAGAGACTGGTCGAGAAGATTAGACTGCACGAC<br>GAGCAGGAGGGCAAGTGCCTGTACTCACTGGAAAGCATCCCTCTG<br>GAGGACCTGCTGAACAACCCCAACCACTACGAGGTGGACCACATC<br>ATTCCAAGATCTGTGTCCTTCGACAACTCTTACCACAACAAAGTGC<br>TCGTGAAGCAGAGCGAGAACTCCAAAAAATCCAACCTGACCCCTT<br>ACCAGTACTTTAACAGCGGCAAGTCCAAGCTCTCTTACAACCAGTT<br>TAAACAACACATCCTGAACCTGAGCAAGTCCCAGGATAGAATCAG<br>CAAAAAAAAGAAAGAGTATCTGCTGGAAGAACGGGACATCAACA<br>AGTTCGAGGTGCAAAAAGAGTTCATCAATAGAAACCTGGTGGATA<br>CCCGGTACGCCACAAGAGAGCTGACAAACTACCTGAAGGCCTACT<br>TCAGCGCCAACAATATGAACGTGAAGGTGAAAACGATCAACGGCA<br>GCTTCACCGATTACCTGCGGAAAGTGTGGAAGTTTAAGAAGGAAC<br>GGAACCACGGCTACAAGCACCACGCCGAGGACGCCCTGATTATCG<br>CTAATGCCGATTTCCTGTTCAAAGAGAACAAGAAGCTGAAAGCCG

	TGAACTCTGTGCTGGAAAAACCTGAGATCGAGAGCAAGCAGCTGG<br>ATATCCAGGTGGATAGCGAGGATAACTACAGCGAAATGTTCATCA<br>TCCCTAAGCAGGTCCAGGACATCAAGGACTTCAGAAACTTCAAGT<br>ACAGCCACAGAGTGGACAAGAAGCCTAACAGACGGCTGATCAAC<br>GATACACTGTACAGCACCCGGAAGAAGGACAACTCCACCTACATC<br>GTGCAGACCATCAAAGATATCTATGCCAAAGATAATACCACCCTG<br>AAGAAGCAGTTTGACAAGTCACCCGAGAAGTTCCTCATGTACCAA<br>CACGATCCGCGGACCTTCGAGAAGTTGGAAGTGATCATGAAGCAG<br>TACGCTAATGAGAAGAATCCTCTGGCCAAGTACCACGAGGAAACA<br>GGCGAGTACCTGACCAAATACAGCAAAAAAAACAACGGCCCTATC<br>GTGAAAAGCCTGAAGTACATTGGAAACAAGCTGGGCCGCCACCTA<br>GATGTGACCCACCAGTTCAAGAGCAGCACCAAGAAGTTGGTGAAG<br>CGGAGCATCAAGCCTTATAGATTCGACGTCTACCTGACCGACAAG<br>GGATATAAGTTCATCACCATCAGCTACCTGGACGTGCTGAAGAAA<br>GACAATTACTACTACATACCCGAACAGAAGTACGACAAGCTCAAA<br>CTGGGCAAGGCCATCGACAAAAACGCCAAGTTTATCGCTAGCTTC<br>TACAAGAATGATCTGATCAAGCTGGACGGCGAGATCTACAAGATC<br>ATCGGCGTGAGTAGCGACACCAGAAACATGATCGAACTGGATCTG<br>CCTGACATCAGATACAAAGAATACTGCGAGCTGAACAATATCAAG<br>GGCAAACCTAGAATCATAAAGACCATCGGCAAAAAGGTGAATAGC<br>ATCGAAAAACTGACAACCGACGTGCTGGGCAACGTGTTCACCAAC<br>ACCCAGTACACAAAACCTCAGCTGCTGTTCAAGCGAGGAAATGCC<br>TATCCCTATGACGTGCCCGATTATGCCAGCCTGGGCAGCGGCTCCC<br>CCAAGAAAAAACGCAAGGTGGAAGATCCTAAGAAAAAGCGGAAA<br>GGTTCTAGTGAAACCCCGGGAACAAGTGAGTCGGCCACCCCTGAA<br>GGTGGATCAGGGGGTAGCGGATCCTCTTACGATAATTATGTTACG<br>ATTCTGGACGAGGAAACCTTAAAGGCTTGGATCGCTAAATTAGAG<br>AAGGCTCCTGTTTTCGCTTTCGACACGGAAACGGATTCTCTGGACA<br>ATATTAGTGCGAATCTTGTTGGTCTGAGTTTCGCAATTGAACCGGG<br>TGTTGCTGCTTACATCCCTGTGGCACACGACTACCTGGACGCTCCG<br>GACCAGATTTCACGTGAACGCGCTCTGGAACTGCTGAAGCCTTTAT<br>TAGAGGACGAGAAAGCTTTGAAAGTTGGTCAGAATTTGAAGTATG<br>CTCGTGGAATCTTAGCTAATTATGGTATCGAGTTGCGCGGTATCGC<br>TTTCGACACGATGTTGGAATCTTATATCCTGAACTCTGTCGCTGGT<br>CGCCATGACATGGACTCTCTGGCTGAGCGCTGGCTGAAACATAAG<br>ACGATTACCTTCGAGGAAATCGCAGGAAAGGGTAAGAACCAGCTC<br>ACGTTCAATCAAATCGCTCTGGAGGAAGCTGGTCGCTATGCTGCTG<br>AGGACGCTGACGTTACTCTGCAACTGCACTTGAAGATGTGGCCTG<br>ACTTGCAGAAGCATAAGGGTCCACTGAATGTTTTTGAAAACATTG<br>AGATGCCTTTGGTTCCAGTTCTGTCTCGTATCGAGCGCAATGGCGT<br>TAAAATTGACCCAAAGGTTTTACATAACCACTCAGAGGAACTGAC<br>GCTGCGCTTAGCCGAATTGGAGAAAAAGGCTCACGAGATCGCTGG<br>CGAAGAGTTCAATCTGTCATCTACGAAACAACTGCAGACTATCCTG<br>TTCGAGAAGCAAGGTATCAAGCCATTAAAAAAGACCCCTGGCGGT<br>GCTCCGTCTACCTCTGAGGAAGTTTTGGAGGAGTTAGCTTTGGATT<br>ACCCTCTGCCGAAGGTTATCTTGGAATACCGCGGTTTGGCTAAATT

	GAAGTCTACTTATACGGATAAACTTCCTTTGATGATTAATCCAAAG<br>ACGGGTCGCGTTCACACGTCGTACCATCAAGCTGTTACCGCTACCG<br>GTCGCCTGTCTTCTACGGATCCGAATTTACAGAATATTCCTGTGCG<br>CAATGAGGAGGGCCGCCGCATTCGTCAAGCTTTTATCGCTCCGGA<br>AGACTACGTTATCGTTTCTGCTGATTATTCTCAAAATGAATTACGT<br>ATCATGGCTCACCTGTCTCGCGATAAGGGTCTGTTGACGGCCTTTG<br>CTGAGGGTAAGGACATTCATCGTGCTACCGCTGCTGAGGTTTACGG<br>CCTGCCGTTGGAAACGGTTACGTCTGAACAGCGTCGCTCTGCTAAG<br>CGTATTAATTTCGGCTTAATCTACGGTATGTCTGCGTTTGGCTTAGC<br>TCGTCAGCTGAATATCCCGCGCAAGGAAGCTCAAAAATATATGGA<br>TCTGTATTTTGAGCGTTACCACGGTGTTTTGGAATACATGGAGCGT<br>ACGCGCGCGCAAGCTAAGGAACAAGGTTATGTGGAAACCTTGGAT<br>GGTCGTCGCTTGTACTTGCCTGACATTAAGTCTTCTAACGGCGCCC<br>GCCGCGCTGCTGCCGAGCGCGCTGCTATCAATGCTCCGATGCAAG<br>GTACTGCTGCTGATATTATTAAGCGTGCTATGATCGCTGTGGACGC<br>TTGGCTGCAAGCTGAACAGCCTCGCGTTCGCATGATTATGCAAGTT<br>CATGACGAGTTGGTTTTCGAGGTGCATAAGGACGACGTGGACGCT<br>GTTGCTAAACAAATCCACCAGTTGATGGAGAATTGCACGCGCTTA<br>GACGTTCCGCTGCTGGTTGAAGTTGGTTCTGGTGAAAACTGGGACC<br>AGGCTCACTAATGGCTGAAAACTGGGACCAGGCTCACTAACTCAG<br>ATCCTACTAGGTTTAATAAACATCTTTATTTTCATTACATCTGTGTG<br>TTGGTTTTTTGTGTGGTACCCAATTCGCCCTATAGTGAGTCGTATTA<br>CTCACTGGCCGTCGTTTTACAACGTCGTGACTGGGAAAACCCTGGC<br>GTTACCCAACTTAATCGCCTTGCAGCACATCCCCCTTTCGCCAGCT<br>GGCGTAATAGCGAAGAGGCCCGCACCGATCGCCCTTCCCAACAGT<br>TGCGCAGCCTGAATGGCGAATGGGACGCGCCCTGTAGCGGCGCAT<br>TAAGCGCGGCGGGTGTGGTGGTTACGCGCAGCGTGACCGCTACAC<br>TTGCCAGCGCCCTAGCGCCCGCTCCTTTCGCTTTCTTCCCTTCCTTT<br>CTCGCCACGTTCGCCGGCTTTCCCCGTCAAGCTCTAAATCGGGGGC<br>TCCCTTTAGGGTTCCGATTTAGTGCTTTACGGCACCTCGACCCCAA<br>AAAACTTGATTAGGGTGATGGTTCACGTAGTGGGCCATCGCCCTG<br>ATAGACGGTTTTTCGCCCTTTGACGTTGGAGTCCACGTTCTTTAAT<br>AGTGGACTCTTGTTCCAAACTGGAACAACACTCAACCCTATCTCGG<br>TCTATTCTTTTGATTTATAAGGGATTTTGCCGATTTCGGCCTATTGG<br>TTAAAAAATGAGCTGATTTAACAAAAATTTAACGCGAATTTTAAC<br>AAAATATTAACGCTTACAATTTAGGTGGCACTTTTCGGGGAAATGT<br>G<br><br>**Ampicillin resistance cassette-ColE1 origin of replication-hU6 promoter-gRNA sequence-SlugCas9 gRNA scaffold-CMV promoter-NLS-mCherry-GSGM- Slug-nCas9(D10A)-NLS-PolI5MΔ-stop-SV40 polyA**

References

1. Halperin, S. O. *et al.* CRISPR-guided DNA polymerases enable diversification of all nucleotides in a tunable window. *Nature* **560**, 248–252 (2018).
