# Supplementary material for: Nickase fidelity drives EvolvR-mediated diversification in mammalian cells
Source: Nat Commun. 2025 Apr 19;16:3723. doi: 10.1038/s41467-025-58414-0 (PMC12009436; doi:10.1038/s41467-025-58414-0)
Supplement: Supplementary file 2 — Description of Additional Supplementary Files [file 41467_2025_58414_MOESM2_ESM.pdf]

Description of Additional Supplementary Files

Supplementary Data 1: Oligonucleotide sequences
