# Supplementary material for: Nickase fidelity drives EvolvR-mediated diversification in mammalian cells
Source: Nat Commun. 2025 Apr 19;16:3723. doi: 10.1038/s41467-025-58414-0 (PMC12009436; doi:10.1038/s41467-025-58414-0)
Supplement: Supplementary file 4 — Reporting Summary [file 41467_2025_58414_MOESM4_ESM.pdf]

nature portfolio

Corresponding author(s): John E. Dueber, David V. Schaffer

Last updated by author(s): 03-09-2025

Reporting Summary

Nature Portfolio wishes to improve the reproducibility of the work that we publish. This form provides structure for consistency and transparency in reporting. For further information on Nature Portfolio policies, see our Editorial Policies and the Editorial Policy Checklist.

Statistics

For all statistical analyses, confirm that the following items are present in the figure legend, table legend, main text, or Methods section.

n/a	Confirmed	
☐	☒	The exact sample size (*n*) for each experimental group/condition, given as a discrete number and unit of measurement
☐	☒	A statement on whether measurements were taken from distinct samples or whether the same sample was measured repeatedly
☐	☒	The statistical test(s) used AND whether they are one- or two-sided<br>*Only common tests should be described solely by name; describe more complex techniques in the Methods section.*
☐	☒	A description of all covariates tested
☐	☒	A description of any assumptions or corrections, such as tests of normality and adjustment for multiple comparisons
☐	☒	A full description of the statistical parameters including central tendency (e.g. means) or other basic estimates (e.g. regression coefficient) AND variation (e.g. standard deviation) or associated estimates of uncertainty (e.g. confidence intervals)
☐	☒	For null hypothesis testing, the test statistic (e.g. *F*, *t*, *r*) with confidence intervals, effect sizes, degrees of freedom and *P* value noted<br>*Give P values as exact values whenever suitable.*
☒	☐	For Bayesian analysis, information on the choice of priors and Markov chain Monte Carlo settings
☒	☐	For hierarchical and complex designs, identification of the appropriate level for tests and full reporting of outcomes
☒	☐	Estimates of effect sizes (e.g. Cohen's *d*, Pearson's *r*), indicating how they were calculated

Our web collection on statistics for biologists contains articles on many of the points above.

Software and code

Policy information about availability of computer code

Data collection	*Provide a description of all commercial, open source and custom code used to collect the data in this study, specifying the version used OR state that no software was used.*
Data analysis	All tests for statistical significance were performed using GraphPad Prism version 10.4.0.

For manuscripts utilizing custom algorithms or software that are central to the research but not yet described in published literature, software must be made available to editors and reviewers. We strongly encourage code deposition in a community repository (e.g. GitHub). See the Nature Portfolio guidelines for submitting code & software for further information.

Data

Policy information about availability of data

All manuscripts must include a data availability statement. This statement should provide the following information, where applicable:

- Accession codes, unique identifiers, or web links for publicly available datasets
- A description of any restrictions on data availability
- For clinical datasets or third party data, please ensure that the statement adheres to our policy

Source data for all figures in this study are provided in the Source Data file. Next generation sequencing data used in this study are available in the NCBI SRA database under accession code PRJNA1157996.

Research involving human participants, their data, or biological material

Policy information about studies with human participants or human data. See also policy information about sex, gender (identity/presentation), and sexual orientation and race, ethnicity and racism.

Reporting on sex and gender	NA
Reporting on race, ethnicity, or other socially relevant groupings	NA
Population characteristics	NA
Recruitment	NA
Ethics oversight	NA

Note that full information on the approval of the study protocol must also be provided in the manuscript.

Field-specific reporting

Please select the one below that is the best fit for your research. If you are not sure, read the appropriate sections before making your selection.

[x] Life sciences   [ ] Behavioural & social sciences   [ ] Ecological, evolutionary & environmental sciences

For a reference copy of the document with all sections, see nature.com/documents/nr-reporting-summary-flat.pdf

Life sciences study design

All studies must disclose on these points even when the disclosure is negative.

Sample size	No sample-size calculations were performed prior to experiments. Biological triplicates were used to enable high-throughput characterization of new gRNAs and EvolvR engineering improvements while affording sufficient statistical power to determine statistical significance for large effect sizes.
Data exclusions	Next generation sequencing base calls of quality score less than 30 were excluded from analysis to minimize the influence of base calling errors on variant calling.
Replication	BFP to GFP mutation assays were all successfully reproduced at least once. Sequencing readouts were not reproduced due to the cost-prohibitive nature of performing repeated next-generation sequencing runs.
Randomization	Cells within a cell culture were well-mixed prior to seeding and therefore were allocated randomly into each of the different biological conditions used in this study. Moreover, randomization is not relevant to this study as all experiments were performed on largely genetically identical cell lines.
Blinding	The data in this study was collected automatically by randomly sampling from populations of cells using flow cytometry or next-generation sequencing of gDNA. Accordingly, blinding the investigator to experimental group allocation would not influence the conclusions of this study.

Reporting for specific materials, systems and methods

We require information from authors about some types of materials, experimental systems and methods used in many studies. Here, indicate whether each material, system or method listed is relevant to your study. If you are not sure if a list item applies to your research, read the appropriate section before selecting a response.

Materials & experimental systems

n/a	Involved in the study	
x		Antibodies
	x	Eukaryotic cell lines
x		Palaeontology and archaeology
x		Animals and other organisms
x		Clinical data
x		Dual use research of concern
x		Plants

Methods

n/a	Involved in the study	
x		ChIP-seq
	x	Flow cytometry
x		MRI-based neuroimaging

Eukaryotic cell lines

Policy information about cell lines and Sex and Gender in Research

Cell line source(s)	A375 and HEK293T cells were purchased from the UC Berkeley Cell Culture facility. BFP HEK293 cells were donated by Jacob Corn's lab. All cell lines were derived from female human cells.
Authentication	We did not authenticate any of the cell lines used.
Mycoplasma contamination	A375 and HEK293T cells were obtained from the UC Berkeley Cell Culture Facility, which screens cell lines for mycoplasma prior to storing them by fluorescence microscopy of a Hoescht stain. BFP HEK293 cells were tested for mycoplasma using the Lonza MycoAlert micoplasma detection kit by Lonza and were not detectably contaminated.
Commonly misidentified lines (See ICLAC register)	HEK cells are used in this study owing to their ease of transfection and genetic engineering.

Plants

Seed stocks	NA
Novel plant genotypes	NA
Authentication	NA

Flow Cytometry

Plots

Confirm that:

- [x] The axis labels state the marker and fluorochrome used (e.g. CD4-FITC).
- [x] The axis scales are clearly visible. Include numbers along axes only for bottom left plot of group (a 'group' is an analysis of identical markers).
- [x] All plots are contour plots with outliers or pseudocolor plots.
- [x] A numerical value for number of cells or percentage (with statistics) is provided.

Methodology

Sample preparation	Cells were trypsinized and resuspended in 300 µL PBS.
Instrument	An Attune NxT was used for experiments not requiring sorting. A Sony SH800Z was used for experiments requiring cell sorting and for some experiments not requiring cell sorting.
Software	FlowJo V10.10.0
Cell population abundance	Purity of cells post-sorting was not measured. Cells were collected using the "Normal" setting for sorting on a Sony SH800Z sorter.
Gating strategy	The frequency of GFP positive cells was determined by gating such that there were zero GFP positive cells detected in an untransfected BFP HEK293 population of unimodal background GFP fluorescence. The specific cutoff for GFP positive cells was determined on a per-experiment basis according to the criterion above using the parental BFP HEK293 cells used in the transfection and, prior to the experiment, observed under a microscope to contain no GFP positive cells.

- [x] Tick this box to confirm that a figure exemplifying the gating strategy is provided in the Supplementary Information.
